# 5‐HT_3_ receptor antagonists for preventing postoperative nausea and vomiting after gynecological surgery: A systematic review and network meta‐analysis

**DOI:** 10.1002/ijgo.70197

**Published:** 2025-05-09

**Authors:** Hongxia Xu, Lingyan Rong, Shaohui Yang, Jiankun Xing, Huajun Dong, Huihui Liu, Xiaotao Chen, Lingyan Liu

**Affiliations:** ^1^ Department of Clinical Pharmacy Wendeng Hospital of Traditional Chinese Orthopedics and Traumatology of Shandong Province Weihai China

**Keywords:** 5‐HT_3_ antagonists, gynecological surgery, PONV

## Abstract

**Background:**

Gynecological surgery is generally associated with a high risk of postoperative nausea and vomiting (PONV), for which a combination of antiemetic therapies is advised, but adherence to these protocols is often low. Given the current reality, a preferred 5‐HT_3_ receptor antagonist for preventing PONV as a result of gynecological operations might be desirable. However, the efficiency of different 5‐HT_3_ receptor antagonists in gynecological operations was not clear.

**Objective:**

To assess the effectiveness of different 5‐HT_3_ antagonists in preventing PONV after gynecological surgery.

**Search Strategy:**

Electronic databases, including PubMed, Embase, the Cochrane Library, and Web of Science, were searched for randomized clinical trials (RCTs) from their inception up to September 20, 2023.

**Selection Criteria:**

Patients who received only 5‐HT_3_ antagonists to prevent nausea and vomiting following gynecologic surgical procedures were included. Only RCT articles and English language literature were included.

**Data Collection and Analysis:**

Two investigators independently assessed the study quality and performed data extraction. R software and STATA 17 were used for this network meta‐analysis to compare treatments using a frequentist approach.

**Main Results:**

Palonosetron demonstrated superior efficacy compared with ondansetron, with a significant difference in “acute nausea,” “overall nausea,” “acute vomiting,” “late vomiting,” “late PONV,” “overall PONV,” “late rescue medicine” and “>24 h rescue medicine.” There was a significant difference between palonosetron and ramosetron in “acute nausea,” between ramosetron and ondansetron in “>24 h nausea,” and between granisetron and ondansetron in “late vomiting.” Additionally, granisetron and palonosetron are generally ranked higher in the P‐score system.

**Conclusions:**

In gynecological surgery, palonosetron demonstrated superior efficacy to ondansetron. Granisetron seemed to be the most effective alternative to palonosetron in our study.

## 1 INTRODUCTION

Postoperative nausea and vomiting (PONV) is a common and distressing complication that can lead to a range of issues, from fluid–electrolyte imbalances to pulmonary aspiration and delayed recovery.^1^ In adults, factors such as being female, having a history of motion sickness, and not smoking can heighten the risk of PONV. Surgeries such as laparoscopies, bariatric procedures, and gynecological operations are known to elevate the risk of PONV.^2^ Gynecological surgery is associated with a high risk of PONV, with a frequency ranging between 20% and 60%.^3^, ^4^

A combination of two or more antiemetic therapies targeting the different pathways of antiemetic therapy for patients at high risk for PONV was recommended.^2^, ^5^ However, compliance with PONV prevention protocols among these patients remains very poor in some places, and a substantial number of patients did not receive the recommended prophylactic treatments.^6^, ^7^, ^8^, ^9^, ^10^, ^11^, ^12^, ^13^ In a retrospective study of patients undergoing laparoscopic gynecological surgery, only 21% of patients received PONV prophylaxis.^7^ In another study, only 37% of moderate‐ and high‐risk patients received the specified prophylaxis.^8^ There was also evidence that 85.7% of high‐risk patients were given a smaller number of preventive agents than recommended by standard guidelines.^9^ Compared with white patients, black patients with high‐risk PONV showed a 43% lower adherence rate to the protocols.^10^.

There were many reasons for the low adherence to guidelines among high‐risk PONV patients: concerns from the anesthesiologists about the unnecessary adverse effects of complex drugs used in anesthesia^11^; the infrequent use of PONV prophylaxis protocols or risk stratification in developing countries^14^, ^15^, ^16^; inconsistent availability of antiemetic prophylactic medications^16^; patient reluctance to financially invest in antiemetic therapy,^17^ and so on.

With regard to PONV prevention, 5‐HT_3_ receptor antagonists were widely used as the first line of defense owing to their proven efficacy and fewer side effects.^2^, ^18^ For gynecological surgery, it is necessary to take a variety of measures to encourage physicians to adhere to the guidelines regarding the recommended combinations, but given the current reality, a preferred 5‐HT_3_ receptor antagonist for preventing PONV as a result of gynecological operations might be desirable. However, the relative effectiveness of these antagonists in gynecological surgery remains controversial. Palonosetron has often been preferred over other 5‐HT_3_ receptor antagonists for PONV prevention, but some studies present conflicting outcomes.^19^, ^20^, ^21^ There has been some debate over the effectiveness of different first‐generation 5‐HT_3_ receptor antagonists. Some studies showed no significant difference between ramosetron and ondansetron, and others suggested that ramosetron may be more effective.^22^, ^23^, ^24^

Due to these discrepancies and to the need for clarity in clinical practice, we designed this network meta‐analysis to assess the efficacy of various 5‐HT_3_ antagonists in preventing PONV after gynecological surgery. Using the evidence available, we aim to guide healthcare providers in improving postoperative recovery.

## 2 METHODS

This systematic review and network meta‐analysis was registered with the International Prospective Registry of Systematic Reviews (PROSPERO) under the identifier CRD42024496745.

### 2.1 Study selection

We included randomized controlled trials (RCTs) published in English and reported relevant outcomes. A comprehensive search was conducted across several databases, including PubMed, Embase, the Cochrane Library, and Web of Science, from their inception up to September 20, 2023. The search strategy incorporated Medical Subject Heading (MeSH) terms and free‐text words, encompassing “gynecologic surgical procedures,” “postoperative nausea and vomiting,” “ondansetron,” “granisetron,” “dolasetron,” “tropisetron,” “ramosetron,” “azasetron,” and “palonosetron.” The detailed search strategy is outlined in Data S1.

#### Inclusion and exclusion criteria

Patients who received 5‐HT_3_ antagonists to prevent nausea and vomiting following gynecologic surgical procedures were included in the study, whereas patients who used antiemetic medications other than 5‐HT3 antagonists were excluded. Only RCTs articles were included and only English‐language literature.

#### Outcome measures

The outcome measures were as follows:

- Nausea: “acute nausea,” “late nausea,” “>24 h nausea,” and “overall nausea.”
- Vomiting: “acute vomiting,” “late vomiting,” “>24 h vomiting,” and “overall vomiting.”
- PONV: “acute PONV,” “Late PONV,” “>24 h PONV,” and “overall PONV.”
- Rescue medicine: “acute rescue medicine,” “late rescue medicine,” “>24 h rescue medicine,” and “overall rescue medicine.”
- Adverse reaction.

“Acute” and “late” periods were defined as follows—when the first 24 h post‐surgery were divided into two time periods, the first was defined as the “acute” period, and the second as the “late” period. If the first 24 h post‐surgery were divided into three or more parts, the time period (≥2 h or combination should be considered if ≤2 h) before 2–6 h was defined as the “acute” period, and the time period after 6 h as the “late” period. “>24 h” was defined as more than 24 h postoperatively and “overall” period was defined as the whole recorded time in articles.

#### Study identification

We screened the literature based on the inclusion and exclusion criteria mentioned earlier. Two authors screened titles and abstracts for eligibility. Full texts of potentially relevant studies were retrieved and further assessed for inclusion.

### 2.2 Risk of bias assessment and data extraction

The risk of bias assessment of all included studies was evaluated using the Cochrane Handbook for Systematic Reviews of Interventions,^25^ which assessed key indicators, including sequence generation, allocation concealment, blinding of participants and outcome assessors, and completeness or selectivity of outcome reporting.

Data extracted from the selected studies included the author's name, publication year, study design, sample size, patient age, surgical methods, anesthesia techniques, postoperative analgesia approaches, interventional drugs, and the outcomes of interest.

Two investigators independently assessed the study quality and performed data extraction. When consensus could not be reached, the third author resolved the discrepancies.

### 2.3 Statistical analysis

R software (version 4.2.1) and STATA 17 were used for this network meta‐analysis to compare treatments using a frequentist approach. The conclusions from direct and indirect comparisons were obtained. Heterogeneity in indirect comparison meta‐analysis was quantified using the *I* ^2^ statistic, while the *Q* statistic was used to assess inconsistencies between direct and indirect effects. A random‐effects model was applied to pool data. Outcomes were reported as risk ratios (RRs) with 95% confidence intervals (CIs), and statistical significance was set at *P* values less than 0.05.

P‐scores were utilized to rank the probability of effectiveness for each intervention. The transitivity assumption was assessed by comparing the distribution of potential effect modifiers across comparisons, such as publication years, mean age, weight, duration of surgery, and anesthesia, etc. Sensitivity analyses were performed by separately excluding studies involving open surgery, non‐opioid postoperative anesthesia, spinal anesthesia. or propofol maintenance anesthesia. Egger's test and funnel plots were employed to assess publication bias in studies with 10 or more trials.

## 3 RESULTS

**FIGURE 1:**
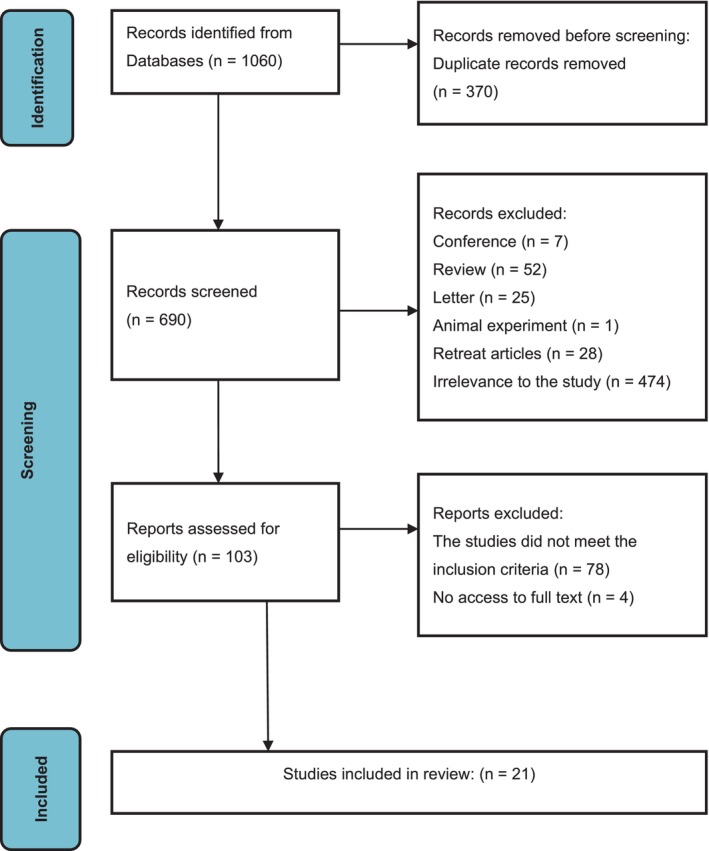
Flow diagram of literature screening and selection process.

A comprehensive search yielded a total of 1060 documents. After the elimination of duplicates and screening of articles that did not meet the inclusion criteria based on their titles and abstracts, 103 studies underwent a full‐text review. Ultimately, 21 studies were deemed eligible for inclusion in this network meta‐analysis (Figure 1).^21^, ^23^, ^26^, ^27^, ^28^, ^29^, ^30^, ^31^, ^32^, ^33^, ^34^, ^35^, ^36^, ^37^, ^38^, ^39^, ^40^, ^41^, ^42^, ^43^, ^44^ The collective dataset encompassed 1959 female participants, with ages ranging from 26.4 to 52.5 years. All 21 RCT studies vwere published between 1999 and 2023. A summary of the characteristics of each study is presented in Table 1.

**TABLE 1:** Characteristics of included randomized controlled trials (RCTs).

Number	Year	Test type	Included population	ASA	Anesthesia methods (major maintained medicine)	Surgical methods	Intervention (number in each group)	Postoperative analgesia	Period definition (h)			Outcome
									Acute	Late	>24 h	
Das S 2022	2022	RCT	100 female patients, aged 18–60 years, gynecological surgery.	I–II	General anesthesia (sevoflurane)	Laparoscopic surgery	Ramosteron (50) Ondansetron (50)	Diclofenac paracetamol	0–6	6–24	/	⑦⑧⑪⑫⑬⑭⑯⑰
Bandyopadhyay D 2022	2022	RCT	90 female patients, aged 35–60 years, gynecological surgery.	I–II	General anesthesia	/	Granisetron (30) Palanosetron (30) Ondansetron (30)	Tramadol Diclofenac	0–4	4–24	/	①②④⑤⑦⑧⑩⑪⑫⑬⑭⑯⑰
Balyan R 2022	2022	RCT	130 female patients, aged 18–70 years, gynecological surgery.	I–II	General anesthesia (sevoflurane)	Laparoscopic surgery	Ondansetron (65) Palonosetron (65)	Morphine Paracetamol Diclofenac	0–2	2–24	24–48	①②③④⑤⑥⑦⑧⑨⑪⑬⑭⑮
Yadav M 2022	2022	RCT	90 female patients, ≥21 years old, gynecological surgery.	I–II	General anesthesia (sevoflurane)	Laparoscopic surgery	Palonosetron (45) Ondansetron (45)	Fentanyl	0–2	6–24	/	①②④⑤⑦⑧⑩⑪⑫⑯⑰
Lee WS 2015	2015	RCT	105 female patients, laparoscopic hysterectomy.	I–II	General anesthesia (sevoflurane)	Laparoscopic surgery	Palonosetron (35) Ramosetron (35) Granisetron (35)	Diclofenac	0–6	6–24	24–48	①②③④⑤⑥⑫⑬⑭⑮
Kim SH 2015	2015	RCT	200 patients, gynecological surgery	/	General anesthesia (sevoflurane)	Laparoscopic surgery	Palonosetron (44) Ramosetron (44)	Ketorolac Fentanyl	Before discharge from PACU	24 h after discharge from PACU	48 h after discharge from PACU	⑦⑧⑨
Park SK 2013	2013	RCT	100 female patients, ≥20 years old, gynecological surgery	I–II	General anesthesia (sevoflurane)	Laparoscopic surgery	Ramosetron (50) Palonosetron (50)	Fentanyl	0–6	6–24	24–48	①②③④⑤⑥⑦⑧⑨⑩⑪⑫⑯⑰
Kim YY 2013	2013	RCT	100 female patients, ≥18 years old, gynecological surgery	I–II	General anesthesia (sevoflurane)	Laparoscopic surgery	Ondansetron (50) Palonosetron (50)	Fentanyl Ketorolac	0–2	2–24	24–72	①②③④⑤⑥⑦⑧⑨⑩⑪⑫⑬⑭⑮⑯⑰
Daria U 2012	2012	RCT	180 female patients, aged 18–55 years, gynecological surgery	I–II	General anesthesia	Laparoscopic surgery	Ondasetron (30) Granisetron (30)	/	0–6	/	/	⑦⑪
Park SK 2011	2011	RCT	Patients ≥21 years old, gynecological surgery	I–II	General anesthesia (sevoflurane)	Laparoscopic surgery	Ondansetron (45) Palonosetron (45)	Fentanyl	0–2	6–24	/	①②④⑤⑦⑧⑩⑪⑫⑯⑰
Bajwa SS 2011	2011	RCT	60 Patients, aged 25–40 years, ligation surgery	I–II	General anesthesia (propofol and halothane)	Laparoscopic surgery	Ondansetron (30) Palonosetron (30)	Diclofenac	0–6	6–12	24–72	①②③④⑤⑥⑪⑯⑰
Kim SI 2009	2009	RCT	162 female patients, aged 21–71 years, gynecological surgery	/	General anesthesia (sevoflurane)	Laparoscopic and open surgery	Ramosetron (54) Ondansetron (54)	Fentanyl	0–6	6–24	/	①②④⑤⑩⑪⑫⑬⑭⑯⑰
Bhatia N 2008	2008	RCT	120 female patients, aged 18–40 years, gynecological surgery	I–II	General anesthesia (propofol)	Laparoscopic surgery	Ondansetron (30) Granisetron (30)	Ketorolac tromethamine	/	/	/	⑩⑪⑫⑯⑰
Yun MJ 2010	2010	RCT	98 female patients, aged 20–65 years, gynecological surgery	I–II	General anesthesia (sevoflurane)	Laparoscopic surgery	Ondansetron (49) Azasetron (49)	Nalbuphine Ketorolac	0–6	6–12	24–48	①②③④⑤⑥⑪⑫⑬⑭⑮⑯⑰
Ekinci O 2011	2011	RCT	Female patients, aged 20–72 years, total abdominal hysterectomy	I–III	General anesthesia (sevoflurane)	Open surgery	Tropisetron (20) Ondansetron (20)	Dipyrone	/	/	/	⑩⑪⑰
Patil SB 2023	2023	RCT	100 female patients, aged 23–65 years, hysterectomy	/	Spinal anesthesia	/	Ondansetron (50) Ramosetron (50)	/	0–6	6–24	/	①②③④⑤⑥⑩⑪⑫⑬⑭⑮
Tarigonda S 2021	2021	RCT	Female patients, aged 35–70 years, abdominal hysterectomy	I–II	General anesthesia (sevoflurane)	Open surgery	Ondansetron (30) Ramosetron (30) Palonosetron (30)	/	0–6	12–24	/	①②④⑪⑬
Tsui SL 1999	1999	RCT	121 women, gynecological Laparotomy.	I–II	General anesthesia (sevoflurane)	Open surgery	Tropisetron (37) Ondansetron (39)	Morphine	0–12	/	/	⑩⑬⑯⑰
Bridges JD 2006	2006	RCT	194 female patients, aged 18–78 years, gynecological and breast surgery	/	Most general anesthesia (sevoflurane)	Laparoscopic and open surgery	Dolasetron (66) Granisetron (62) Ondansetron (66)	/	0–6	6–24	/	⑦⑧⑪⑫
Lee JW 2011	2011	RCT	120 women, aged 18–60 years, abdominal hysterectomy	I–II	General anesthesia (sevoflurane)	Open surgery	Ramosetron (60) Ondansetron (60)	Fentanyl Ketorolac	0–2	2–24	24–48	①②③④⑤⑥⑦⑧⑨⑪⑬⑭⑮
Sumitha CS 2021	2021	RCT	120 female patients, aged 18–60 years, lower abdominal surgery	I–II	General anesthesia (halothane)	Open surgery	Granisetron (30) Ondansetron (30)	Paracetamol Ketorolac	/	6–24	/	②⑤⑧⑪⑫⑯⑰

*Note*: ① acute nausea; ② late nausea; ③ >24 h nausea; ④ overall nausea; ⑤ acute vomiting; ⑥ late vomiting; ⑦ >24 h vomiting; ⑧ overall vomiting; ⑨ acute PONV; ⑩ late PONV; ⑪ >24 h PONV; ⑫ overall PONV; ⑬ acute rescue medicine; ⑭ late rescue medicine; ⑮ >24 h rescue medicine; ⑯ overall rescue medicine; ⑰ adverse reaction.

Abbreviation: PACU, post‐anesthesia care unit; PONV, postoperative nausea and vomiting..

Generally, data extraction was performed directly from the articles. For the calculation of PONV, some data were obtained from the “complete response (CR)”.^21^, ^37^, ^40^, ^43^ Some data regarding the use of rescue medication were derived from the failure rates reported in the literature.^37^

**FIGURE 2:**
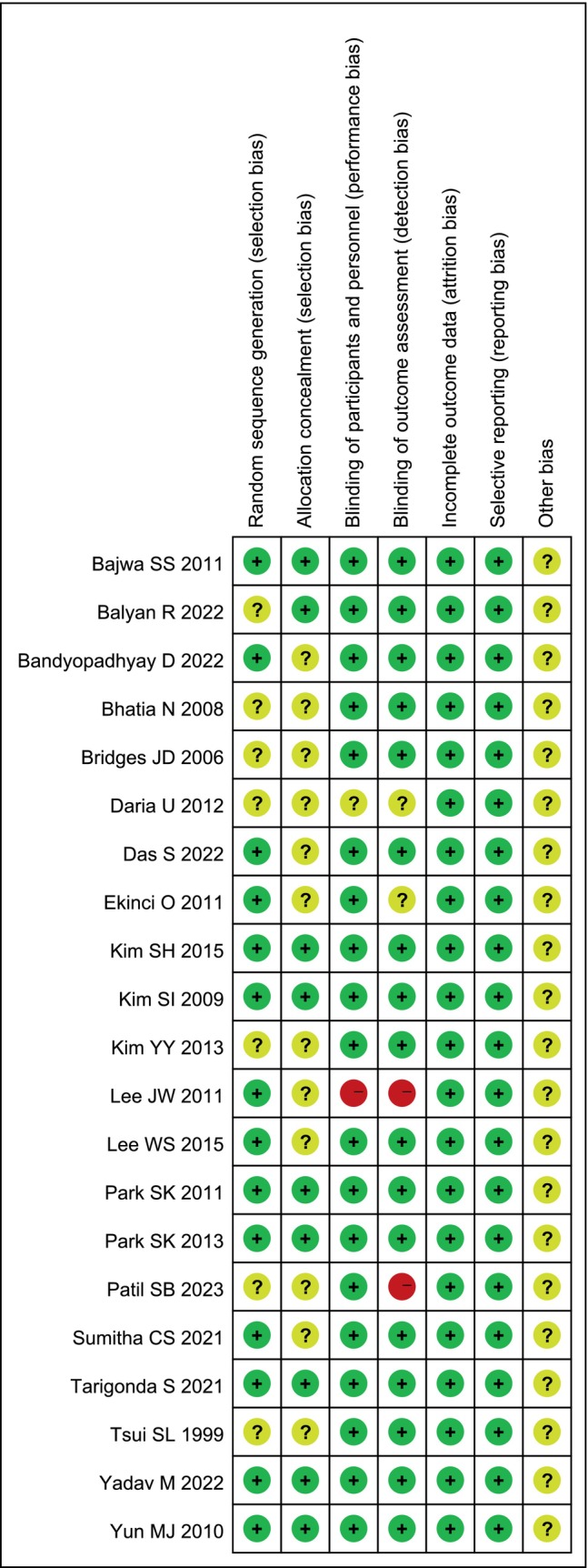
Graph of risk of bias.

Twenty‐one trials were included for quality evaluation, which was done using Revman 5.3 (Figure 2).

We observed low heterogeneity between studies in this network (Data S2) and no significant inconsistencies among direct and indirect comparisons (Data S3).

The funnel plot of the studies with 10 or more trials all demonstrated a fundamental symmetry, suggesting the absence of publication bias in the reviewed articles (Data S4).

### 3.1 Nausea

For “acute nausea,” 13 RCTs (Data S5) included in the analysis showed that palonosetron was significantly more effective than ondansetron (RR 1.48, 95% CI 1.17–1.87) and ramosetron (RR 0.77, 95% CI 0.60–0.99) (Table 2). Based on the P‐score, palonosetron was associated with the highest efficacy, followed by granisetron, azasetron, ramosetron, and ondansetron (Table 3).

**TABLE 2:** Network calculation of nausea.

Acute nausea	Azasetron	—	0.82 (0.37; 1.80)	—	—
	1.40 (0.41; 4.74)	Granisetron	0.20 (0.01; 4.00)	0.84 (0.30; 2.41)	0.83 (0.28; 2.48)
	0.82 (0.37; 1.80)	0.58 (0.23; 1.48)	Ondansetron	1.47 (1.13; 1.92)	1.14 (0.89; 1.46)
	1.21 (0.53; 2.75)	0.87 (0.34; 2.18)	1.48 (1.17; 1.87)	Palanosetron	0.82 (0.63; 1.09)
	0.93 (0.41; 2.11)	0.67 (0.26; 1.68)	1.14 (0.91; 1.43)	0.77 (0.60; 0.99)	Ramosetron
Late nausea	Azasetron	—	0.88 (0.42; 1.81)	—	—
	1.29 (0.41; 4.01)	Granisetron	0.41 (0.12; 1.41)	1.71 (0.50; 5.87)	1.00 (0.25; 3.93)
	0.88 (0.42; 1.81)	0.68 (0.28; 1.63)	Ondansetron	1.49 (1.06; 2.09)	0.85 (0.53; 1.37)
	1.17 (0.53; 2.58)	0.91 (0.37; 2.20)	1.33 (0.98; 1.82)	Palanosetron	1.14 (0.63; 2.04)
	0.96 (0.42; 2.19)	0.75 (0.30; 1.83)	1.10 (0.75; 1.61)	0.82 (0.54; 1.24)	Ramosetron
>24h nausea	Azasetron	—	1.50 (0.57; 3.92)	—	—
	3.93 (0.33; 46.66)	Granisetron	—	0.50 (0.05; 5.28)	1.00 (0.06; 15.40)
	1.50 (0.57; 3.92)	0.38 (0.04; 3.73)	Ondansetron	1.29 (0.79; 2.11)	6.37 (1.46; 27.75)
	2.20 (0.75; 6.40)	0.56 (0.06; 5.23)	1.47 (0.92; 2.34)	Palanosetron	1.14 (0.59; 2.20)
	3.13 (0.94; 10.38)	0.80 (0.08; 7.66)	2.09 (1.02; 4.28)	1.42 (0.78; 2.61)	Ramosetron
Overall nausea	Azasetron	—	0.84 (0.46;1.52)	—	—
	2.22 (0.79;6.23)	Granisetron	0.35 (0.15; 0.82)	3.00 (0.32; 28.31)	—
	0.84 (0.46;1.52)	0.38 (0.16; 0.88)	Ondansetron	1.60 (1.14;2.25)	1.05 (0.68; 1.62)
	1.24 (0.63;2.41)	0.56 (0.23; 1.36)	1.47 (1.09; 1.99)	Palanosetron	0.97 (0.59; 1.59)
	1.01 (0.51;2.02)	0.46 (0.18; 1.13)	1.20 (0.85; 1.71)	0.82 (0.57; 1.18)	Ramosetron
	1.02 (0.46;2.29)	0.46 (0.17; 1.26)	1.22 (0.71; 2.10)	0.83 (0.44; 1.54)	1.01 (0.53; 1.94)

**TABLE 3:** P‐score of outcome measures.

	Palanosetron	Granisetron	Ramosetron	Ondansetron	Azasetron	Tropisetron	Dolasetron
Acute nausea	0.7590	0.7506	0.3796	0.1417	0.4691		
Late nausea	0.7138	0.7000	0.3945	0.2264	0.4653		
>24 h nausea	0.5756	0.7327	0.8101	0.2694	0.1121		
Overall nausea	0.6818	0.9424	0.4062	0.1379	0.4026	0.4292	
Acute vomiting	0.6130	0.7129	0.3550	0.0882	0.7310		
Late vomiting	0.7188	0.9139	0.4606	0.2281		0.1785	
>24 h vomiting	0.7953	0.6329	0.6203	0.2859	0.1656		
Overall vomiting	0.6918	0.8543	0.1587	0.2547	0.5730	0.4675	
Acute PONV	0.7904	0.6574	0.4813	0.3950			0.1759
Late PONV	0.7811	0.5752	0.4975	0.2188			0.4275
>24 h PONV	0.6486		0.7423	0.1091			
Overall PONV	0.7029	0.6963	0.5156	0.2195	0.7205		0.1452
Acute rescue medicine	0.3804	0.7263	0.6276	0.3531	0.4125		
Late rescue medicine	0.7110	0.9163	0.3769	0.2054	0.2904		
>24 h rescue medicine	0.6322	0.6467	0.8098	0.2602	0.1510		
Overall rescue medicine	0.4545	0.8902	0.5639	0.1600		0.4315	
Adverse reaction	0.5890	0.2980	0.5040	0.4476	0.5626	0.5989	

For “late nausea,” the analysis of 14 RCTs (Data S5) found no statistically significant difference among all 5‐HT_3_ antagonists (Table 2), with the P‐score ranking consistent with that for “acute nausea” (Table 3).

In the case of “>24 h nausea,” ramosetron outperformed ondansetron in 8 RCTs (Data S5) (RR 2.09, 95% CI 1.02–4.28) (Table 2). According to the P‐score, ramosetron and azasetron were associated with the best and worst effects, respectively (Table 3).

Based on 12 RCTs (Data S5) included in “overall nausea,” palonosetron was significantly more effective than ondansetron (RR 1.47, 95% CI 1.09–1.99) (Table 2). According to the P‐score, granisetron emerged as the best choice, followed by palanosetron, tropisetron, ramosetron, azasetron, and ondansetron (Table 3).

### 3.2 Vomiting

In the analysis of “acute vomiting”, there was a significant difference in palonosetron's effectiveness over ondansetron (RR 2.04, 95% CI 1.14–3.66) based on 13 RCTs (Data S5). In accordance with P‐scores, azasetron appeared to have the most potential (Table 4), followed by granisetron, palanosetron, ramosetron, and ondansetron (Table 3).

**TABLE 4:** Network calculation of vomiting.

Acute vomiting	Azasetron	—	0.20 (0.01; 4.06)	—	—
	0.75 (0.02;30.38)	Granisetron	0.33 (0.01; 7.87)	0.52 (0.04;6.02)	0.33 (0.01; 7.91)
	0.20 (0.01; 4.06)	0.27 (0.03; 2.29)	Ondansetron	1.54 (0.79;3.01)	1.63 (0.99; 2.69)
	0.41 (0.02; 8.76)	0.54 (0.06; 4.71)	2.04 (1.14; 3.66)	Palanosetron	0.51 (0.24; 1.08)
	0.28 (0.01; 5.87)	0.37 (0.04; 3.22)	1.39 (0.87; 2.23)	0.68 (0.37; 1.27)	Ramosetron
Late vomiting	Azasetron	—	2.00 (0.19; 21.34)	—	—
	7.03 (0.49; 100.64)	Granisetron	0.26 (0.07; 0.99)	1.06 (0.16; 6.98)	1.00 (0.02; 49.04)
	2.00 (0.19; 21.34)	0.28 (0.08; 0.96)	Ondansetron	2.01 (1.22;3.31)	1.20 (0.62; 2.34)
	3.79 (0.34; 42.32)	0.54 (0.15; 1.92)	1.90 (1.19; 3.02)	Palanosetron	0.96 (0.30; 3.07)
	2.66 (0.23; 30.49)	0.38 (0.10; 1.44)	1.33 (0.74; 2.39)	0.70 (0.36; 1.38)	Ramosetron
>24 h vomiting	Azasetron	—	3.00 (0.13; 71.89)	—	—
	8.52 (0.07; 976.89)	Granisetron	—	1.00 (0.02; 49.04)	1.00 (0.02; 49.04)
	3.00 (0.13; 71.89)	0.35 (0.01; 11.90)	Ondansetron	2.83 (0.42; 19.02)	2.34 (0.82; 6.69)
	11.28 (0.33; 384.71)	1.32 (0.04; 42.36)	3.76 (0.81; 17.51)	Palanosetron	0.37 (0.03; 3.96)
	6.44 (0.23; 179.48)	0.76 (0.02; 24.16)	2.15 (0.79; 5.79)	0.57 (0.11; 2.84)	Ramosetron
Overall vomiting	Azasetron	—	0.62 (0.16; 2.42)	—	—
	1.73 (0.30; 9.87)	Granisetron	0.33 (0.11; 1.00)	2.00 (0.16; 24.33)	—
	0.62 (0.16; 2.42)	0.36 (0.12; 1.09)	Ondansetron	1.71 (0.84; 3.47)	0.89 (0.38; 2.08)
	1.11 (0.25; 4.96)	0.65 (0.18; 2.25)	1.78 (0.94; 3.36)	Palanosetron	0.41 (0.13; 1.32)
	0.52 (0.11; 2.41)	0.30 (0.08; 1.12)	0.83 (0.41; .71)	0.47 (0.21; 1.04)	Ramosetron
	0.81 (0.17; 3.94)	0.47 (0.12; 1.85)	1.30 (0.58; 2.94)	0.73 (0.26; 2.05)	1.56 (0.53; 4.61)

For “late vomiting,” 13 RCTs (Data S5) revealed that granisetron (RR 0.28, 95% CI 0.08–0.96) and palanosetron (RR 1.90, 95% CI 1.19–3.02) were significantly more effective than ondansetron (Table 4). Based on the P‐score, granisetron was the best choice, followed by palanosetron, ramosetron, ondansetron, and azasetron (Table 3).

Eight RCTs reported “>24 h vomiting” statistics (Data S5), which did not show statistical significance among all 5‐HT_3_ antagonists (Table 4). However, palanosetron seemed to be the most effective treatment, followed by granisetron, ramosetron, ondansetron, and azasetron (Table 3).

For “overall vomiting,” 13 RCTs (Data S5) showed that treatment efficacies were nearly equal among all 5‐HT_3_ antagonists (Table 4). Granisetron appeared to be the most suitable choice, followed by palanosetron, azasetron, tropisetron, ondansetron, and ramosetron (Table 3).

### 3.3 PONV

In the analysis of “acute PONV,” In the 11 RCTs (Data S5), no statistically significant differences were observed among all 5‐HT_3_ antagonists (Table 5). However, palonosetron seemed to be the most effective treatment for “acute PONV,” followed by granisetron, ramosetron, ondansetron, and dolasetron (Table 3).

**TABLE 5:** Network calculation of PONV.

Acute PONV	Dolasetron	1.48 (0.70; 3.11)	1.29 (0.63; 2.64)	—	—
	1.50 (0.76; 2.97)	Granisetron	0.85 (0.48; 1.48)	1.00 (0.02; 50.24)	—
	1.28 (0.66; 2.49)	0.85 (0.49; 1.49)	Ondansetron	1.44 (0.92; 2.26)	0.89 (0.54; 1.48)
	1.61 (0.75; 3.47)	1.07 (0.55; 2.11)	1.26 (0.86; 1.85)	Palanosetron	0.99 (0.59; 1.66)
	1.34 (0.61; 2.92)	0.89 (0.45; 1.78)	1.05 (0.70; 1.57)	0.83 (0.55; 1.26)	Ramosetron
Late PONV	Dolasetron	1.06 (0.53; 2.13)	0.94 (0.49; 1.84)	—	—
	1.11 (0.57; 2.15)	Granisetron	0.78 (0.44; 1.37)	2.50 (0.51; 12.35)	—
	0.91 (0.48; 1.73)	0.82 (0.47; 1.44)	Ondansetron	1.44 (1.07; 1.93)	0.85 (0.45; 1.62)
	1.23 (0.61; 2.45)	1.11 (0.60; 2.05)	1.34 (1.02; 1.77)	Palanosetron	0.97 (0.66; 1.43)
	1.06 (0.50; 2.24)	0.96 (0.49; 1.88)	1.16 (0.79; 1.72)	0.87 (0.62; 1.22)	Ramosetron
>24 h PONV	Ondansetron	1.28 (0.70; 2.34)	8.00 (0.98; 65.21)	—	—
	1.46 (0.82; 2.62)	Palanosetron	0.97 (0.57; 1.63)	—	—
	1.57 (0.74; 3.31)	1.07 (0.64; 1.78)	Ramosetron	—	—
Overall PONV	Azasetron	—	—	0.75 (0.46; 1.23)	—
	0.66 (0.33; 1.31)	Dolasetron	1.25 (0.74; 2.13)	1.33 (0.78; 2.27)	—
	0.95 (0.51; 1.77)	1.46 (0.89; 2.37)	Granisetron	0.77 (0.49; 1.20)	0.96 (0.49; 1.85)
	0.75 (0.46; 1.23)	1.14 (0.70; 1.86)	0.79 (0.54; 1.14)	Ondansetron	1.35 (1.04; 1.77)
	0.94 (0.55; 1.62)	1.43 (0.85; 2.43)	0.99 (0.66; 1.48)	1.25 (1.00; 1.58)	Palanosetron
	0.86 (0.50; 1.50)	1.31 (0.77; 2.25)	0.90 (0.59; 1.38)	1.15 (0.90; 1.47)	0.92 (0.71; 1.19)

Abbreviation: PONV, postoperative nausea and vomiting.

Palonosetron was superior to ondansetron (RR 1.34, 95% CI 1.02–1.77) (Table 5) in the treatment of “late PONV,” according to 11 RCTs (Data S5). P‐scores indicated that palonosetron was the most effective treatment, followed by granisetron, ramosetron, dolasetron, and ondansetron (Table 3).

Five RCTs (Data S5) reporting “>24 h PONV” did not show significant differences between ramosetron, palanosetron, and ondansetron, which were analyzed (Table 5). However, ramosetron emerged as the preferred choice, followed by palanosetron and ondansetron (Table 3).

For “overall PONV,” 14 RCTs (Data S5) included in the study showed a significant difference between palanosetron and ondansetron (RR 1.25, 95% CI 1.00–1.58) (Table 5). According to the P‐score, azasetron appears to be the most effective treatment for “overall PONV,” followed by palanosetron, granisetron, ramosetron, and ondansetron, as well as dolasetron (Table 3).

### 3.4 Rescue medicine

In the analysis of “acute rescue medicine,” No significant difference was found among the 5‐HT_3_ antagonists in the analysis of 10 RCTs (Data S5)  (Table 6). Granisetron seems to be the least likely to require rescue medicine, followed by ramosetron, azasetron, palanosetron, and ondansetron (Table 3).

**TABLE 6:** Network calculation of rescue medicine.

Acute rescue medicine	Azasetron	—	1.00 (0.31; 3.24)	—	—
	1.90 (0.22; 16.58)	Granisetron	0.33 (0.01; 7.87)	0.60 (0.08; 4.51)	1.00 (0.07; 15.36)
	1.00 (0.31; 3.24)	0.53 (0.09; 3.25)	Ondansetron	1.03 (0.65; 1.63)	1.22 (0.77; 1.93)
	1.01 (0.29; 3.55)	0.53 (0.09; 3.26)	1.01 (0.65; 1.58)	Palanosetron	1.22 (0.54; 2.79)
	1.23 (0.35; 4.31)	0.65 (0.10; 4.07)	1.23 (0.79; 1.92)	1.22 (0.69; 2.15)	Ramosetron
Late rescue medicine	Azasetron	—	1.00 (0.31; 3.24)	—	—
	3.54 (0.61; 20.69)	Granisetron	0.25 (0.06; 1.08)	1.06 (0.16; 6.98)	1.00 (0.02; 49.04)
	1.00 (0.31; 3.24)	0.28 (0.08; 1.05)	Ondansetron	1.81 (1.24; 2.66)	1.12 (0.61; 2.07)
	1.77 (0.52; 6.10)	0.50 (0.13; 1.93)	1.77 (1.21; 2.59)	Palanosetron	3.00 (0.13; 71.22)
	1.19 (0.32; 4.44)	0.33 (0.08; 1.41)	1.19 (0.65; 2.16)	0.67 (0.33; 1.35)	Ramosetron
>24h rescue medicine	Azasetron	—	3.00 (0.13; 71.89)	—	—
	10.23 (0.09;1151.07)	Granisetron	—	1.00 (0.02; 49.04)	1.00 (0.02; 49.04)
	3.00 (0.13; 71.89)	0.29 (0.01; 9.68)	Ondansetron	2.40 (1.03; 5.57)	5.34 (0.95; 30.12)
	7.41 (0.28; 197.52)	0.73 (0.02; 23.53)	2.47 (1.08; 5.65)	Palanosetron	1.00 (0.02; 49.04)
	14.10 (0.40; 491.45)	1.38 (0.04; 44.76)	4.70 (0.96; 22.98)	1.90 (0.34; 10.68)	Ramosetron
Overall rescue medicine	Granisetron	0.28 (0.08; 1.05)	2.00 (0.18; 22.46)	—	—
	0.33 (0.09; 1.20)	Ondansetron	1.13 (0.64; 1.97)	1.83 (0.87; 3.88)	1.26 (0.54; 2.93)
	0.42 (0.10; 1.67)	1.28 (0.77; 2.14)	Palanosetron	0.70 (0.24; 2.02)	—
	0.47 (0.11;2.00)	1.44 (0.77; 2.73)	1.13 (0.56; 2.27)	Ramosetron	—
	0.41 (0.09; 1.94)	1.26 (0.54; 2.93)	0.98 (0.37; 2.64)	0.87 (0.30; 2.51)	Tropisetron

For “late rescue medicine,” nine RCTs (Data S5) demonstrated that the effectiveness of palanosetron was significantly higher than that of ondansetron (RR 1.77, 95% CI 1.21–2.59) (Table 6). Granisetron appears to be the most effective treatment for delayed rescue, followed by palanosetron, ramosetron, azasetron, and ondansetron (Table 3).

Among six RCTs (Data S5) that evaluated “>24 h rescue medicine,” palanosetron performed significantly better than ondansetron (RR 2.47, 95% CI 1.08–5.65) (Table 6). Ramosetron emerged as the choice with the most potential to avoid rescue medicine, followed by granisetron, palanosetron, ondansetron, and azasetron (Table 3).

The analysis of “overall rescue medicine” (Data S5) included 10 RCTs, but none of them showed statistical significance (Table 6). It appeared that granisetron would require the least amount of rescue medication, followed by ramosetron, palanosetron, tropisetron, and ondansetron (Table 3).

### 3.5 Adverse reaction

In 17 RCTs (Data S5) used in the analysis of “adverse reaction,” a statistically significant difference was not observed among all 5‐HT_3_ antagonists (Table 7). The P‐score of total adverse reactions indicated that tropisetron seemed to have fewer adverse reactions, followed by palanosetron, azasetron, ramosetron, ondansetron, and granisetron (Table 3).

**TABLE 7:** Network calculation of adverse reaction.

Azasetron	—	0.92 (0.40; 2.09)	—	—	—
0.76 (0.25; 2.30)	Granisetron	1.08 (0.51; 2.30)	5.00 (0.60; 41.91)	—	—
0.92 (0.40; 2.09)	1.20 (0.58; 2.51)	Ondansetron	1.04 (0.80; 1.35)	1.06 (0.73; 1.54)	1.14 (0.46; 2.81)
0.98 (0.41; 2.31)	1.28 (0.59; 2.77)	1.07 (0.83; 1.36)	Palanosetron	0.96 (0.61; 1.54)	—
0.94 (0.39; 2.28)	1.23 (0.55; 2.74)	1.02 (0.74; 1.42)	0.96 (0.68; 1.36)	Ramosetron	—
1.05 (0.31; 3.55)	1.37 (0.43; 4.39)	1.14 (0.46; 2.81)	1.07 (0.42; 2.73)	1.12 (0.43; 2.91)	Tropisetron

The primary adverse effects of 5‐HT_3_ antagonists in our study included headache, dizziness, constipation, drowsiness and so on. Those adverse reactions were generally well tolerated (Data S12).

### 3.6 Transitivity and sensitivity analyses

Transitivity was evaluated across publication years, mean age, weight, duration of surgery, and anesthesia in this study, with no significant inconsistency found. (Data S6).

Six studies reported open or mostly open surgery, and the pooled estimates were not significantly impacted by the exclusion of data from these studies (Data S7).

In six studies, non‐opioid analgesia was used in postoperative analgesia, and removing these data did not significantly alter the pooled estimates. (Data S8).

We excluded one study that used spinal anesthesia and did not significantly change the pooled estimates (Data S9).

Two studies maintained anesthesia with propofol, and exclusion of data from these studies did not significantly influence the pooled estimates compared with the overall analysis (Data S10).

## 4 DISCUSSION

In the present study, after gynecological surgery, palonosetron demonstrates significantly superior efficacy to ondansetron across multiple endpoints, including  “acute nausea,” “overall nausea,” “acute vomiting,” “late vomiting,” “late PONV,” “overall PONV,” “late rescue medicine,” and “>24 h rescue medicine.” Many previous studies indicated that palonosetron generally demonstrated significantly superior efficacy in the prevention of PONV compared with ondansetron.^45^, ^46^, ^47^, ^48^, ^49^, ^50^ However, a meta‐analysis showed that there was no significant difference between palonosetron and ondansetron in treating overall PONV within the initial 24 h, while palonosetron showed superior antiemetic effects specifically for vomiting control.^20^ Another study suggested that the rates of nausea and vomiting in patients administered with palonosetron were not significantly different from those who received ondansetron during the first 24 h following surgery. Furthermore, no significant disparities were detected in the severity of nausea or the necessity for rescue antiemetic medication between the two groups.^51^ Our research findings agreed with the majority of previous studies: the efficacy of palonosetron significantly surpassed that of ondansetron. If a choice must be made between palonosetron and ondansetron, palonosetron is the preferred option.

Our study found that there were no significant differences between palonosetron and granisetron in PONV prophylaxis for all indicators, including nausea, vomiting, PONV, rescue medicine, and adverse effects. Moreover, in P‐score rating system, both palonosetron and granisetron acheived similarly high scores, with no statistically significant difference between their rankings. But a meta‐analysis showed that palonosetron was significantly more effective in preventing acute vomiting and PONV in both acute and late phases compared to granisetron.^19^

When we specifically considered the acute postoperative period, we also found that palonosetron was more effective than ondansetron and there were no significant differences between palonosetron and granisetron in our study.

In this study, we found that palonosetron, in comparison to ramosetron, only exhibited significantly enhanced efficacy in “acute nausea.” A meta‐analysis showed that postoperative vomiting was considerably lower in the palonosetron group than in the ramosetron group. But no significant differences were demonstrated in the incidence of nausea, PONV, use of antiemetics, and adverse effects.^52^ Another meta‐analysis pointed out that palonosetron significantly surpassed ramosetron in late‐phase vomiting and PONV but not in early‐phase vomiting and PONV.^19^

In first‐generation 5‐HT_3_ receptor antagonists, we found that aside from granisetron showing significantly superior efficacy to ondansetron in “late vomiting” and ramosetron demonstrating better performance in “>24 h nausea” compared with ondansetron, the remainder of the first‐generation 5‐HT_3_ receptor antagonists exhibited no significant differences in various indicators. Some articles showed that granisetron exhibited significantly superior efficacy over ondansetron.^53^, ^54^, ^55^, ^56^ However, a meta‐analysis pointed out that although ganisetron was better than ondansetron for PONV, these differences were not statistically significant.^57^ One study showed no significant difference in the reduction of PONV incidence between ramosetron and ondansetron,^58^ but other studies showed that ramosetron may be significantly more effective than ondansetron for the prevention of PONV in laparoscopic surgery under general anesthesia.^59^, ^60^, ^61^

There were a limited number of studies of tropisetron, azasetron, and dolasetron in our research, and no significant differences were observed among them or compared with other first‐generation 5‐HT_3_ receptor antagonists across various indicators. Their P‐score ratings were generally higher than those of ondansetron.

Based on these conclusions, we propose the following treatment plan for the clinical application of 5‐HT_3_ receptor antagonists to prevent PONV in gynecological surgery:

1. The efficacy of granisetron was comparable to that of palonosetron in preventing PONV in gynecological surgery. From a cost‐effectiveness perspective, granisetron may be an optimal alternative to palonosetron for antiemetic use.
2. Palonosetron's effect was significantly superior to ondansetron. If these are the only two drugs available for selection, palonosetron is recommended for the prevention of PONV in gynecological surgery.

However, there were still some limitations in this study. The number of articles available for certain medicines, such as azasetron and tropisetron, was limited, which will potentially affect the outcome of the analysis. More relevant research in the future is needed.

## 5 CONCLUSIONS

The data in our study suggest that in preventing PONV in gynecological surgery, palonosetron's effect was significantly superior to ondansetron, and the efficacy of granisetron was comparable to that of palonosetron. Among the first‐generation 5‐HT_3_ receptor antagonists, granisetron generally exhibited better effects than other 5‐HT_3_ receptor antagonists.

## AUTHOR CONTRIBUTIONS

HX designed the study and supervised the overall project. LR and SY participated in study identification, selection, and quality assessment. JX, HL, and HX did data abstraction. HD, LL, and XC participated in statistical analysis. HX wrote the manuscript.

## FUNDING INFORMATION

2021 Clinical Research Funds of Shandong Medical Association‐Qilu Specialized Funding.

## CONFLICT OF INTEREST STATEMENT

The authors declare have no conflictS of interest.

## Supporting information


Data S1.

Data S2.

Data S3.

Data S4.

Data S5.

Data S6.

Data S7.

Data S8.

Data S9.

Data S10.

Data S11.

Data S12.

## Data Availability

Data sharing is not applicable to this article as no datasets were generated or analyzed during the current study.
