# Supplementary material for: 5‐HT3 receptor antagonists for preventing postoperative nausea and vomiting after gynecological surgery: A systematic review and network meta‐analysis
Source: Int J Gynaecol Obstet. 2025 May 9;171(1):177–89. doi: 10.1002/ijgo.70197 (PMC12447676; doi:10.1002/ijgo.70197)
Supplement: Supplementary file 1 — Data S1. [file IJGO-171-177-s005.docx]

**Data S1 The search strategies of English databases**

| database | search strategy |
| --- | --- |
| PubMed | **((("Gynecologic Surgical Procedures"[Mesh]) OR (((((((((((((((((((Procedures, Gynecologic Surgical[Title/Abstract]) OR (Surgical Procedure, Gynecologic[Title/Abstract])) OR (Surgery, Gynecological[Title/Abstract])) OR (Gynecological Surgeries[Title/Abstract])) OR (Gynecological Surgery[Title/Abstract])) OR (Surgeries, Gynecological[Title/Abstract])) OR (Gynecologic Surgical Procedure[Title/Abstract])) OR (Surgical Procedures, Gynecologic[Title/Abstract])) OR (Gynecological Surgical Procedure[Title/Abstract])) OR (Gynecological Surgical Procedures[Title/Abstract])) OR (Procedure, Gynecological Surgical[Title/Abstract])) OR (Procedures, Gynecological Surgical[Title/Abstract])) OR (Surgical Procedure, Gynecological[Title/Abstract])) OR (Surgical Procedures, Gynecological[Title/Abstract])) OR (Procedure, Gynecologic Surgical[Title/Abstract])) OR (Gynecologic Surgery[Title/Abstract])) OR (Gynecologic Surgeries[Title/Abstract])) OR (Surgeries, Gynecologic[Title/Abstract])) OR (Surgery, Gynecologic[Title/Abstract]))) AND (("Postoperative Nausea and Vomiting"[Mesh]) OR ((((((((((PONV[Title/Abstract]) OR (Nausea[Title/Abstract] AND Vomiting, Postoperative[Title/Abstract])) OR (Vomiting, Postoperative[Title/Abstract])) OR (Postoperative Emesis[Title/Abstract])) OR (Postoperative Vomiting[Title/Abstract])) OR (Emesis, Postoperative[Title/Abstract])) OR (Emeses, Postoperative[Title/Abstract])) OR (Postoperative Emeses[Title/Abstract])) OR (Postoperative Nausea[Title/Abstract])) OR (Nausea, Postoperative[Title/Abstract])))) AND ((((((((((((((("Ondansetron"[Mesh]) OR (((((((((((((Ondansetron, (+,-)-Isomer[Title/Abstract]) OR (Zofran ODT[Title/Abstract])) OR (ODT, Zofran[Title/Abstract])) OR (Ondansetron, (R)-Isomer[Title/Abstract])) OR (Ondansetron, (S)-Isomer[Title/Abstract])) OR (Zofran[Title/Abstract])) OR (Ondansetron Hydrochloride[Title/Abstract])) OR (Hydrochloride, Ondansetron[Title/Abstract])) OR (Ondansetron Monohydrochloride[Title/Abstract])) OR (Monohydrochloride, Ondansetron[Title/Abstract])) OR (Ondansetron Monohydrochloride Dihydrate[Title/Abstract])) OR (Dihydrate, Ondansetron Monohydrochloride[Title/Abstract])) OR (Monohydrochloride Dihydrate, Ondansetron[Title/Abstract]))) OR ("Granisetron"[Mesh])) OR (((((Kytril[Title/Abstract]) OR (Granisetron Hydrochloride[Title/Abstract])) OR (Hydrochloride, Granisetron[Title/Abstract])) OR (Granisetron Monohydrochloride[Title/Abstract])) OR (Monohydrochloride, Granisetron[Title/Abstract]))) OR ("dolasetron" [Supplementary Concept])) OR ((((dolasetron mesylate[Title/Abstract]) OR (dolasetron mesylate monohydrate[Title/Abstract])) OR (dolasetron mesilate monohydrate[Title/Abstract])) OR (Anzemet[Title/Abstract]))) OR ("Tropisetron"[Mesh])) OR (((Navoban[Title/Abstract]) OR (Indole 3 carboxylic Acid Tropine Ester[Title/Abstract])) OR (Tropisetron Hydrochloride[Title/Abstract]))) OR ("ramosetron" [Supplementary Concept])) OR ((ramosetron hydrochloride[Title/Abstract]) OR (Nasea[Title/Abstract]))) OR ("azasetron" [Supplementary Concept])) OR (azasetron, (+-)-isomer[Title/Abstract])) OR (("azasetron"[Supplementary Concept] OR "azasetron"[All Fields]) AND "isomer"[Title/Abstract])) OR ("Palonosetron"[Mesh])) OR ((((((Palonosetron, (R-(R*,R*))-isomer[Title/Abstract]) OR (Palonosetron, (3R)-[Title/Abstract])) OR (Palonosetron, (R-(R*,S*))-isomer[Title/Abstract])) OR (Aloxi[Title/Abstract])) OR (Palonosetron, (S-(R*,S*))-isomer[Title/Abstract])) OR (Palonosetron Hydrochloride[Title/Abstract])))** Filters: **from 1000/1/1 - 2023/9/20** |
| Embase | #5 AND #10 AND #39  [472](https://www.embase.com/)  **#39**  #11 OR #12 OR #13 OR #14 OR #15 OR #16 OR #17 OR #18 OR #19 OR #20 OR #21 OR #22 OR #23 OR #24 OR #25 OR #26 OR #27 OR #28 OR #29 OR #30 OR #31 OR #32 OR #33 OR #34 OR #35 OR #36 OR #37 OR #38  [30,459](https://www.embase.com/)  **#38**  (**palonosetron,**:ab,ti AND **r-**:ab,ti AND **r*,r***:ab,ti AND **-isomer**:ab,ti OR (**palonosetron,**:ab,ti AND **3r**:ab,ti AND **-**:ab,ti) OR (**palonosetron,**:ab,ti AND **r-**:ab,ti AND **r*,s***:ab,ti AND **-isomer**:ab,ti) OR **aloxi**:ab,ti OR (**palonosetron,**:ab,ti AND **s-**:ab,ti AND **r*,s***:ab,ti AND **-isomer**:ab,ti) OR **'palonosetron hydrochloride'**:ab,ti) AND [01-01-2023]/sd NOT [21-09-2023]/sd  [1](https://www.embase.com/)  **#37**  (**palonosetron,**:ab,ti AND **r-**:ab,ti AND **r*,r***:ab,ti AND **-isomer**:ab,ti OR (**palonosetron,**:ab,ti AND **3r**:ab,ti AND **-**:ab,ti) OR (**palonosetron,**:ab,ti AND **r-**:ab,ti AND **r*,s***:ab,ti AND **-isomer**:ab,ti) OR **aloxi**:ab,ti OR (**palonosetron,**:ab,ti AND **s-**:ab,ti AND **r*,s***:ab,ti AND **-isomer**:ab,ti) OR **'palonosetron hydrochloride'**:ab,ti) AND [<1966-2022]/py  [103](https://www.embase.com/)  **#36**  (**'palonosetron'**/exp OR **'palonosetron'**) AND [01-01-2023]/sd NOT [21-09-2023]/sd  [150](https://www.embase.com/)  **#35**  (**'palonosetron'**/exp OR **'palonosetron'**) AND [<1966-2022]/py  [2,514](https://www.embase.com/)  **#34**  **azasetron,**:ab,ti AND **+-**:ab,ti AND **-isomer**:ab,ti AND [01-01-2023]/sd NOT [21-09-2023]/sd  [0](https://www.embase.com/)  **#33**  **azasetron,**:ab,ti AND **+-**:ab,ti AND **-isomer**:ab,ti AND [<1966-2022]/py  [0](https://www.embase.com/)  **#32**  (**'azasetron'**/exp OR **'azasetron'**) AND [01-01-2023]/sd NOT [21-09-2023]/sd  [15](https://www.embase.com/)  **#31**  (**'azasetron'**/exp OR **'azasetron'**) AND [<1966-2022]/py  [331](https://www.embase.com/)  **#30**  (**'ramosetron hydrochloride'**:ab,ti OR **nasea**:ab,ti) AND [01-01-2023]/sd NOT [21-09-2023]/sd  [0](https://www.embase.com/)  **#29**  (**'ramosetron hydrochloride'**:ab,ti OR **nasea**:ab,ti) AND [<1966-2022]/py  [43](https://www.embase.com/)  **#28**  (**'ramosetron'**/exp OR **'ramosetron'**) AND [01-01-2023]/sd NOT [21-09-2023]/sd  [66](https://www.embase.com/)  **#27**  (**'ramosetron'**/exp OR **'ramosetron'**) AND [<1966-2022]/py  [967](https://www.embase.com/)  **#26**  (**navoban**:ab,ti OR **'indole 3 carboxylic acid tropine ester'**:ab,ti OR **'tropisetron hydrochloride'**:ab,ti) AND [01-01-2023]/sd NOT [21-09-2023]/sd  [1](https://www.embase.com/)  **#25**  (**navoban**:ab,ti OR **'indole 3 carboxylic acid tropine ester'**:ab,ti OR **'tropisetron hydrochloride'**:ab,ti) AND [<1966-2022]/py  [90](https://www.embase.com/)  **#24**  (**'tropisetron'**/exp OR **'tropisetron'**) AND [01-01-2023]/sd NOT [21-09-2023]/sd  [122](https://www.embase.com/)  **#23**  (**'tropisetron'**/exp OR **'tropisetron'**) AND [<1966-2022]/py  [3,816](https://www.embase.com/)  **#22**  (**'dolasetron mesylate'**:ab,ti OR **'dolasetron mesylate monohydrate'**:ab,ti OR **'dolasetron mesilate monohydrate'**:ab,ti OR **anzemet**:ab,ti) AND [01-01-2023]/sd NOT [21-09-2023]/sd  [0](https://www.embase.com/)  **#21**  (**'dolasetron mesylate'**:ab,ti OR **'dolasetron mesylate monohydrate'**:ab,ti OR **'dolasetron mesilate monohydrate'**:ab,ti OR **anzemet**:ab,ti) AND [<1966-2022]/py  [64](https://www.embase.com/)  **#20**  (**'dolasetron mesilate'**/exp OR **'dolasetron mesilate'**) AND [01-01-2023]/sd NOT [21-09-2023]/sd  [23](https://www.embase.com/)  **#19**  (**'dolasetron mesilate'**/exp OR **'dolasetron mesilate'**) AND [<1966-2022]/py  [1,473](https://www.embase.com/)  **#18**  (**kytril**:ab,ti OR **'granisetron hydrochloride'**:ab,ti OR **'hydrochloride, granisetron'**:ab,ti OR **'granisetron monohydrochloride'**:ab,ti OR **'monohydrochloride, granisetron'**:ab,ti) AND [randomized controlled trial]/lim AND [01-01-2023]/sd NOT [21-09-2023]/sd  [0](https://www.embase.com/)  **#17**  (**kytril**:ab,ti OR **'granisetron hydrochloride'**:ab,ti OR **'hydrochloride, granisetron'**:ab,ti OR **'granisetron monohydrochloride'**:ab,ti OR **'monohydrochloride, granisetron'**:ab,ti) AND [randomized controlled trial]/lim AND [<1966-2022]/py  [28](https://www.embase.com/)  **#16**  (**'granisetron'**/exp OR **'granisetron'**) AND [01-01-2023]/sd NOT [21-09-2023]/sd  [153](https://www.embase.com/)  **#15**  (**'granisetron'**/exp OR **'granisetron'**) AND [<1966-2022]/py  [6,046](https://www.embase.com/)  **#14**  (**ondansetron,**:ab,ti AND **+,-**:ab,ti AND **-isomer**:ab,ti OR **'zofran odt'**:ab,ti OR **'odt, zofran'**:ab,ti OR (**ondansetron,**:ab,ti AND **r**:ab,ti AND **-isomer**:ab,ti) OR (**ondansetron,**:ab,ti AND **s**:ab,ti AND **-isomer**:ab,ti) OR **zofran**:ab,ti OR **'ondansetron hydrochloride'**:ab,ti OR **'hydrochloride, ondansetron'**:ab,ti OR **'ondansetron monohydrochloride'**:ab,ti OR **'monohydrochloride, ondansetron'**:ab,ti OR **'ondansetron monohydrochloride dihydrate'**:ab,ti OR **'dihydrate, ondansetron monohydrochloride'**:ab,ti OR **'monohydrochloride dihydrate, ondansetron'**:ab,ti) AND [01-01-2023]/sd NOT [21-09-2023]/sd  [12](https://www.embase.com/)  **#13**  (**ondansetron,**:ab,ti AND **+,-**:ab,ti AND **-isomer**:ab,ti OR **'zofran odt'**:ab,ti OR **'odt, zofran'**:ab,ti OR (**ondansetron,**:ab,ti AND **r**:ab,ti AND **-isomer**:ab,ti) OR (**ondansetron,**:ab,ti AND **s**:ab,ti AND **-isomer**:ab,ti) OR **zofran**:ab,ti OR **'ondansetron hydrochloride'**:ab,ti OR **'hydrochloride, ondansetron'**:ab,ti OR **'ondansetron monohydrochloride'**:ab,ti OR **'monohydrochloride, ondansetron'**:ab,ti OR **'ondansetron monohydrochloride dihydrate'**:ab,ti OR **'dihydrate, ondansetron monohydrochloride'**:ab,ti OR **'monohydrochloride dihydrate, ondansetron'**:ab,ti) AND [<1966-2022]/py  [447](https://www.embase.com/)  **#12**  (**'ondansetron'**/exp OR **'ondansetron'**) AND [01-01-2023]/sd NOT [21-09-2023]/sd  [1,331](https://www.embase.com/)  **#11**  (**'ondansetron'**/exp OR **'ondansetron'**) AND [<1966-2022]/py  [21,732](https://www.embase.com/)  **#10**  #6 OR #7 OR #8 OR #9  [16,047](https://www.embase.com/)^*^  **#9**  (**ponv**:ab,ti OR (**nausea**:ab,ti AND **'vomiting, postoperative'**:ab,ti) OR **'vomiting, postoperative'**:ab,ti OR **'postoperative emesis'**:ab,ti OR **'postoperative vomiting'**:ab,ti OR **'emesis, postoperative'**:ab,ti OR **'emeses, postoperative'**:ab,ti OR **'postoperative emeses'**:ab,ti OR **'postoperative nausea'**:ab,ti OR **'nausea, postoperative'**:ab,ti) AND [01-01-2023]/sd NOT [21-09-2023]/sd  [682](https://www.embase.com/)  **#8**  (**ponv**:ab,ti OR (**nausea**:ab,ti AND **'vomiting, postoperative'**:ab,ti) OR **'vomiting, postoperative'**:ab,ti OR **'postoperative emesis'**:ab,ti OR **'postoperative vomiting'**:ab,ti OR **'emesis, postoperative'**:ab,ti OR **'emeses, postoperative'**:ab,ti OR **'postoperative emeses'**:ab,ti OR **'postoperative nausea'**:ab,ti OR **'nausea, postoperative'**:ab,ti) AND [<1966-2022]/py  [8,549](https://www.embase.com/)  **#7**  (**'postoperative nausea and vomiting'**/exp OR **'postoperative nausea and vomiting'**) AND [01-01-2023]/sd NOT [21-09-2023]/sd  [1,249](https://www.embase.com/)  **#6**  (**'postoperative nausea and vomiting'**/exp OR **'postoperative nausea and vomiting'**) AND [<1966-2022]/py  [14,185](https://www.embase.com/)  **#5**  #1 OR #2 OR #3 OR #4  [196,387](https://www.embase.com/)  **#4**  (**'gynecologic surgery'**/exp OR **'gynecologic surgery'**) AND [01-01-2023]/sd NOT [21-09-2023]/sd  [8,920](https://www.embase.com/)  **#3**  (**'gynecologic surgery'**/exp OR **'gynecologic surgery'**) AND [<1966-2022]/py  [189,118](https://www.embase.com/)  **#2**  (**'procedures, gynecologic surgical'**:ab,ti OR **'surgical procedure, gynecologic'**:ab,ti OR **'surgery, gynecological'**:ab,ti OR **'gynecological surgeries'**:ab,ti OR **'gynecological surgery'**:ab,ti OR **'surgeries, gynecological'**:ab,ti OR **'gynecologic surgical procedure'**:ab,ti OR **'surgical procedures, gynecologic'**:ab,ti OR **'gynecological surgical procedure'**:ab,ti OR **'gynecological surgical procedures'**:ab,ti OR **'procedure, gynecological surgical'**:ab,ti OR **'procedures, gynecological surgical'**:ab,ti OR **'surgical procedure, gynecological'**:ab,ti OR **'surgical procedures, gynecological'**:ab,ti OR **'procedure, gynecologic surgical'**:ab,ti OR **'gynecologic surgery'**:ab,ti OR **'gynecologic surgeries'**:ab,ti OR **'surgeries, gynecologic'**:ab,ti OR **'surgery, gynecologic'**:ab,ti) AND [01-01-2023]/sd NOT [21-09-2023]/sd  [371](https://www.embase.com/)   - [Edit](https://www.embase.com/) - [Email alert](https://www.embase.com/) - [RSS feed](https://www.embase.com/)   **#1**  (**'procedures, gynecologic surgical'**:ab,ti OR **'surgical procedure, gynecologic'**:ab,ti OR **'surgery, gynecological'**:ab,ti OR **'gynecological surgeries'**:ab,ti OR **'gynecological surgery'**:ab,ti OR **'surgeries, gynecological'**:ab,ti OR **'gynecologic surgical procedure'**:ab,ti OR **'surgical procedures, gynecologic'**:ab,ti OR **'gynecological surgical procedure'**:ab,ti OR **'gynecological surgical procedures'**:ab,ti OR **'procedure, gynecological surgical'**:ab,ti OR **'procedures, gynecological surgical'**:ab,ti OR **'surgical procedure, gynecological'**:ab,ti OR **'surgical procedures, gynecological'**:ab,ti OR **'procedure, gynecologic surgical'**:ab,ti OR **'gynecologic surgery'**:ab,ti OR **'gynecologic surgeries'**:ab,ti OR **'surgeries, gynecologic'**:ab,ti OR **'su** |
| Cochrane | #1 MeSH descriptor: [Gynecologic Surgical Procedures] explode all trees  #2 (Procedures, Gynecologic Surgical):ti,ab,kw OR (Surgical Procedure, Gynecologic):ti,ab,kw OR (Surgery, Gynecological):ti,ab,kw OR (Gynecological Surgeries):ti,ab,kw OR (Gynecological Surgery):ti,ab,kw (Word variations have been searched)  #3 (Surgeries, Gynecological):ti,ab,kw OR (Gynecologic Surgical Procedure):ti,ab,kw OR (Surgical Procedures, Gynecologic):ti,ab,kw OR (Gynecological Surgical Procedure):ti,ab,kw OR (Gynecological Surgical Procedures):ti,ab,kw (Word variations have been searched)  #4 (Procedure, Gynecological Surgical):ti,ab,kw OR (Procedures, Gynecological Surgical):ti,ab,kw OR (Surgical Procedure, Gynecological):ti,ab,kw OR (Surgical Procedures, Gynecological):ti,ab,kw OR (Procedure, Gynecologic Surgical):ti,ab,kw (Word variations have been searched)  #5 (Gynecologic Surgery):ti,ab,kw OR (Gynecologic Surgeries):ti,ab,kw OR (Surgeries, Gynecologic):ti,ab,kw OR (Surgery, Gynecologic):ti,ab,kw (Word variations have been searched)  #6 #1 or #2 or #3 or #4 or #5  #7 MeSH descriptor: [Postoperative Nausea and Vomiting] explode all trees  #8 (PONV):ti,ab,kw OR (Nausea and Vomiting, Postoperative):ti,ab,kw OR (Vomiting, Postoperative):ti,ab,kw OR (Postoperative Emesis):ti,ab,kw OR (Postoperative Vomiting):ti,ab,kw (Word variations have been searched)  #9 (Emesis, Postoperative):ti,ab,kw OR (Emeses, Postoperative):ti,ab,kw OR (Postoperative Emeses):ti,ab,kw OR (Postoperative Nausea):ti,ab,kw OR (Nausea, Postoperative):ti,ab,kw (Word variations have been searched)  #10 #7 or #8 or #9  #11 MeSH descriptor: [Ondansetron] explode all trees  #12 (Zofran):ti,ab,kw OR (Ondansetron Hydrochloride):ti,ab,kw OR (Hydrochloride, Ondansetron):ti,ab,kw OR (Ondansetron Monohydrochloride):ti,ab,kw OR (Monohydrochloride, Ondansetron):ti,ab,kw (Word variations have been searched)  #13 (Ondansetron, Isomer):ti,ab,kw OR (Zofran ODT):ti,ab,kw OR (ODT, Zofran):ti,ab,kw OR (Ondansetron, (R)Isomer):ti,ab,kw OR (Ondansetron, (S)Isomer):ti,ab,kw (Word variations have been searched)  #14 (Ondansetron Monohydrochloride Dihydrate):ti,ab,kw OR (Dihydrate, Ondansetron Monohydrochloride):ti,ab,kw OR (Monohydrochloride Dihydrate, Ondansetron):ti,ab,kw (Word variations have been searched)  #15 MeSH descriptor: [Granisetron] explode all trees  #16 (Kytril):ti,ab,kw OR (Granisetron Hydrochloride):ti,ab,kw OR (Hydrochloride, Granisetron):ti,ab,kw OR (Granisetron Monohydrochloride):ti,ab,kw OR (Monohydrochloride, Granisetron):ti,ab,kw (Word variations have been searched)  #17 MeSH descriptor: [] explode all trees  #18 (dolasetron mesylate):ti,ab,kw OR (dolasetron mesylate monohydrate):ti,ab,kw OR (dolasetron mesilate monohydrate):ti,ab,kw OR (Anzemet):ti,ab,kw (Word variations have been searched)  #19 MeSH descriptor: [Tropisetron] explode all trees  #20 (Navoban):ti,ab,kw OR (Indole 3 carboxylic Acid Tropine Ester):ti,ab,kw OR (Tropisetron Hydrochloride):ti,ab,kw (Word variations have been searched)  #21 MeSH descriptor: [] explode all trees  #22 (ramosetron hydrochloride):ti,ab,kw OR (Nasea):ti,ab,kw (Word variations have been searched)  #23 MeSH descriptor: [] explode all trees  #24 (azasetron, isomer):ti,ab,kw (Word variations have been searched)  #25 MeSH descriptor: [Palonosetron] explode all trees  #26 (Palonosetron, (R-(R*,R*))isomer):ti,ab,kw OR (Palonosetron, (3R)):ti,ab,kw OR (Palonosetron, (R-(R*,S*))isomer):ti,ab,kw OR (Aloxi):ti,ab,kw OR (Palonosetron, (S-(R*,S*))isomer):ti,ab,kw (Word variations have been searched)  #27 (Palonosetron Hydrochloride):ti,ab,kw (Word variations have been searched)  #28 #11 or #12 or #13 or #14 or #15 or #16 or #17 or #18 or #19 or #20 or #21 or #22 or #23 or #24 or #25 or #26 or #27  #29 #6 and #10 and #28 with Cochrane Library publication date to Sep 2023 |
| Web of Science | 1: (((((((((((((((((((TS=(Gynecologic Surgical Procedures)) OR TS=(Procedures, Gynecologic Surgical)) OR TS=(Surgical Procedure, Gynecologic)) OR TS=(Surgery, Gynecological)) OR TS=(Gynecological Surgeries)) OR TS=(Gynecological Surgery)) OR TS=(Surgeries, Gynecological)) OR TS=(Gynecologic Surgical Procedure)) OR TS=(Surgical Procedures, Gynecologic)) OR TS=(Gynecological Surgical Procedure)) OR TS=(Gynecological Surgical Procedures)) OR TS=(Procedure, Gynecological Surgical)) OR TS=(Procedures, Gynecological Surgical)) OR TS=(Surgical Procedure, Gynecological)) OR TS=(Surgical Procedures, Gynecological)) OR TS=(Procedure, Gynecologic Surgical)) OR TS=(Gynecologic Surgery)) OR TS=(Gynecologic Surgeries)) OR TS=(Surgeries, Gynecologic)) OR TS=(Surgery, Gynecologic)  2: ((((((((((TS=(Postoperative Nausea and Vomiting)) OR TS=(PONV)) OR TS=(Nausea and Vomiting, Postoperative)) OR TS=(Vomiting, Postoperative)) OR TS=(Postoperative Emesis)) OR TS=(Postoperative Vomiting)) OR TS=(Emesis, Postoperative)) OR TS=(Emeses, Postoperative)) OR TS=(Postoperative Emeses)) OR TS=(Postoperative Nausea)) OR TS=(Nausea, Postoperative)  3: (((((((((((((TS=(Ondansetron)) OR TS=(Ondansetron, (+,-)-Isomer )) OR TS=(Zofran ODT)) OR TS=(ODT, Zofran)) OR TS=(Ondansetron, (R)-Isomer)) OR TS=(Ondansetron, (S)-Isomer)) OR TS=(Zofran)) OR TS=(Ondansetron Hydrochloride)) OR TS=(Hydrochloride, Ondansetron)) OR TS=(Ondansetron Monohydrochloride)) OR TS=(Monohydrochloride, Ondansetron)) OR TS=(Ondansetron Monohydrochloride Dihydrate)) OR TS=(Dihydrate, Ondansetron Monohydrochloride)) OR TS=(Monohydrochloride Dihydrate, Ondansetron)  4: (((((TS=(Granisetron)) OR TS=(Kytril)) OR TS=(Granisetron Hydrochloride)) OR TS=(Hydrochloride, Granisetron)) OR TS=(Granisetron Monohydrochloride)) OR TS=(Monohydrochloride, Granisetron)  5: ((((TS=(dolasetron)) OR TS=(dolasetron mesylate)) OR TS=(dolasetron mesylate monohydrate)) OR TS=(dolasetron mesilate monohydrate)) OR TS=(Anzemet)  6: (((TS=(Tropisetron)) OR TS=(Navoban)) OR TS=(Indole 3 carboxylic Acid Tropine Ester)) OR TS=(Tropisetron Hydrochloride)  7: ((TS=(ramosetron)) OR TS=(ramosetron hydrochloride)) OR TS=(Nasea)  8: (TS=(azasetron)) OR TS=(azasetron, (+-)-isomer )  9: (((TS=(Palonosetron)) OR TS=(Palonosetron, isomer)) OR TS=(Aloxi)) OR TS=(Palonosetron Hydrochloride)  10: #3 OR #4 OR #5 OR #6 OR #7 OR #8 OR #9  11: #10 AND #1 AND #2  12: #11 入库时间: 1900-01-01 to 2023-09-20 |
