# Supplementary material for: 5‐HT3 receptor antagonists for preventing postoperative nausea and vomiting after gynecological surgery: A systematic review and network meta‐analysis
Source: Int J Gynaecol Obstet. 2025 May 9;171(1):177–89. doi: 10.1002/ijgo.70197 (PMC12447676; doi:10.1002/ijgo.70197)
Supplement: Supplementary file 2 — Data S2. [file IJGO-171-177-s009.docx]

**Data S2 The heterogeneity within the network**

|  | tau^^2^ | tau | I^^2^ |
| --- | --- | --- | --- |
| Acute nausea | 0 | 0 | 0% [0.0%; 56.6%] |
| Late nausea | 0.0450 | 0.2121 | 20% [0.0%; 57.2%] |
| >24h nausea | 0.0031 | 0.0553 | 0.7% [0.0%; 74.8%] |
| Overall nausea | 0.0450 | 0.2122 | 34.5% [0.0%; 69.8%] |
| Acute vomiting | 0 | 0 | 0% [0.0%; 56.6%] |
| Late vomiting | 0 | 0 | 0% [0.0%; 58.3%] |
| >24h vomiting | 0 | 0 | 0% [0.0%; 74.6%] |
| Overall vomiting | 0.1920 | 0.4382 | 38.1% [0.0%; 70.5%] |
| Acute PONV | 0.0583 | 0.2415 | 29.6% [0.0%; 66.3%] |
| Late PONV | 0.0311 | 0.1764 | 24% [0.0%; 63.1%] |
| >24h PONV | 0.0543 | 0.2331 | 25.3% [0.0%; 71.3%] |
| Overall PONV | 0.0316 | 0.1777 | 32% [0.0%; 64.1%] |
| Acute rescue medicine | 0 | 0 | 0% [0.0%; 62.4%] |
| Late rescue medicine | 0 | 0 | 0% [0.0%; 67.6%] |
| >24h rescue medicine | 0 | 0 | 0% [0.0%; 84.7%] |
| Overall rescue medicine | 0.0892 | 0.2987 | 25.4% [0.0%; 66.1%] |
| Adverse reaction | 0.0435 | 0.2085 | 30.1% [0.0%; 62.4%] |
