# Supplementary material for: 5‐HT3 receptor antagonists for preventing postoperative nausea and vomiting after gynecological surgery: A systematic review and network meta‐analysis
Source: Int J Gynaecol Obstet. 2025 May 9;171(1):177–89. doi: 10.1002/ijgo.70197 (PMC12447676; doi:10.1002/ijgo.70197)
Supplement: Supplementary file 3 — Data S3. [file IJGO-171-177-s011.docx]

**Data S3 Inconsistency between studies**

|  | Q | df | p-value | tau.within | tau^2^.within |
| --- | --- | --- | --- | --- | --- |
| Acute nausea | 3.11 | 6 | 0.7947 | 0.1222 | 0.0149 |
| Late nausea | 8.46 | 7 | 0.2939 | 0.1194 | 0.0142 |
| >24h nausea | 3.16 | 2 | 0.2059 | 0 | 0 |
| Overall nausea | 4.66 | 3 | 0.1987 | 0.1622 | 0.0263 |
| Acute vomiting | 4.98 | 6 | 0.5459 | 0 | 0 |
| Late vomiting | 2.93 | 5 | 0.7115 | 0 | 0 |
| >24h vomiting | 0.66 | 2 | 0.7199 | 0 | 0 |
| Overall vomiting | 2.69 | 3 | 0.4415 | 0.4520 | 0.2043 |
| Acute PONV | 2.36 | 4 | 0.6698 | 0.3777 | 0.1427 |
| Late PONV | 3.56 | 4 | 0.4690 | 0.1932 | 0.0373 |
| >24h PONV | 2.84 | 1 | 0.0921 | 0 | 0 |
| Overall PONV | 10.35 | 8 | 0.2410 | 0.1408 | 0.0198 |
| Acute rescue medicine | 0.78 | 5 | 0.9783 | 0 | 0 |
| Late rescue medicine | 3.07 | 3 | 0.3814 | 0 | 0 |
| >24h rescue medicine | 0.13 | 1 | 0.7179 | 0 | 0 |
| Overall rescue medicine | 6.97 | 3 | 0.0730 | 0 | 0 |
| Adverse reaction | 2.64 | 5 | 0.7549 | 0.2792 | 0.0780 |

**Netsplit of“Acute nausea”**

| comparison | k | prop | nma | direct | indirect | RoR | z | p-value |
| --- | --- | --- | --- | --- | --- | --- | --- | --- |
| Azasetron vs Granisetron | 0 | 0 | 1.3994 | . | 1.3994 | . | . | . |
| Azasetron vs Ondansetron | 1 | 1 | 0.8182 | 0.8182 | . | . | . | . |
| Azasetron vs Palanosetron | 0 | 0 | 1.2113 | . | 1.2113 | . | . | . |
| Azasetron vs Ramosetron | 0 | 0 | 0.9321 | . | 0.9321 | . | . | . |
| Granisetron vs Ondansetron | 1 | 0.1 | 0.5847 | 0.2 | 0.6558 | 0.305 | -0.74 | 0.4602 |
| Granisetron vs Palanosetron | 2 | 0.77 | 0.8656 | 0.8445 | 0.9409 | 0.8976 | -0.1 | 0.9233 |
| Granisetron vs Ramosetron | 1 | 0.72 | 0.6661 | 0.8333 | 0.3785 | 2.2015 | 0.76 | 0.4498 |
| Palanosetron vs Ondansetron | 7 | 0.79 | 0.6755 | 0.6783 | 0.6646 | 1.0206 | 0.07 | 0.9452 |
| Ramosetron vs Ondansetron | 4 | 0.82 | 0.8777 | 0.8797 | 0.8688 | 1.0126 | 0.04 | 0.967 |
| Palanosetron vs Ramosetron | 3 | 0.81 | 0.7696 | 0.825 | 0.5757 | 1.433 | 1.12 | 0.2625 |

**Netsplit of“Late nausea”**

| comparison | k | prop | nma | direct | indirect | RoR | z | p-value |
| --- | --- | --- | --- | --- | --- | --- | --- | --- |
| Azasetron vs Granisetron | 0 | 0 | 1.2872 | . | 1.2872 | . | . | . |
| Azasetron vs Ondansetron | 1 | 1 | 0.875 | 0.875 | . | . | . | . |
| Azasetron vs Palanosetron | 0 | 0 | 1.1679 | . | 1.1679 | . | . | . |
| Azasetron vs Ramosetron | 0 | 0 | 0.9596 | . | 0.9596 | . | . | . |
| Granisetron vs Ondansetron | 2 | 0.5 | 0.6798 | 0.4132 | 1.1266 | 0.3668 | -1.13 | 0.2599 |
| Granisetron vs Palanosetron | 2 | 0.51 | 0.9073 | 1.7052 | 0.4652 | 3.6652 | 1.44 | 0.1513 |
| Granisetron vs Ramosetron | 1 | 0.43 | 0.7455 | 1 | 0.5973 | 1.6742 | 0.56 | 0.5775 |
| Palanosetron vs Ondansetron | 7 | 0.83 | 0.7492 | 0.6714 | 1.2694 | 0.5289 | -1.53 | 0.1266 |
| Ramosetron vs Ondansetron | 4 | 0.66 | 0.9119 | 1.175 | 0.5583 | 2.1048 | 1.79 | 0.0733 |
| Palanosetron vs Ramosetron | 3 | 0.5 | 0.8216 | 1.1362 | 0.5915 | 1.9208 | 1.54 | 0.1224 |

**Netsplit of“>24h nausea”**

| comparison |  | k | prop | nma | direct | indirect | RoR | z | p-value |
| --- | --- | --- | --- | --- | --- | --- | --- | --- | --- |
| Azasetron vs Granisetron |  | 0 | 0 | 3.9325 | . | 3.9325 | . | . | . |
| Azasetron vs Ondansetron |  | 1 | 1 | 1.5 | 1.5 | . | . | . | . |
| Azasetron vs Palanosetron |  | 0 | 0 | 2.1975 | . | 2.1975 | . | . | . |
| Azasetron vs Ramosetron |  | 0 | 0 | 3.1284 | . | 3.1284 | . | . | . |
| Granisetron vs Ondansetron |  | 0 | 0 | 0.3814 | . | 0.3814 | . | . | . |
| Granisetron vs Palanosetron |  | 1 | 0.9 | 0.5588 | 0.5 | 1.5224 | 0.3284 | -0.29 | 0.7698 |
| Granisetron vs Ramosetron |  | 1 | 0.69 | 0.7955 | 1 | 0.4827 | 2.0718 | 0.29 | 0.7698 |
| Palanosetron vs Ondansetron |  | 3 | 0.92 | 0.6826 | 0.773 | 0.1785 | 4.3312 | 1.7 | 0.0885 |
| Ramosetron vs Ondansetron |  | 2 | 0.24 | 0.4795 | 0.157 | 0.68 | 0.2309 | -1.7 | 0.0885 |
| Palanosetron vs Ramosetron |  | 2 | 0.85 | 1.4236 | 1.1368 | 4.9237 | 0.2309 | -1.7 | 0.0885 |

**Netsplit of“Overall nausea”**

| comparison | k | prop | nma | direct | indirect | RoR | z | p-value |
| --- | --- | --- | --- | --- | --- | --- | --- | --- |
| Azasetron vs Granisetron | 0 | 0 | 2.22 | . | 2.22 | . | . | . |
| Azasetron vs Ondansetron | 1 | 1 | 0.84 | 0.84 | . | . | . | . |
| Azasetron vs Palanosetron | 0 | 0 | 1.24 | . | 1.24 | . | . | . |
| Azasetron vs Ramosetron | 0 | 0 | 1.01 | . | 1.01 | . | . | . |
| Azasetron vs Tropisetron | 0 | 0 | 1.02 | . | 1.02 | . | . | . |
| Granisetron vs Ondansetron | 3 | 0.99 | 0.38 | 0.35 | 2537.25 | 0 | -1.84 | 0.0656 |
| Granisetron vs Palanosetron | 1 | 0.16 | 0.56 | 3 | 0.41 | 7.38 | 1.6 | 0.1092 |
| Granisetron vs Ramosetron | 0 | 0 | 0.46 | . | 0.46 | . | . | . |
| Granisetron vs Tropisetron | 0 | 0 | 0.46 | . | 0.46 | . | . | . |
| Palanosetron vs Ondansetron | 5 | 0.79 | 0.68 | 0.63 | 0.93 | 0.68 | -1.04 | 0.2987 |
| Ramosetron vs Ondansetron | 2 | 0.65 | 0.83 | 0.95 | 0.64 | 1.48 | 1.03 | 0.3012 |
| Tropisetron vs Ondansetron | 1 | 1 | 0.82 | 0.82 | . | . | . | . |
| Palanosetron vs Ramosetron | 1 | 0.56 | 0.82 | 0.97 | 0.66 | 1.48 | 1.03 | 0.3012 |
| Palanosetron vs Tropisetron | 0 | 0 | 0.83 | . | 0.83 | . | . | . |
| Ramosetron vs Tropisetron | 0 | 0 | 1.01 | . | 1.01 | . | . | . |

**Netsplit of“Acute vomiting”**

| comparison | k | prop | nma | direct | indirect | RoR | z | p-value |
| --- | --- | --- | --- | --- | --- | --- | --- | --- |
| Azasetron vs Granisetron | 0 | 0 | 0.7508 | . | 0.7508 | . | . | . |
| Azasetron vs Ondansetron | 1 | 1 | 0.2 | 0.2 | . | . | . | . |
| Azasetron vs Palanosetron | 0 | 0 | 0.4079 | . | 0.4079 | . | . | . |
| Azasetron vs Ramosetron | 0 | 0 | 0.2787 | . | 0.2787 | . | . | . |
| Granisetron vs Ondansetron | 1 | 0.46 | 0.2664 | 0.3333 | 0.2196 | 1.518 | 0.19 | 0.8496 |
| Granisetron vs Palanosetron | 2 | 0.77 | 0.5433 | 0.5166 | 0.6454 | 0.8005 | -0.08 | 0.9327 |
| Granisetron vs Ramosetron | 1 | 0.47 | 0.3712 | 0.3333 | 0.4075 | 0.818 | -0.09 | 0.9275 |
| Palanosetron vs Ondansetron | 7 | 0.76 | 0.4903 | 0.6483 | 0.1996 | 3.2477 | 1.68 | 0.0923 |
| Ramosetron vs Ondansetron | 4 | 0.88 | 0.7177 | 0.6126 | 2.3471 | 0.261 | -1.81 | 0.0703 |
| Palanosetron vs Ramosetron | 3 | 0.68 | 0.6831 | 0.5116 | 1.2473 | 0.4102 | -1.33 | 0.1851 |

**Netsplit of“Late vomiting”**

| comparison | k | prop | nma | direct | indirect | RoR | z | p-value |
| --- | --- | --- | --- | --- | --- | --- | --- | --- |
| Azasetron vs Granisetron | 0 | 0 | 7.0257 | . | 7.0257 | . | . | . |
| Azasetron vs Ondansetron | 1 | 1 | 2 | 2 | . | . | . | . |
| Azasetron vs Palanosetron | 0 | 0 | 3.7903 | . | 3.7903 | . | . | . |
| Azasetron vs Ramosetron | 0 | 0 | 2.6596 | . | 2.6596 | . | . | . |
| Granisetron vs Ondansetron | 2 | 0.84 | 0.2847 | 0.263 | 0.4291 | 0.613 | -0.29 | 0.7717 |
| Granisetron vs Palanosetron | 2 | 0.45 | 0.5395 | 1.0599 | 0.3073 | 3.449 | 0.95 | 0.3418 |
| Granisetron vs Ramosetron | 1 | 0.12 | 0.3786 | 1 | 0.3326 | 3.0064 | 0.52 | 0.6026 |
| Palanosetron vs Ondansetron | 6 | 0.87 | 0.5277 | 0.4973 | 0.7825 | 0.6355 | -0.64 | 0.5194 |
| Ramosetron vs Ondansetron | 3 | 0.78 | 0.752 | 0.8302 | 0.5269 | 1.5757 | 0.63 | 0.5309 |
| Palanosetron vs Ramosetron | 2 | 0.34 | 0.7017 | 0.9572 | 0.5997 | 1.5961 | 0.64 | 0.5221 |

**Netsplit of“>24h vomiting”**

| comparison | k | prop | nma | direct | indirect | RoR | z | p-value |
| --- | --- | --- | --- | --- | --- | --- | --- | --- |
| Azasetron vs Granisetron | 0 | 0 | 8.5205 | . | 8.5205 | . | . | . |
| Azasetron vs Ondansetron | 1 | 1 | 3 | 3 | . | . | . | . |
| Azasetron vs Palanosetron | 0 | 0 | 11.2816 | . | 11.2816 | . | . | . |
| Azasetron vs Ramosetron | 0 | 0 | 6.4351 | . | 6.4351 | . | . | . |
| Granisetron vs Ondansetron | 0 | 0 | 0.3521 | . | 0.3521 | . | . | . |
| Granisetron vs Palanosetron | 1 | 0.79 | 1.3241 | 1 | 3.8707 | 0.2583 | -0.31 | 0.7563 |
| Granisetron vs Ramosetron | 1 | 0.79 | 0.7553 | 1 | 0.2583 | 3.8707 | 0.31 | 0.7563 |
| Palanosetron vs Ondansetron | 3 | 0.65 | 0.2659 | 0.3537 | 0.1562 | 2.2648 | 0.5 | 0.6195 |
| Ramosetron vs Ondansetron | 2 | 0.89 | 0.4662 | 0.4275 | 0.9681 | 0.4415 | -0.5 | 0.6195 |
| Palanosetron vs Ramosetron | 2 | 0.46 | 0.5704 | 0.3654 | 0.8275 | 0.4415 | -0.5 | 0.6195 |

**Netsplit of“Overall vomiting”**

| comparison | k | prop | nma | direct | indirect | RoR | z | p-value |
| --- | --- | --- | --- | --- | --- | --- | --- | --- |
| Azasetron vs Granisetron | 0 | 0 | 1.73 | . | 1.73 | . | . | . |
| Azasetron vs Ondansetron | 1 | 1 | 0.62 | 0.62 | . | . | . | . |
| Azasetron vs Palanosetron | 0 | 0 | 1.11 | . | 1.11 | . | . | . |
| Azasetron vs Ramosetron | 0 | 0 | 0.52 | . | 0.52 | . | . | . |
| Azasetron vs Tropisetron | 0 | 0 | 0.81 | . | 0.81 | . | . | . |
| Granisetron vs Ondansetron | 3 | 0.99 | 0.36 | 0.33 | 392.07 | 0 | -1.45 | 0.147 |
| Granisetron vs Palanosetron | 1 | 0.25 | 0.65 | 2 | 0.44 | 4.52 | 1.02 | 0.3055 |
| Granisetron vs Ramosetron | 0 | 0 | 0.3 | . | 0.3 | . | . | . |
| Granisetron vs Tropisetron | 0 | 0 | 0.47 | . | 0.47 | . | . | . |
| Palanosetron vs Ondansetron | 5 | 0.8 | 0.56 | 0.58 | 0.47 | 1.23 | 0.26 | 0.7985 |
| Ramosetron vs Ondansetron | 2 | 0.72 | 1.2 | 1.12 | 1.43 | 0.78 | -0.3 | 0.7655 |
| Tropisetron vs Ondansetron | 2 | 1 | 0.77 | 0.77 | . | . | . | . |
| Palanosetron vs Ramosetron | 1 | 0.47 | 0.47 | 0.41 | 0.53 | 0.78 | -0.3 | 0.7655 |
| Palanosetron vs Tropisetron | 0 | 0 | 0.73 | . | 0.73 | . | . | . |
| Ramosetron vs Tropisetron | 0 | 0 | 1.56 | . | 1.56 | . | . | . |

**Netsplit of“Acute PONV”**

| comparison | k | prop | nma | direct | indirect | RoR | z | p-value |
| --- | --- | --- | --- | --- | --- | --- | --- | --- |
| Dolasetron vs Granisetron | 1 | 0.85 | 1.5 | 1.48 | 1.63 | 0.91 | -0.1 | 0.9203 |
| Dolasetron vs Ondansetron | 1 | 0.88 | 1.28 | 1.29 | 1.17 | 1.11 | 0.1 | 0.9203 |
| Dolasetron vs Palanosetron | 0 | 0 | 1.61 | . | 1.61 | . | . | . |
| Dolasetron vs Ramosetron | 0 | 0 | 1.34 | . | 1.34 | . | . | . |
| Granisetron vs Ondansetron | 3 | 1 | 0.85 | 0.85 | 913.37 | 0 | -0.75 | 0.4519 |
| Granisetron vs Palanosetron | 1 | 0.03 | 1.07 | 1 | 1.08 | 0.93 | -0.04 | 0.9708 |
| Granisetron vs Ramosetron | 0 | 0 | 0.89 | . | 0.89 | . | . | . |
| Palanosetron vs Ondansetron | 5 | 0.72 | 0.79 | 0.69 | 1.12 | 0.62 | -1.09 | 0.275 |
| Ramosetron vs Ondansetron | 2 | 0.65 | 0.95 | 1.12 | 0.71 | 1.58 | 1.05 | 0.2917 |
| Palanosetron vs Ramosetron | 2 | 0.63 | 0.83 | 0.99 | 0.62 | 1.58 | 1.05 | 0.2917 |

**Netsplit of“Late PONV”**

| comparison | k | prop | nma | direct | indirect | RoR | z | p-value |
| --- | --- | --- | --- | --- | --- | --- | --- | --- |
| Dolasetron vs Granisetron | 1 | 0.91 | 1.11 | 1.06 | 1.64 | 0.65 | -0.38 | 0.7054 |
| Dolasetron vs Ondansetron | 1 | 0.93 | 0.91 | 0.94 | 0.59 | 1.6 | 0.38 | 0.7054 |
| Dolasetron vs Palanosetron | 0 | 0 | 1.23 | . | 1.23 | . | . | . |
| Dolasetron vs Ramosetron | 0 | 0 | 1.06 | . | 1.06 | . | . | . |
| Granisetron vs Ondansetron | 3 | 0.98 | 0.82 | 0.78 | 13.08 | 0.06 | -1.37 | 0.1704 |
| Granisetron vs Palanosetron | 1 | 0.15 | 1.11 | 2.5 | 0.96 | 2.61 | 1.08 | 0.278 |
| Granisetron vs Ramosetron | 0 | 0 | 0.96 | . | 0.96 | . | . | . |
| Palanosetron vs Ondansetron | 5 | 0.87 | 0.74 | 0.7 | 1.14 | 0.61 | -1.2 | 0.2302 |
| Ramosetron vs Ondansetron | 2 | 0.36 | 0.86 | 1.17 | 0.72 | 1.63 | 1.19 | 0.2359 |
| Palanosetron vs Ramosetron | 2 | 0.77 | 0.87 | 0.97 | 0.59 | 1.63 | 1.19 | 0.2359 |

**Netsplit of“>24h PONV”**

| comparison | k | prop | nma | direct | indirect | RoR | z | p-value |
| --- | --- | --- | --- | --- | --- | --- | --- | --- |
| Palanosetron vs Ondansetron | 2 | 0.93 | 0.6826 | 0.7815 | 0.1209 | 6.4629 | 1.63 | 0.1033 |
| Ramosetron vs Ondansetron | 1 | 0.13 | 0.6377 | 0.125 | 0.8079 | 0.1547 | -1.63 | 0.1033 |
| Palanosetron vs Ramosetron | 2 | 0.95 | 1.0705 | 0.9674 | 6.2519 | 0.1547 | -1.63 | 0.1033 |

**Netsplit of“Overall PONV”**

| comparison | k | prop | nma | direct | indirect | RoR | z | p-value |
| --- | --- | --- | --- | --- | --- | --- | --- | --- |
| Azasetron vs Dolasetron | 0 | 0 | 0.6558 | . | 0.6558 | . | . | . |
| Azasetron vs Granisetron | 0 | 0 | 0.9542 | . | 0.9542 | . | . | . |
| Azasetron vs Ondansetron | 1 | 1 | 0.75 | 0.75 | . | . | . | . |
| Azasetron vs Palanosetron | 0 | 0 | 0.9408 | . | 0.9408 | . | . | . |
| Azasetron vs Ramosetron | 0 | 0 | 0.8619 | . | 0.8619 | . | . | . |
| Dolasetron vs Granisetron | 1 | 0.84 | 1.455 | 1.2525 | 3.2409 | 0.3865 | -1.4 | 0.163 |
| Dolasetron vs Ondansetron | 1 | 0.84 | 1.1437 | 1.3333 | 0.5203 | 2.5629 | 1.4 | 0.163 |
| Dolasetron vs Palanosetron | 0 | 0 | 1.4346 | . | 1.4346 | . | . | . |
| Dolasetron vs Ramosetron | 0 | 0 | 1.3144 | . | 1.3144 | . | . | . |
| Granisetron vs Ondansetron | 4 | 0.71 | 0.786 | 0.7676 | 0.8327 | 0.9218 | -0.19 | 0.8462 |
| Granisetron vs Palanosetron | 2 | 0.38 | 0.986 | 0.9572 | 1.0038 | 0.9536 | -0.11 | 0.9114 |
| Granisetron vs Ramosetron | 1 | 0.26 | 0.9033 | 1.2222 | 0.8118 | 1.5055 | 0.84 | 0.4024 |
| Palanosetron vs Ondansetron | 5 | 0.75 | 0.7972 | 0.7381 | 1.0031 | 0.7358 | -1.13 | 0.2574 |
| Ramosetron vs Ondansetron | 4 | 0.7 | 0.8701 | 0.9845 | 0.6534 | 1.5069 | 1.49 | 0.136 |
| Palanosetron vs Ramosetron | 3 | 0.67 | 0.9162 | 1.0408 | 0.7053 | 1.4758 | 1.38 | 0.1688 |

**Netsplit of“Acute rescue medicine”**

| comparison | k | prop | nma | direct | indirect | RoR | z | p-value |
| --- | --- | --- | --- | --- | --- | --- | --- | --- |
| Azasetron vs Granisetron | 0 | 0 | 1.8985 | . | 1.8985 | . | . | . |
| Azasetron vs Ondansetron | 1 | 1 | 1 | 1 | . | . | . | . |
| Azasetron vs Palanosetron | 0 | 0 | 1.0097 | . | 1.0097 | . | . | . |
| Azasetron vs Ramosetron | 0 | 0 | 1.2268 | . | 1.2268 | . | . | . |
| Granisetron vs Ondansetron | 1 | 0.33 | 0.5267 | 0.3333 | 0.661 | 0.5043 | -0.35 | 0.7286 |
| Granisetron vs Palanosetron | 2 | 0.81 | 0.5318 | 0.6022 | 0.3119 | 1.9309 | 0.28 | 0.7808 |
| Granisetron vs Ramosetron | 1 | 0.45 | 0.6462 | 1 | 0.4493 | 2.2258 | 0.42 | 0.6715 |
| Palanosetron vs Ondansetron | 4 | 0.92 | 0.9904 | 0.9737 | 1.2199 | 0.7981 | -0.26 | 0.7937 |
| Ramosetron vs Ondansetron | 5 | 0.93 | 0.8151 | 0.8197 | 0.7532 | 1.0883 | 0.09 | 0.9258 |
| Palanosetron vs Ramosetron | 2 | 0.48 | 1.2151 | 1.2239 | 1.2071 | 1.0139 | 0.02 | 0.9811 |

**Netsplit of“Late rescue medicine”**

| comparison | k | prop | nma | direct | indirect | RoR | z | p-value |
| --- | --- | --- | --- | --- | --- | --- | --- | --- |
| Azasetron vs Granisetron | 0 | 0 | 3.5424 | . | 3.5424 | . | . | . |
| Azasetron vs Ondansetron | 1 | 1 | 1 | 1 | . | . | . | . |
| Azasetron vs Palanosetron | 0 | 0 | 1.7748 | . | 1.7748 | . | . | . |
| Azasetron vs Ramosetron | 0 | 0 | 1.1851 | . | 1.1851 | . | . | . |
| Granisetron vs Ondansetron | 1 | 0.81 | 0.2823 | 0.25 | 0.4715 | 0.5302 | -0.37 | 0.7102 |
| Granisetron vs Palanosetron | 2 | 0.51 | 0.501 | 1.0599 | 0.2288 | 4.6326 | 1.11 | 0.2652 |
| Granisetron vs Ramosetron | 1 | 0.14 | 0.3345 | 1 | 0.2815 | 3.5519 | 0.59 | 0.5531 |
| Palanosetron vs Ondansetron | 3 | 0.98 | 0.5634 | 0.551 | 1.7338 | 0.3178 | -0.82 | 0.4123 |
| Ramosetron vs Ondansetron | 4 | 0.96 | 0.8438 | 0.8924 | 0.2014 | 4.4313 | 0.92 | 0.3564 |
| Palanosetron vs Ramosetron | 1 | 0.05 | 0.6677 | 3 | 0.6174 | 4.859 | 0.95 | 0.3402 |

**Netsplit of“>24h rescue medicine”**

| comparison | k | prop | nma | direct | indirect | RoR | z | p-value |
| --- | --- | --- | --- | --- | --- | --- | --- | --- |
| Azasetron vs Granisetron | 0 | 0 | 10.2259 | . | 10.2259 | . | . | . |
| Azasetron vs Ondansetron | 1 | 1 | 3 | 3 | . | . | . | . |
| Azasetron vs Palanosetron | 0 | 0 | 7.414 | . | 7.414 | . | . | . |
| Azasetron vs Ramosetron | 0 | 0 | 14.1044 | . | 14.1044 | . | . | . |
| Granisetron vs Ondansetron | 0 | 0 | 0.2934 | . | 0.2934 | . | . | . |
| Granisetron vs Palanosetron | 1 | 0.8 | 0.725 | 1 | 0.2018 | 4.9566 | 0.36 | 0.7179 |
| Granisetron vs Ramosetron | 1 | 0.8 | 1.3793 | 1 | 4.9566 | 0.2018 | -0.36 | 0.7179 |
| Palanosetron vs Ondansetron | 2 | 0.96 | 0.4046 | 0.417 | 0.1873 | 2.2263 | 0.36 | 0.7179 |
| Ramosetron vs Ondansetron | 2 | 0.84 | 0.2127 | 0.1873 | 0.417 | 0.4492 | -0.36 | 0.7179 |
| Palanosetron vs Ramosetron | 1 | 0.2 | 1.9024 | 1 | 2.2263 | 0.4492 | -0.36 | 0.7179 |

**Netsplit of“Overall rescue medicine”**

| comparison | k | prop | nma | direct | indirect | RoR | z | p-value |
| --- | --- | --- | --- | --- | --- | --- | --- | --- |
| Granisetron vs Ondansetron | 2 | 0.99 | 0.3264 | 0.2820 1 | 144.1181 | 0 | -1.85 | 0.0643 |
| Granisetron vs Palanosetron | 1 | 0.33 | 0.4182 | 2 | 0.195 | 10.2588 | 1.55 | 0.1219 |
| Granisetron vs Ramosetron | 0 | 0 | 0.4714 | . | 0.4714 | . | . | . |
| Granisetron vs Tropisetron | 0 | 0 | 0.4119 | . | 0.4119 | . | . | . |
| Palanosetron vs Ondansetron | 4 | 0.84 | 0.7804 | 0.8885 | 0.3876 | 2.2923 | 1.15 | 0.2492 |
| Ramosetron vs Ondansetron | 2 | 0.72 | 0.6923 | 0.5455 | 1.2723 | 0.4288 | -1.18 | 0.2399 |
| Tropisetron vs Ondansetron | 2 | 1 | 0.7924 | 0.7924 | . | . | . | . |
| Palanosetron vs Ramosetron | 1 | 0.44 | 1.1272 | 0.7 | 1.6326 | 0.4288 | -1.18 | 0.2399 |
| Palanosetron vs Tropisetron | 0 | 0 | 0.9849 | . | 0.9849 | . | . | . |
| Ramosetron vs Tropisetron | 0 | 0 | 0.8738 | . | 0.8738 | . | . | . |

**Netsplit of“Adverse reaction”**

| comparison | k | prop | nma | direct | indirect | RoR | z | p-value |
| --- | --- | --- | --- | --- | --- | --- | --- | --- |
| Azasetron vs Granisetron | 0 | 0 | 0.7623 | . | 0.7623 | . | . | . |
| Azasetron vs Ondansetron | 1 | 1 | 0.9167 | 0.9167 | . | . | . | . |
| Azasetron vs Palanosetron | 0 | 0 | 0.9776 | . | 0.9776 | . | . | . |
| Azasetron vs Ramosetron | 0 | 0 | 0.9392 | . | 0.9392 | . | . | . |
| Azasetron vs Tropisetron | 0 | 0 | 1.0476 | . | 1.0476 | . | . | . |
| Granisetron vs Ondansetron | 4 | 0.95 | 1.2025 | 1.0833 | 8.8374 | 0.1226 | -1.22 | 0.2234 |
| Granisetron vs Palanosetron | 1 | 0.13 | 1.2825 | 5 | 1.0454 | 4.7827 | 1.35 | 0.1786 |
| Granisetron vs Ramosetron | 0 | 0 | 1.232 | . | 1.232 | . | . | . |
| Granisetron vs Tropisetron | 0 | 0 | 1.3743 | . | 1.3743 | . | . | . |
| Palanosetron vs Ondansetron | 7 | 0.89 | 0.9376 | 0.9594 | 0.7781 | 1.2329 | 0.52 | 0.6023 |
| Ramosetron vs Ondansetron | 5 | 0.76 | 0.976 | 0.9415 | 1.0925 | 0.8617 | -0.39 | 0.6994 |
| Tropisetron vs Ondansetron | 1 | 1 | 0.875 | 0.875 | . | . | . | . |
| Palanosetron vs Ramosetron | 2 | 0.55 | 0.9607 | 0.9648 | 0.9555 | 1.0097 | 0.03 | 0.9783 |
| Palanosetron vs Tropisetron | 0 | 0 | 1.0716 | . | 1.0716 | . | . | . |
| Ramosetron vs Tropisetron | 0 | 0 | 1.1155 | . | 1.1155 | . | . | . |
