# Supplementary material for: 5‐HT3 receptor antagonists for preventing postoperative nausea and vomiting after gynecological surgery: A systematic review and network meta‐analysis
Source: Int J Gynaecol Obstet. 2025 May 9;171(1):177–89. doi: 10.1002/ijgo.70197 (PMC12447676; doi:10.1002/ijgo.70197)

**Data S4 Funnel plot**

**Funnel plot and the value of Egger’s test of “Acute nausea”**


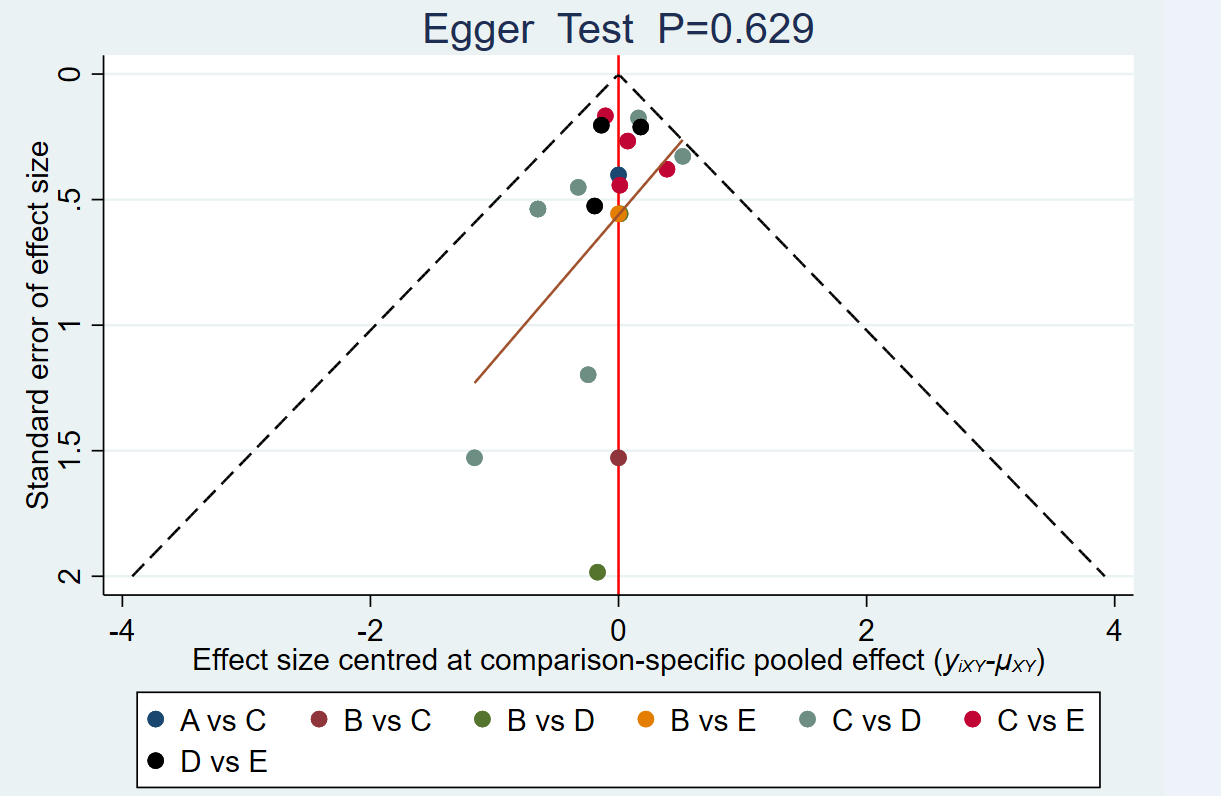


**Funnel plot and the value of Egger’s test of “Late nausea”**


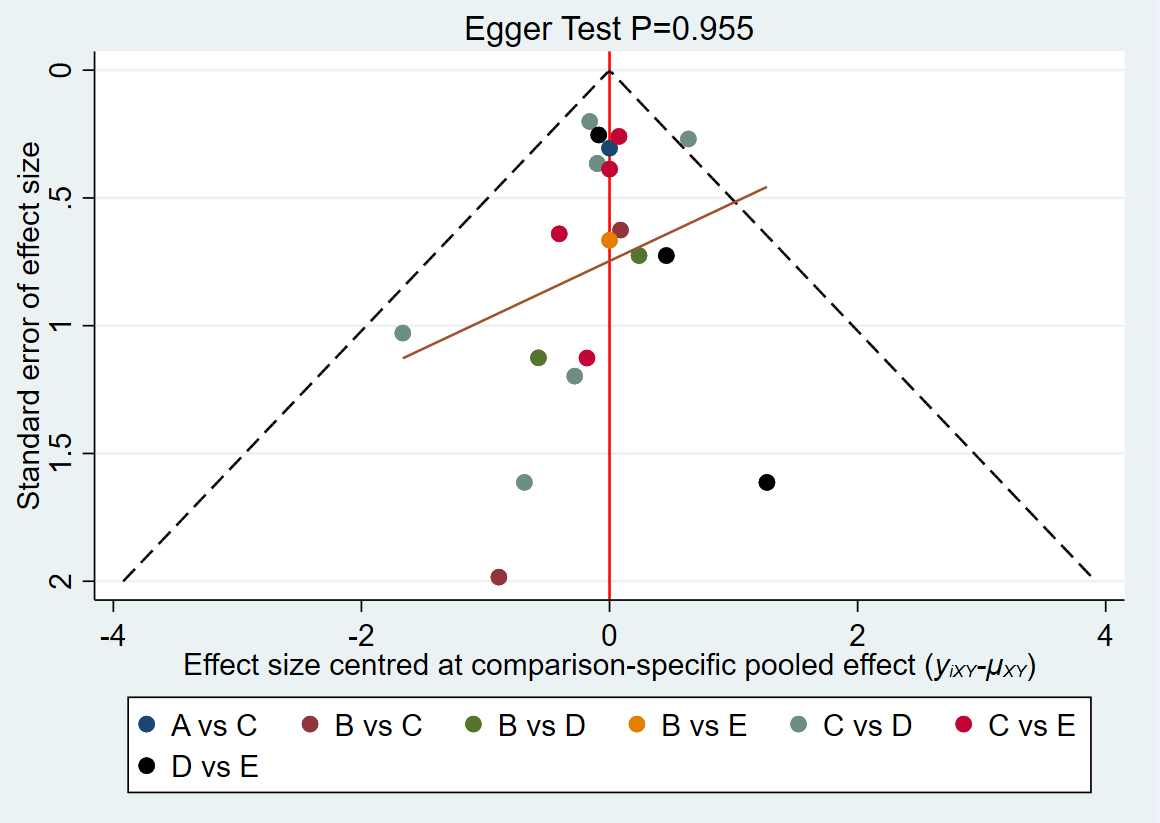


**Funnel plot and the value of Egger’s test of “Overall nausea”**


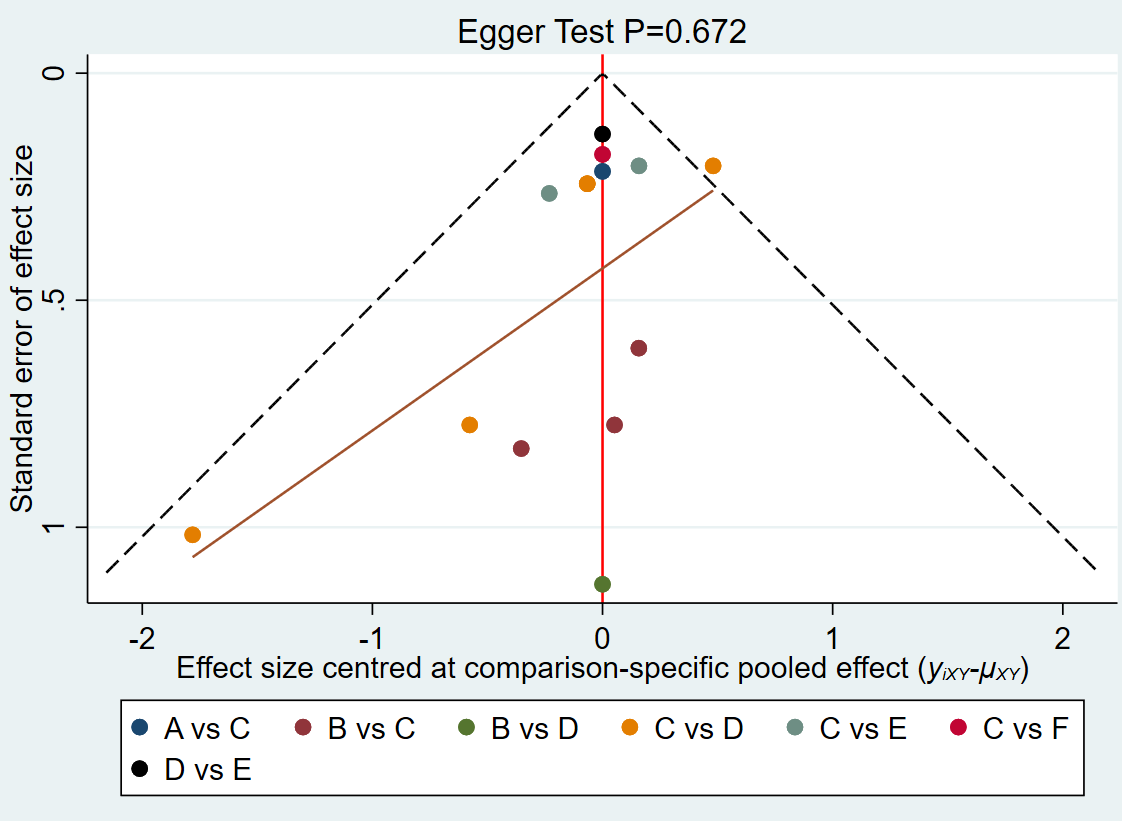


**Funnel plot and the value of Egger’s test of “Acute vomiting”**


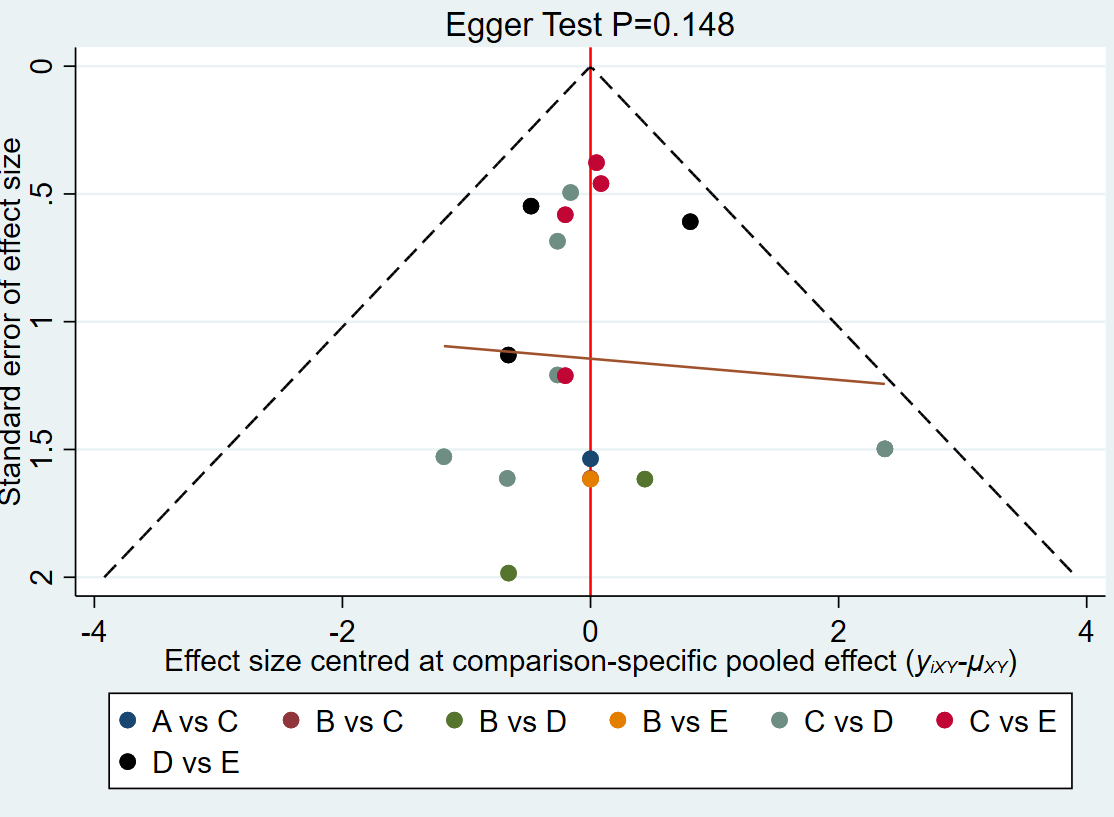


**Funnel plot and the value of Egger’s test of “****Late vomiting”**


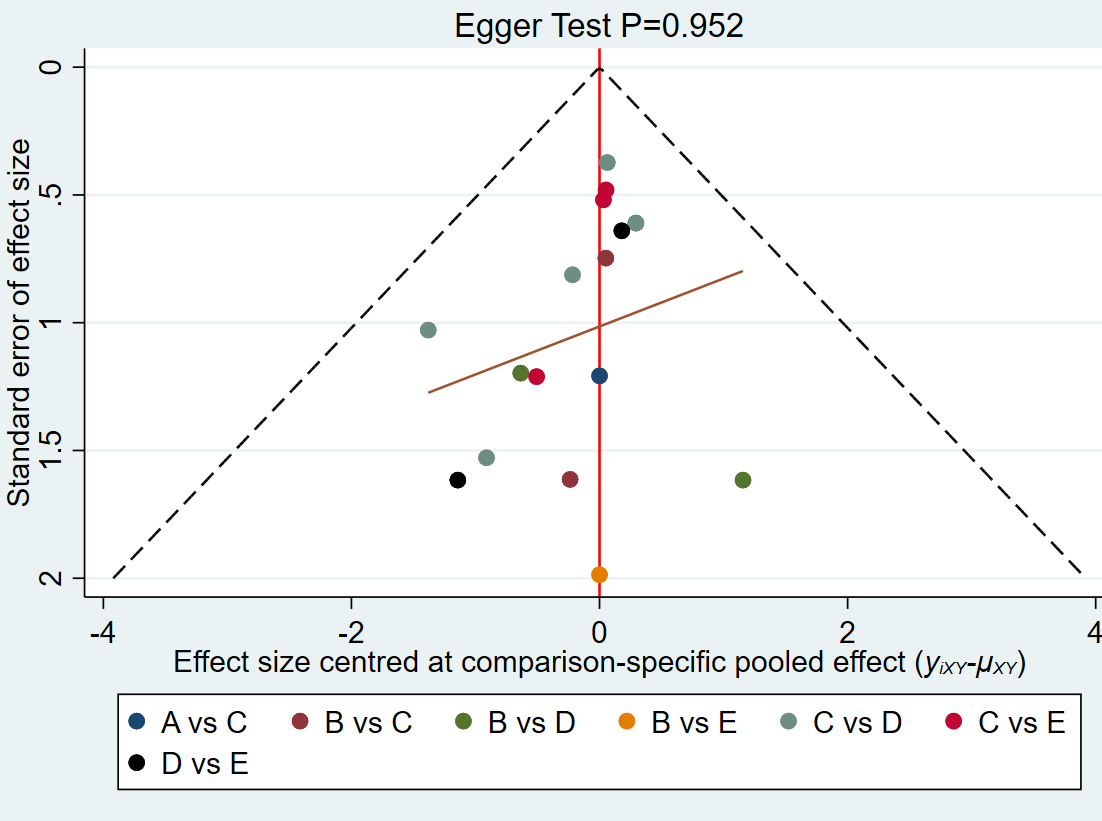


**Funnel plot and the value of Egger’s test of “Overall vomiting”**


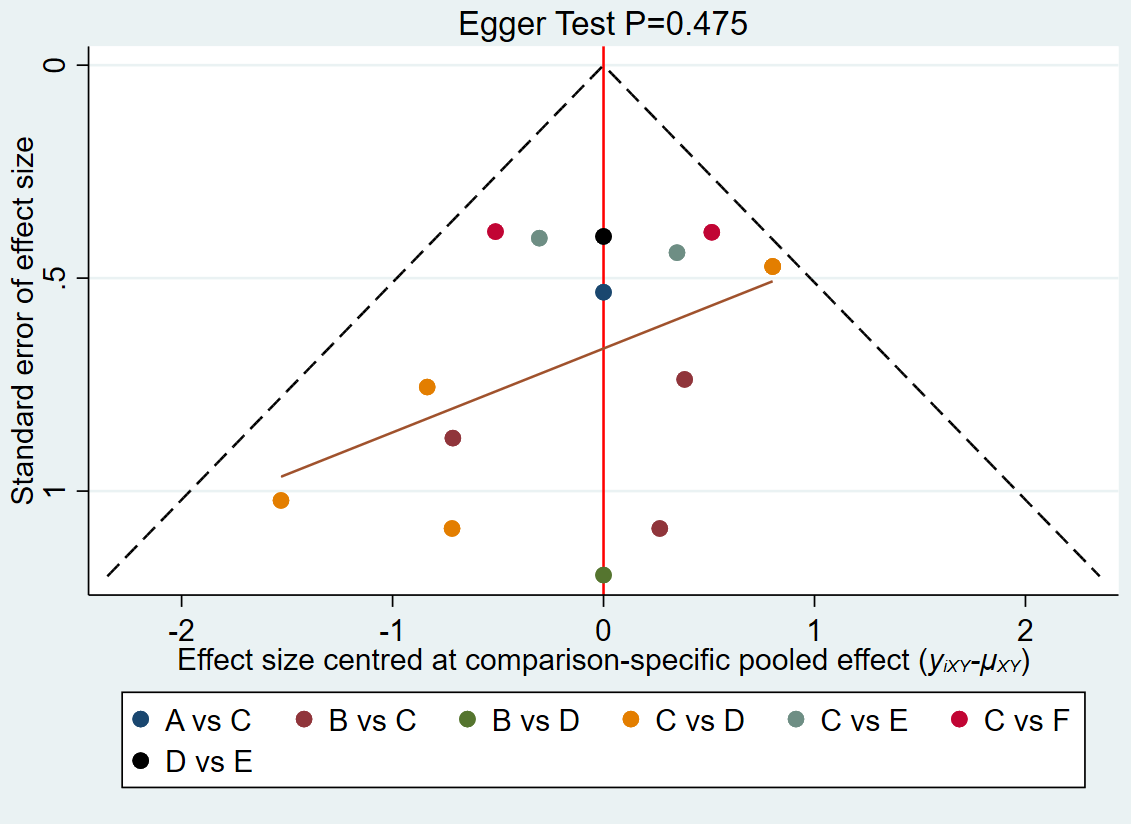


**Funnel plot and the value of Egger’s test of “****Acute PONV”**


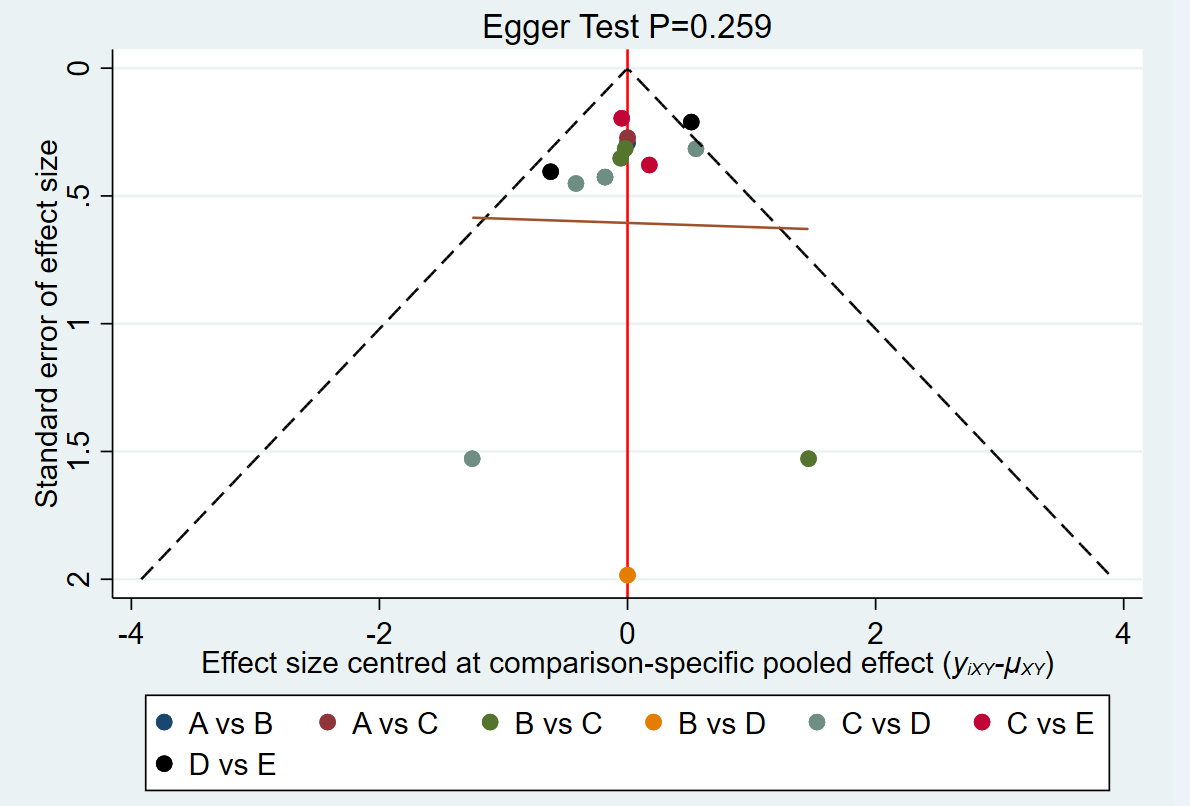


**Funnel plot and the value of Egger’s test of “****Late PONV”**


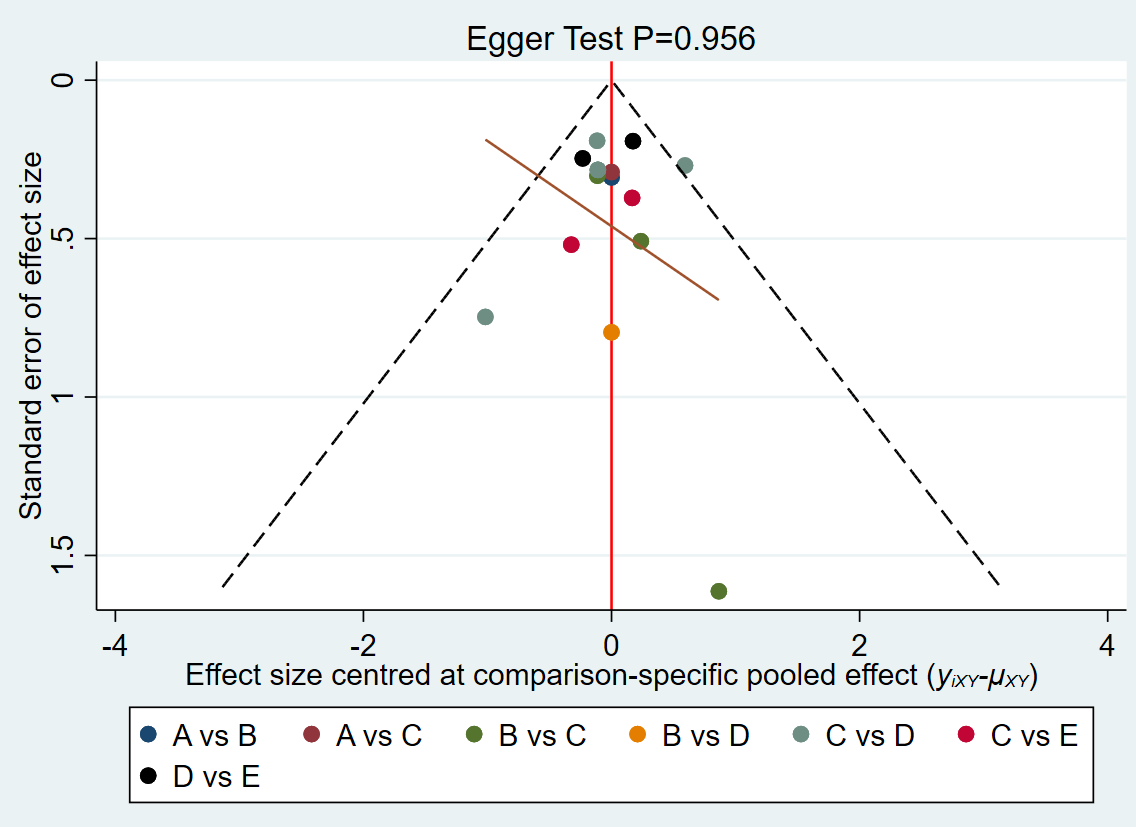


**Funnel plot and the value of Egger’s test of “Overall PONV”**


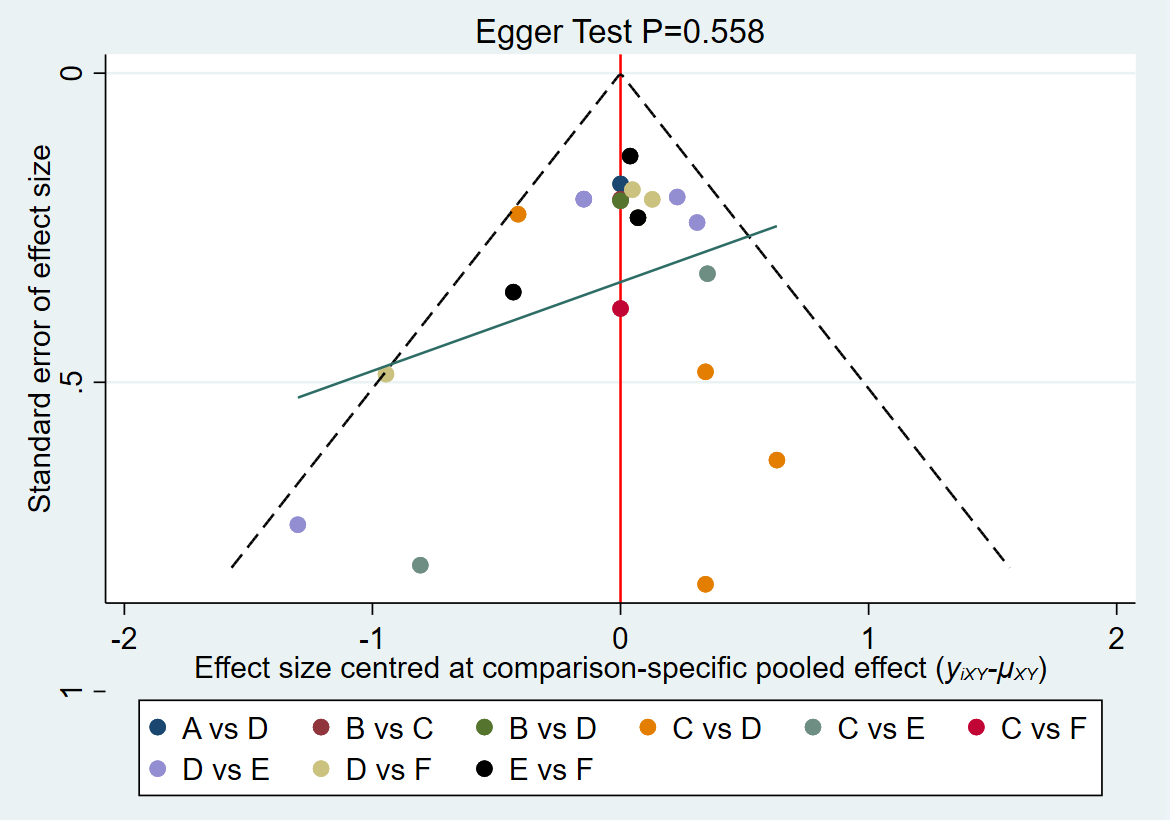


**Funnel plot and the value of Egger’s test of “Acute rescue medicine”**


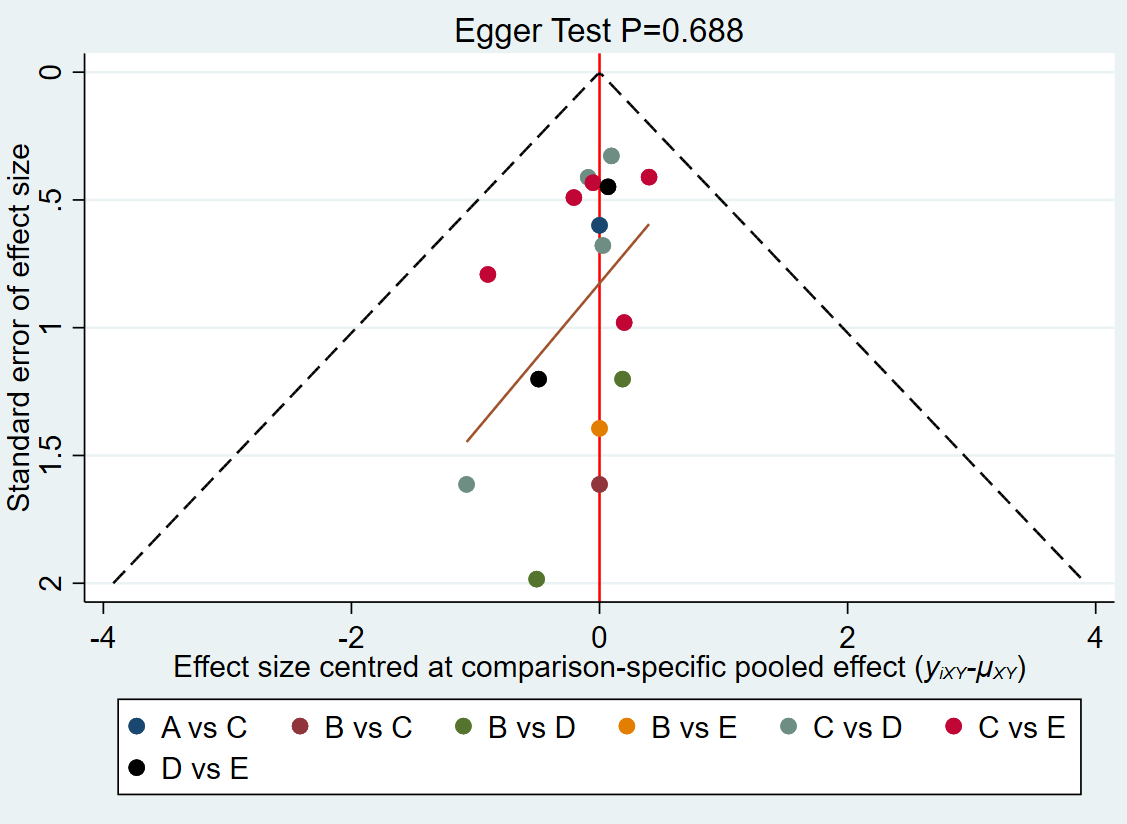


**Funnel plot and the value of Egger’s test of “Overall rescue medicine”**


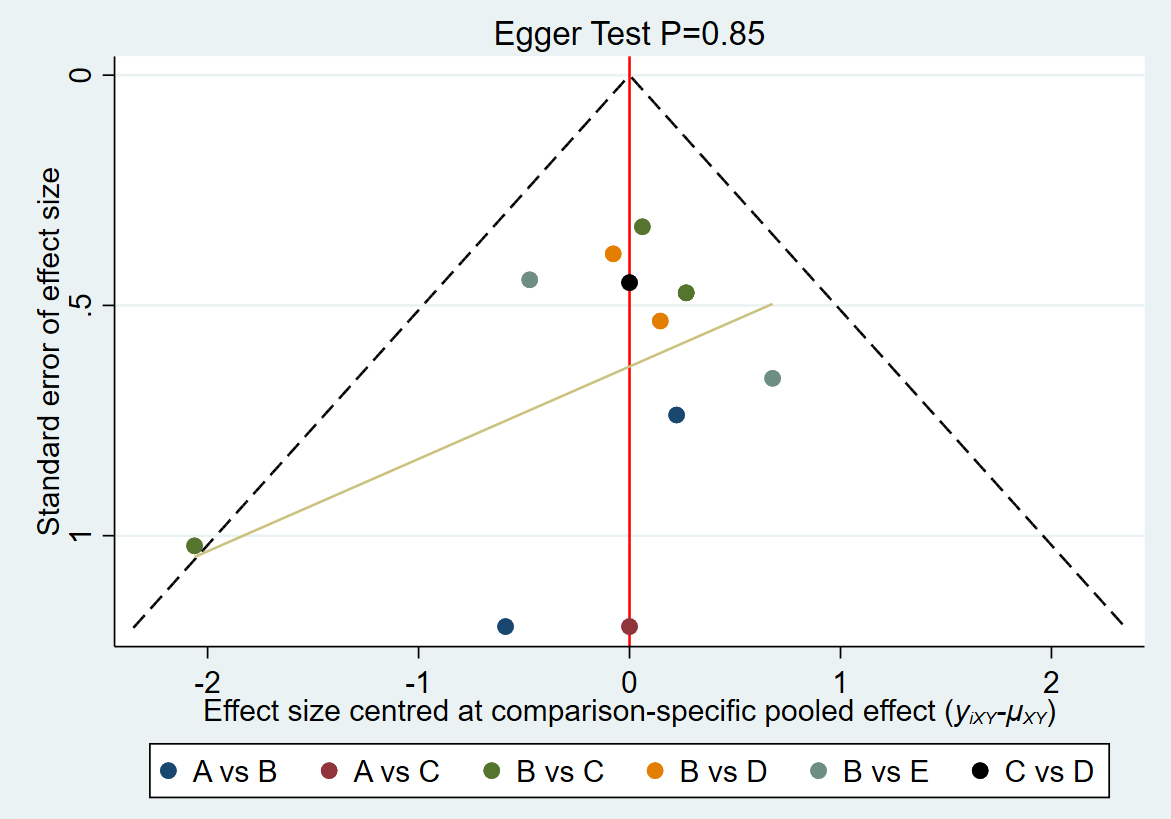


**Funnel plot and the value of Egger’s test of “Adverse reaction”**


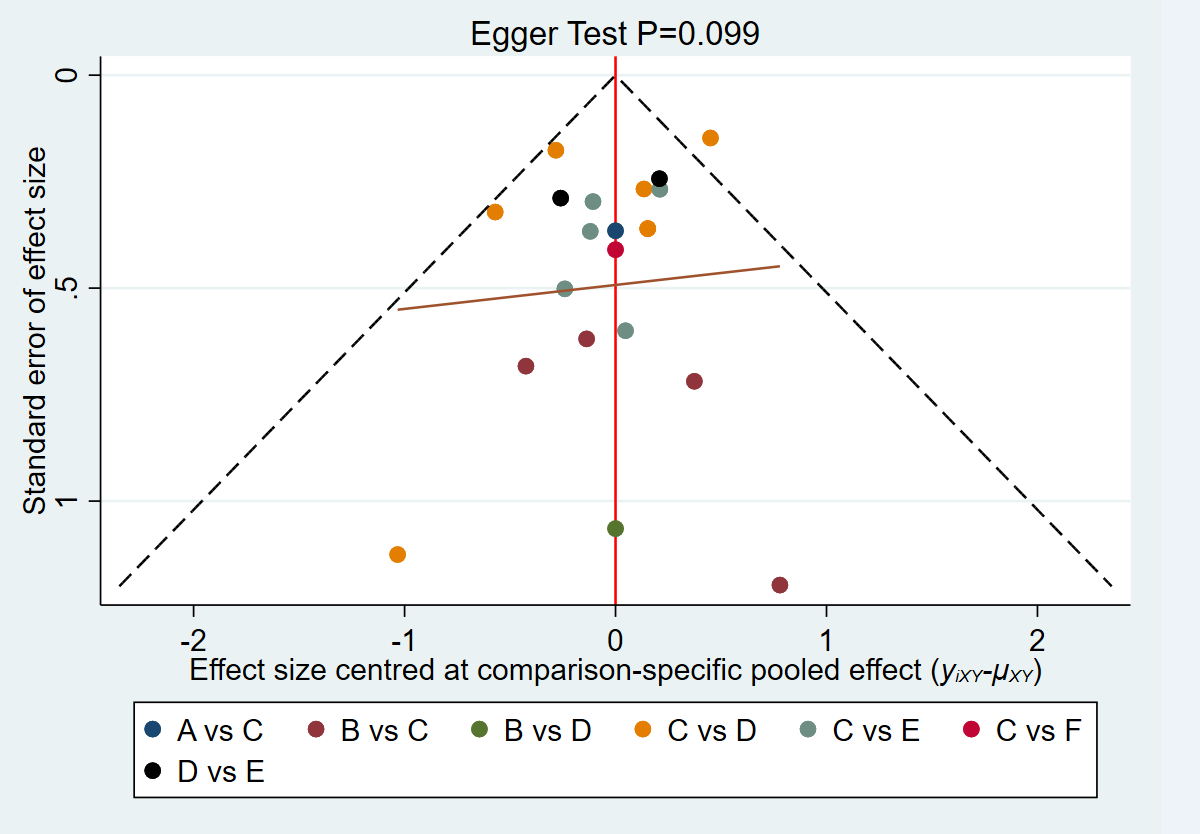

Supplement: Supplementary file 4 — Data S4. [file IJGO-171-177-s012.docx]
