# Supplementary material for: 5‐HT3 receptor antagonists for preventing postoperative nausea and vomiting after gynecological surgery: A systematic review and network meta‐analysis
Source: Int J Gynaecol Obstet. 2025 May 9;171(1):177–89. doi: 10.1002/ijgo.70197 (PMC12447676; doi:10.1002/ijgo.70197)

**Data S5 Network graph of the outcomes**

**Network graph of “Acute nausea”**


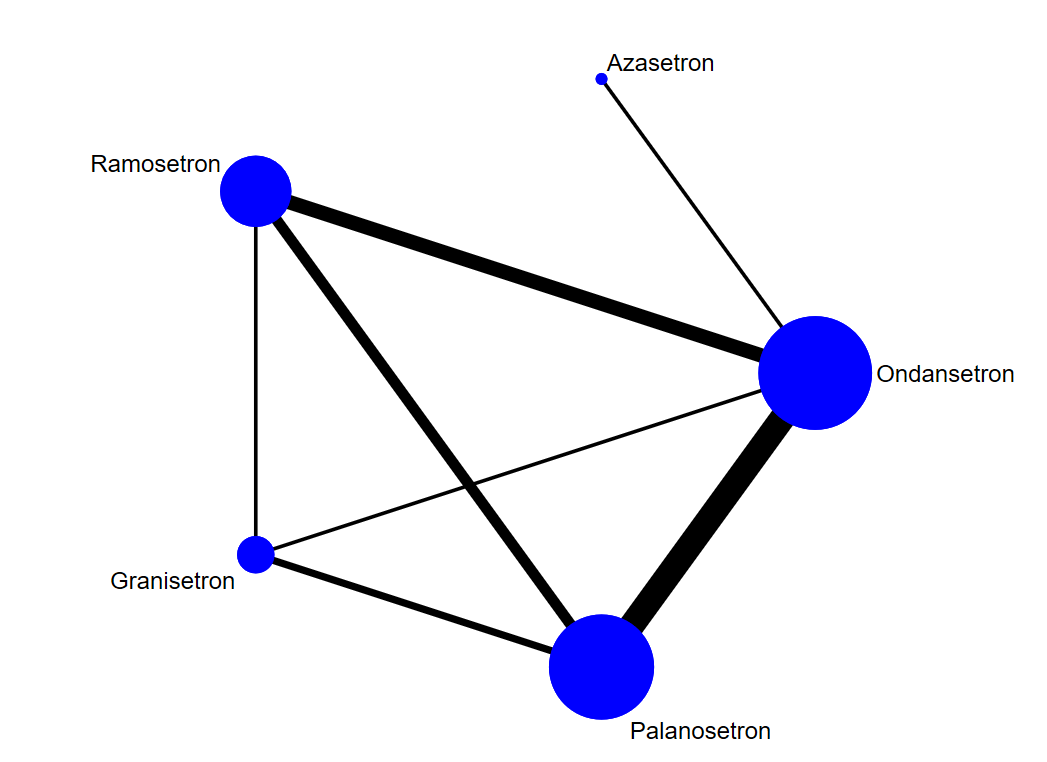


**Network graph of “Late nausea”**


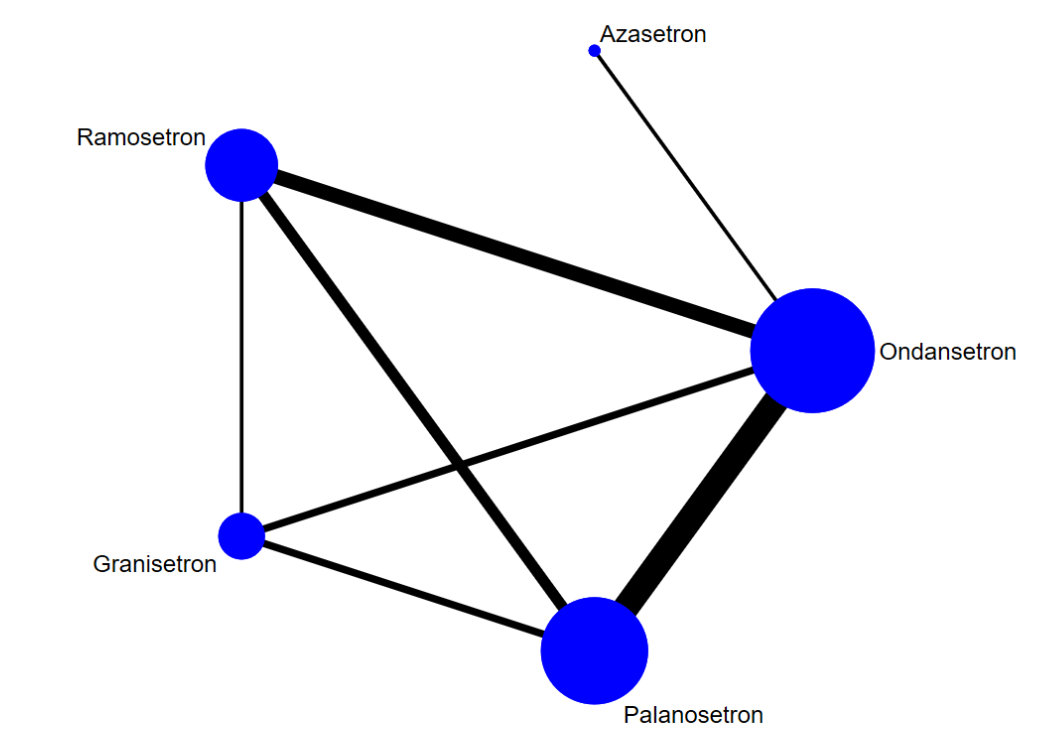


**Network graph of “>24h nausea”**


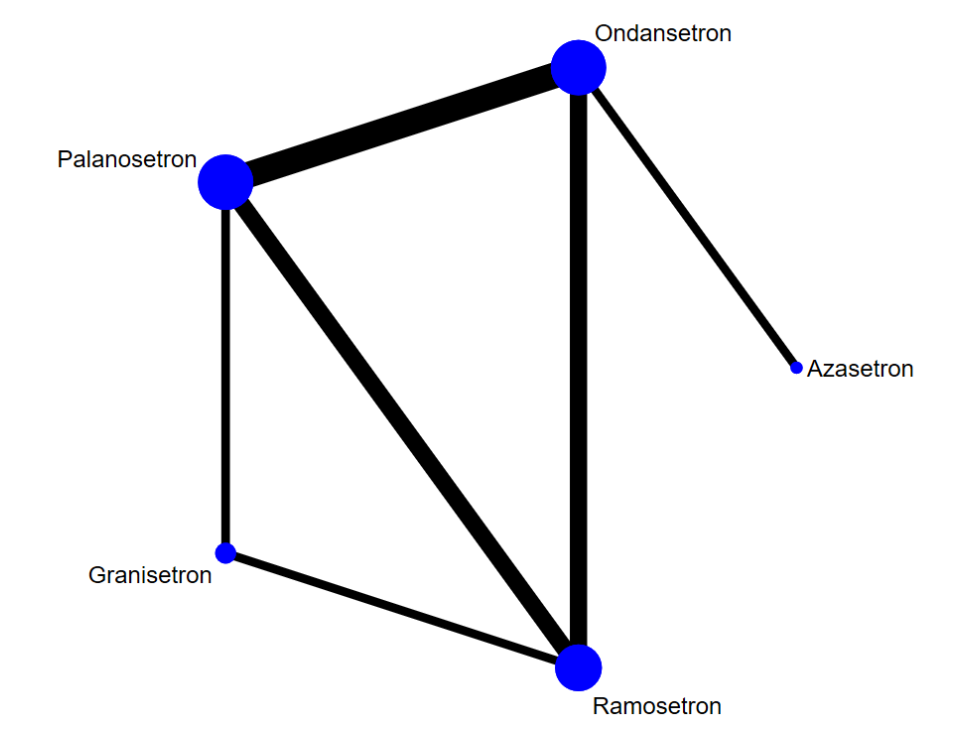


**Network graph of “Overall nausea”**


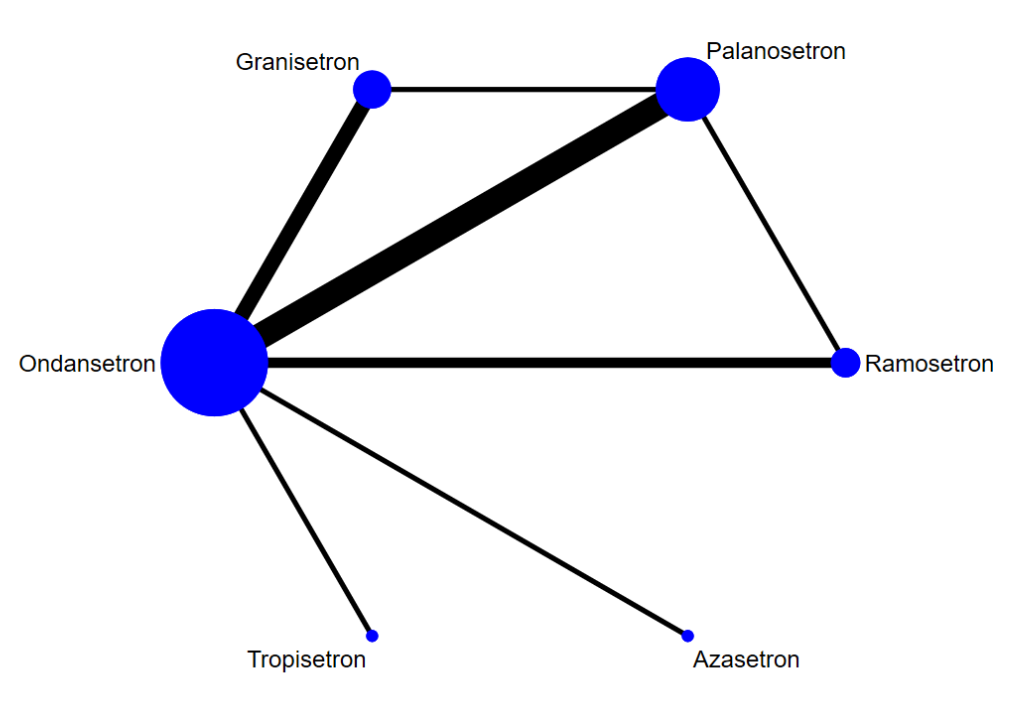


**Network graph of “Acute vomiting”**


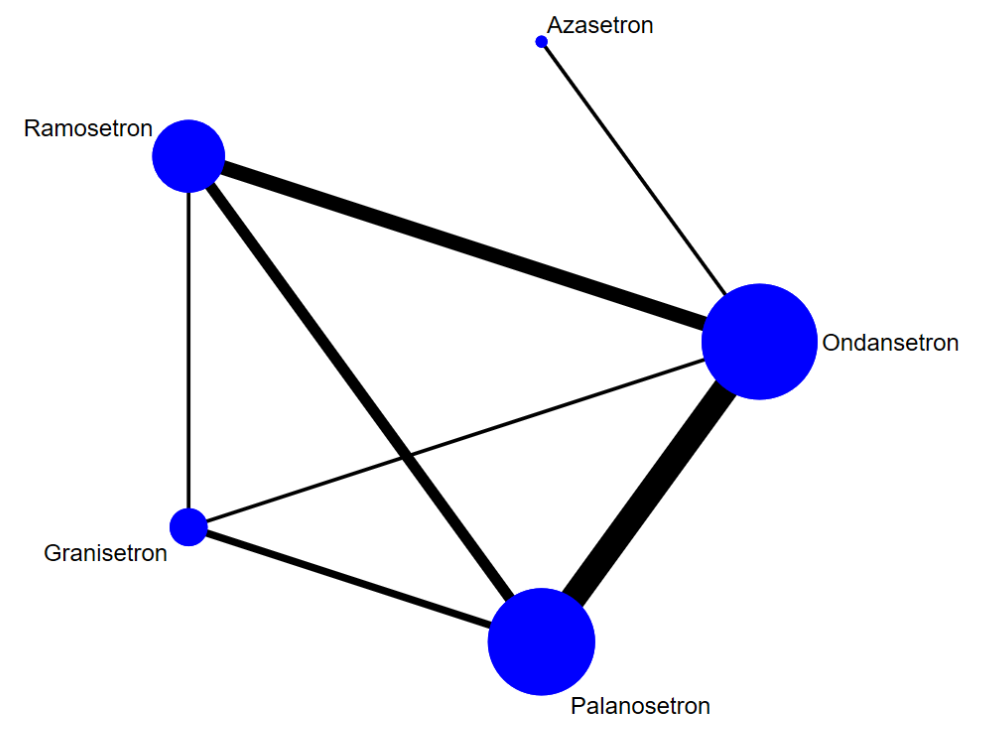


**Network graph of “****Late vomiting”**


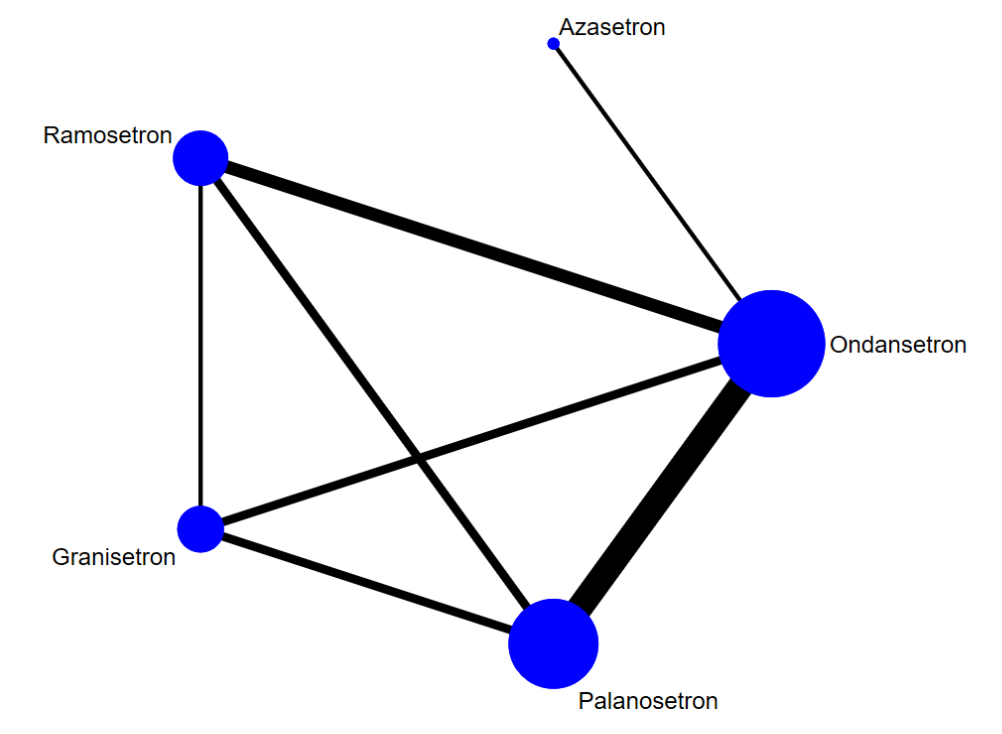


**Network graph of “>24h vomiting”**


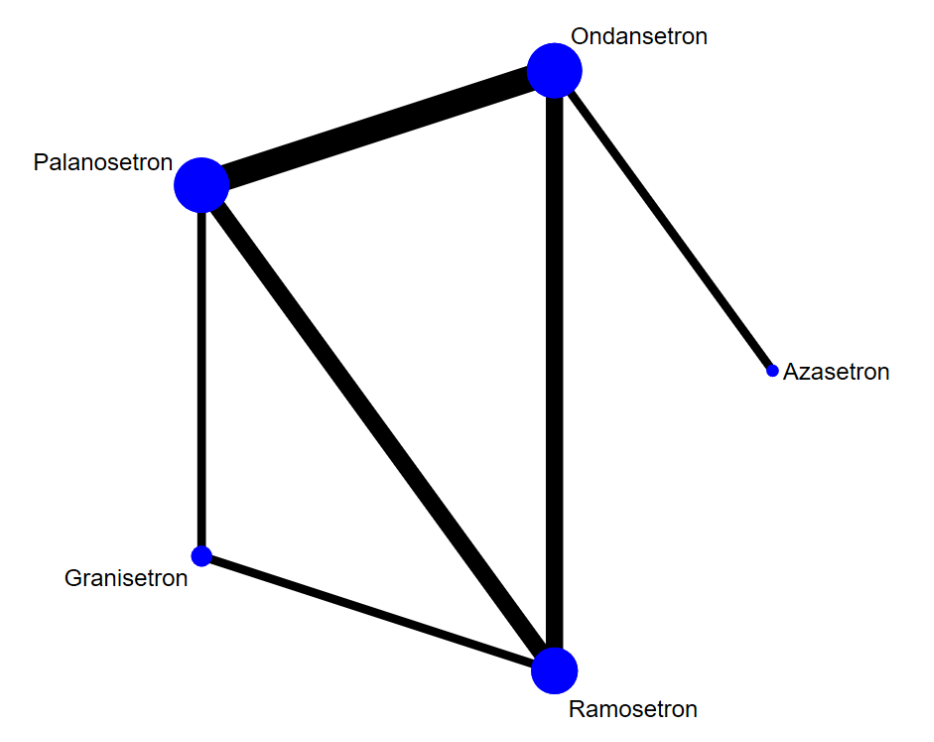


**Network graph of “Overall vomiting”**


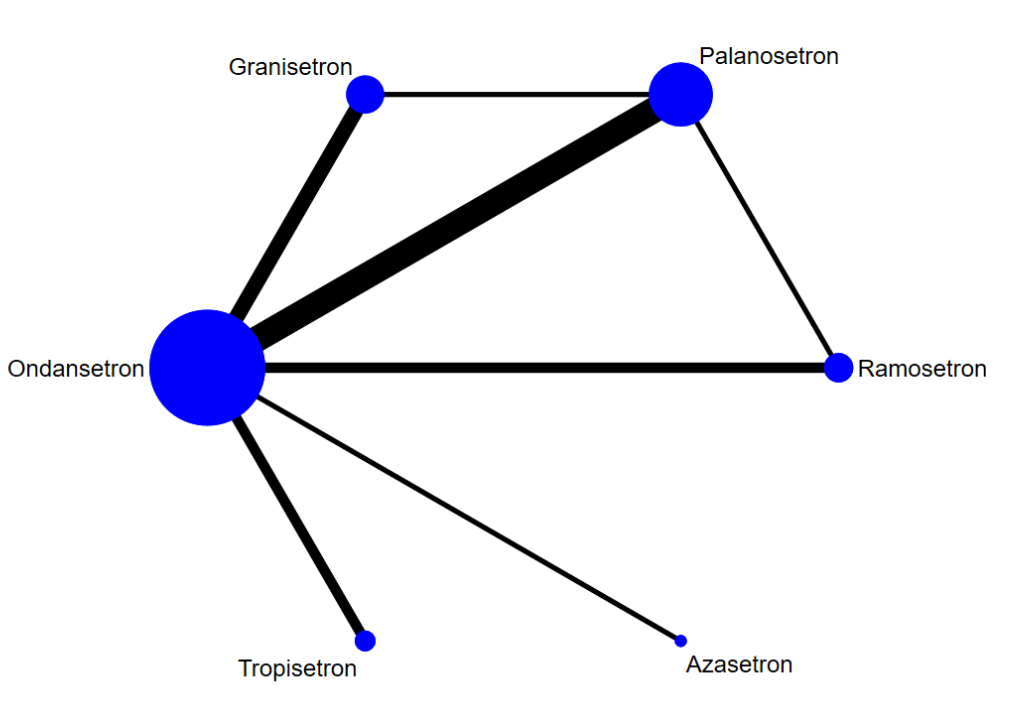


**Network graph of “****Acute PONV”**


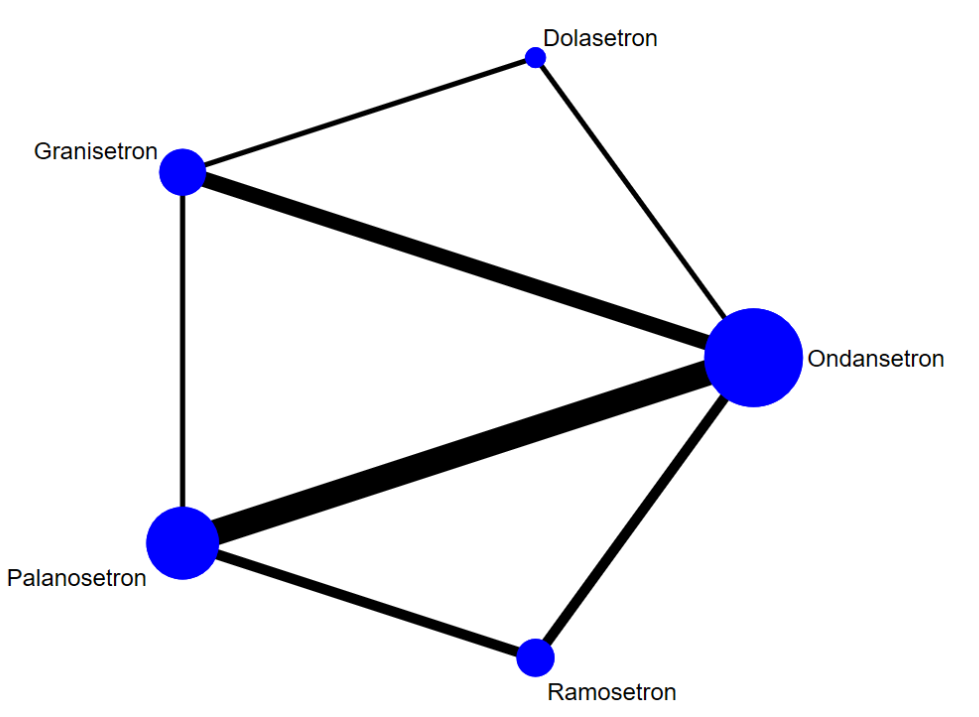


**Network graph of “****Late PONV”**


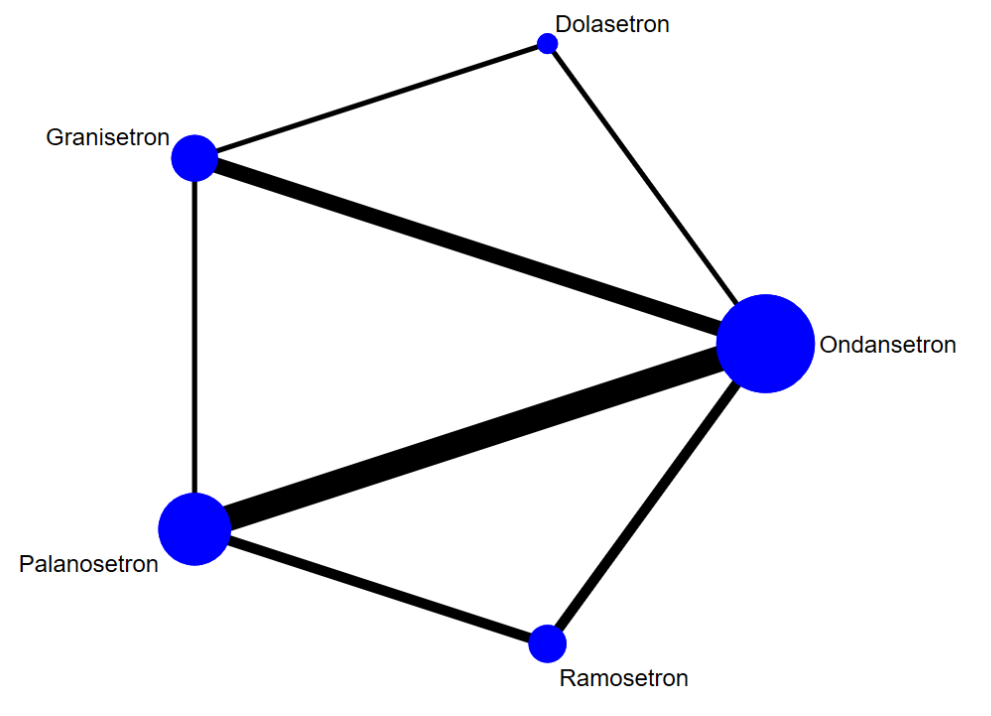


**Network graph of “>24h PONV”**


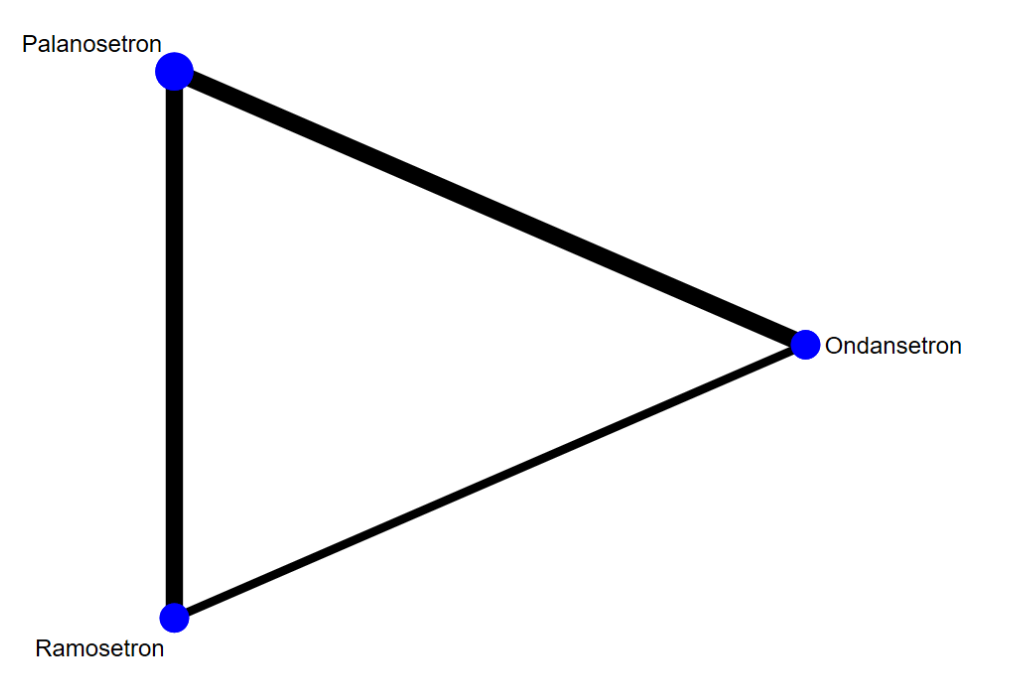


**Network graph of “Overall PONV”**


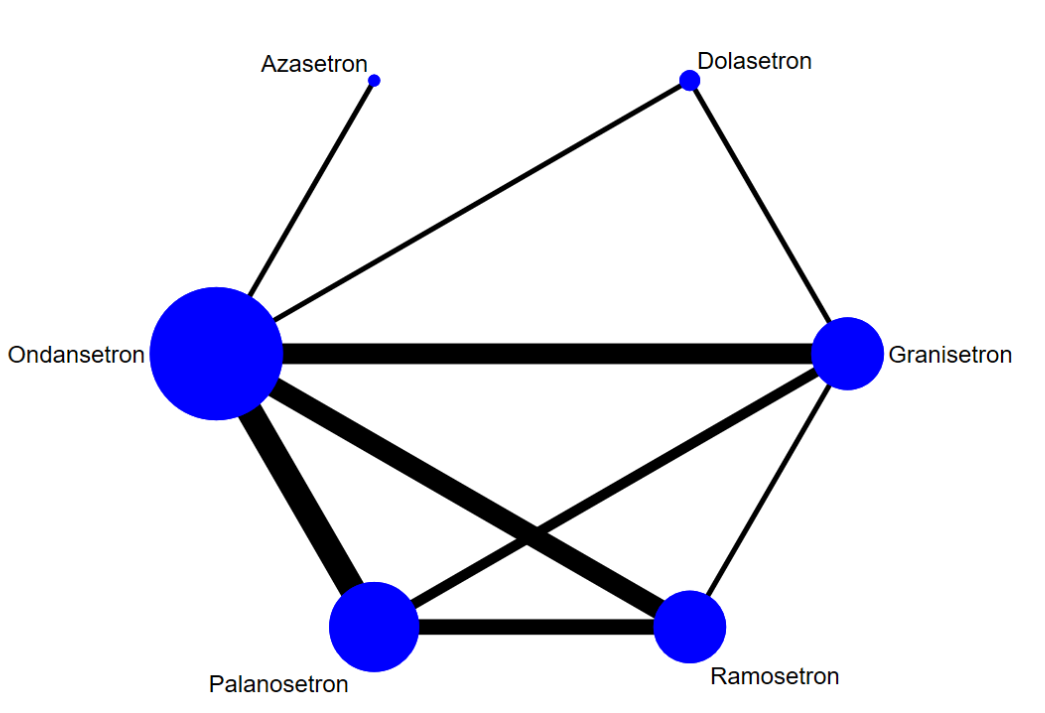


**Network graph of “Acute rescue medicine”**


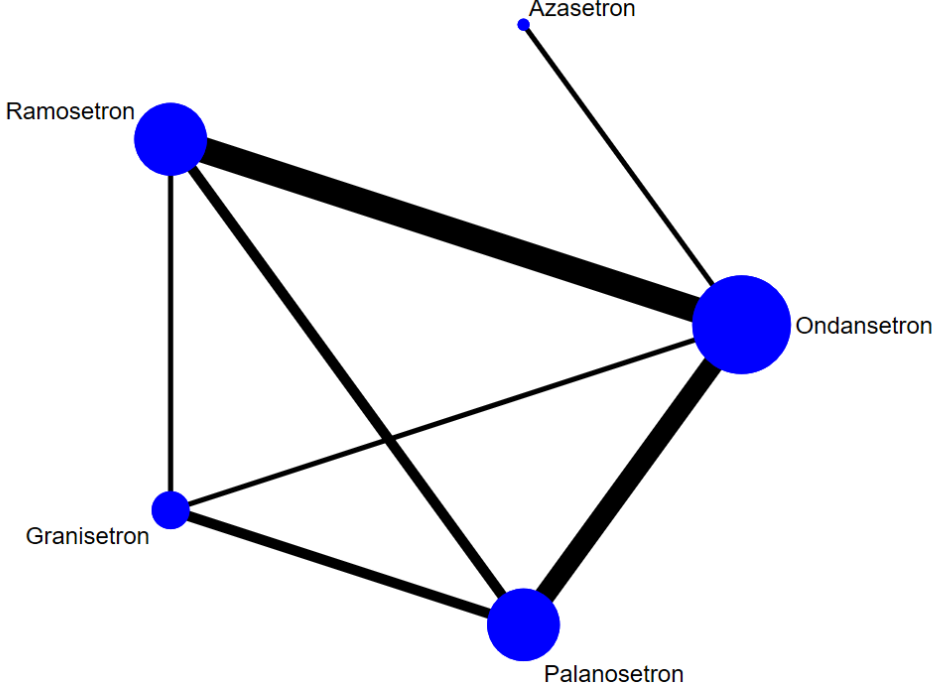


**Network graph of “Late rescue medicine”**


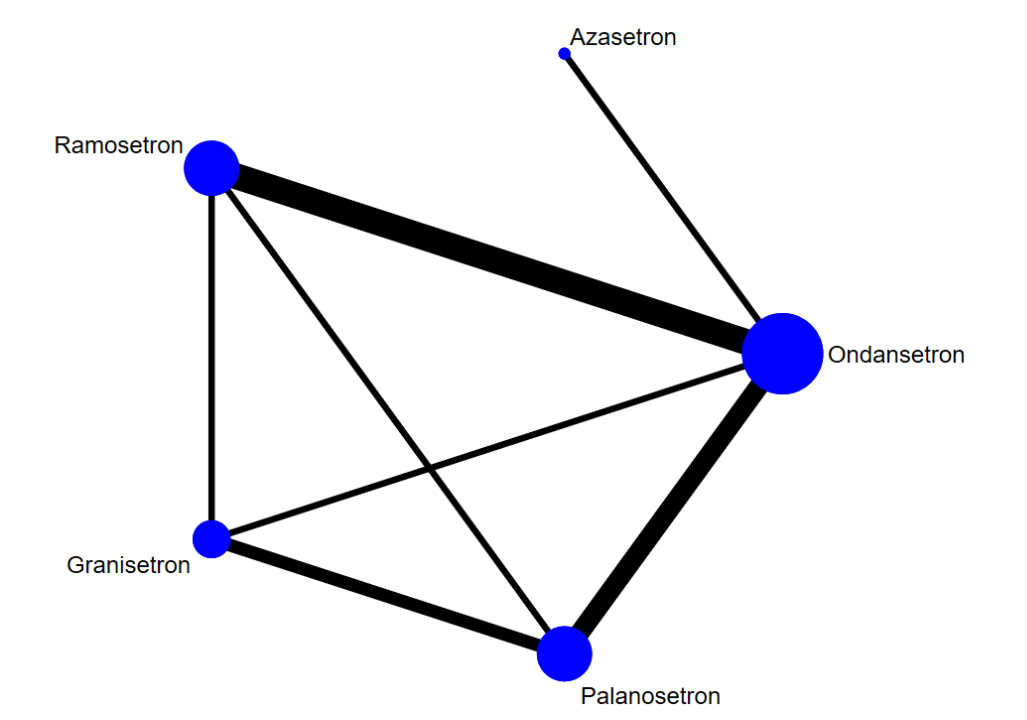


**Network graph of “>24h rescue medicine”**


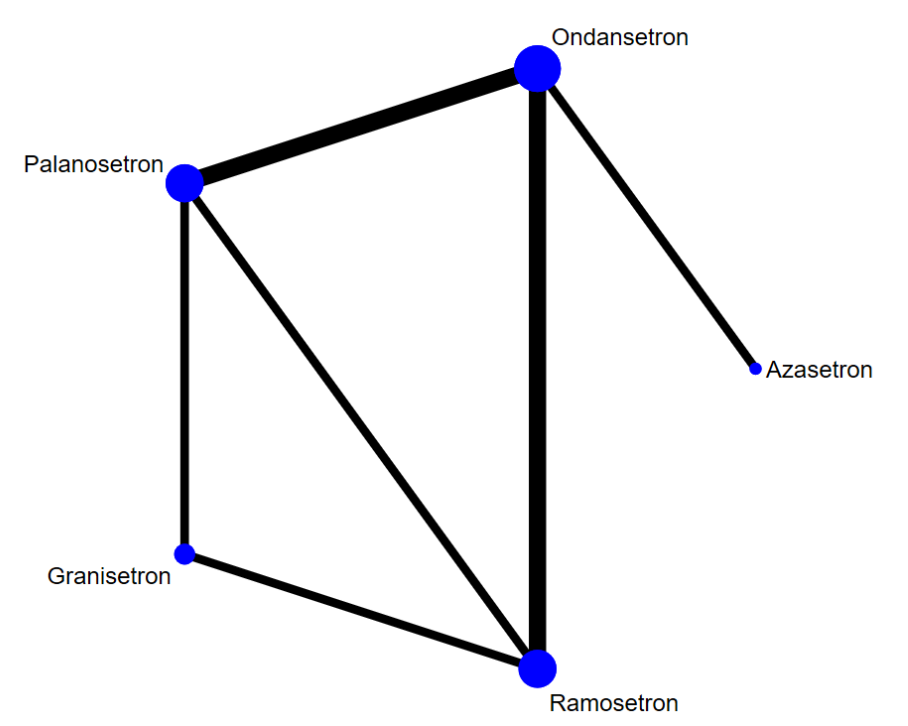


**Network graph of “Overall rescue medicine”**


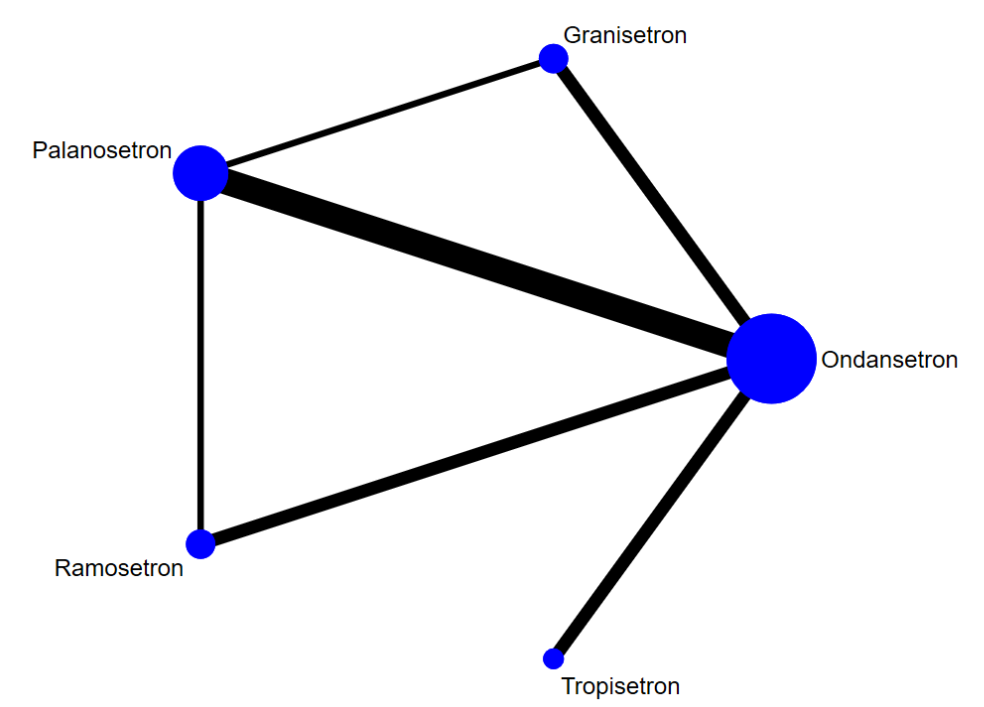


**Network graph of “Adverse reaction”**


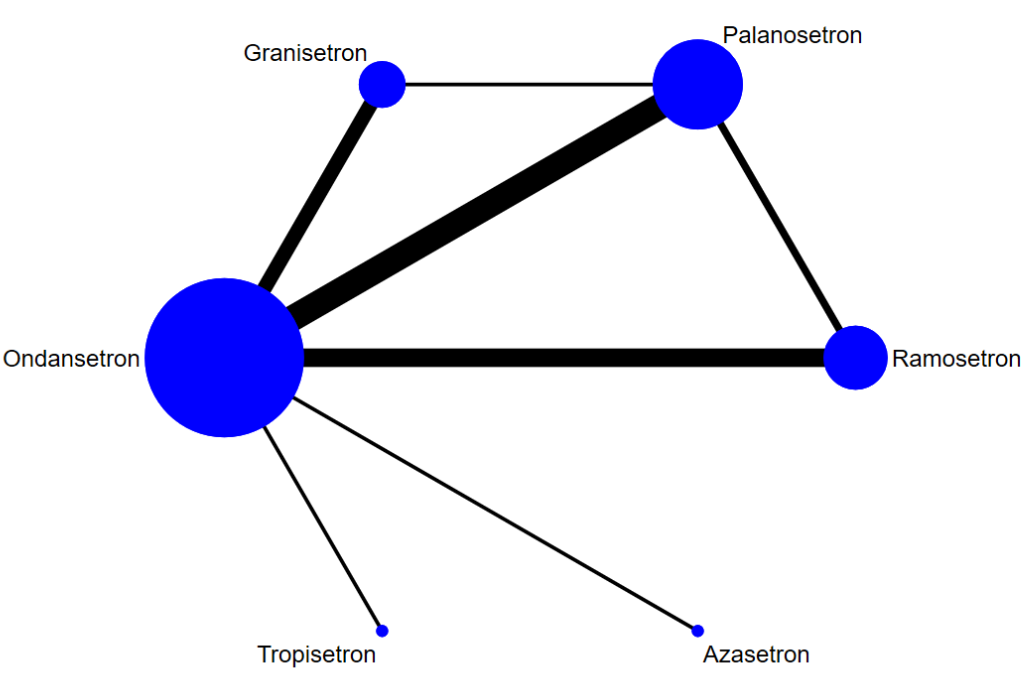

Supplement: Supplementary file 5 — Data S5. [file IJGO-171-177-s004.docx]
