# Supplementary material for: 5‐HT3 receptor antagonists for preventing postoperative nausea and vomiting after gynecological surgery: A systematic review and network meta‐analysis
Source: Int J Gynaecol Obstet. 2025 May 9;171(1):177–89. doi: 10.1002/ijgo.70197 (PMC12447676; doi:10.1002/ijgo.70197)
Supplement: Supplementary file 6 — Data S6. [file IJGO-171-177-s008.docx]

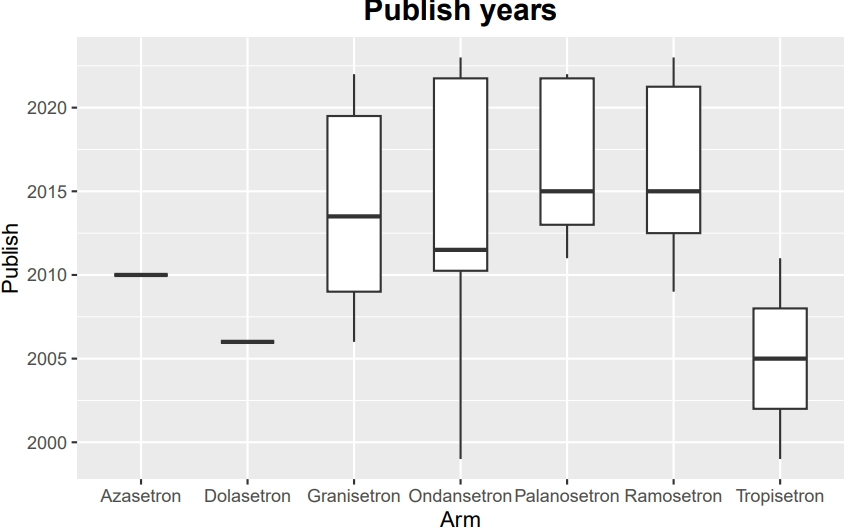


Publication year distribution of the included studies（P=0.227）


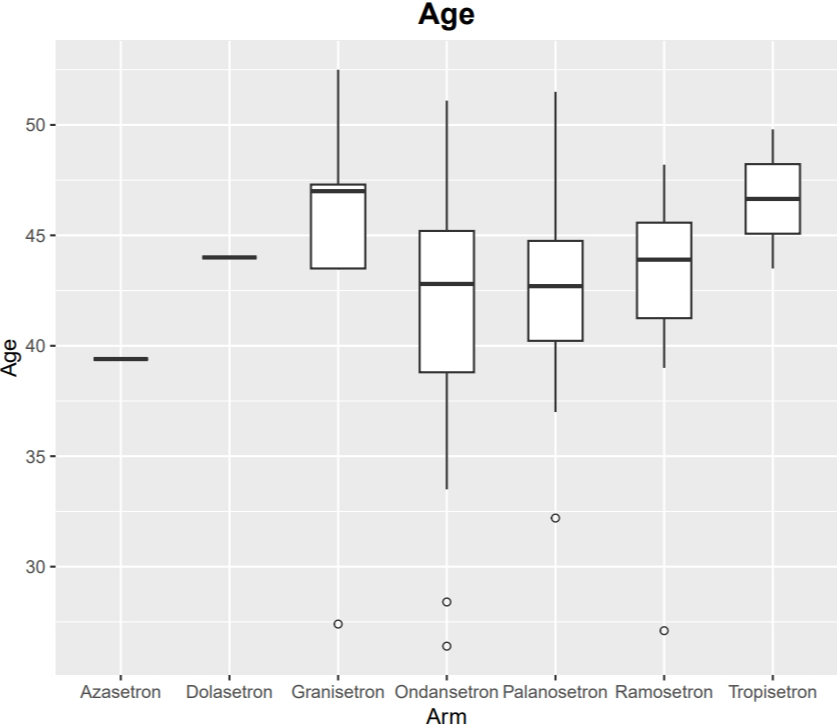


Age distribution of the included studies（P=0.936）


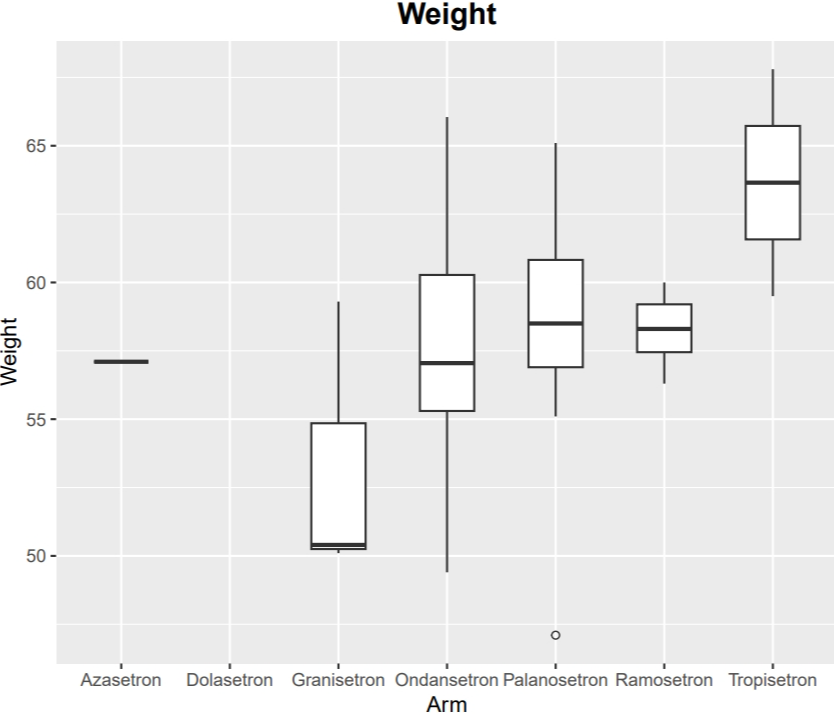


Weight distribution of the included studies（P=0.256）


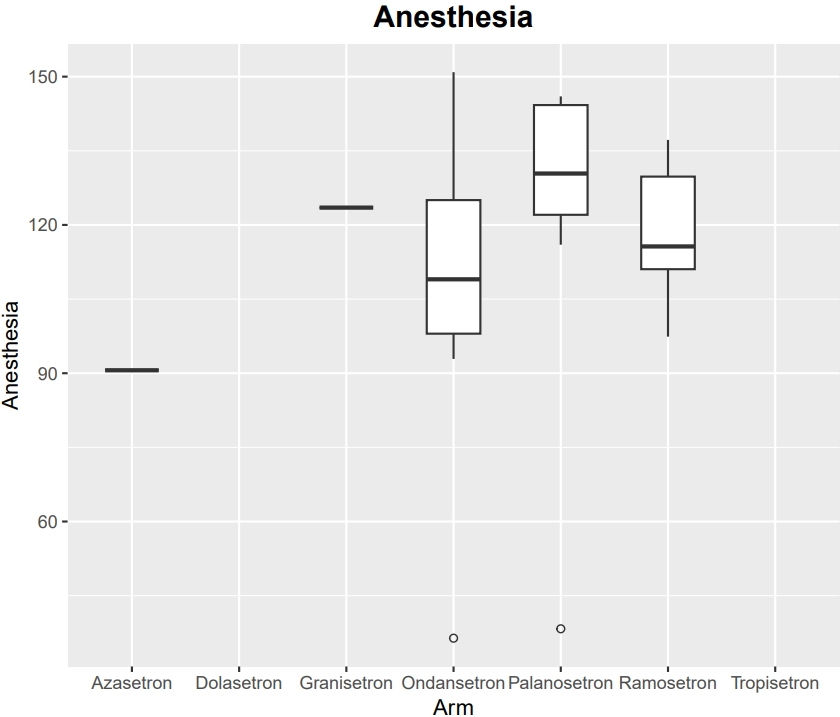


Anesthesia duration time distribution of the included studies（P=0.827）


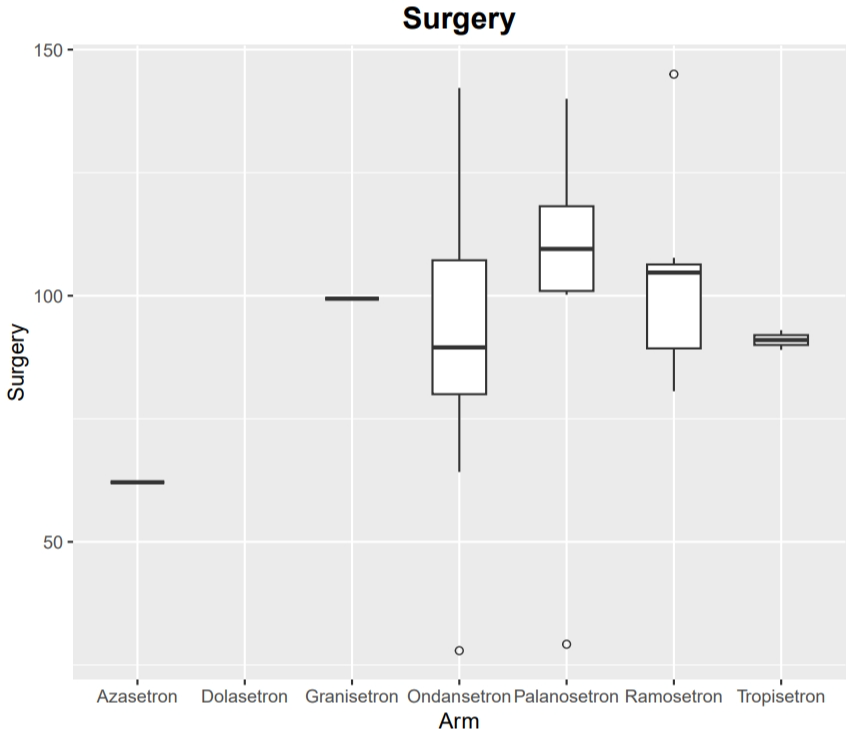


Surgery duration time distribution of the included studies（P=0.974）
