# Supplementary material for: 5‐HT3 receptor antagonists for preventing postoperative nausea and vomiting after gynecological surgery: A systematic review and network meta‐analysis
Source: Int J Gynaecol Obstet. 2025 May 9;171(1):177–89. doi: 10.1002/ijgo.70197 (PMC12447676; doi:10.1002/ijgo.70197)

**Data S7 Sensitivity of exclusing open surgery**

**Network calculation of “Acute nausea”**

| Azasetron | . | 0.82 (0.37; 1.80) | . | . |
| --- | --- | --- | --- | --- |
| 1.47 (0.43; 5.07) | Granisetron | 0.20 (0.01; 4.00) | 0.84 (0.30; 2.41) | 0.83 (0.28;2.48) |
| 0.82 (0.37; 1.80) | 0.56 (0.21; 1.45) | Ondansetron | 1.71 (1.13; 2.59) | 1.07 (0.69;1.68) |
| 1.34 (0.57; 3.16) | 0.91 (0.36; 2.31) | 1.64 (1.17; 2.30) | Palanosetron | 0.73 (0.49; 1.07) |
| 0.94 (0.40; 2.22) | 0.64 (0.25; 1.61) | 1.14 (0.81; 1.62) | 0.70 (0.50;0.97) | Ramosetron |

**P-score of “Acute nausea”**

Palanosetron 0.7889

Granisetron 0.7554

Azasetron 0.4430

Ramosetron 0.3503

Ondansetron 0.1625

**Forest diagram of “Acute nausea”**


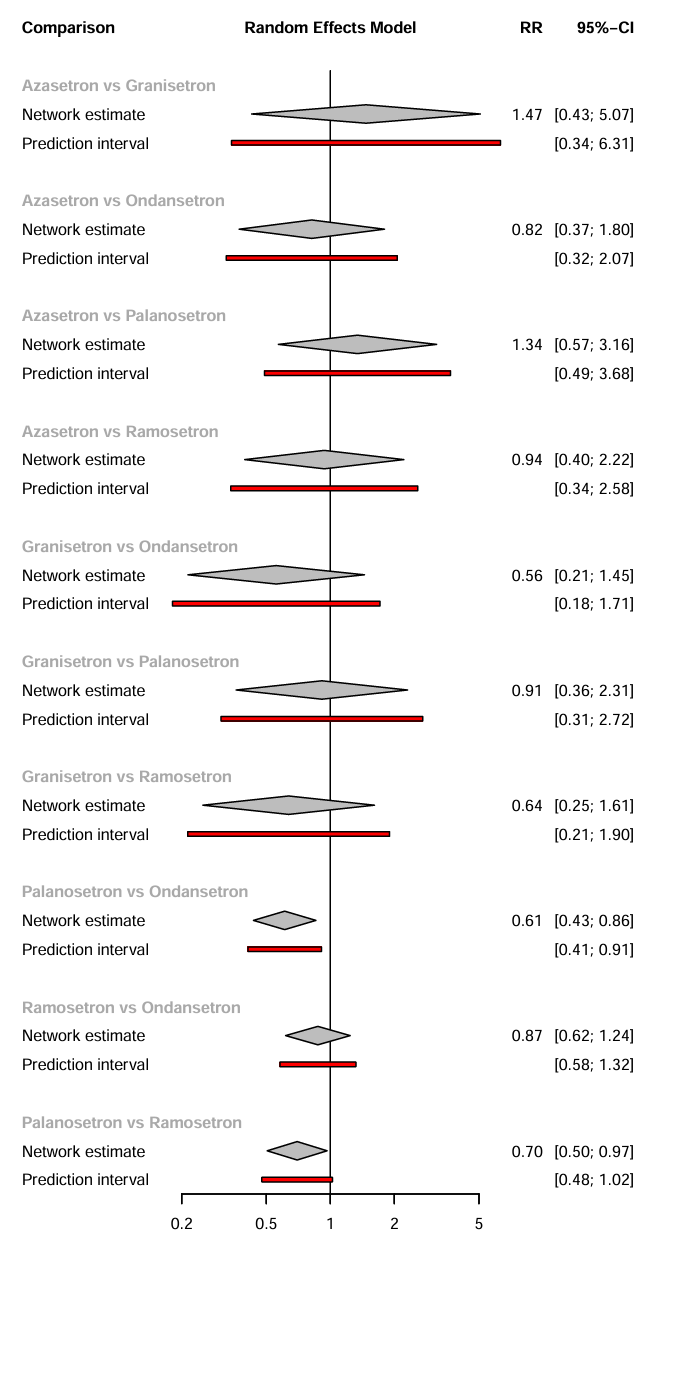


**Network calculation of “Late nausea”**

| Azasetron | . | 0.88 (0.37; 2.07) | . | . |
| --- | --- | --- | --- | --- |
| 1.34 (0.37; 4.83) | Granisetron | 0.38 (0.10; 1.48) | 1.72 (0.48; 6.21) | 1.00 (0.24; 4.23) |
| 0.88 (0.37; 2.07) | 0.65 (0.25; 1.69) | Ondansetron | 1.50 (1.00; 2.26) | 0.87 (0.43; 1.74) |
| 1.21 (0.47; 3.09) | 0.90 (0.34; 2.36) | 1.38 (0.95; 2.01) | Palanosetron | 1.16 (0.57; 2.34) |
| 1.05 (0.39; 2.87) | 0.79 (0.29; 2.11) | 1.20 (0.72; 2.02) | 0.87 (0.51; 1.48) | Ramosetron |

**P-score of “Late nausea”**

Granisetron 0.6871

Palanosetron 0.6809

Ramosetron 0.4798

Azasetron 0.4379

Ondansetron 0.2143

**Forest diagram of “Late nausea”**


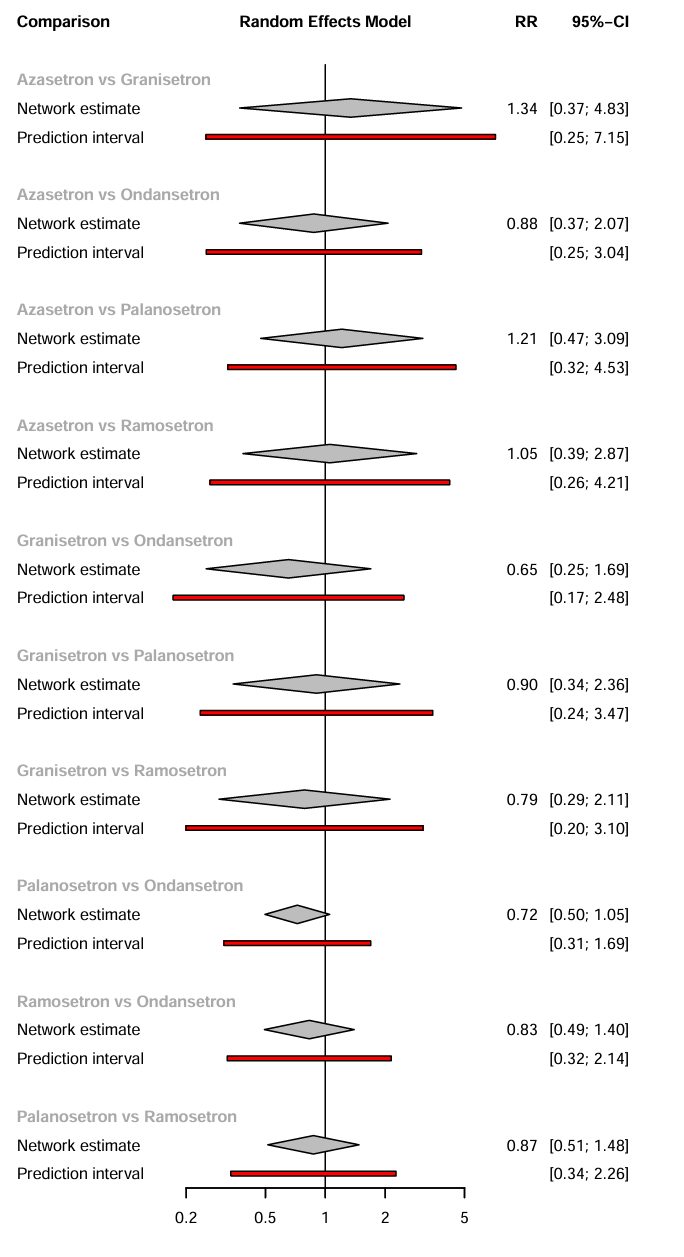


**Network calculation of “>24h nausea”**

| Azasetron | . | 1.50 (0.58; 3.89) | . | . |
| --- | --- | --- | --- | --- |
| 3.54 (0.30; 41.91) | Granisetron | . | 0.50 (0.05; 5.27) | 1.00 (0.07; 15.36) |
| 1.50 (0.58; 3.89) | 0.42 (0.04; 4.15) | Ondansetron | 1.30 (0.80; 2.10) | 5.00 (0.61; 41.28) |
| 2.06 (0.71; 5.97) | 0.58 (0.06; 5.44) | 1.37 (0.85; 2.20) | Palanosetron | 1.14 (0.59; 2.18) |
| 2.59 (0.76; 8.75) | 0.73 (0.08; 7.04) | 1.72 (0.81; 3.68) | 1.26 (0.67; 2.35) | Ramosetron |

**P-score of “>24h nausea”**

Ramosetron 0.7536

Granisetron 0.7250

Palanosetron 0.5915

Ondansetron 0.3008

Azasetron 0.1291

**Forest diagram of “>24h nausea”**


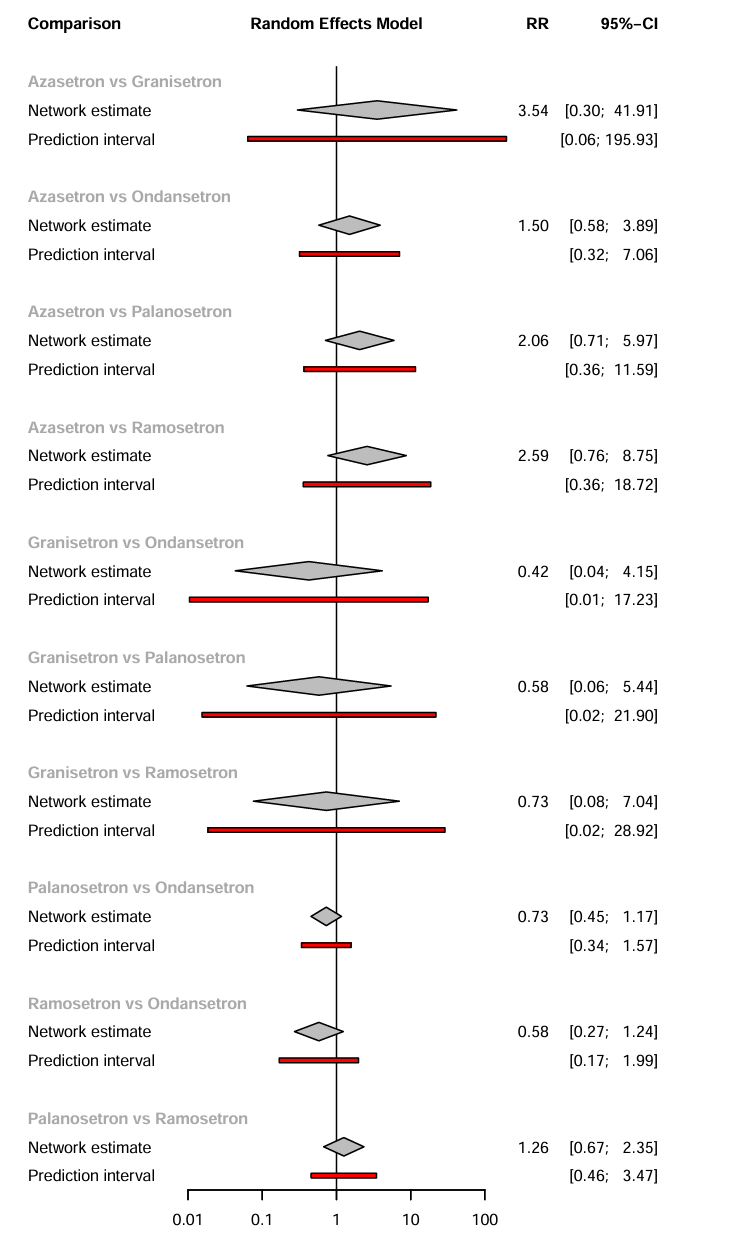


**Network calculation of “Overall nausea”**

| Azasetron | . | 0.84 (0.45; 1.57) | . | . |
| --- | --- | --- | --- | --- |
| 2.43 (0.76; 7.81) | Granisetron | 0.31 (0.12; 0.84) | 3.00 (0.32; 28.55) | . |
| 0.84 (0.45; 1.57) | 0.35 (0.13; 0.93) | Ondansetron | 1.61 (1.13; 2.30) | 1.05 (0.67; 1.66) |
| 1.25 (0.62; 2.52) | 0.51 (0.18; 1.44) | 1.49 (1.08; 2.04) | Palanosetron | 0.97 (0.57; 1.65) |
| 1.01 (0.49; 2.10) | 0.42 (0.15; 1.19) | 1.21 (0.83; 1.75) | 0.81 (0.55; 1.20) | Ramosetron |

**P-score of “Overall nausea”**

Granisetron 0.9399

Palanosetron 0.6707

Ramosetron 0.3875

Azasetron 0.3822

Ondansetron 0.1197

**Forest diagram of “Overall nausea”**


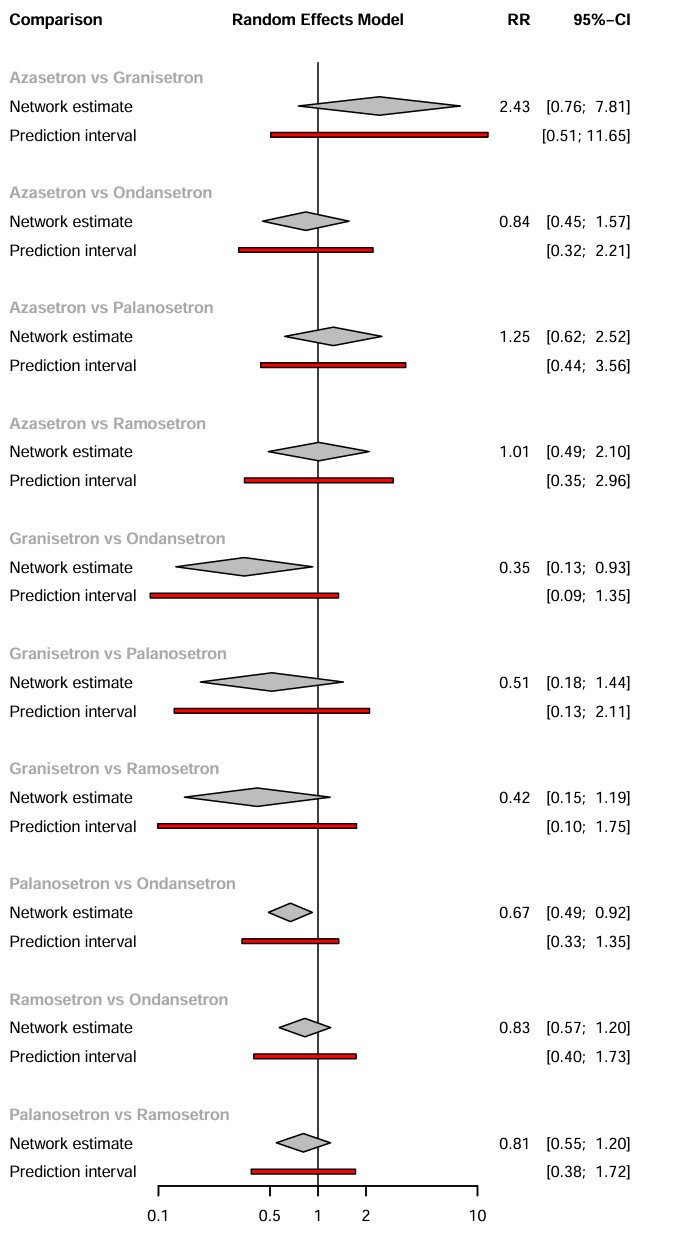


**Network calculation of “Acute vomiting”**

| Azasetron | . | 0.20 (0.01; 4.40) | . | . |
| --- | --- | --- | --- | --- |
| 0.72 (0.02; 32.42) | Granisetron | 0.33 (0.01; 8.50) | 0.52 (0.04; 6.36) | 0.33 (0.01; 8.54) |
| 0.20 (0.01; 4.40) | 0.28 (0.03; 2.54) | Ondansetron | 1.29 (0.48; 3.48) | 1.71 (0.76; 3.84) |
| 0.40 (0.02; 9.71) | 0.55 (0.06; 5.10) | 1.99 (0.88; 4.48) | Palanosetron | 0.34 (0.10; 1.11) |
| 0.25 (0.01; 6.02) | 0.35 (0.04; 3.26) | 1.26 (0.62; 2.58) | 0.63 (0.27; 1.52) | Ramosetron |

**P-score of “Acute vomiting”**

Azasetron 0.7324

Granisetron 0.7071

Palanosetron 0.5957

Ramosetron 0.3166

Ondansetron 0.1482

**Forest diagram of “Acute vomiting”**


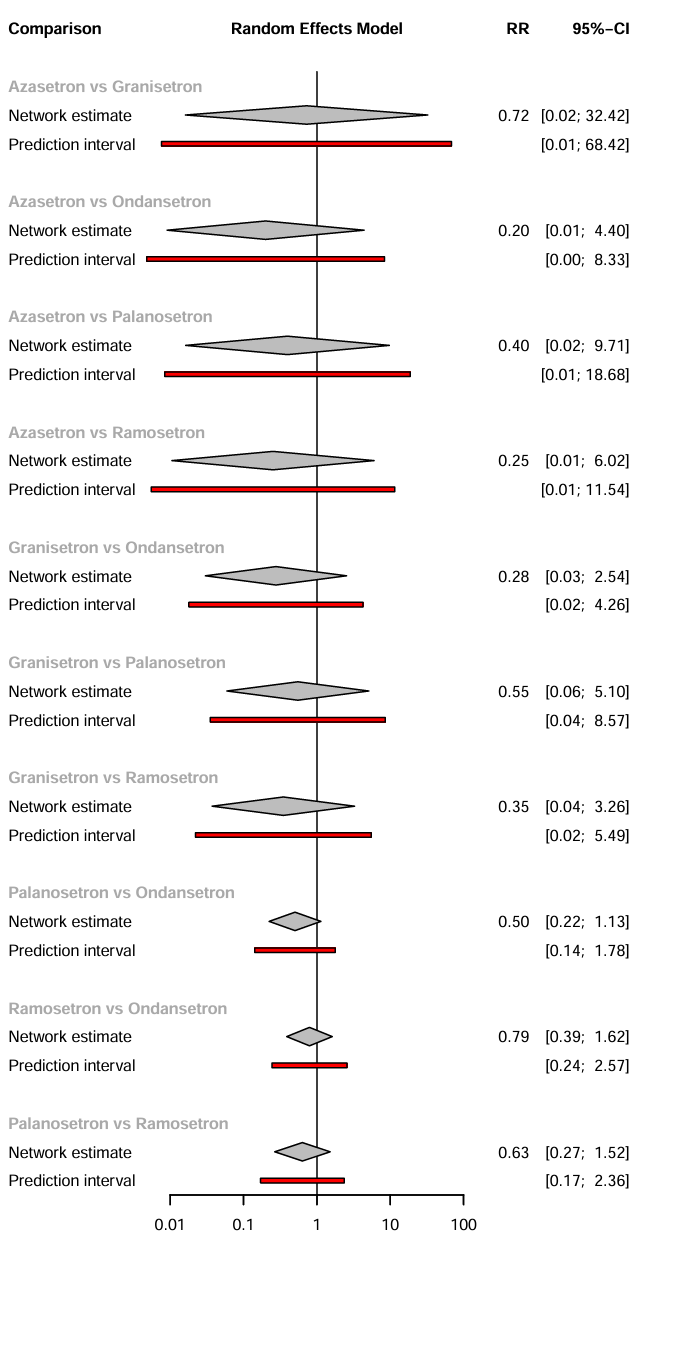


**Network calculation of “Late vomiting”**

| Azasetron | . | 2.00 (0.19; 21.34) | . | . |
| --- | --- | --- | --- | --- |
| 7.21 (0.48; 108.30) | Granisetron | 0.25 (0.06; 1.08) | 1.06 (0.16; 6.98) | 1.00 (0.02; 49.04) |
| 2.00 (0.19; 21.34) | 0.28 (0.07; 1.04) | Ondansetron | 2.01 (1.22; 3.31) | 1.15 (0.58; 2.30) |
| 3.78 (0.34; 42.19) | 0.52 (0.13; 2.05) | 1.89 (1.19; 3.01) | Palanosetron | 0.96 (0.30; 3.07) |
| 2.59 (0.22; 29.84) | 0.36 (0.09; 1.50) | 1.30 (0.71; 2.37) | 0.69 (0.34; 1.36) | Ramosetron |

**P-score of “Late vomiting”**

Granisetron 0.9094

Palanosetron 0.7229

Ramosetron 0.4496

Ondansetron 0.2375

Azasetron 0.1806

**Forest diagram of “Late vomiting”**


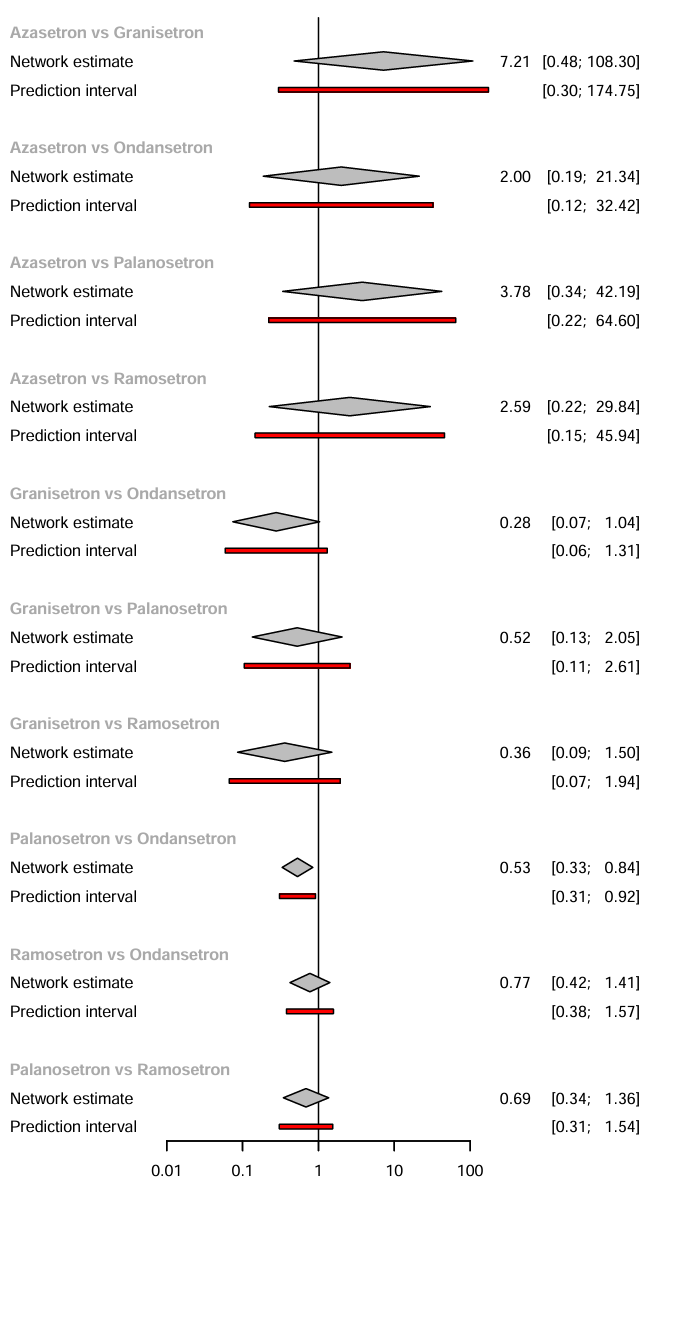


**Network calculation of “>24h vomiting”**

| Azasetron | . | 3.00 (0.13; 71.89) | . | . |
| --- | --- | --- | --- | --- |
| 8.84 (0.08; 1017.00) | Granisetron | . | 1.00 (0.02; 49.04) | 1.00 (0.02; 49.04) |
| 3.00 (0.13;71.89) | 0.34 (0.01; 11.53) | Ondansetron | 2.83 (0.42; 19.02) | 2.50 (0.84; 7.45) |
| 11.52 (0.34;393.31) | 1.30 (0.04; 41.72) | 3.84 (0.82;17.93) | Palanosetron | 0.37 (0.03; 3.96) |
| 6.78 (0.24; 191.18) | 0.77 (0.02; 24.58) | 2.26 (0.81; 6.32) | 0.59 (0.12; 2.96) | Ramosetron |

**P-score of “>24h vomiting”**

Palanosetron 0.7920

Granisetron 0.6355

Ramosetron 0.6276

Ondansetron 0.2821

Azasetron 0.1627

**Forest diagram of “>24h vomiting”**


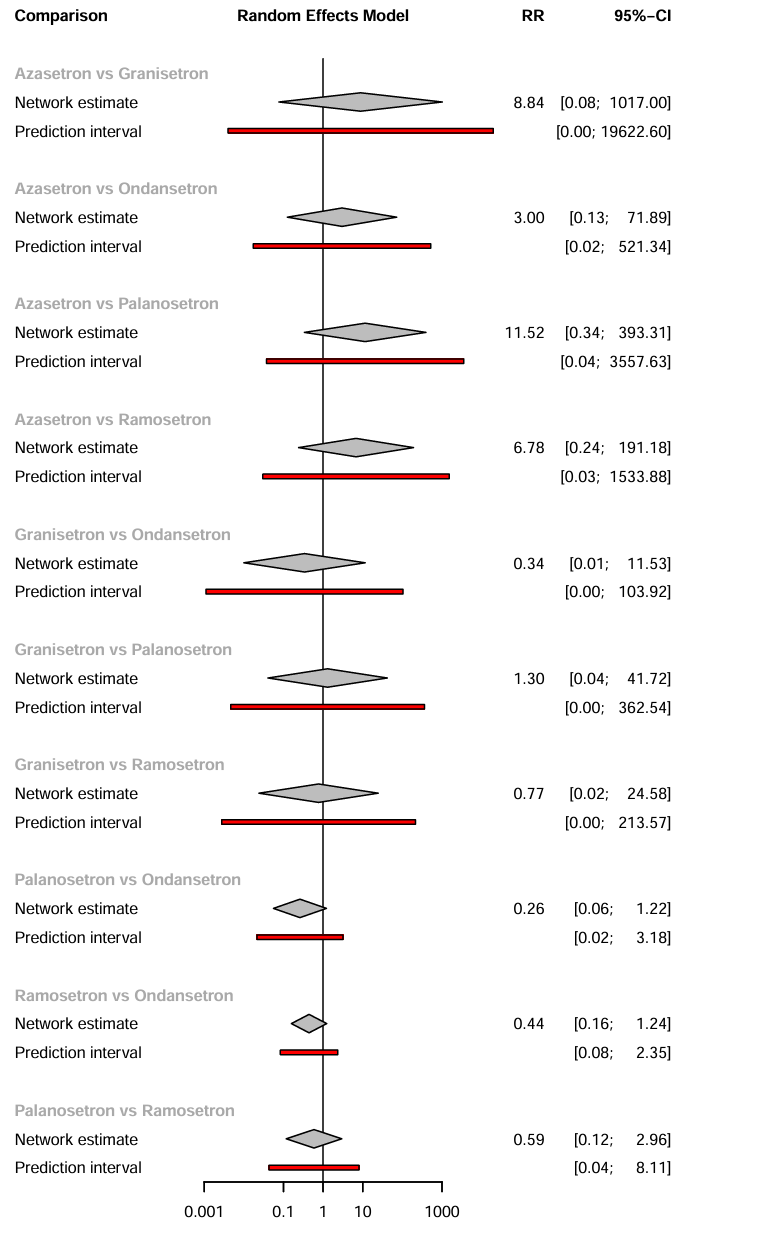


**Network calculation of “Overall vomiting”**

| Azasetron | . | 0.62 (0.16;2.40) | . | . |
| --- | --- | --- | --- | --- |
| 1.55 (0.25; 9.71) | Granisetron | 0.36 (0.10; 1.26) | 2.00 (0.16; 24.25) | . |
| 0.62 (0.16; 2.40) | 0.40 (0.12; 1.41) | Ondansetron | 1.71 (0.84; 3.45) | 0.89 (0.39; 2.07) |
| 1.11 (0.25; 4.91) | 0.72 (0.18; 2.84) | 1.77 (0.94; 3.34) | Palanosetron | 0.41 (0.13; 1.31) |
| 0.52 (0.11; 2.39) | 0.34 (0.08; 1.41) | 0.83 (0.41; 1.70) | 0.47 (0.21; 1.04) | Ramosetron |

**P-score of “Overall vomiting”**

Granisetron 0.8038

Palanosetron 0.7008

Azasetron 0.5797

Ondansetron 0.2634

Ramosetron 0.1522

**Forest diagram of “Overall vomiting”**


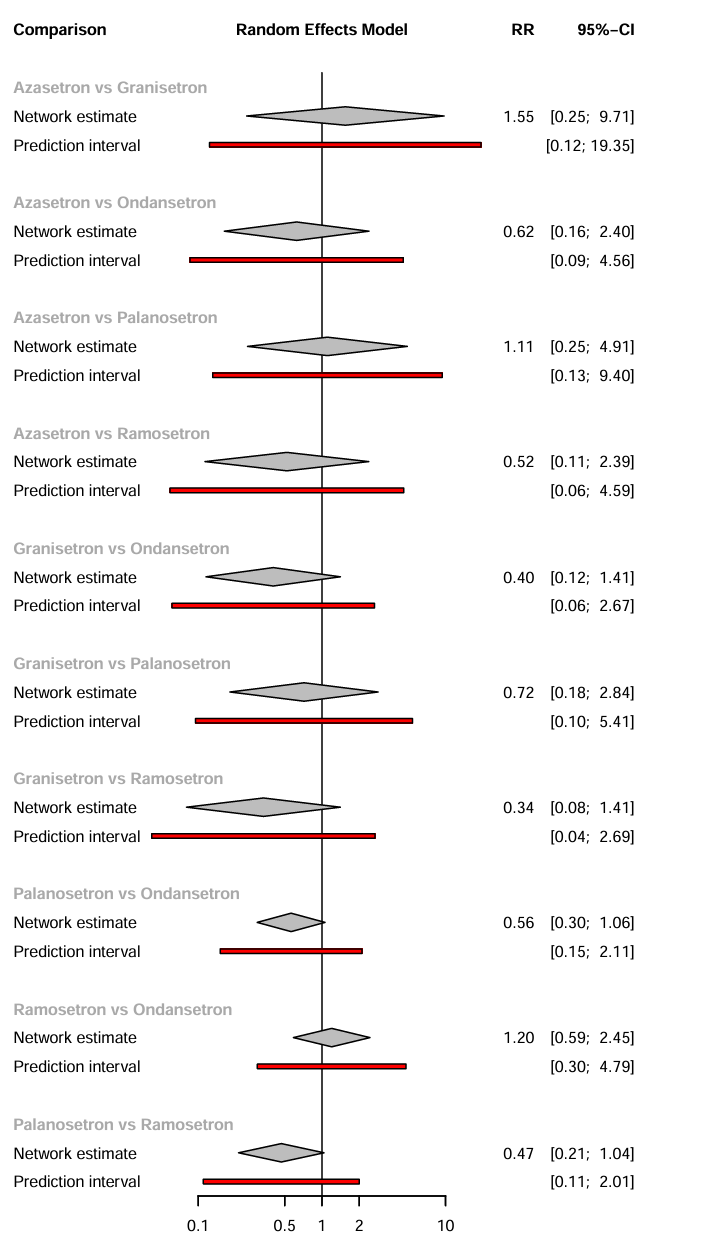


**Network calculation of “Acute PONV”**

| Granisetron | 0.80 (0.32; 1.97) | 1.00 (0.02; 51.57) | . |
| --- | --- | --- | --- |
| 0.81 (0.33; 2.01) | Ondansetron | 1.47 (0.89; 2.44) | 0.96 (0.45; 2.05) |
| 1.08 (0.39; 2.95) | 1.33 (0.85; 2.08) | Palanosetron | 1.04 (0.56; 1.93) |
| 0.97 (0.34; 2.79) | 1.20 (0.69; 2.07) | 0.90 (0.54; 1.50) | Ramosetron |

**P-score of “Acute PONV”**

Palanosetron 0.7035

Granisetron 0.5475

Ramosetron 0.5186

Ondansetron 0.2304

**Forest diagram of “Acute PONV”**


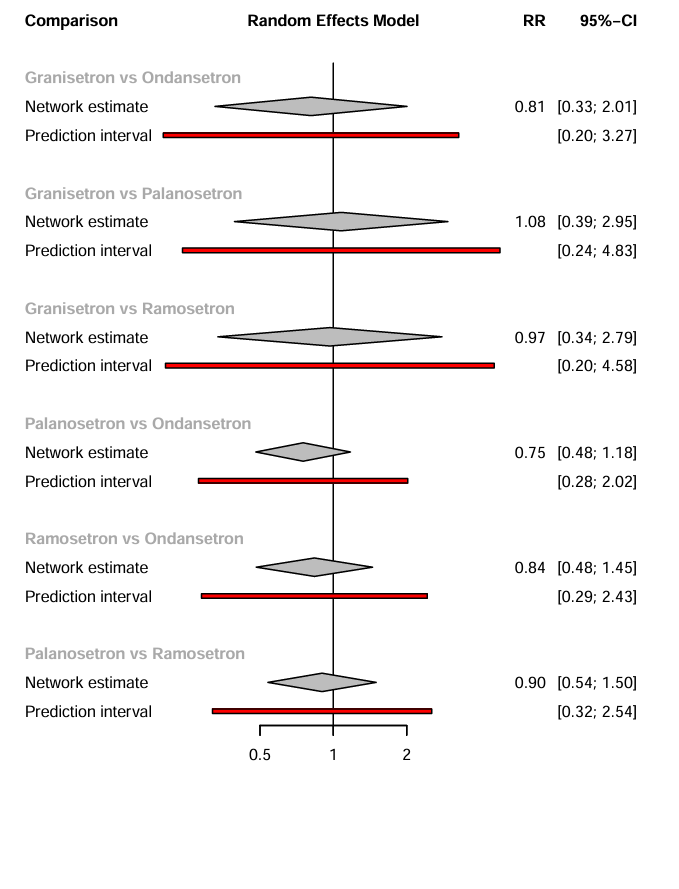


**Network calculation of “Late PONV”**

| Granisetron | 0.62 (0.21; 1.83) | 2.50 (0.50; 12.50) | . |
| --- | --- | --- | --- |
| 0.76 (0.27; 2.14) | Ondansetron | 1.44 (1.06; 1.96) | 1.17 (0.39; 3.48) |
| 1.08 (0.37; 3.12) | 1.42 (1.05; 1.92) | Palanosetron | 0.97 (0.64; 1.47) |
| 1.03 (0.33; 3.16) | 1.35 (0.85; 2.16) | 0.95 (0.65; 1.40) | Ramosetron |

**P-score of “Late PONV”**

Palanosetron 0.7150

Ramosetron 0.6052

Granisetron 0.5419

Ondansetron 0.1379

**Forest diagram of “Late PONV”**


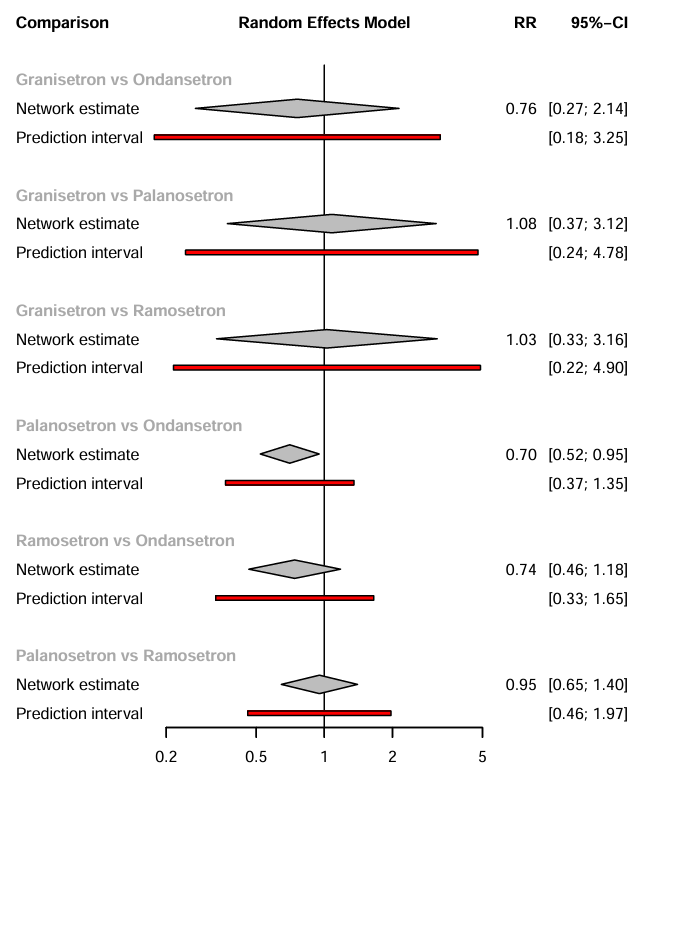


Network calculation of “>24h PONV”

| Ondansetron | 1.32 (0.81; 2.17) | . |
| --- | --- | --- |
| 1.32 (0.81; 2.17) | Palanosetron | 0.96 (0.64; 1.43) |
| 1.27 (0.67; 2.40) | 0.96 (0.64; 1.43) | Ramosetron |

P-score of “>24h PONV”

Palanosetron 0.7247

Ramosetron 0.5910

Ondansetron 0.1843

Forest diagram of “>24h PONV”


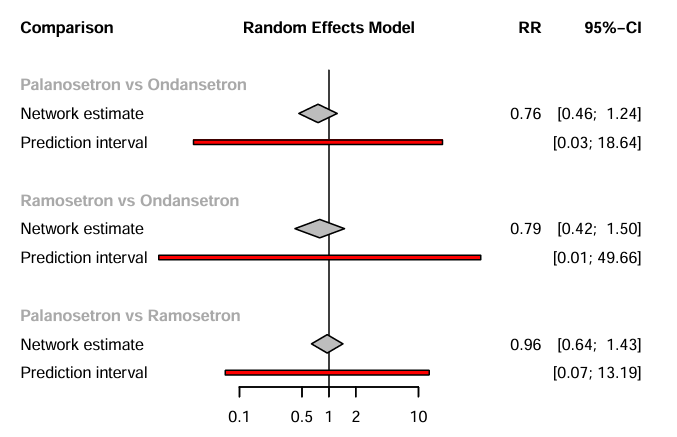


**Network calculation of “Overall PONV”**

| Azasetron | . | 0.75 (0.44; 1.27) | . | . |
| --- | --- | --- | --- | --- |
| 1.14 (0.53; 2.45) | Granisetron | 0.50 (0.21; 1.20) | 0.97 (0.49; 1.90) | 1.22 (0.53; 2.84) |
| 0.75 (0.44; 1.27) | 0.66 (0.38; 1.15) | Ondansetron | 1.47 (1.07; 2.02) | 1.05 (0.73; 1.52) |
| 1.00 (0.55; 1.82) | 0.88 (0.51; 1.54) | 1.34 (1.02; 1.75) | Palanosetron | 1.10 (0.73; 1.65) |
| 0.92 (0.50; 1.69) | 0.81 (0.46; 1.44) | 1.23 (0.91; 1.65) | 0.92 (0.67; 1.25) | Ramosetron |

**P-score of “Overall PONV”**

Granisetron 0.7467

Palanosetron 0.6299

Azasetron 0.5831

Ramosetron 0.4603

Ondansetron 0.0799

**Forest diagram of “Overall PONV”**


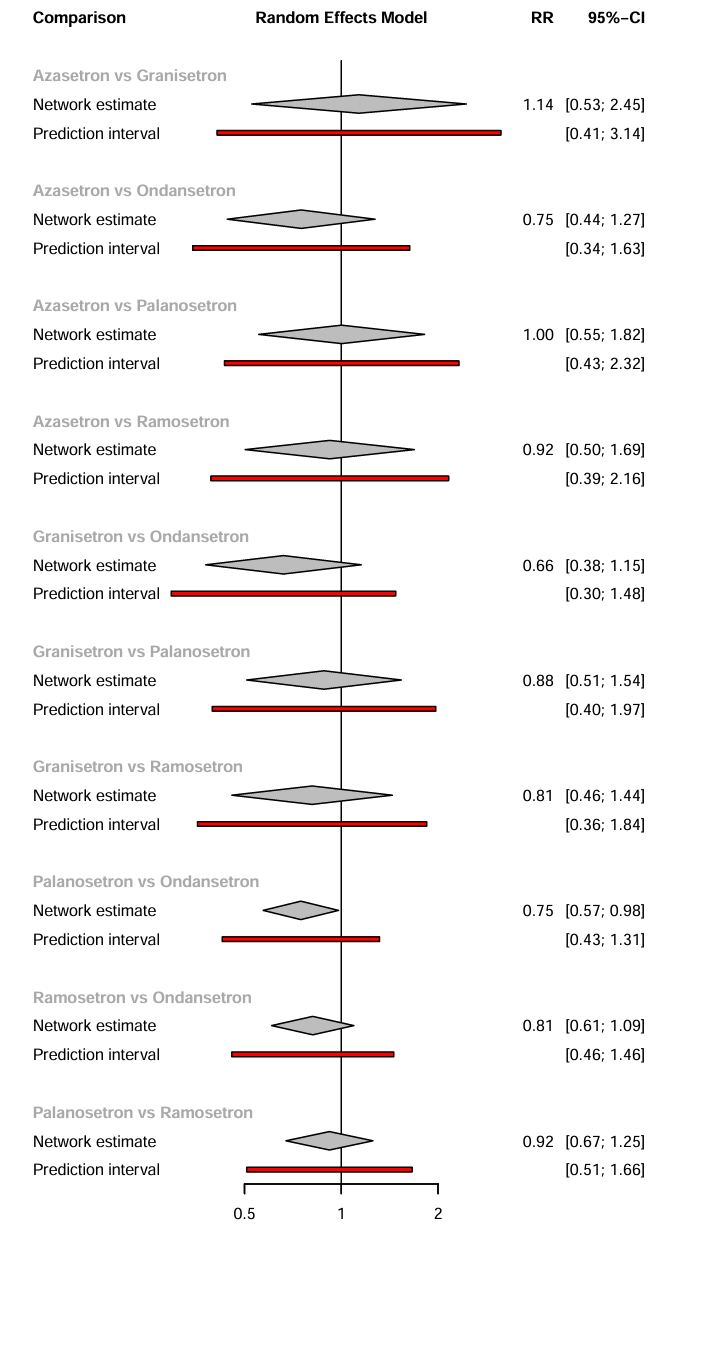


**Network calculation of “Acute rescue medicine”**

| Azasetron | . | 1.00 (0.31; 3.24) | . | . |
| --- | --- | --- | --- | --- |
| 2.00 (0.23; 17.59) | Granisetron | 0.33 (0.01;7.87) | 0.60 (0.08; 4.51) | 1.00 (0.07; 15.36) |
| 1.00 (0.31; 3.24) | 0.50 (0.08; 3.13) | Ondansetron | 0.98 (0.56;1.73) | 1.66 (0.78; 3.52) |
| 0.98 (0.27; 3.57) | 0.49 (0.08; 3.03) | 0.98 (0.56; 1.70) | Palanosetron | 2.00 (0.19; 21.06) |
| 1.69 (0.43; 6.68) | 0.85 (0.13; 5.67) | 1.69 (0.82; 3.46) | 1.73 (0.72; 4.15) | Ramosetron |

**P-score of “Acute rescue medicine”**

Ramosetron 0.7544

Granisetron 0.7127

Azasetron 0.3775

Ondansetron 0.3353

Palanosetron 0.3200

**Forest diagram of “Acute rescue medicine”**


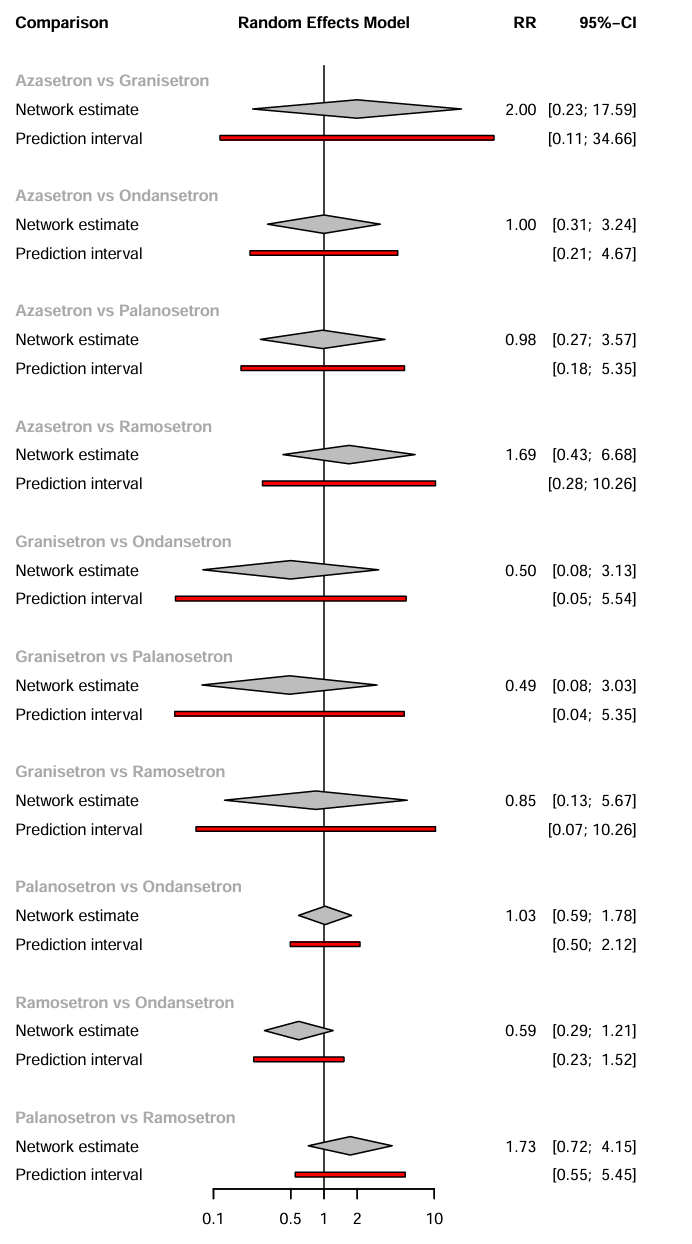


**Network calculation of “Late rescue medicine”**

| Azasetron | . | 1.00 (0.31; 3.24) | . | . |
| --- | --- | --- | --- | --- |
| 3.63 (0.62; 21.21) | Granisetron | 0.25 (0.06; 1.08) | 1.06 (0.16; 6.98) | 1.00 (0.02; 49.04) |
| 1.00 (0.31; 3.24) | 0.28 (0.07; 1.03) | Ondansetron | 1.81 (1.24; 2.66) | 1.85 (0.75; 4.55) |
| 1.79 (0.52; 6.14) | 0.49 (0.13; 1.89) | 1.79 (1.22;2.61) | Palanosetron | 3.00 (0.13; 71.22) |
| 2.00 (0.46; 8.59) | 0.55 (0.12; 2.60) | 2.00 (0.84; 4.75) | 1.12 (0.44; 2.85) | Ramosetron |

**P-score of “Late rescue medicine”**

Granisetron 0.8800

Ramosetron 0.6456

Palanosetron 0.5948

Azasetron 0.2328

Ondansetron 0.1469

**Forest diagram of “Late rescue medicine”**


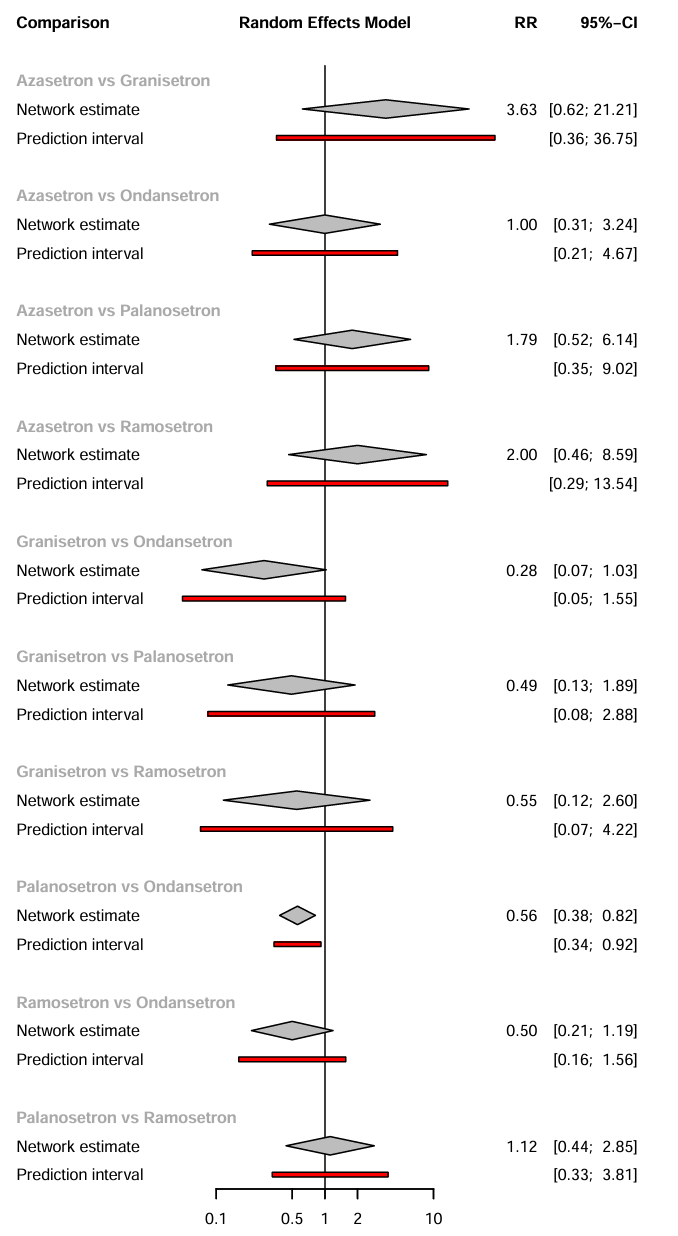


**Network calculation of “>24h rescue medicine”**

| Azasetron | . | 3.00 (0.13; 71.89) | . | . |
| --- | --- | --- | --- | --- |
| 8.85 (0.08; 1027.06) | Granisetron | . | 1.00 (0.02; 49.04) | 1.00 (0.02; 49.04) |
| 3.00 (0.13; 71.89) | 0.34 (0.01; 11.66) | Ondansetron | 2.40 (1.03; 5.57) | 4.00 (0.46; 34.54) |
| 7.32 (0.27; 195.14) | 0.83 (0.02; 27.82) | 2.44 (1.07; 5.59) | Palanosetron | 1.00 (0.02; 49.04) |
| 10.69 (0.26; 431.94) | 1.21 (0.04; 40.60) | 3.56 (0.53; 23.72) | 1.46 (0.20; 10.67) | Ramosetron |

**P-score of “>24h rescue medicine”**

Ramosetron 0.7470

Palanosetron 0.6695

Granisetron 0.6353

Ondansetron 0.2844

Azasetron 0.1638

**Forest diagram of “>24h rescue medicine”**


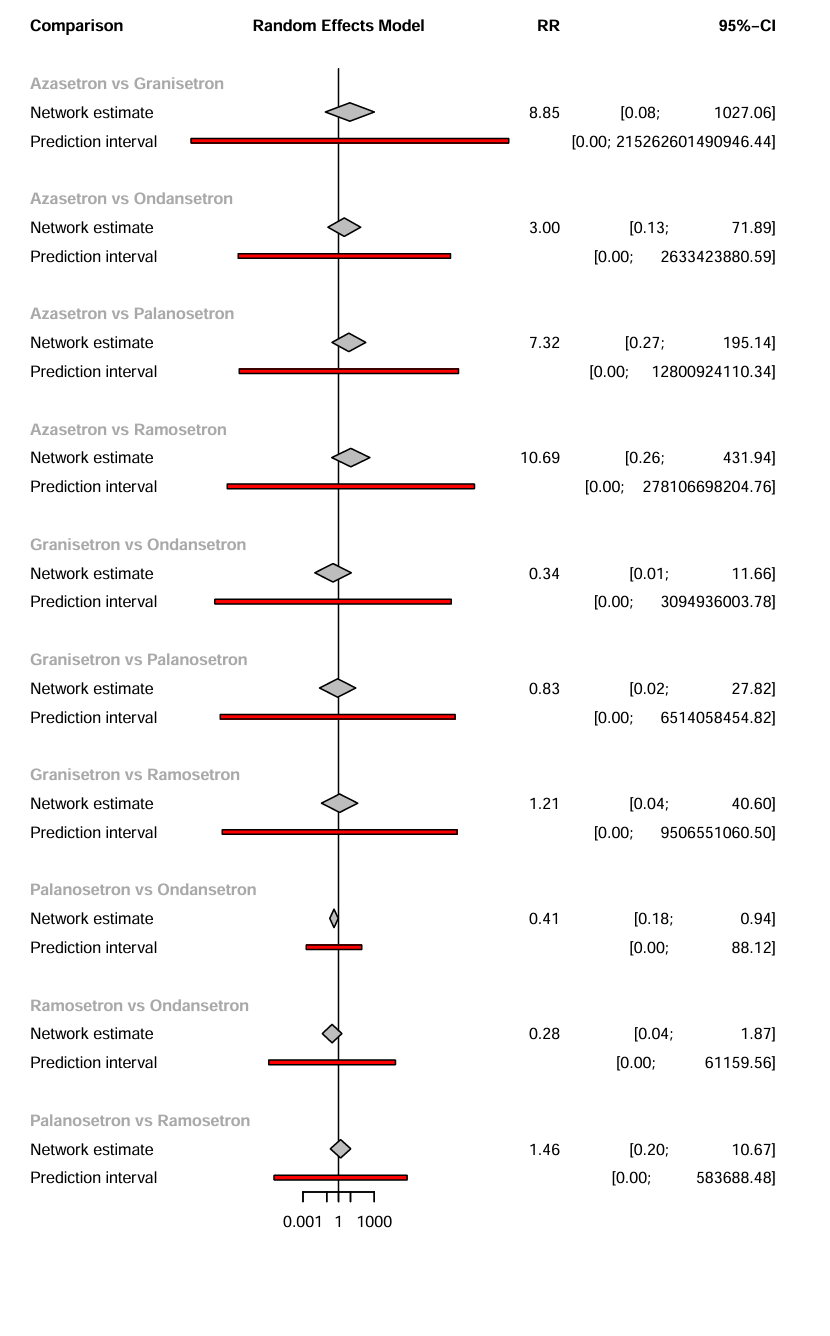


**Network calculation of “Overall rescue medicine”**

| Azasetron | . | 0.62 (0.16; 2.42) | . | . | . |
| --- | --- | --- | --- | --- | --- |
| 1.73 (0.30; 9.87) | Granisetron | 0.33 (0.11; 1.00) | 2.00 (0.16; 24.33) | . | . |
| 0.62 (0.16; 2.42) | 0.36 (0.12; 1.09) | Ondansetron | 1.71 (0.84; 3.47) | 0.89 (0.38; 2.08) | 1.30 (0.58; 2.94) |
| 1.11 (0.25; 4.96) | 0.65 (0.18; 2.25) | 1.78 (0.94; 3.36) | Palanosetron | 0.41 (0.13; 1.32) | . |
| 0.52 (0.11; 2.41) | 0.30 (0.08; 1.12) | 0.83 (0.41; 1.71) | 0.47 (0.21;1.04) | Ramosetron | . |
| 0.81 (0.17; 3.94) | 0.47 (0.12; 1.85) | 1.30 (0.58; 2.94) | 0.73 (0.26; 2.05) | 1.56 (0.53; 4.61) | Tropisetron |

**P-score of “Overall rescue medicine”**

Granisetron 0.8543

Palanosetron 0.6918

Azasetron 0.5730

Tropisetron 0.4675

Ondansetron 0.2547

Ramosetron 0.1587

**Forest diagram of “Overall rescue medicine”**


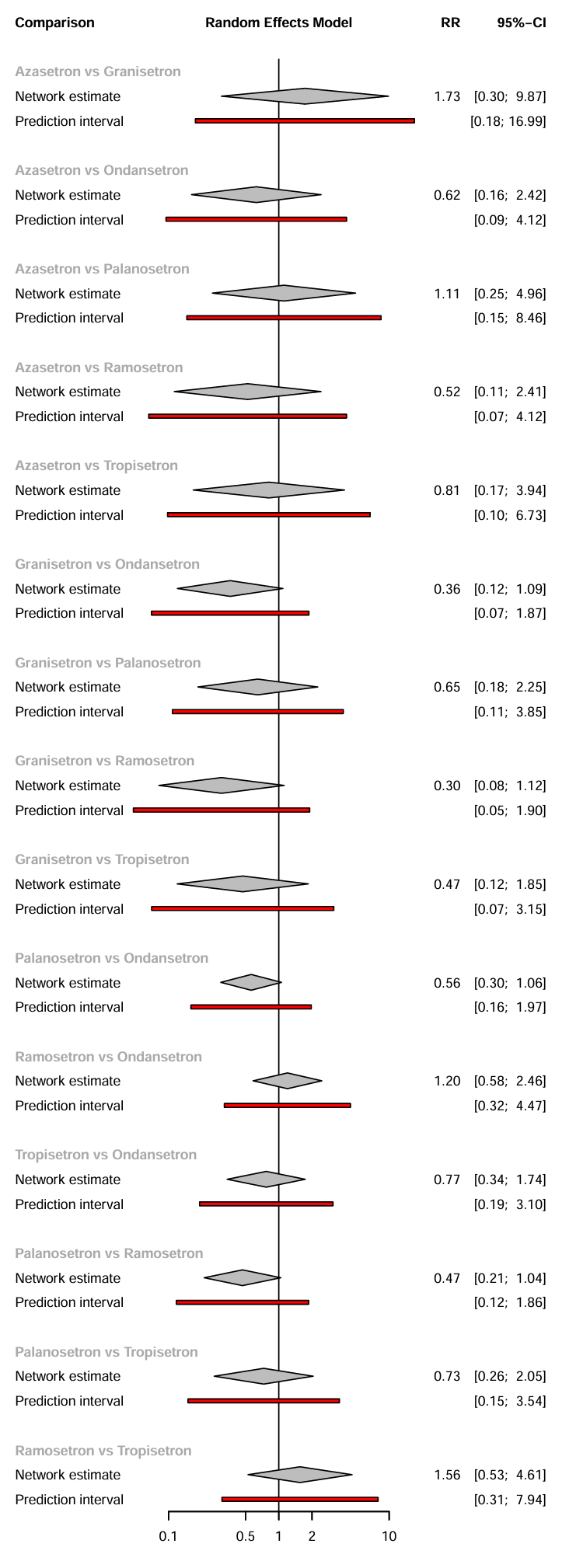


**Network calculation of “Adverse reaction”**

| Azasetron | . | 0.92 (0.37; 2.29) | . | . |
| --- | --- | --- | --- | --- |
| 0.78 (0.21; 2.88) | Granisetron | 1.00 (0.38; 2.62) | 5.00 (0.57; 43.48) | . |
| 0.92 (0.37; 2.29) | 1.17 (0.46; 2.96) | Ondansetron | 1.09 (0.77; 1.53) | 0.98 (0.55; 1.75) |
| 1.02 (0.39; 2.69) | 1.30 (0.50; 3.43) | 1.11 (0.81; 1.53) | Palanosetron | 0.78 (0.37; 1.64) |
| 0.86 (0.31; 2.41) | 1.10 (0.39; 3.10) | 0.94 (0.59; 1.51) | 0.84 (0.51; 1.39) | Ramosetron |

**P-score of “Adverse reaction”**

Palanosetron 0.6799

Azasetron 0.5777

Ondansetron 0.4771

Ramosetron 0.4031

Granisetron 0.3622

**Forest diagram of “Adverse reaction”**


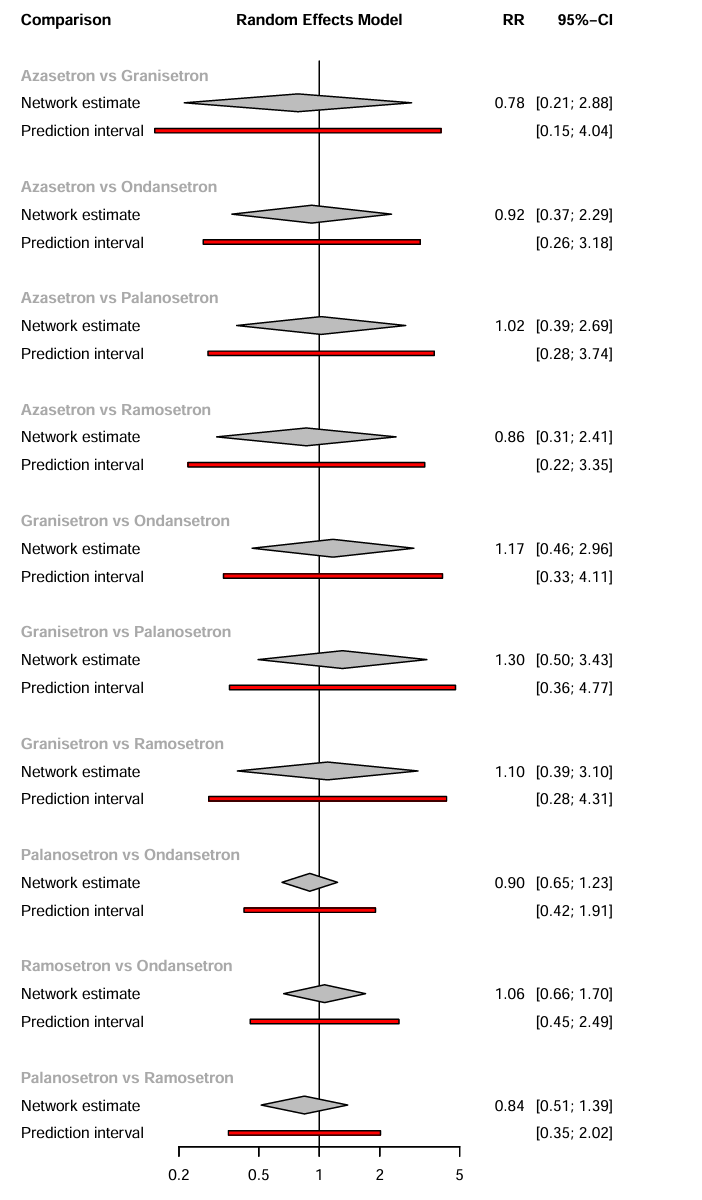

Supplement: Supplementary file 7 — Data S7. [file IJGO-171-177-s007.docx]
