# Supplementary material for: 5‐HT3 receptor antagonists for preventing postoperative nausea and vomiting after gynecological surgery: A systematic review and network meta‐analysis
Source: Int J Gynaecol Obstet. 2025 May 9;171(1):177–89. doi: 10.1002/ijgo.70197 (PMC12447676; doi:10.1002/ijgo.70197)

**Data S8 Sensitivity of exclusing non-opioid analgesia**

**Network calculation of** **“acute nausea”**

| Azasetron | . | 0.82 (0.37; 1.81) | . | . |
| --- | --- | --- | --- | --- |
| 3.38 (0.16; 72.35) | Granisetron | 0.20 (0.01; 4.01) | 1.00 (0.02; 48.89) | . |
| 0.82 (0.37; 1.81) | 0.24 (0.01; 4.67) | Ondansetron | 1.48 (1.12; 1.94) | 1.13 (0.88; 1.46) |
| 1.22 (0.53; 2.79) | 0.36 (0.02; 7.00) | 1.49 (1.16; 1.90) | Palanosetron | 0.81 (0.60; 1.09) |
| 0.92 (0.40; 2.10) | 0.27 (0.01; 5.30) | 1.12 (0.89; 1.42) | 0.76 (0.58; 0.98) | Ramosetron |

**P-score of “acute nausea”**

Granisetron 0.7907

Palanosetron 0.7273

Azasetron 0.4520

Ramosetron 0.3678

Ondansetron 0.1622

**Forest diagram of “acute nausea”**


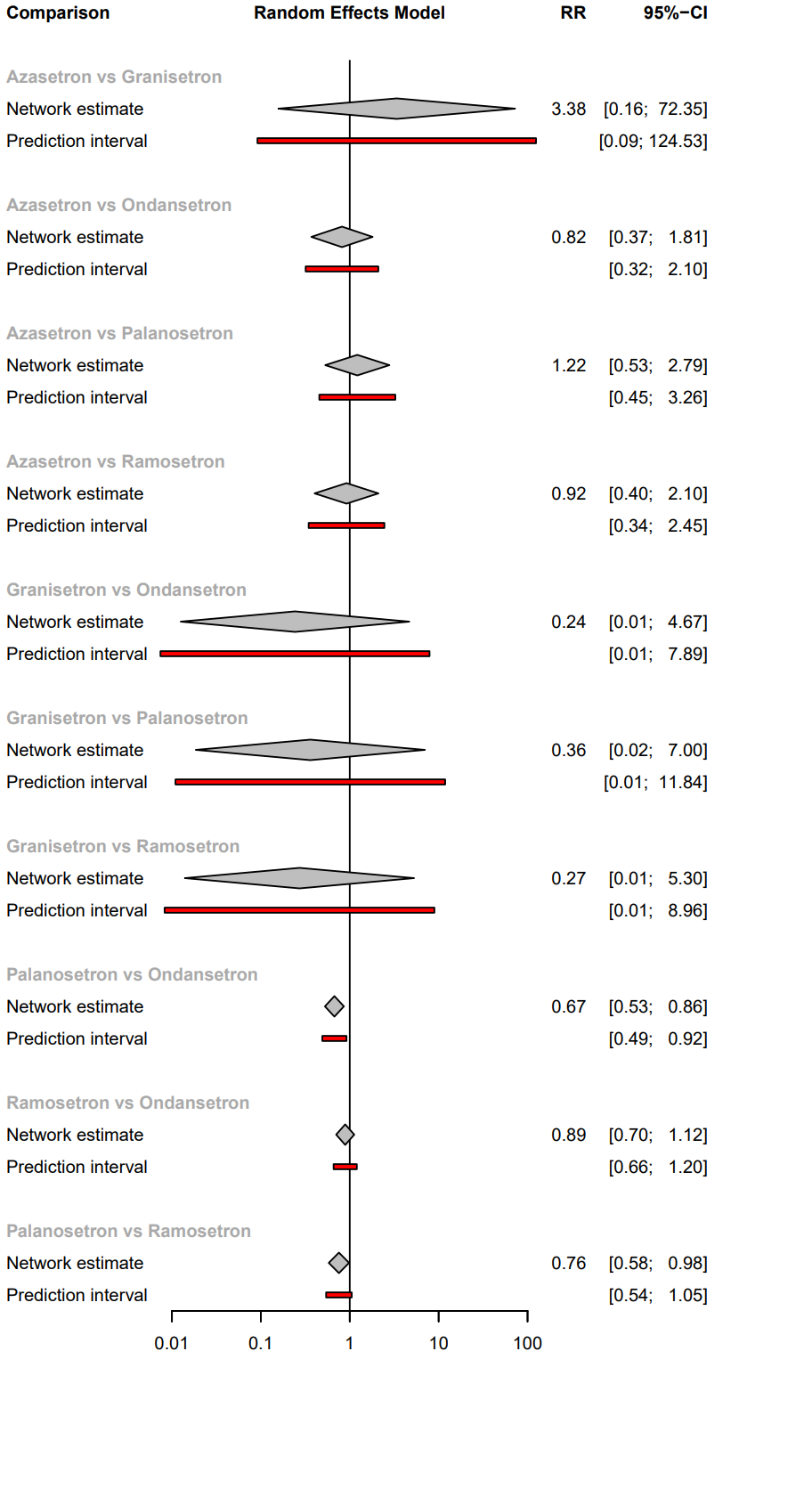


**Network calculation of “Late nausea”**

| Azasetron | . | 0.88 (0.37; 2.04) | . | . |
| --- | --- | --- | --- | --- |
| 1.88 (0.38; 9.21) | Granisetron | 0.38 (0.10; 1.47) | 3.00 (0.30; 29.52) | . |
| 0.88 (0.37; 2.04) | 0.47 (0.12; 1.78) | Ondansetron | 1.51 (1.01; 2.26) | 0.86 (0.50; 1.49) |
| 1.16 (0.46; 2.94) | 0.62 (0.16; 2.47) | 1.33 (0.92; 1.93) | Palanosetron | 1.20 (0.56; 2.56) |
| 0.94 (0.36; 2.47) | 0.50 (0.12; 2.06) | 1.07 (0.68; 1.70) | 0.81 (0.48; 1.34) | Ramosetron |

**P-score of “Late nausea”**

Granisetron 0.8083

Palanosetron 0.6509

Azasetron 0.4412

Ramosetron 0.3590

Ondansetron 0.2405

**Forest diagram of “Late nausea”**


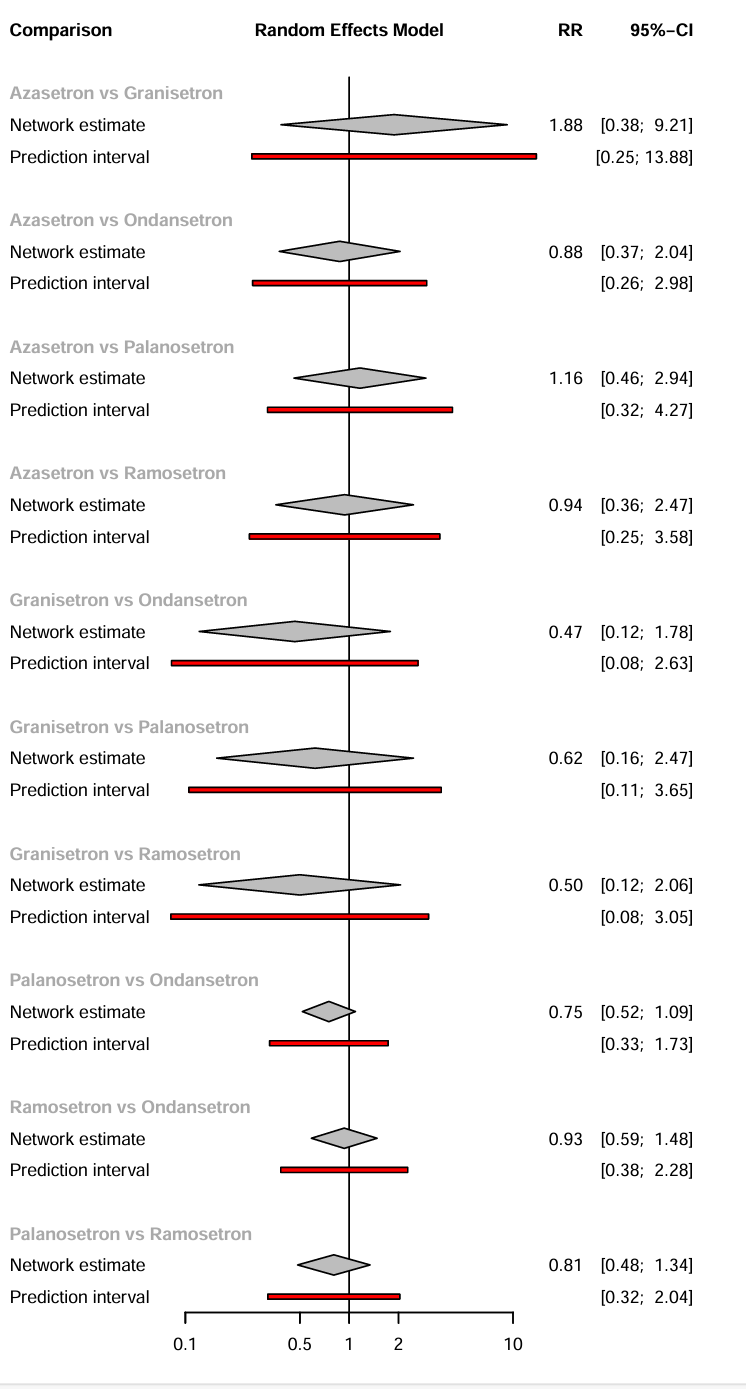


**Network calculation of “>24h nausea”**

| Azasetron | 1.50 (0.43; 5.25) | . | . |
| --- | --- | --- | --- |
| 1.50 (0.43; 5.25) | Ondansetron | 1.17 (0.54; 2.55) | 6.36 (1.31; 30.85) |
| 2.22 (0.52; 9.40) | 1.48 (0.72; 3.03) | Palanosetron | 1.08 (0.38; 3.13) |
| 3.69 (0.74; 18.44) | 2.46 (0.90; 6.74) | 1.66 (0.67; 4.12) | Ramosetron |

**P-score of “>24h nausea”**

Ramosetron 0.9225

Palanosetron 0.6175

Ondansetron 0.3069

Azasetron 0.1531

**Forest diagram of “>24h nausea”**


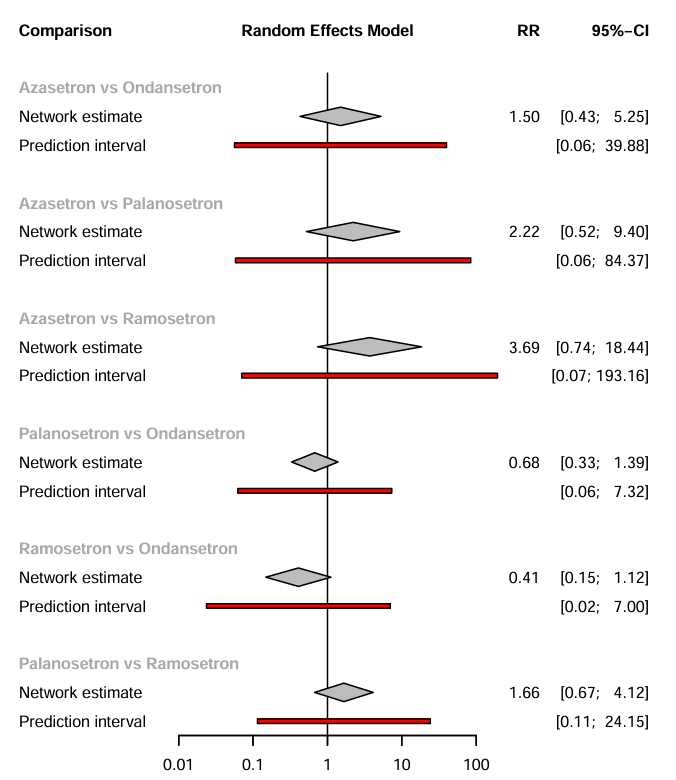


**Network calculation of “Overall nausea”**

| Azasetron | . | 0.84 (0.42;1.69) | . | . | . |
| --- | --- | --- | --- | --- | --- |
| 2.40 (0.70; 8.22) | Granisetron | 0.31 (0.11;0.87) | 3.00 (0.31; 29.19) | . | . |
| 0.84 (0.42; 1.69) | 0.35 (0.13; 0.96) | Ondansetron | 1.59 (1.06; 2.38) | 0.89 (0.45; 1.77) | 1.22 (0.63; 2.36) |
| 1.22 (0.55; 2.70) | 0.51 (0.17; 1.48) | 1.45 (1.00; 2.11) | Palanosetron | 0.97 (0.52; 1.80) | . |
| 0.96 (0.41; 2.28) | 0.40 (0.13; 1.24) | 1.15 (0.69; 1.90) | 0.79 (0.49; 1.29) | Ramosetron | . |
| 1.02 (0.39; 2.68) | 0.43 (0.13; 1.43) | 1.22 (0.63; 2.36) | 0.84 (0.39; 1.79) | 1.06 (0.46; 2.43) | Tropisetron |

**P-score of “Overall nausea”**

Granisetron 0.9300

Palanosetron 0.6548

Tropisetron 0.4417

Azasetron 0.4187

Ramosetron 0.3686

Ondansetron 0.1862

**Forest diagram of “Overall nausea”**


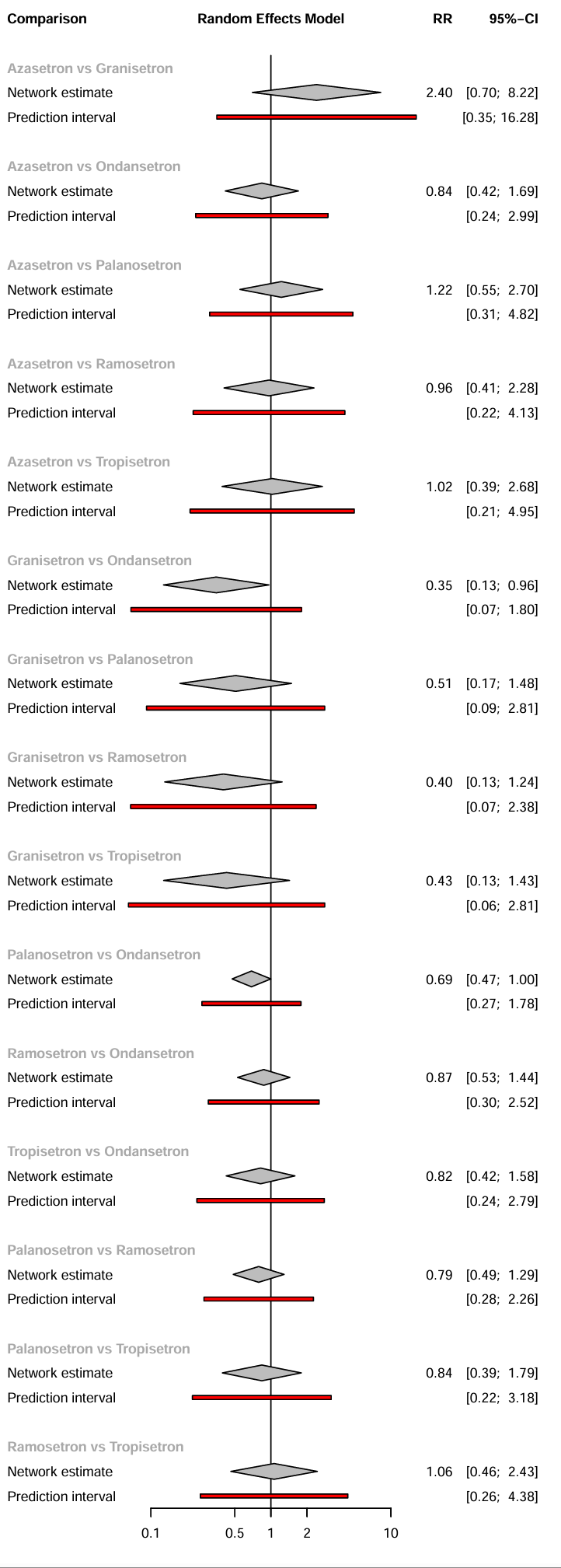


**Network calculation of “Acute vomiting”**

| Azasetron | . | 0.20 (0.01; 4.24) | . | . |
| --- | --- | --- | --- | --- |
| 0.54 (0.01; 41.95) | Granisetron | 0.33 (0.01; 8.20) | 1.00 (0.02; 50.48) | . |
| 0.20 (0.01; 4.24) | 0.37 (0.02; 8.28) | Ondansetron | 1.40 (0.67; 2.95) | 1.65 (0.92; 2.94) |
| 0.39 (0.02; 8.87) | 0.72 (0.03; 16.73) | 1.95 (1.01; 3.78) | Palanosetron | 0.46 (0.19; 1.11) |
| 0.27 (0.01; 5.99) | 0.50 (0.02; 11.58) | 1.35 (0.78; 2.32) | 0.69 (0.34; 1.40) | Ramosetron |

**P-score of “Acute vomiting”**

Azasetron 0.7445

Palanosetron 0.6302

Granisetron 0.5929

Ramosetron 0.3864

Ondansetron 0.1460

**Forest diagram of “Acute vomiting”**


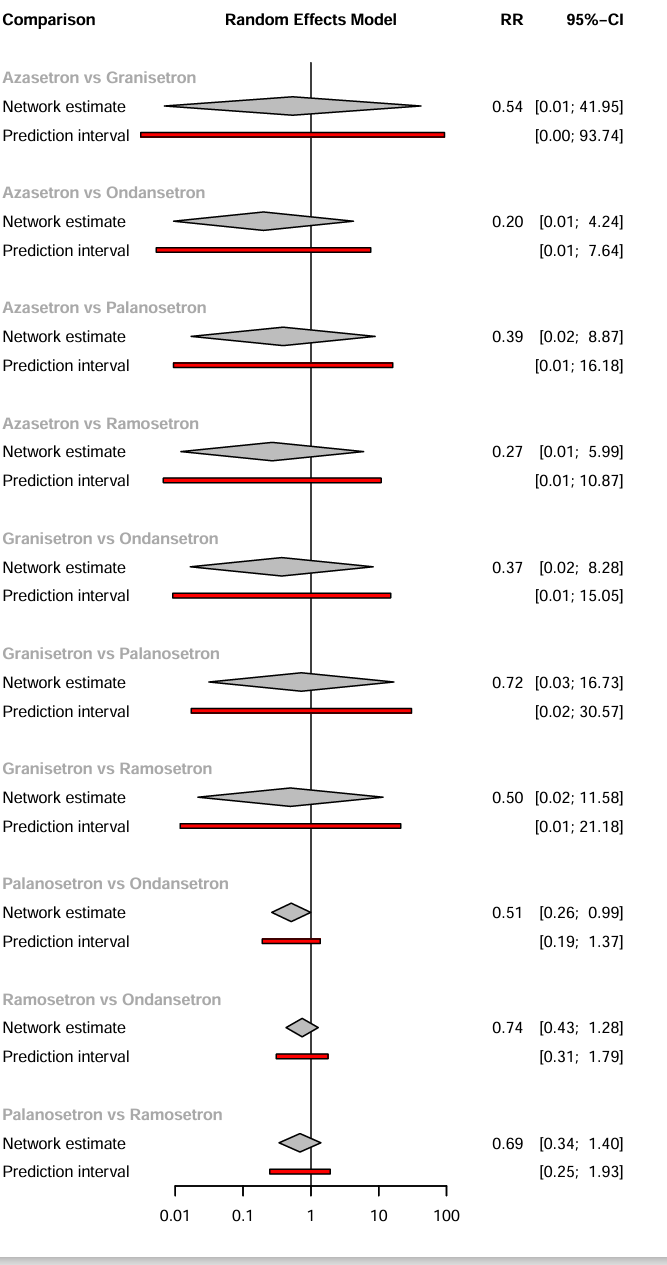


**Network calculation of “Late vomiting”**

| Azasetron | . | 2.00 (0.19; 21.34) | . | . |
| --- | --- | --- | --- | --- |
| 7.06 (0.44; 113.72) | Granisetron | 0.25 (0.06; 1.08) | 2.00 (0.19; 20.90) | . |
| 2.00 (0.19; 21.34) | 0.28 (0.07; 1.21) | Ondansetron | 1.96 (1.18; 3.25) | 1.20 (0.62; 2.34) |
| 3.80 (0.34; 42.57) | 0.54 (0.12; 2.46) | 1.90 (1.18; 3.06) | Palanosetron | 0.80 (0.23; 2.81) |
| 2.54 (0.22; 29.13) | 0.36 (0.07; 1.72) | 1.27 (0.70; 2.30) | 0.67 (0.33; 1.34) | Ramosetron |

**P-score of “Late vomiting”**

Granisetron 0.8898

Palanosetron 0.7357

Ramosetron 0.4453

Ondansetron 0.2458

Azasetron 0.1834

**Forest diagram of “Late vomiting”**


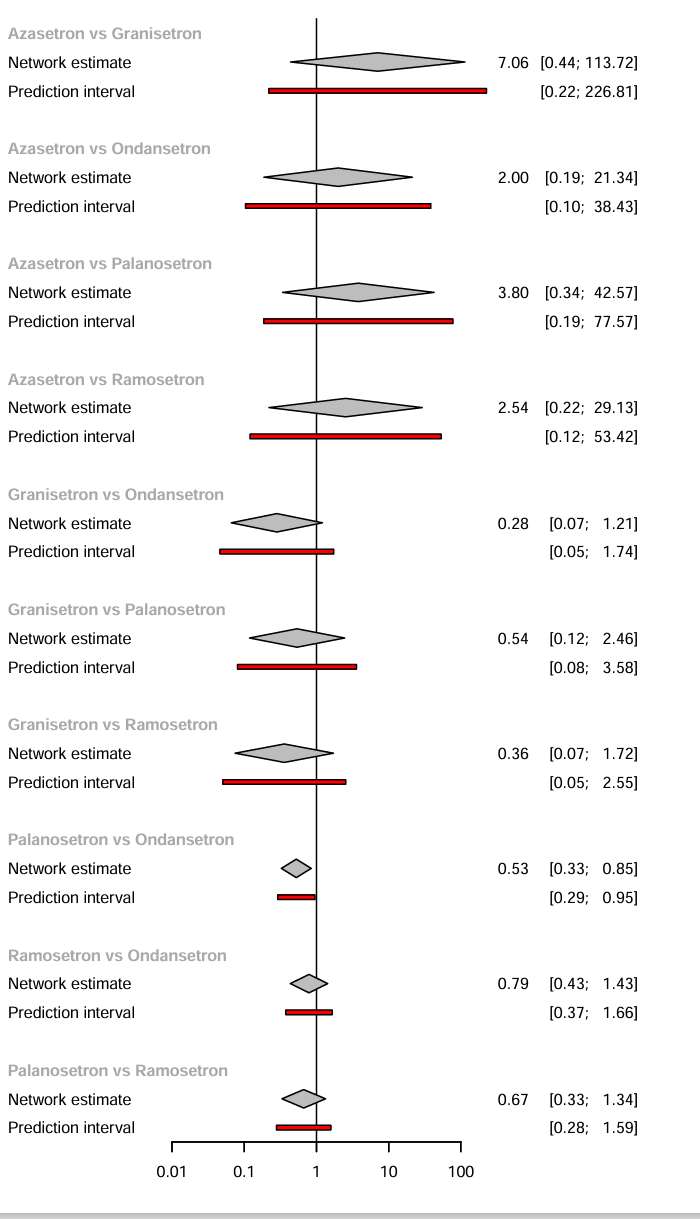


**Network calculation of “>24h vomiting”**

| Azasetron | 3.00 (0.13; 71.89) | . | . |
| --- | --- | --- | --- |
| 3.00 (0.13; 71.89) | Ondansetron | 3.93 (0.44; 35.00) | 2.34 (0.82; 6.69) |
| 16.71 (0.43; 644.68) | 5.57 (0.92; 33.82) | Palanosetron | 0.20 (0.01; 4.06) |
| 6.47 (0.23; 181.56) | 2.16 (0.78; 5.93) | 0.39 (0.06; 2.56) | Ramosetron |

**P-score of “>24h vomiting”**

Palanosetron 0.9136

Ramosetron 0.6529

Ondansetron 0.2834

Azasetron 0.1501

**Forest diagram of “>24h vomiting”**


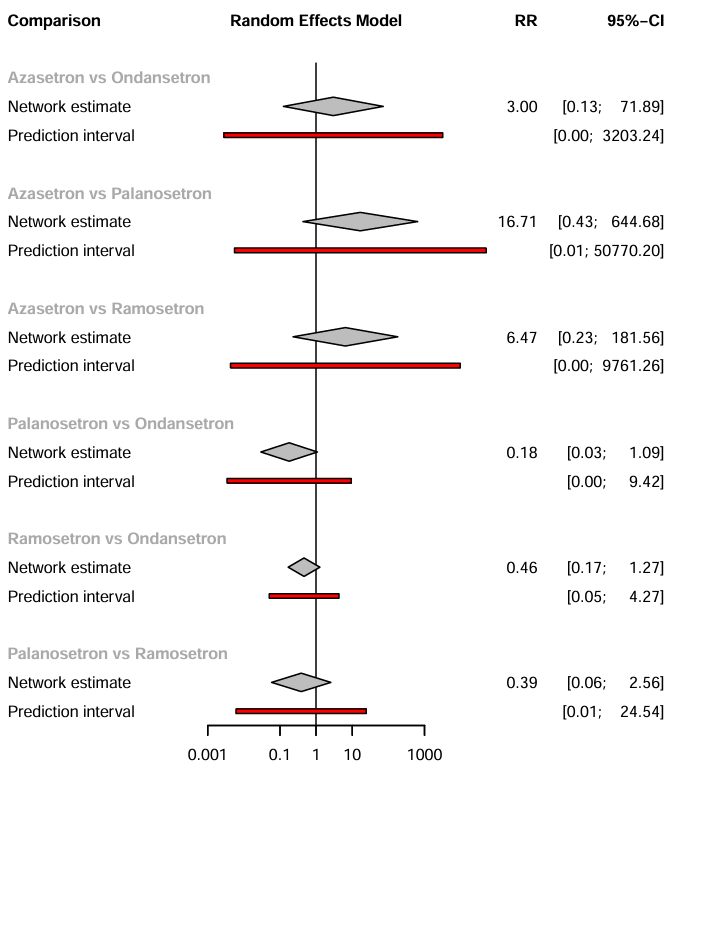


**Network calculation of “Overall vomiting”**

| Azasetron | . | 0.62 (0.13; 3.09) | . | . | . |
| --- | --- | --- | --- | --- | --- |
| 2.09 (0.18; 23.90) | Granisetron | 0.22 (0.03; 1.47) | 2.00 (0.14; 28.04) | . | . |
| 0.62 (0.13; 3.09) | 0.30 (0.05; 1.88) | Ondansetron | 1.70 (0.71; 4.06) | 1.22 (0.29; 5.21) | 2.17 (0.52; 9.09) |
| 1.16 (0.19; 6.92) | 0.55 (0.08; 3.88) | 1.85 (0.83; 4.13) | Palanosetron | 0.41 (0.10; 1.75) | . |
| 0.60 (0.09; 4.20) | 0.29 (0.03; 2.39) | 0.96 (0.32; 2.89) | 0.52 (0.17; 1.56) | Ramosetron | . |
| 1.36 (0.16; 11.60) | 0.65 (0.06; 6.69) | 2.17 (0.52; 9.09) | 1.17 (0.23; 6.05) | 2.25 (0.37; 13.68) | Tropisetron |

**P-score of “Overall vomiting”**

Granisetron 0.7729

Tropisetron 0.6417

Palanosetron 0.6151

Azasetron 0.5035

Ramosetron 0.2431

Ondansetron 0.2237

**Forest diagram of “Overall vomiting”**


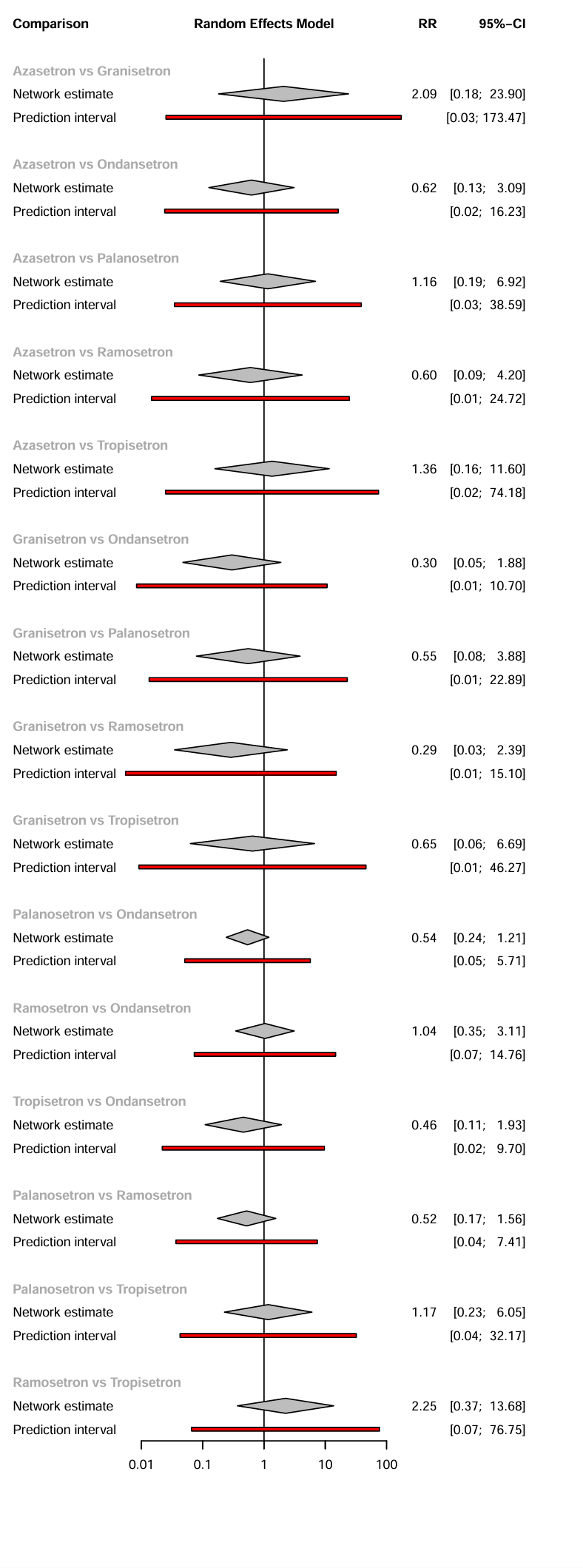


**Network calculation of “Acute PONV”**

| Dolasetron | 1.48 (0.64; 3.43) | 1.29 (0.57; 2.93) | . | . |
| --- | --- | --- | --- | --- |
| 1.51 (0.69; 3.28) | Granisetron | 0.84 (0.45; 1.56) | 1.00 (0.02; 51.27) | . |
| 1.27 (0.59; 2.73) | 0.84 (0.45; 1.57) | Ondansetron | 1.47 (0.90; 2.40) | 0.77 (0.29; 2.02) |
| 1.67 (0.69; 4.05) | 1.11 (0.51; 2.39) | 1.31 (0.84; 2.06) | Palanosetron | 1.03 (0.57; 1.87) |
| 1.47 (0.56; 3.89) | 0.98 (0.41; 2.32) | 1.16 (0.63; 2.12) | 0.88 (0.52; 1.49) | Ramosetron |

**P-score of “Acute PONV”**

Palanosetron 0.7597

Granisetron 0.6170

Ramosetron 0.5644

Ondansetron 0.3669

Dolasetron 0.1920

**Forest diagram of “Acute PONV”**


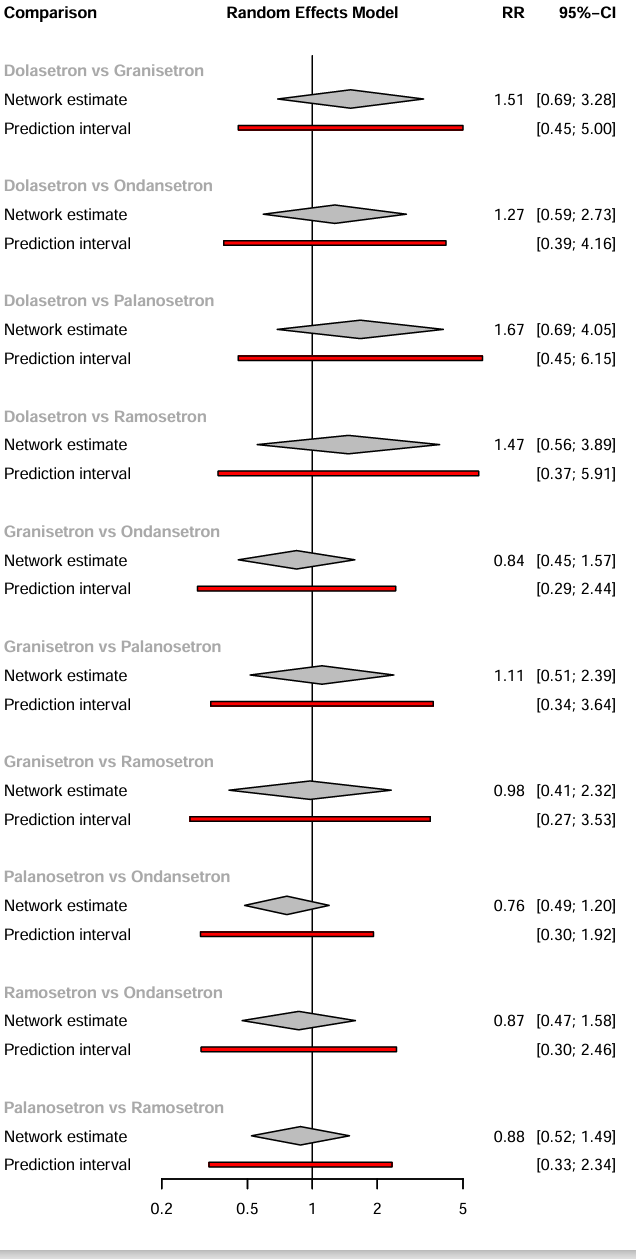


**Network calculation of “Late PONV”**

| Dolasetron | 1.06 (0.50; 2.26) | 0.94 (0.46; 1.95) | . | . |
| --- | --- | --- | --- | --- |
| 1.09 (0.53; 2.23) | Granisetron | 0.79 (0.43; 1.47) | 2.50 (0.49; 12.68) | . |
| 0.92 (0.46; 1.86) | 0.85 (0.46; 1.55) | Ondansetron | 1.44 (1.04; 2.00) | 0.71 (0.30; 1.68) |
| 1.24 (0.58; 2.65) | 1.14 (0.58; 2.23) | 1.34 (0.99; 1.83) | Palanosetron | 0.97 (0.63; 1.51) |
| 1.07 (0.46; 2.45) | 0.98 (0.46; 2.08) | 1.15 (0.73; 1.83) | 0.86 (0.58; 1.28) | Ramosetron |

**P-score of “Late PONV”**

Palanosetron 0.7751

Granisetron 0.5442

Ramosetron 0.4974

Dolasetron 0.4304

Ondansetron 0.2528

**Forest diagram of “Late PONV”**


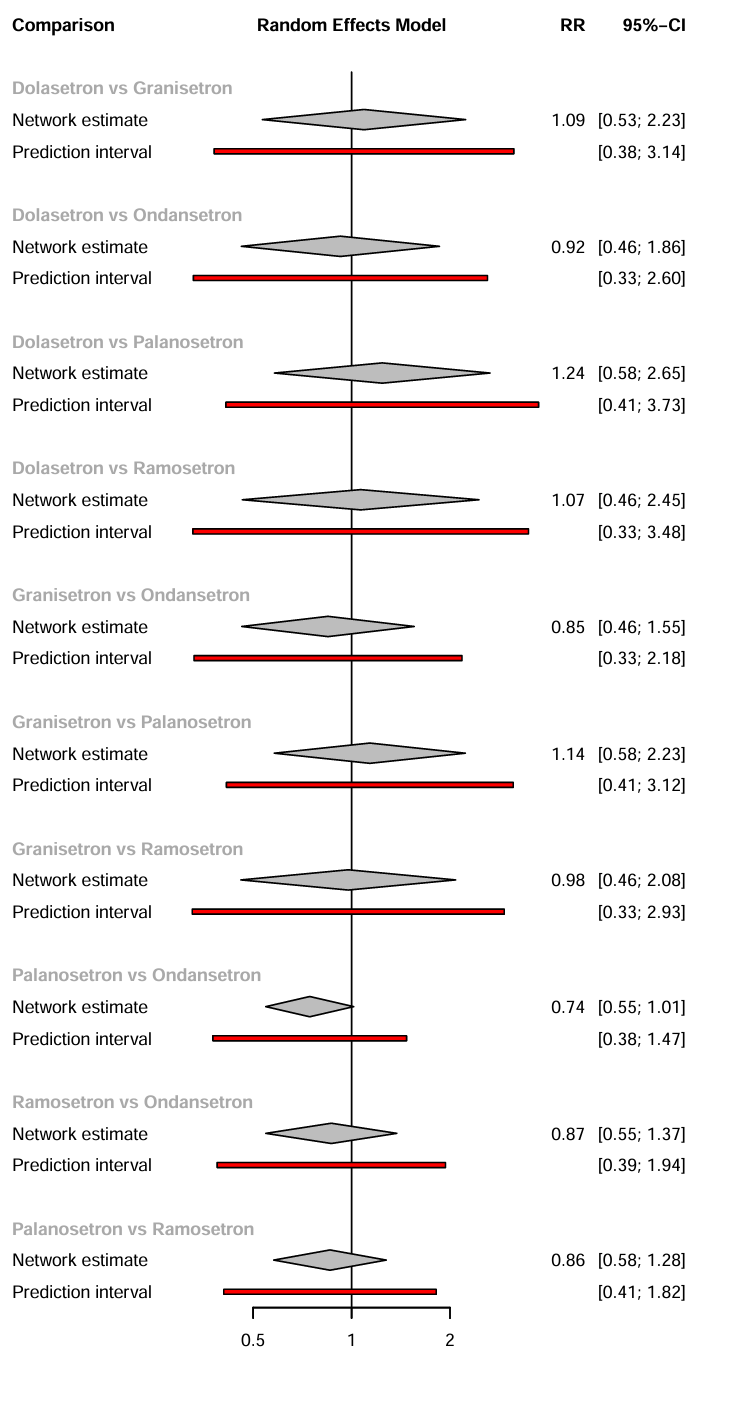


**Network calculation of “>24h PONV”**

| Ondansetron | 1.28 (0.70; 2.34) | 8.00 (0.98; 65.21) |
| --- | --- | --- |
| 1.46 (0.82; 2.62) | Palanosetron | 0.97 (0.57; 1.63) |
| 1.57 (0.74; 3.31) | 1.07 (0.64; 1.78) | Ramosetron |

**P-score of “>24h PONV”**

Ramosetron 0.7423

Palanosetron 0.6486

Ondansetron 0.1091

**Forest diagram of “>24h PONV”**


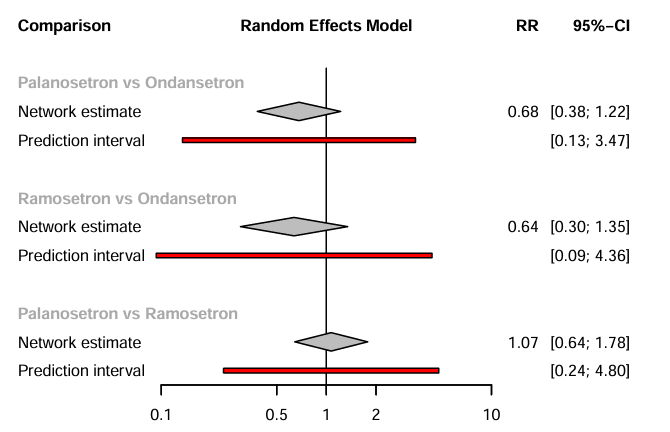


**Network calculation of “Overall PONV”**

| Azasetron | . | . | 0.75 (0.44; 1.27) | . | . |
| --- | --- | --- | --- | --- | --- |
| 0.61 (0.29; 1.28) | Dolasetron | 1.25 (0.72; 2.19) | 1.33 (0.76; 2.34) | . | . |
| 0.81 (0.39; 1.69) | 1.35 (0.79; 2.30) | Granisetron | 0.88 (0.53; 1.47) | 2.50 (0.50; 12.48) | . |
| 0.75 (0.44; 1.27) | 1.24 (0.72; 2.13) | 0.92 (0.55; 1.53) | Ondansetron | 1.36 (1.03; 1.80) | 1.05 (0.72; 1.54) |
| 0.99 (0.55; 1.78) | 1.64 (0.90; 2.98) | 1.22 (0.69; 2.15) | 1.32 (1.02; 1.71) | Palanosetron | 0.96 (0.66; 1.39) |
| 0.88 (0.48; 1.62) | 1.45 (0.78; 2.71) | 1.08 (0.59; 1.96) | 1.17 (0.86; 1.60) | 0.88 (0.65; 1.21) | Ramosetron |

**P-score of “Overall PONV”**

Palanosetron 0.7910

Azasetron 0.7290

Ramosetron 0.5744

Granisetron 0.4848

Ondansetron 0.2956

Dolasetron 0.1252

**Forest diagram of “Overall PONV”**


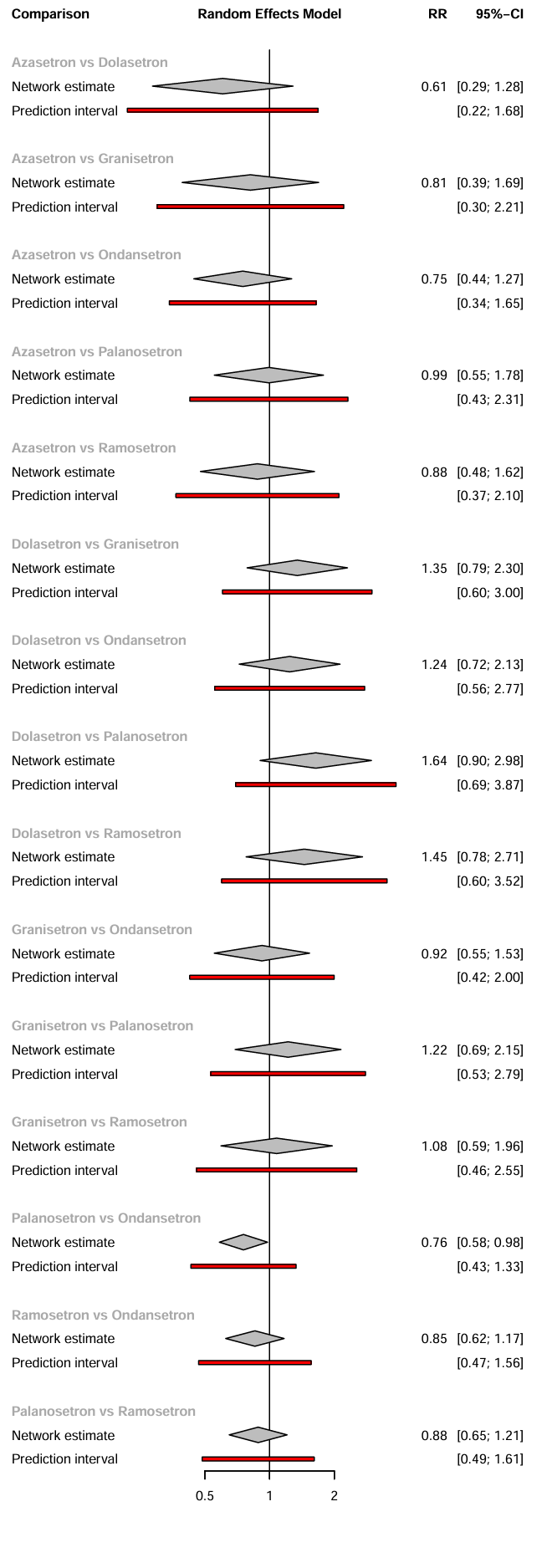


**Network calculation of “Acute rescue medicine”**

| Azasetron | . | 1.00 (0.31; 3.24) | . | . |
| --- | --- | --- | --- | --- |
| 2.30 (0.09; 61.47) | Granisetron | 0.33 (0.01; 7.87) | 1.00 (0.02; 48.82) | . |
| 1.00 (0.31; 3.24) | 0.43 (0.02; 9.35) | Ondansetron | 1.03 (0.65; 1.63) | 1.12 (0.69; 1.81) |
| 1.01 (0.29; 3.56) | 0.44 (0.02; 9.60) | 1.01 (0.64; 1.59) | Palanosetron | 1.14 (0.47; 2.75) |
| 1.11 (0.31; 3.93) | 0.48 (0.02; 10.71) | 1.11 (0.69; 1.78) | 1.10 (0.60; 2.00) | Ramosetron |

**P-score of “Acute rescue medicine”**

Granisetron 0.6926

Ramosetron 0.5428

Azasetron 0.4350

Palanosetron 0.4260

Ondansetron 0.4037

**Forest diagram of “Acute rescue medicine”**


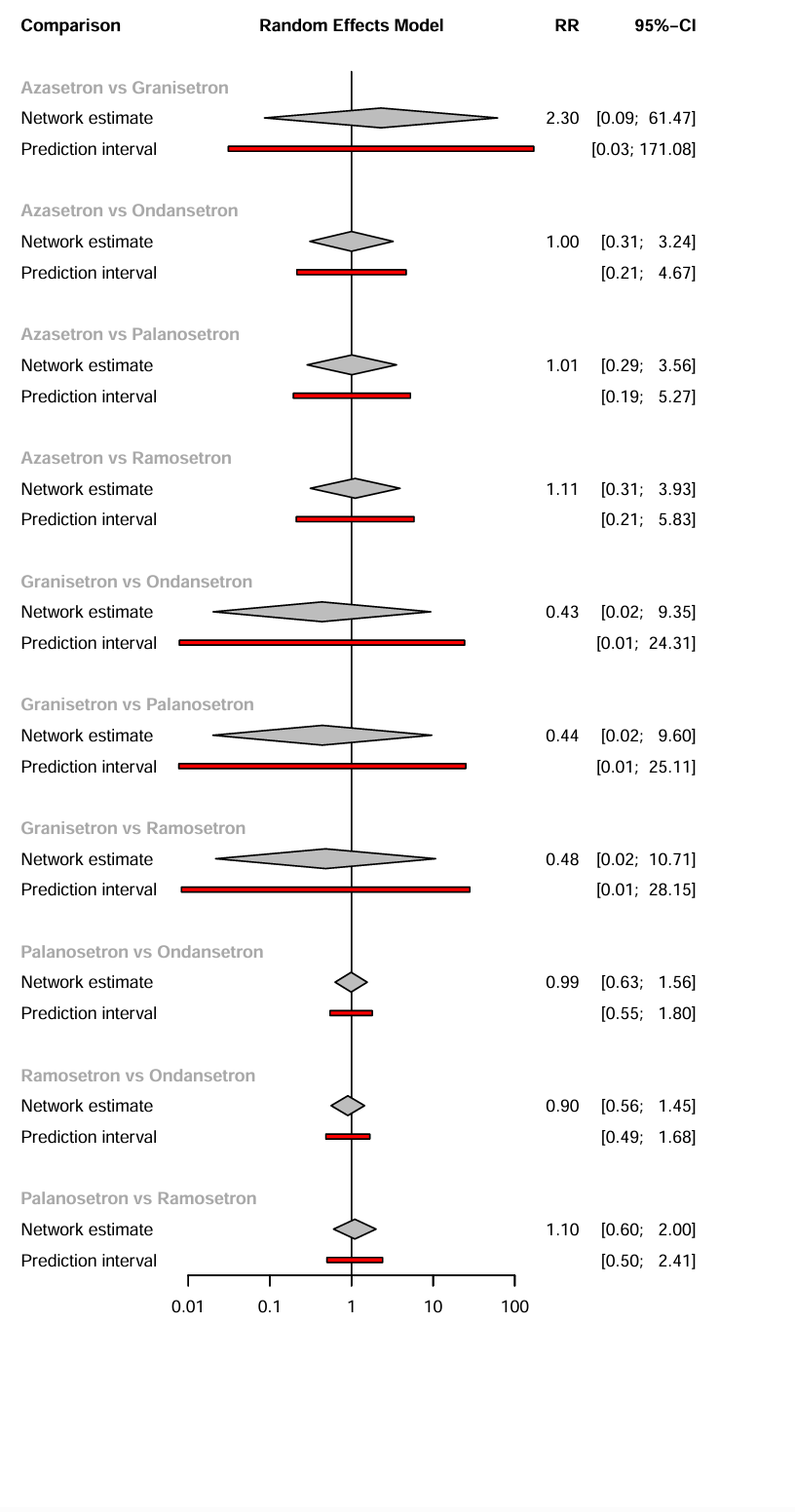


**Network calculation of “Late rescue medicine”**

| Azasetron | . | 1.00 (0.29; 3.50) | . | . |
| --- | --- | --- | --- | --- |
| 3.43 (0.48; 24.47) | Granisetron | 0.25 (0.05; 1.15) | 2.00 (0.18; 21.76) | . |
| 1.00 (0.29; 3.50) | 0.29 (0.06; 1.33) | Ondansetron | 1.84 (1.10; 3.08) | 1.05 (0.51; 2.17) |
| 1.84 (0.48; 7.15) | 0.54 (0.11; 2.61) | 1.84 (1.10; 3.08) | Palanosetron | . |
| 1.05 (0.25; 4.47) | 0.31 (0.06; 1.64) | 1.05 (0.51; 2.17) | 0.57 (0.23; 1.38) | Ramosetron |

**P-score of “Late rescue medicine”**

Granisetron 0.8826

Palanosetron 0.7291

Azasetron 0.3177

Ramosetron 0.3176

Ondansetron 0.2530

**Forest diagram of “Late rescue medicine”**


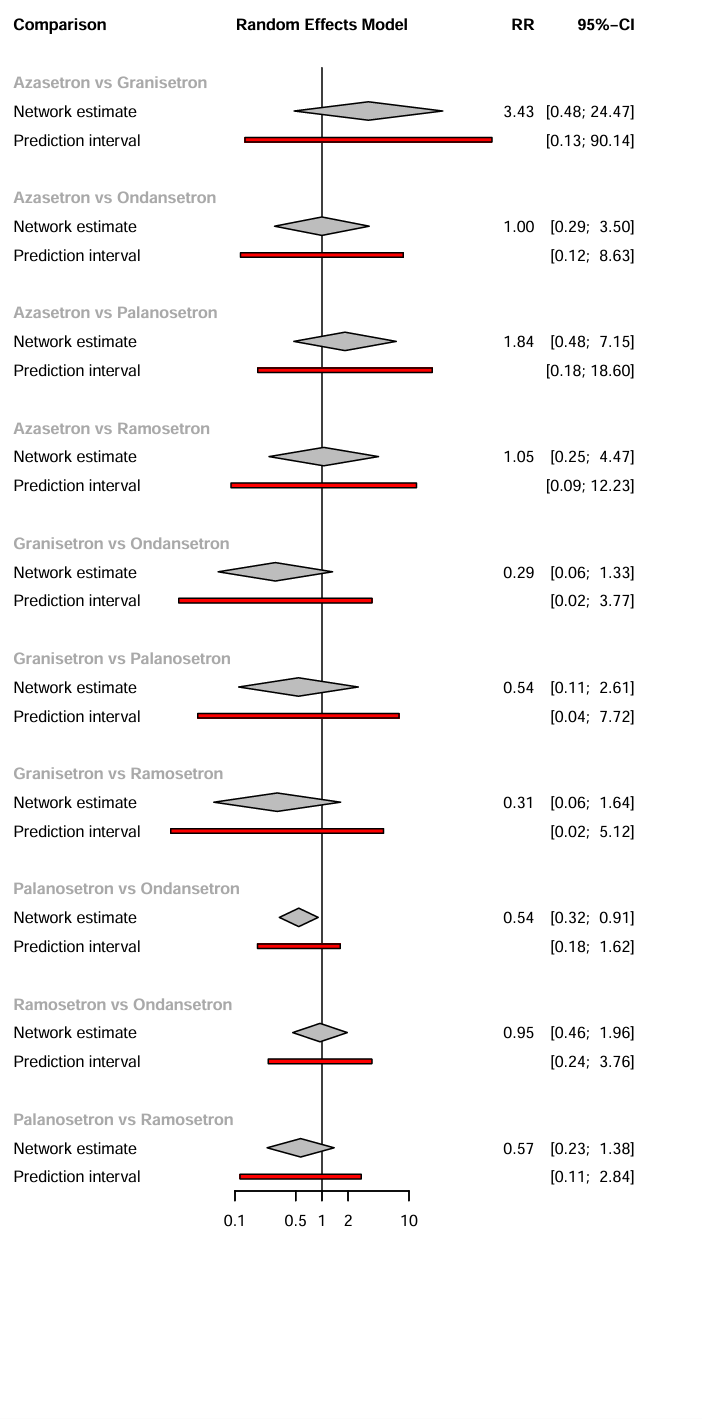


**Network calculation of “>24h rescue medicine”**

| Azasetron | 3.00 (0.13; 71.89) | . | . |
| --- | --- | --- | --- |
| 3.00 (0.13; 71.89) | Ondansetron | 2.40 (1.03; 5.57) | 5.34 (0.95; 30.12) |
| 7.19 (0.27; 192.43) | 2.40 (1.03; 5.57) | Palanosetron | . |
| 16.02 (0.43; 596.29) | 5.34 (0.95; 30.12) | 2.23 (0.32; 15.26) | Ramosetron |

**P-score of “>24h rescue medicine”**

Ramosetron 0.8990

Palanosetron 0.6889

Ondansetron 0.2670

Azasetron 0.1450

**Forest diagram of “>24h rescue medicine”**


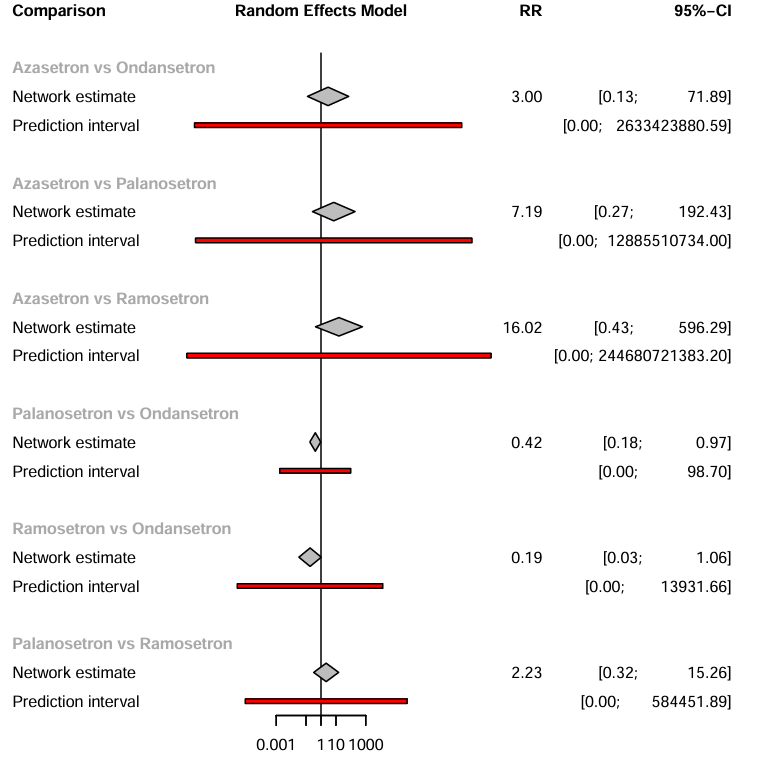


**Network calculation of “Overall rescue medicine”**

| Granisetron | 0.22 (0.04; 1.10) | 2.00 (0.17; 23.06) | . | . |
| --- | --- | --- | --- | --- |
| 0.28 (0.06; 1.36) | Ondansetron | 1.14 (0.63; 2.06) | 1.83 (0.83; 4.04) | 1.25 (0.51; 3.01) |
| 0.36 (0.07; 1.90) | 1.30 (0.75; 2.24) | Palanosetron | 0.70 (0.23; 2.14) | . |
| 0.41 (0.07; 2.25) | 1.45 (0.74; 2.83) | 1.11 (0.53; 2.34) | Ramosetron | . |
| 0.35 (0.06; 2.14) | 1.25 (0.51; 3.01) | 0.96 (0.34; 2.71) | 0.86 (0.28; 2.61) | Tropisetron |

**P-score of “Overall rescue medicine”**

Granisetron 0.8874

Ramosetron 0.5569

Palanosetron 0.4649

Tropisetron 0.4198

Ondansetron 0.1710

**Forest diagram of “Overall rescue medicine”**


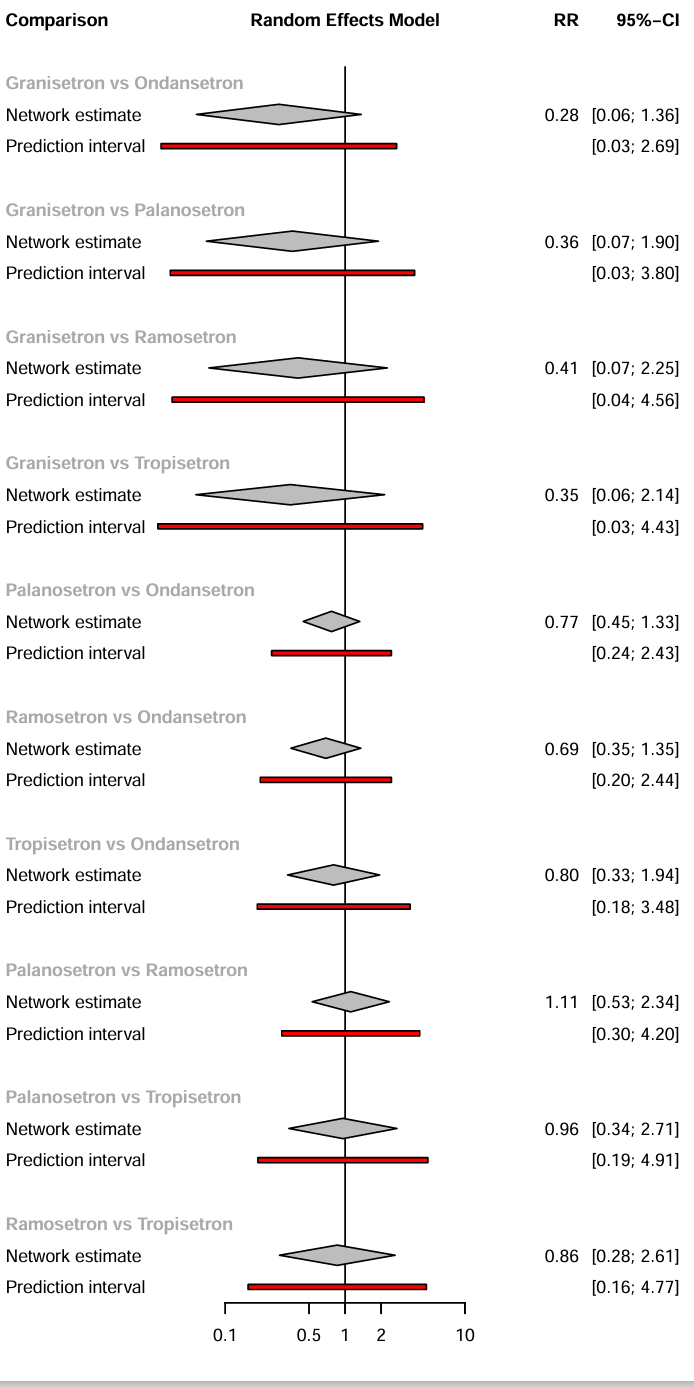


**Network calculation of “Adverse reaction”**

| Azasetron | . | 0.92 (0.40; 2.09) | . | . |
| --- | --- | --- | --- | --- |
| 0.67 (0.19; 2.38) | Granisetron | 1.14 (0.41; 3.14) | 5.00 (0.60; 41.91) | . |
| 0.92 (0.40; 2.09) | 1.37 (0.52; 3.61) | Ondansetron | 0.96 (0.73; 1.27) | 1.07 (0.72; 1.58) |
| 0.91 (0.38; 2.16) | 1.36 (0.51; 3.68) | 0.99 (0.77; 1.29) | Palanosetron | 0.96 (0.61; 1.54) |
| 0.91 (0.38; 2.23) | 1.37 (0.49; 3.80) | 1.00 (0.71; 1.40) | 1.00 (0.70; 1.43) | Ramosetron |

**P-score of “Adverse reaction”**

Azasetron 0.6190

Ondansetron 0.5448

Ramosetron 0.5380

Palanosetron 0.5307

Granisetron 0.2675

**Forest diagram of “Adverse reaction”**


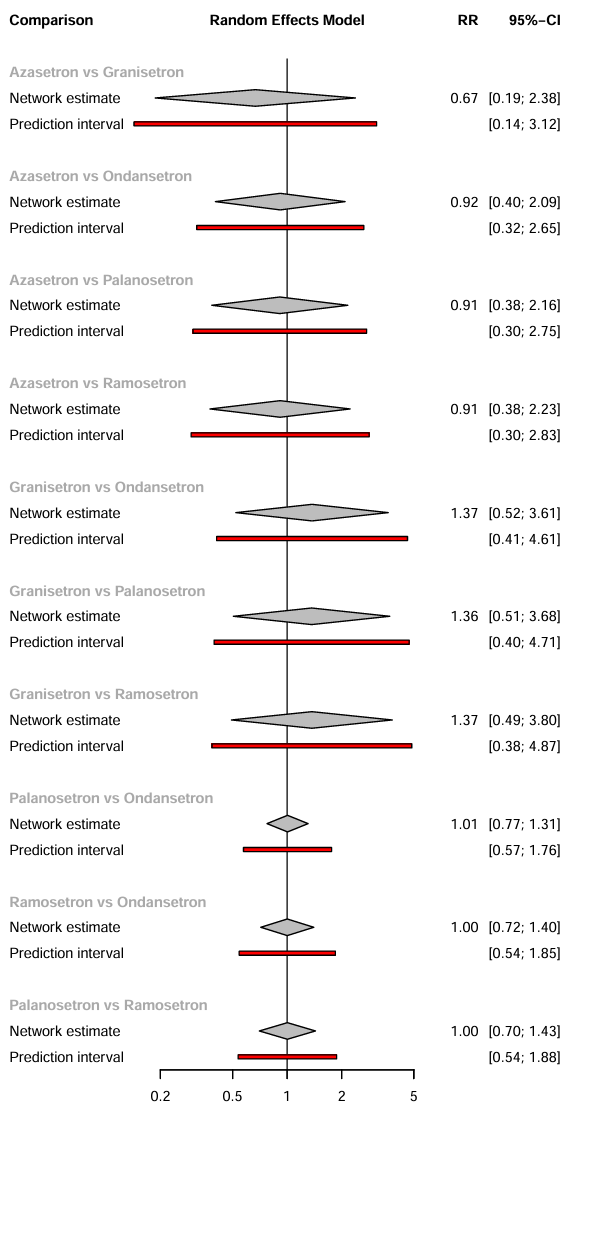

Supplement: Supplementary file 8 — Data S8. [file IJGO-171-177-s003.docx]
