# Supplementary material for: 5‐HT3 receptor antagonists for preventing postoperative nausea and vomiting after gynecological surgery: A systematic review and network meta‐analysis
Source: Int J Gynaecol Obstet. 2025 May 9;171(1):177–89. doi: 10.1002/ijgo.70197 (PMC12447676; doi:10.1002/ijgo.70197)

**Data S9 Sensitivity of exclusing spinal anaesthesia**

**Network calculation of “Acute nausea”**

| Azasetron | . | 0.82 (0.37; 1.80) | . | . |
| --- | --- | --- | --- | --- |
| 1.40 (0.41; 4.74) | Granisetron | 0.20 (0.01; 4.00) | 0.84 (0.30; 2.41) | 0.83 (0.28; 2.48) |
| 0.82 (0.37; 1.80) | 0.58 (0.23; 1.48) | Ondansetron | 1.47 (1.13; 1.92) | 1.14 (0.88;1.47) |
| 1.21 (0.53; 2.76) | 0.87 (0.34; 2.18) | 1.48 (1.17; 1.88) | Palanosetron | 0.82 (0.63; 1.09) |
| 0.93 (0.41; 2.12) | 0.67 (0.26; 1.68) | 1.14 (0.90; 1.44) | 0.77 (0.60; 0.99) | Ramosetron |

**P-score of “Acute nausea”**

Palanosetron 0.7587

Granisetron 0.7507

Azasetron 0.4687

Ramosetron 0.3785

Ondansetron 0.1434

**Forest diagram of “Acute nausea”**


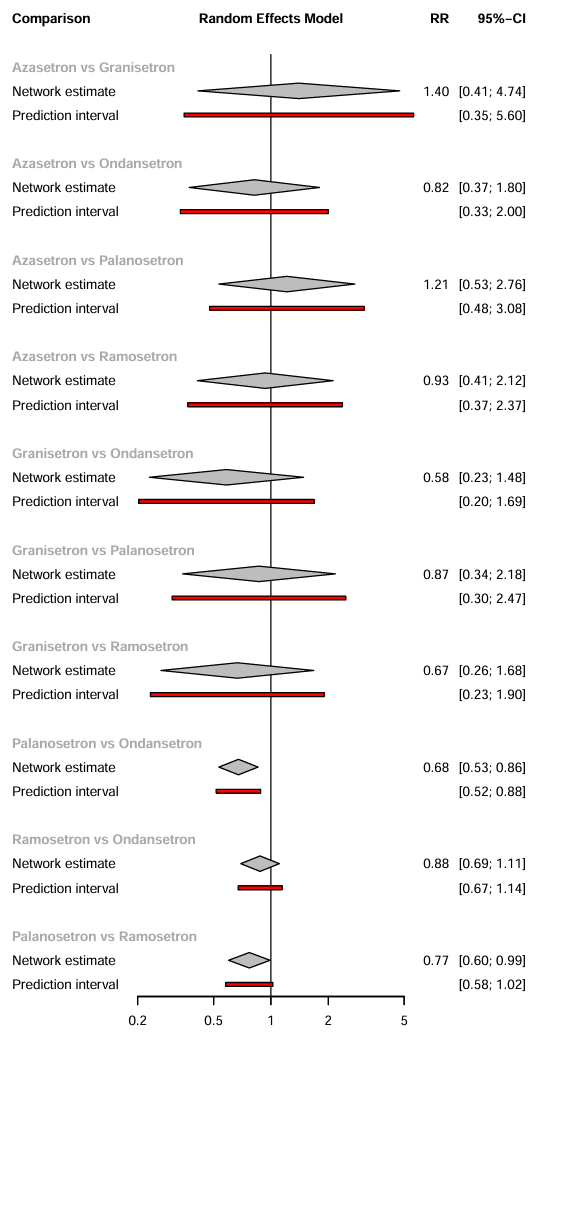


**Network calculation of “Late nausea”**

| Azasetron | . | 0.88 (0.41; 1.89) | . | . |
| --- | --- | --- | --- | --- |
| 1.28 (0.39; 4.13) | Granisetron | 0.41 (0.12; 1.45) | 1.71 (0.49; 5.97) | 1.00 (0.25; 4.02) |
| 0.88 (0.41; 1.89) | 0.69 (0.28; 1.67) | Ondansetron | 1.50 (1.05; 2.15) | 0.81 (0.47; 1.38) |
| 1.17 (0.51; 2.71) | 0.92 (0.37; 2.26) | 1.34 (0.96; 1.86) | Palanosetron | 1.12 (0.60; 2.08) |
| 0.95 (0.39; 2.28) | 0.74 (0.30; 1.86) | 1.08 (0.71; 1.66) | 0.81 (0.52; 1.26) | Ramosetron |

**P-score of “Late nausea”**

Palanosetron 0.7139

Granisetron 0.6918

Azasetron 0.4695

Ramosetron 0.3830

Ondansetron 0.2419

**Forest diagram of “Late nausea”**


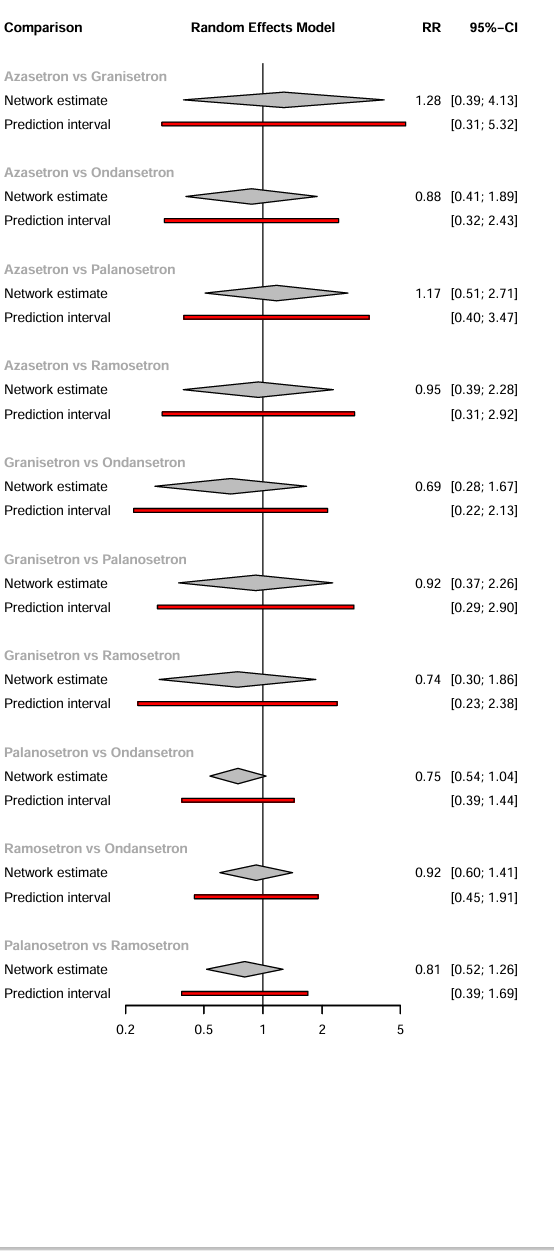


**Network calculation of “>24h nausea”**

| Azasetron | . | 1.50 (0.55; 4.11) | . | . |
| --- | --- | --- | --- | --- |
| 3.71 (0.30; 46.21) | Granisetron | . | 0.50 (0.05; 5.39) | 1.00 (0.06; 15.66) |
| 1.50 (0.55; 4.11) | 0.40 (0.04; 4.08) | Ondansetron | 1.27 (0.74; 2.19) | 8.00 (1.01; 63.63) |
| 2.11 (0.68; 6.57) | 0.57 (0.06; 5.41) | 1.41 (0.83; 2.38) | Palanosetron | 1.15 (0.56; 2.35) |
| 2.87 (0.78; 10.56) | 0.77 (0.08; 7.63) | 1.91 (0.84; 4.37) | 1.36 (0.69; 2.69) | Ramosetron |

**P-score of “>24h nausea”**

Ramosetron 0.7766

Granisetron 0.7252

Palanosetron 0.5743

Ondansetron 0.2925

Azasetron 0.1313

**Forest diagram of “>24h nausea”**


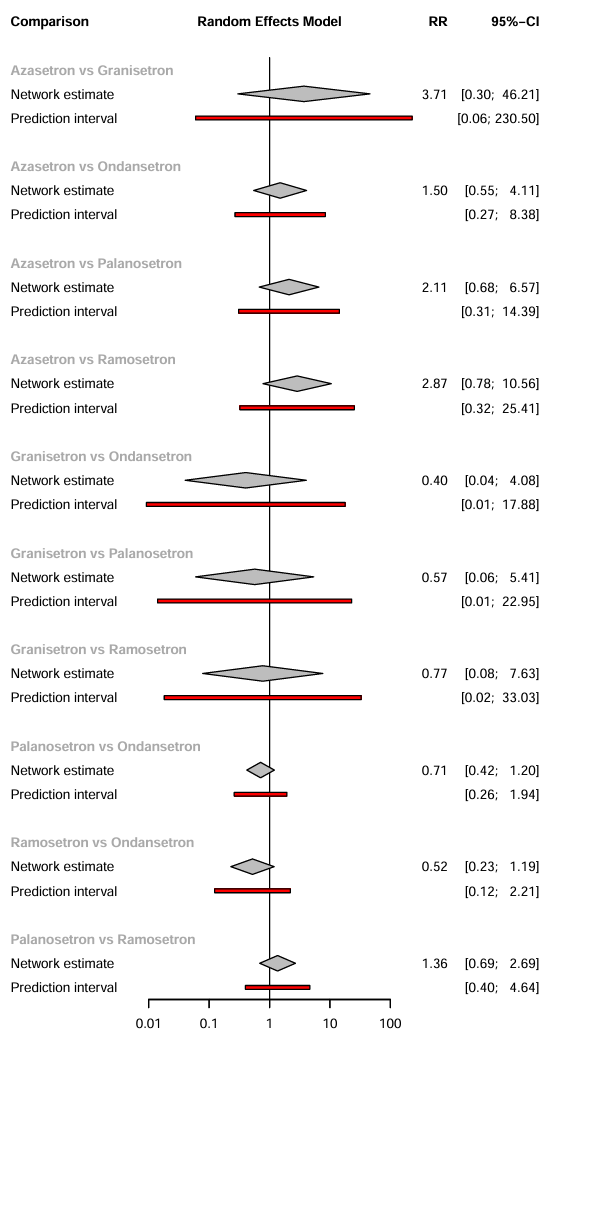


**Network calculation of “Overall nausea”**

| Azasetron | . | 0.84 (0.46; 1.52) | . | . | . |
| --- | --- | --- | --- | --- | --- |
| 2.22 (0.79; 6.23) | Granisetron | 0.35 (0.15; 0.82) | 3.00 (0.32; 28.31) | . | . |
| 0.84 (0.46; 1.52) | 0.38 (0.16; 0.88) | Ondansetron | 1.60 (1.14; 2.25) | 1.05 (0.68; 1.62) | 1.22 (0.71; 2.10) |
| 1.24 (0.63; 2.41) | 0.56 (0.23; 1.36) | 1.47 (1.09; 1.99) | Palanosetron | 0.97 (0.59; 1.59) | . |
| 1.01 (0.51; 2.02) | 0.46 (0.18; 1.13) | 1.20 (0.85; 1.71) | 0.82 (0.57; 1.18) | Ramosetron | . |
| 1.02 (0.46; 2.29) | 0.46 (0.17; 1.26) | 1.22 (0.71; 2.10) | 0.83 (0.44; 1.54) | 1.01 (0.53; 1.94) | Tropisetron |

**P-score of “Overall nausea”**

Granisetron 0.9424

Palanosetron 0.6818

Tropisetron 0.4292

Ramosetron 0.4062

Azasetron 0.4026

Ondansetron 0.1379

**Forest diagram of “Overall nausea”**


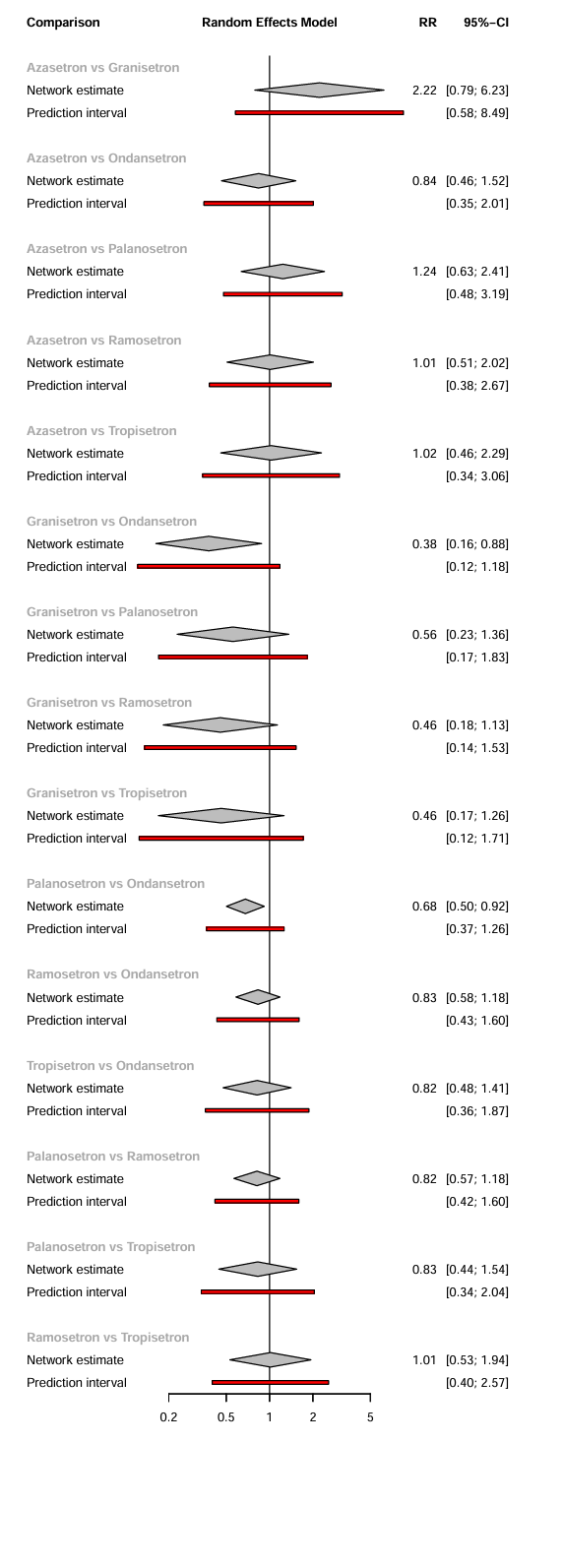


**Network calculation of “Acute vomiting”**

| Azasetron | . | 0.20 (0.01; 4.06) | . | . |
| --- | --- | --- | --- | --- |
| 0.73 (0.02; 29.57) | Granisetron | 0.33 (0.01; 7.87) | 0.52 (0.04; 6.02) | 0.33 (0.01; 7.91) |
| 0.20 (0.01; 4.06) | 0.27 (0.03; 2.37) | Ondansetron | 1.54 (0.79; 3.01) | 1.70 (0.86; 3.35) |
| 0.40 (0.02; 8.53) | 0.54 (0.06; 4.71) | 1.98 (1.08; 3.62) | Palanosetron | 0.51 (0.24; 1.08) |
| 0.26 (0.01; 5.58) | 0.36 (0.04; 3.12) | 1.29 (0.71; 2.37) | 0.65 (0.34; 1.26) | Ramosetron |

**P-score of “Acute vomiting”**

Azasetron 0.7370

Granisetron 0.7120

Palanosetron 0.6127

Ramosetron 0.3174

Ondansetron 0.1209

**Forest diagram of “Acute vomiting”**


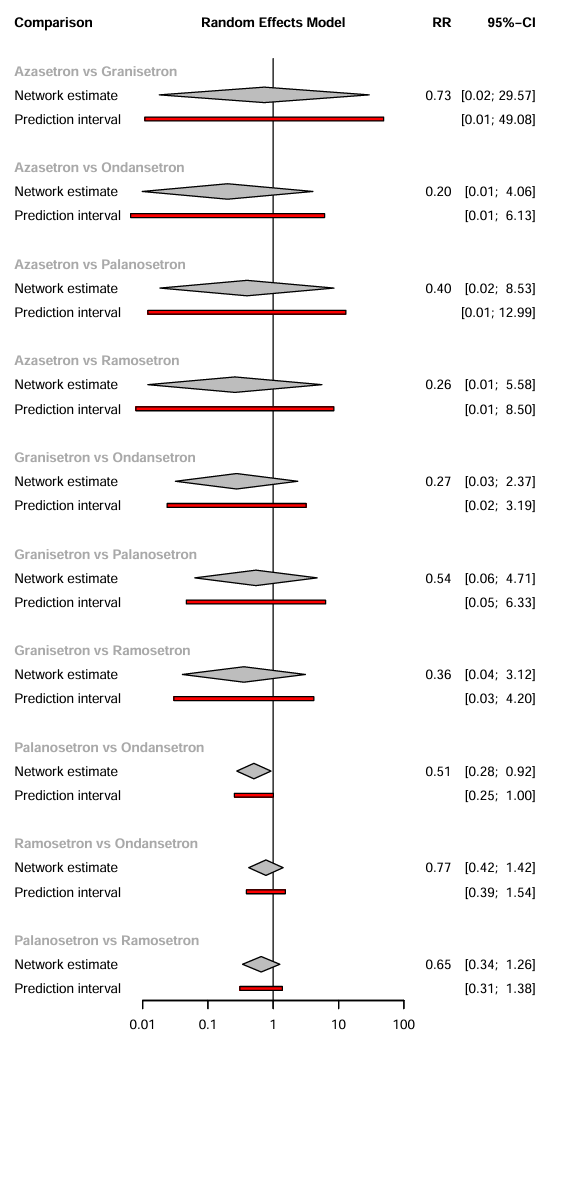


**Network calculation of “Late vomiting”**

| Azasetron | . | 2.00 (0.19; 21.34) | . | . |
| --- | --- | --- | --- | --- |
| 7.06 (0.49; 101.08) | Granisetron | 0.26 (0.07; 0.99) | 1.06 (0.16; 6.98) | 1.00 (0.02; 49.04) |
| 2.00 (0.19; 21.34) | 0.28 (0.08; 0.96) | Ondansetron | 2.01 (1.22; 3.31) | 1.23 (0.51; 2.96) |
| 3.83 (0.34; 42.77) | 0.54 (0.15; 1.93) | 1.91 (1.20; 3.06) | Palanosetron | 0.96 (0.30; 3.07) |
| 2.84 (0.24; 33.70) | 0.40 (0.10; 1.61) | 1.42 (0.69; 2.91) | 0.74 (0.35; 1.59) | Ramosetron |

**P-score of “Late vomiting”**

Granisetron 0.9078

Palanosetron 0.7026

Ramosetron 0.4867

Ondansetron 0.2278

Azasetron 0.1751

**Forest diagram of “Late vomiting”**


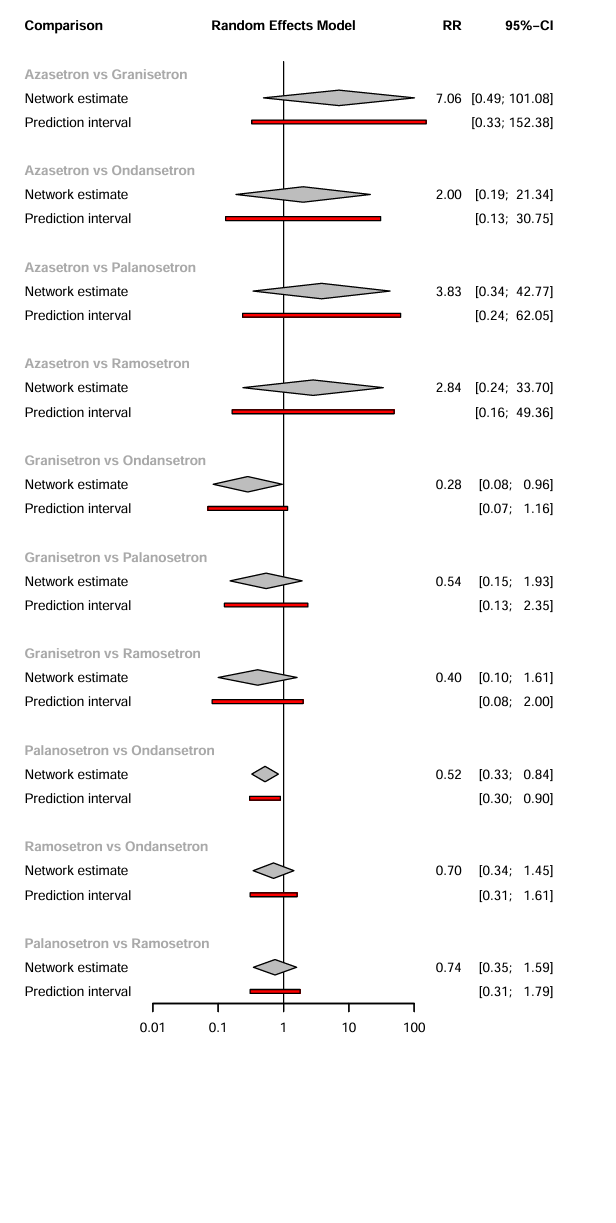


**Network calculation of “>24h vomiting”**

| Azasetron | . | 3.00 (0.13; 71.89) | . | . |
| --- | --- | --- | --- | --- |
| 5.08 (0.03; 739.50) | Granisetron | . | 1.00 (0.02; 49.04) | 1.00 (0.02; 49.04) |
| 3.00 (0.13; 71.89) | 0.59 (0.01; 27.34) | Ondansetron | 2.83 (0.42; 19.02) | 1.00 (0.02; 49.59) |
| 8.44 (0.22; 318.73) | 1.66 (0.05; 56.62) | 2.81 (0.48; 16.35) | Palanosetron | 0.37 (0.03; 3.96) |
| 3.06 (0.06; 164.40) | 0.60 (0.02; 20.53) | 1.02 (0.09; 11.29) | 0.36 (0.04; 2.93) | Ramosetron |

**P-score of “>24h vomiting”**

Palanosetron 0.7977

Granisetron 0.5863

Ramosetron 0.4438

Ondansetron 0.4407

Azasetron 0.2315

**Forest diagram of “>24h vomiting”**


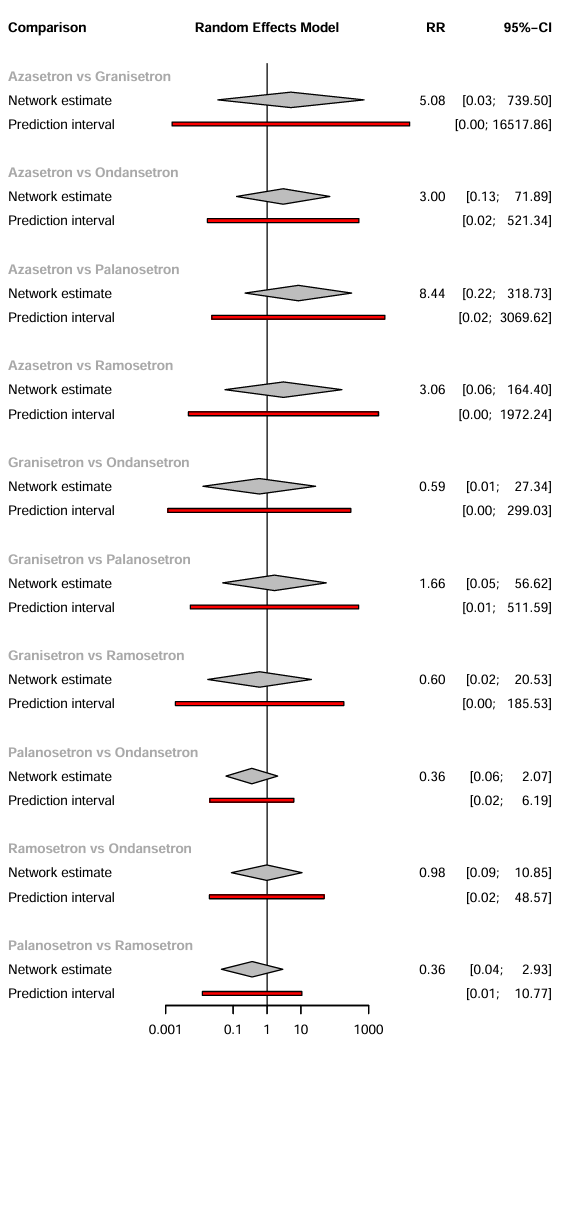


**Network calculation of “Overall vomiting”**

| Azasetron | . | 0.62 (0.16; 2.42) | . | . | . |
| --- | --- | --- | --- | --- | --- |
| 1.73 (0.30; 9.87) | Granisetron | 0.33 (0.11; 1.00) | 2.00 (0.16; 24.33) | . | . |
| 0.62 (0.16; 2.42) | 0.36 (0.12; 1.09) | Ondansetron | 1.71 (0.84; 3.47) | 0.89 (0.38; 2.08) | 1.30 (0.58; 2.94) |
| 1.11 (0.25; 4.96) | 0.65 (0.18; 2.25) | 1.78 (0.94; 3.36) | Palanosetron | 0.41 (0.13; 1.32) | . |
| 0.52 (0.11; 2.41) | 0.30 (0.08; 1.12) | 0.83 (0.41;1.71) | 0.47 (0.21;1.04) | Ramosetron | . |
| 0.81 (0.17; 3.94) | 0.47 (0.12; 1.85) | 1.30 (0.58; 2.94) | 0.73 (0.26; 2.05) | 1.56 (0.53; 4.61) | Tropisetron |

**P-score of “Overall vomiting”**

Granisetron 0.8543

Palanosetron 0.6918

Azasetron 0.5730

Tropisetron 0.4675

Ondansetron 0.2547

Ramosetron 0.1587

**Forest diagram of “Overall vomiting”**


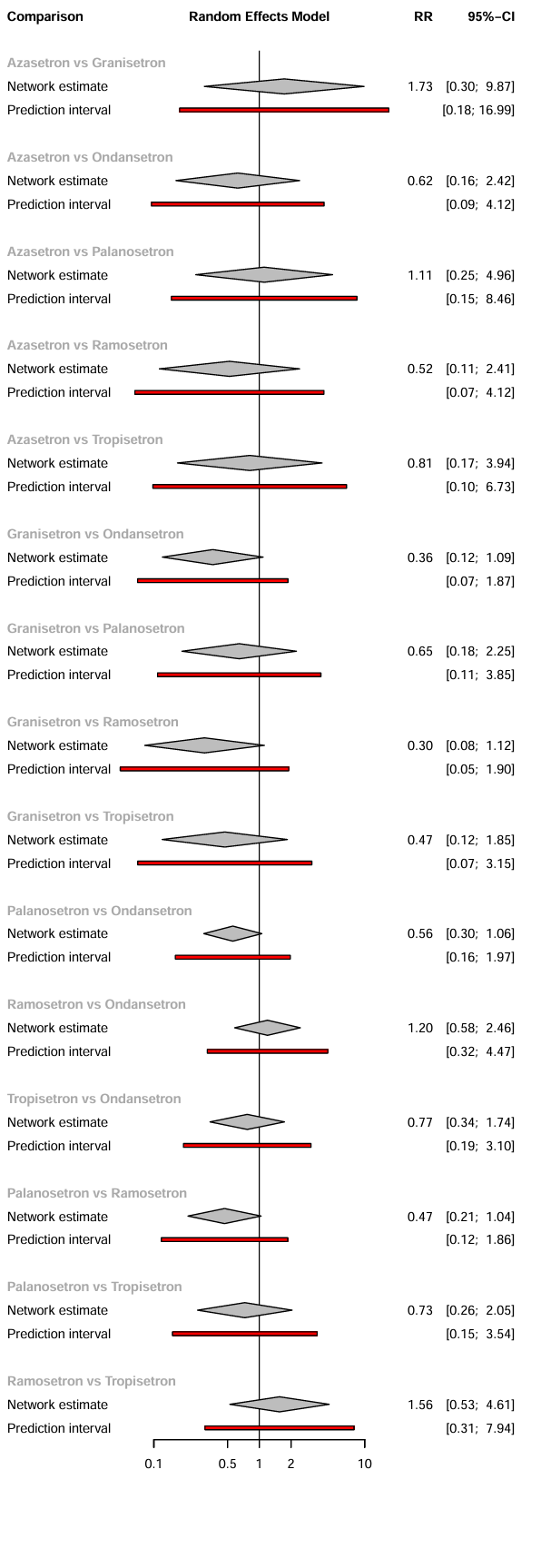


**Network calculation of “Acute PONV”**

| Dolasetron | 1.48 (0.70; 3.11) | 1.29 (0.63; 2.64) | . | . |
| --- | --- | --- | --- | --- |
| 1.50 (0.76; 2.97) | Granisetron | 0.85 (0.48; 1.48) | 1.00 (0.02; 50.24) | . |
| 1.28 (0.66; 2.49) | 0.85 (0.49; 1.49) | Ondansetron | 1.44 (0.92;2.26) | 0.89 (0.54; 1.48) |
| 1.61 (0.75; 3.47) | 1.07 (0.55; 2.11) | 1.26 (0.86; 1.85) | Palanosetron | 0.99 (0.59; 1.66) |
| 1.34 (0.61; 2.92) | 0.89 (0.45; 1.78) | 1.05 (0.70; 1.57) | 0.83 (0.55; 1.26) | Ramosetron |

**P-score of “Acute PONV”**

Palanosetron 0.7904

Granisetron 0.6574

Ramosetron 0.4813

Ondansetron 0.3950

Dolasetron 0.1759

**Forest diagram of “Acute PONV”**


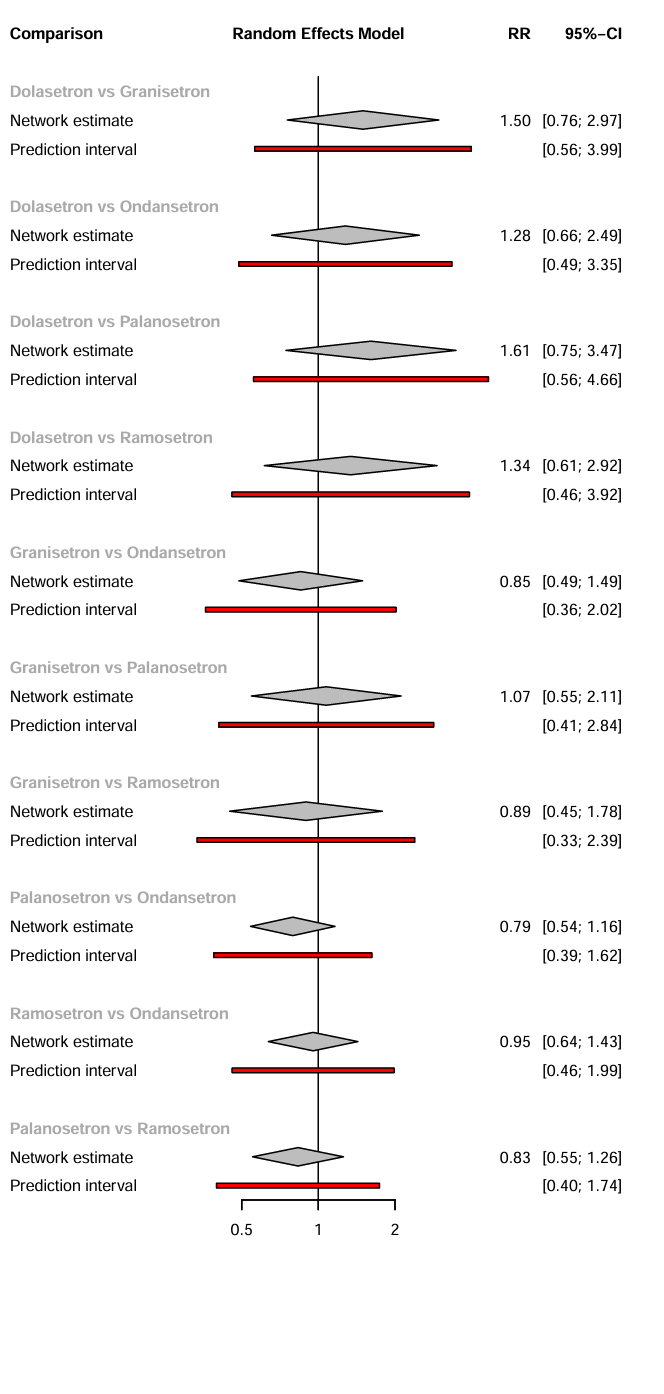


**Network calculation of “Late PONV”**

| Dolasetron | 1.06 (0.53; 2.13) | 0.94 (0.49; 1.84) | . | . |
| --- | --- | --- | --- | --- |
| 1.11 (0.57; 2.15) | Granisetron | 0.78 (0.44; 1.37) | 2.50 (0.51; 12.35) | . |
| 0.91 (0.48; 1.73) | 0.82 (0.47; 1.44) | Ondansetron | 1.44 (1.07; 1.93) | 0.85 (0.45; 1.62) |
| 1.23 (0.61; 2.45) | 1.11 (0.60; 2.05) | 1.34 (1.02; 1.77) | Palanosetron | 0.97 (0.66; 1.43) |
| 1.06 (0.50; 2.24) | 0.96 (0.49; 1.88) | 1.16 (0.79; 1.72) | 0.87 (0.62; 1.22) | Ramosetron |

**P-score of “Late PONV”**

Palanosetron 0.7811

Granisetron 0.5752

Ramosetron 0.4975

Dolasetron 0.4275

Ondansetron 0.2188

**Forest diagram of “Late PONV”**


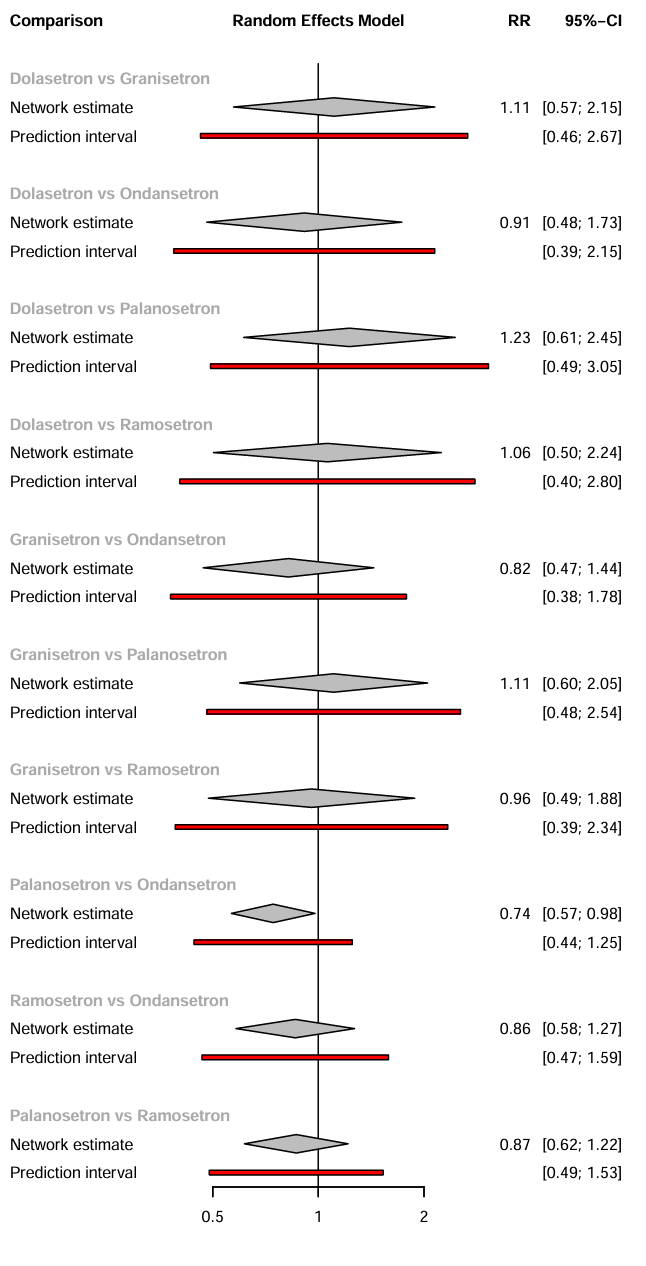


**Network calculation of “>24h PONV”**

| Ondansetron | 1.28 (0.70; 2.34) | 8.00 (0.98; 65.21) |
| --- | --- | --- |
| 1.46 (0.82; 2.62) | Palanosetron | 0.97 (0.57; 1.63) |
| 1.57 (0.74; 3.31) | 1.07 (0.64; 1.78) | Ramosetron |

**P-score of “>24h PONV”**

Ramosetron 0.7423

Palanosetron 0.6486

Ondansetron 0.1091

**Forest diagram of “>24h PONV”**


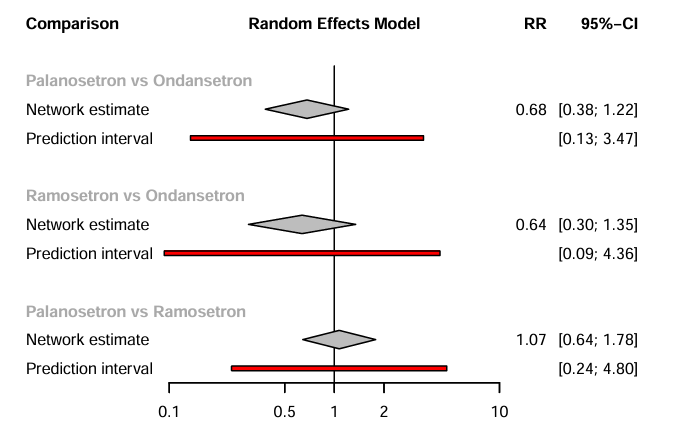


**Network calculation of “Overall PONV”**

| Azasetron | . | . | 0.75 (0.48; 1.18) | . | . |
| --- | --- | --- | --- | --- | --- |
| 0.65 (0.34; 1.23) | Dolasetron | 1.25 (0.77; 2.05) | 1.33 (0.81; 2.19) | . | . |
| 0.93 (0.52; 1.66) | 1.44 (0.92; 2.26) | Granisetron | 0.78 (0.51; 1.20) | 0.95 (0.50; 1.79) | 1.22 (0.55; 2.72) |
| 0.75 (0.48; 1.18) | 1.16 (0.73; 1.82) | 0.80 (0.56; 1.15) | Ondansetron | 1.35 (1.05; 1.73) | 0.93 (0.70; 1.24) |
| 0.92 (0.56; 1.52) | 1.42 (0.87; 2.31) | 0.98 (0.67; 1.45) | 1.23 (0.99; 1.52) | Palanosetron | 1.03 (0.77; 1.38) |
| 0.82 (0.49; 1.36) | 1.26 (0.76; 2.07) | 0.87 (0.58; 1.31) | 1.09 (0.86; 1.38) | 0.89 (0.70; 1.13) | Ramosetron |

**P-score of “Overall PONV”**

Azasetron 0.7613

Palanosetron 0.7119

Granisetron 0.7029

Ramosetron 0.4409

Ondansetron 0.2464

Dolasetron 0.1365

**Forest diagram of “Overall PONV”**


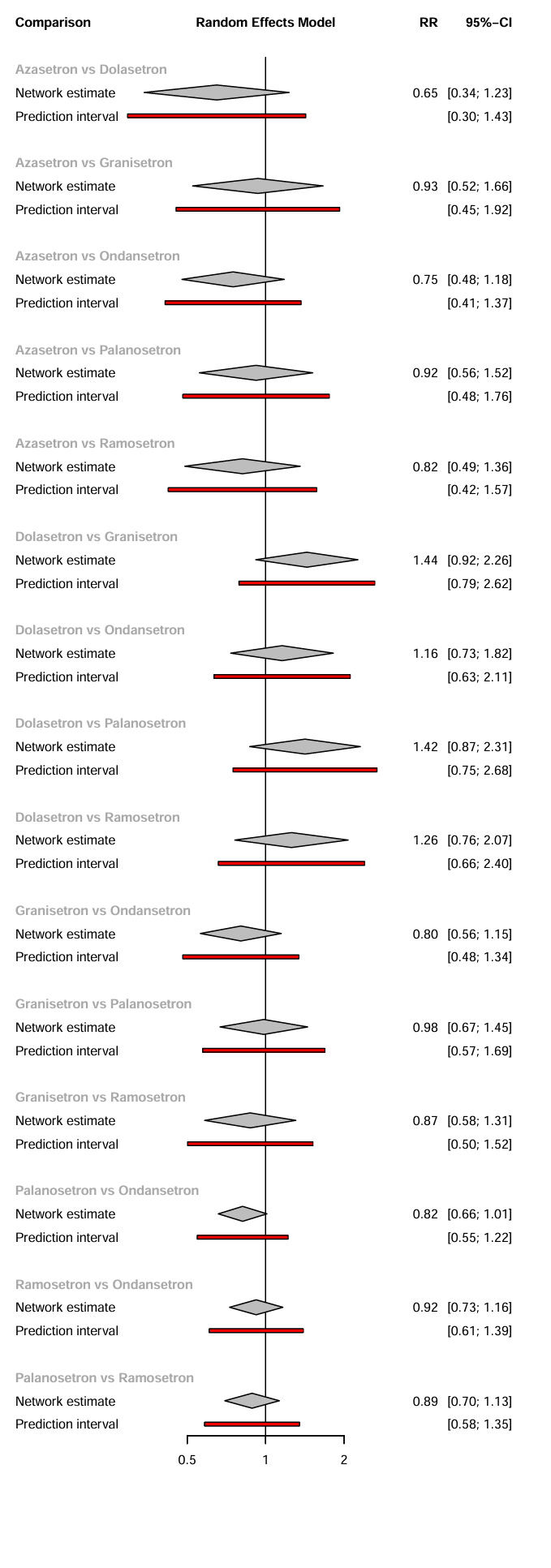


**Network calculation of “Acute rescue medicine”**

| Azasetron | . | 1.00 (0.31; 3.24) | . | . |
| --- | --- | --- | --- | --- |
| 1.91 (0.22; 16.64) | Granisetron | 0.33 (0.01; 7.87) | 0.60 (0.08; 4.51) | 1.00 (0.07; 15.36) |
| 1.00 (0.31; 3.24) | 0.52 (0.08; 3.24) | Ondansetron | 1.03 (0.65; 1.63) | 1.23 (0.77; 1.99) |
| 1.01 (0.29; 3.56) | 0.53 (0.09; 3.26) | 1.01 (0.65; 1.58) | Palanosetron | 1.22 (0.54; 2.79) |
| 1.24 (0.35; 4.38) | 0.65 (0.10; 4.11) | 1.24 (0.79; 1.96) | 1.23 (0.69; 2.18) | Ramosetron |

**P-score of “Acute rescue medicine”**

Granisetron 0.7262

Ramosetron 0.6336

Azasetron 0.4103

Palanosetron 0.3796

Ondansetron 0.3502

**Forest diagram of “Acute rescue medicine”**


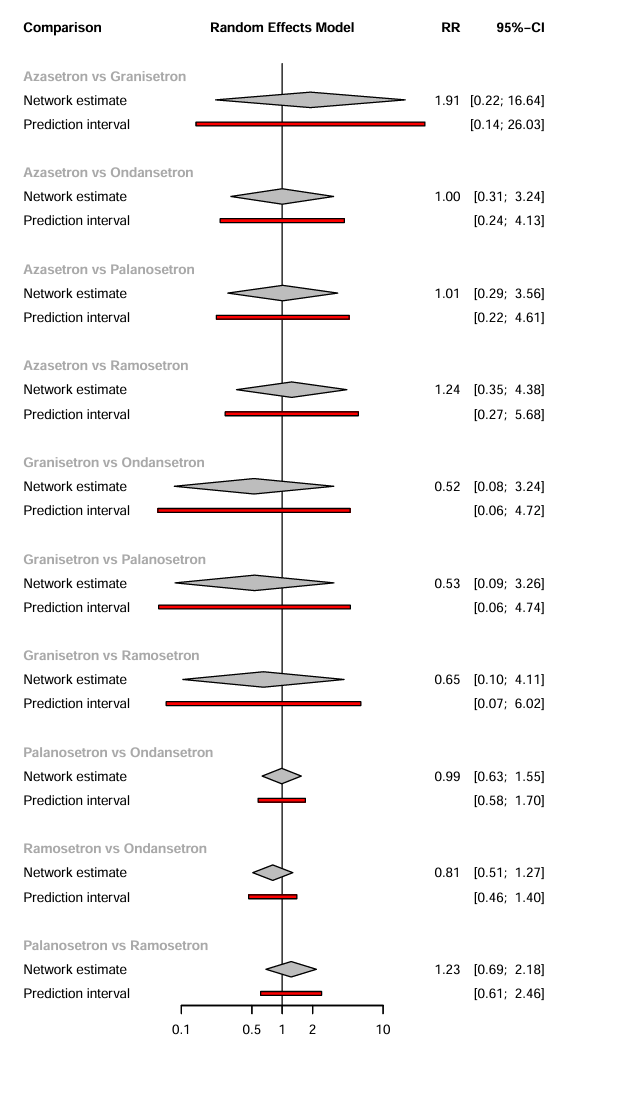


**Network calculation of “Late rescue medicine”**

| Azasetron | . | 1.00 (0.31; 3.26) | . | . |
| --- | --- | --- | --- | --- |
| 3.54 (0.60; 20.84) | Granisetron | 0.25 (0.06; 1.09) | 1.06 (0.16; 7.00) | 1.00 (0.02; 49.15) |
| 1.00 (0.31; 3.26) | 0.28 (0.08; 1.06) | Ondansetron | 1.82 (1.22;2.71) | 1.14 (0.59; 2.19) |
| 1.77 (0.51; 6.17) | 0.50 (0.13; 1.94) | 1.77 (1.20; 2.63) | Palanosetron | 3.00 (0.13; 71.42) |
| 1.21 (0.32; 4.65) | 0.34 (0.08; 1.47) | 1.21 (0.64; 2.30) | 0.68 (0.32; 1.44) | Ramosetron |

**P-score of “Late rescue medicine”**

Granisetron 0.9136

Palanosetron 0.7037

Ramosetron 0.3919

Azasetron 0.2885

Ondansetron 0.2023

**Forest diagram of “Late rescue medicine”**


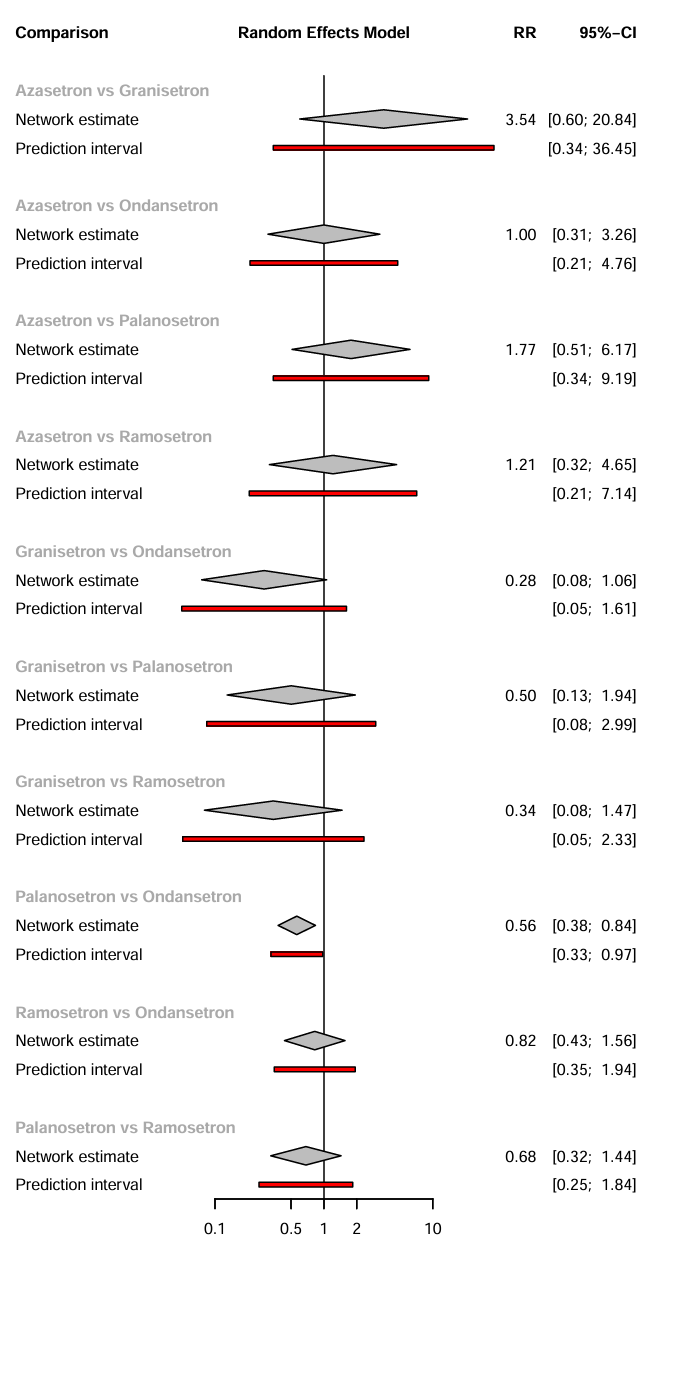


**Network calculation of “>24h rescue medicine”**

| Azasetron | . | 3.00 (0.13; 71.89) | . | . |
| --- | --- | --- | --- | --- |
| 11.30 (0.09; 1385.10) | Granisetron | . | 1.00 (0.02; 49.04) | 1.00 (0.02; 49.04) |
| 3.00 (0.13; 71.89) | 0.27 (0.01; 9.82) | Ondansetron | 2.40 (1.03; 5.57) | 9.00 (0.50; 163.58) |
| 7.48 (0.28; 199.40) | 0.66 (0.02; 23.65) | 2.49 (1.09; 5.72) | Palanosetron | 1.00 (0.02; 49.04) |
| 17.07 (0.33; 884.97) | 1.51 (0.04; 53.99) | 5.69 (0.55; 59.35) | 2.28 (0.21; 24.82) | Ramosetron |

**P-score of “>24h rescue medicine”**

Ramosetron 0.7970

Granisetron 0.6507

Palanosetron 0.6322

Ondansetron 0.2689

Azasetron 0.1512

**Forest diagram of “>24h rescue medicine”**


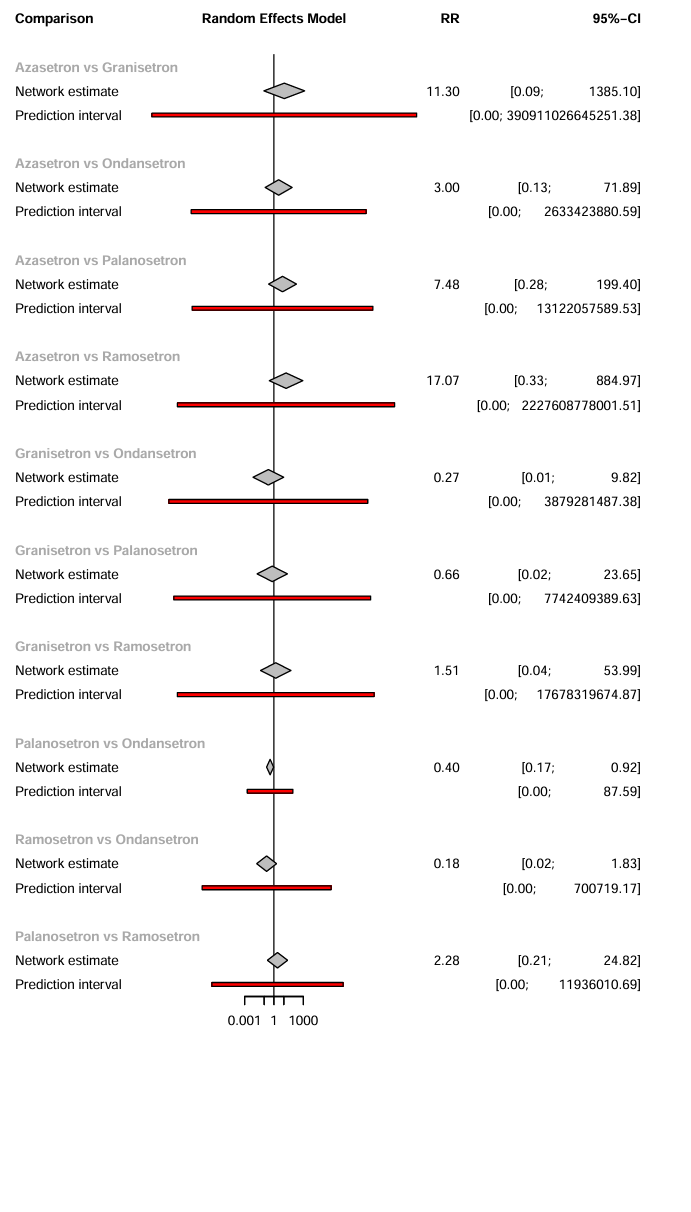


**Network calculation of “Overall rescue medicine”**

| Granisetron | 0.28 (0.07; 1.11) | 2.00 (0.17; 23.52) | . | . |
| --- | --- | --- | --- | --- |
| 0.34 (0.09; 1.30) | Ondansetron | 1.15 (0.62; 2.13) | 2.00 (0.69; 5.84) | 1.24 (0.50; 3.08) |
| 0.44 (0.10; 1.85) | 1.29 (0.73; 2.30) | Palanosetron | 0.70 (0.22; 2.24) | . |
| 0.47 (0.10; 2.26) | 1.39 (0.61; 3.19) | 1.07 (0.46;2.50) | Ramosetron | . |
| 0.42 (0.08; 2.12) | 1.24 (0.50; 3.08) | 0.96 (0.33; 2.81) | 0.89 (0.26; 3.05) | Tropisetron |

**P-score of “Overall rescue medicine”**

Granisetron 0.8741

Ramosetron 0.5233

Palanosetron 0.4767

Tropisetron 0.4284

Ondansetron 0.1976

**Forest diagram of “Overall rescue medicine”**


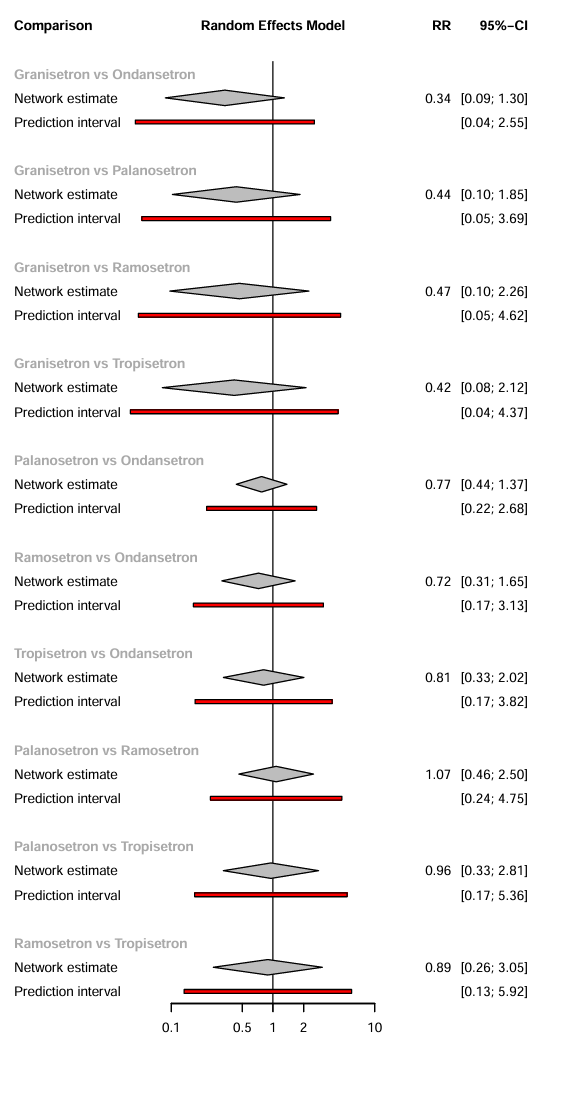


**Network calculation of “Adverse reaction”**

| Azasetron | . | 0.92 (0.40; 2.12) | . | . | . |
| --- | --- | --- | --- | --- | --- |
| 0.76 (0.25; 2.33) | Granisetron | 1.08 (0.51; 2.31) | 5.00 (0.59; 42.14) | . | . |
| 0.92 (0.40; 2.12) | 1.20 (0.57; 2.52) | Ondansetron | 1.05 (0.80; 1.37) | 1.03 (0.69; 1.54) | 1.14 (0.46; 2.85) |
| 0.98 (0.41; 2.34) | 1.28 (0.59; 2.78) | 1.06 (0.82; 1.37) | Palanosetron | 0.97 (0.60;1.56) | . |
| 0.92 (0.37; 2.27) | 1.20 (0.53; 2.71) | 1.00 (0.71; 1.42) | 0.94 (0.65; 1.35) | Ramosetron | . |
| 1.05 (0.30; 3.62) | 1.37 (0.42; 4.45) | 1.14 (0.46; 2.85) | 1.07 (0.42; 2.77) | 1.14 (0.43; 3.04) | Tropisetron |

**P-score of “Adverse reaction”**

Tropisetron 0.6017

Palanosetron 0.5933

Azasetron 0.5662

Ramosetron 0.4721

Ondansetron 0.4621

Granisetron 0.3045

**Forest diagram of “Adverse reaction”**


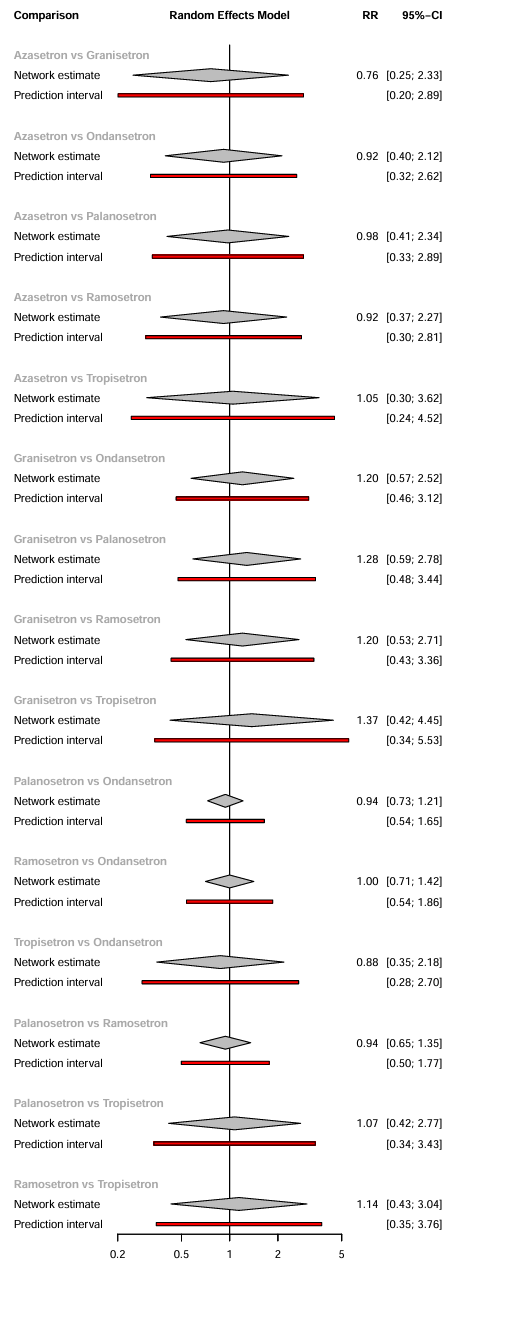

Supplement: Supplementary file 9 — Data S9. [file IJGO-171-177-s002.docx]
