# Supplementary material for: 5‐HT3 receptor antagonists for preventing postoperative nausea and vomiting after gynecological surgery: A systematic review and network meta‐analysis
Source: Int J Gynaecol Obstet. 2025 May 9;171(1):177–89. doi: 10.1002/ijgo.70197 (PMC12447676; doi:10.1002/ijgo.70197)

**Data S10 Sensitivity of exclusing propofol**

**Network calculation of “Acute nausea”**

| Azasetron | . | 0.82 (0.37; 1.80) | . | . |
| --- | --- | --- | --- | --- |
| 1.40 (0.41; 4.73) | Granisetron | 0.20 (0.01; 4.00) | 0.84 (0.30; 2.41) | 0.83 (0.28; 2.48) |
| 0.82 (0.37; 1.80) | 0.59 (0.23; 1.49) | Ondansetron | 1.47 (1.13;1.92) | 1.14 (0.89; 1.46) |
| 1.21 (0.53; 2.75) | 0.86 (0.34; 2.18) | 1.48 (1.16; 1.87) | Palanosetron | 0.82 (0.63; 1.09) |
| 0.93 (0.41; 2.11) | 0.67 (0.26; 1.68) | 1.14 (0.91; 1.43) | 0.77 (0.60; 0.99) | Ramosetron |

**P-score of “Acute nausea”**

Palanosetron 0.7578

Granisetron 0.7502

Azasetron 0.4704

Ramosetron 0.3790

Ondansetron 0.1426

**Forest diagram of “Acute nausea”**


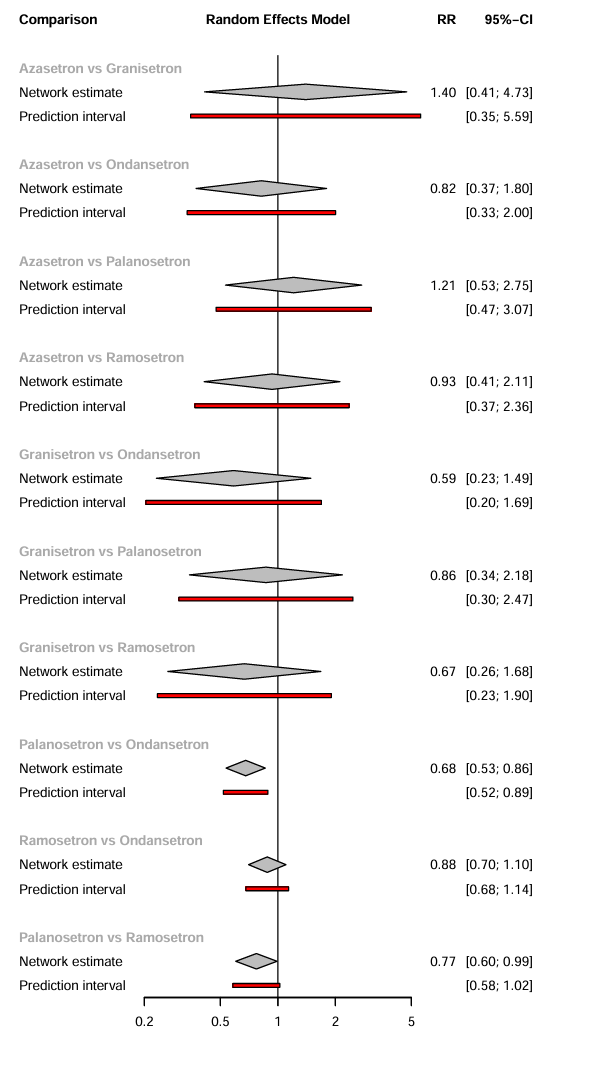


**Network calculation of “Late nausea”**

| Azasetron | . | 0.88 (0.41; 1.87) | . | . |
| --- | --- | --- | --- | --- |
| 1.28 (0.40; 4.11) | Granisetron | 0.41 (0.12; 1.44) | 1.71 (0.49; 5.95) | 1.00 (0.25; 4.00) |
| 0.88 (0.41; 1.87) | 0.68 (0.28; 1.66) | Ondansetron | 1.49 (1.04; 2.13) | 0.85 (0.52; 1.40) |
| 1.17 (0.51; 2.67) | 0.91 (0.37; 2.24) | 1.33 (0.96; 1.85) | Palanosetron | 1.12 (0.61; 2.07) |
| 0.96 (0.40; 2.26) | 0.75 (0.30; 1.86) | 1.09 (0.73; 1.64) | 0.82 (0.53; 1.27) | Ramosetron |

**P-score of “Late nausea”**

Palanosetron 0.7080

Granisetron 0.6938

Azasetron 0.4680

Ramosetron 0.3952

Ondansetron 0.2349

**Forest diagram of “Late nausea”**


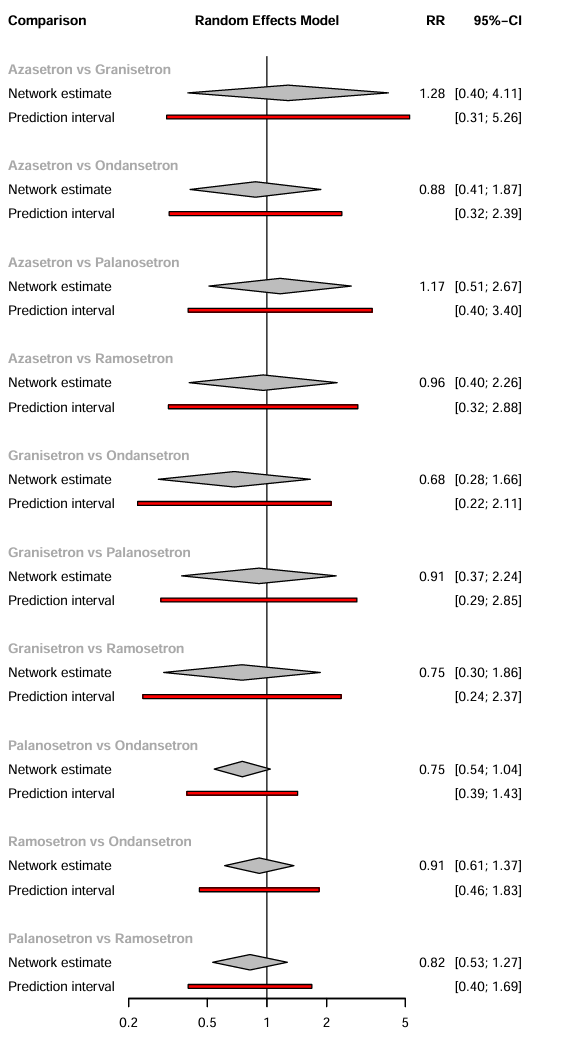


**Network calculation of “>24h nausea”**

| Azasetron | . | 1.50 (0.50; 4.47) | . | . |
| --- | --- | --- | --- | --- |
| 4.00 (0.30; 53.56) | Granisetron | . | 0.50 (0.04; 5.59) | 1.00 (0.06; 16.17) |
| 1.50 (0.50; 4.47) | 0.37 (0.04; 3.94) | Ondansetron | 1.21 (0.64; 2.28) | 6.37 (1.40; 29.04) |
| 2.17 (0.63; 7.54) | 0.54 (0.06; 5.35) | 1.45 (0.80; 2.63) | Palanosetron | 1.16 (0.51; 2.62) |
| 3.40 (0.85; 13.59) | 0.85 (0.08; 8.68) | 2.27 (0.97;5.31) | 1.56 (0.75; 3.24) | Ramosetron |

**P-score of “>24h nausea”**

Ramosetron 0.8145

Granisetron 0.7251

Palanosetron 0.5486

Ondansetron 0.2784

Azasetron 0.1333

**Forest diagram of “>24h nausea”**


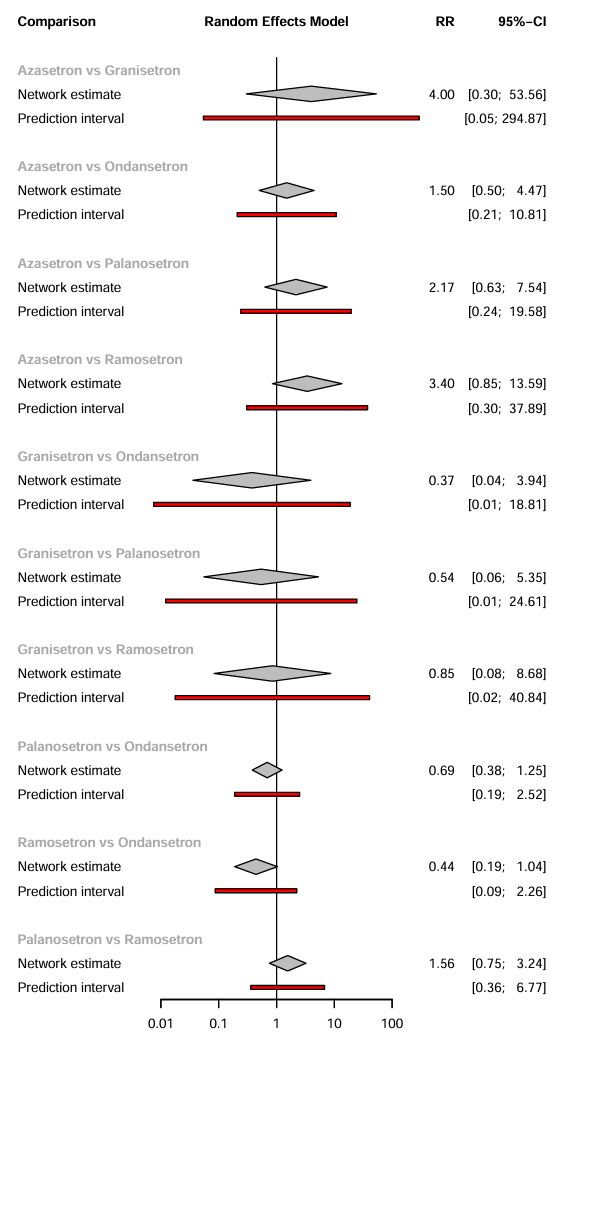


**Network calculation of “Overall nausea”**

| Azasetron | . | 0.84 (0.45; 1.58) | . | . | . |
| --- | --- | --- | --- | --- | --- |
| 2.09 (0.63; 6.89) | Granisetron | 0.36 (0.13; 1.00) | 3.00 (0.31; 28.61) | . | . |
| 0.84 (0.45; 1.58) | 0.40 (0.15; 1.10) | Ondansetron | 1.56 (1.08; 2.26) | 1.05 (0.66;1.67) | 1.22 (0.68; 2.19) |
| 1.21 (0.60; 2.48) | 0.58 (0.20; 1.67) | 1.45 (1.04; 2.01) | Palanosetron | 0.97 (0.57; 1.66) | . |
| 1.00 (0.48; 2.09) | 0.48 (0.16; 1.40) | 1.19 (0.82; 1.74) | 0.82 (0.55; 1.23) | Ramosetron | . |
| 1.02 (0.43; 2.43) | 0.49 (0.15; 1.58) | 1.22 (0.68; 2.19) | 0.84 (0.43; 1.65) | 1.02 (0.51; 2.06) | Tropisetron |

**P-score of “Overall nausea”**

Granisetron 0.8972

Palanosetron 0.6735

Tropisetron 0.4443

Azasetron 0.4184

Ramosetron 0.4102

Ondansetron 0.1564

**Forest diagram of “Overall nausea”**


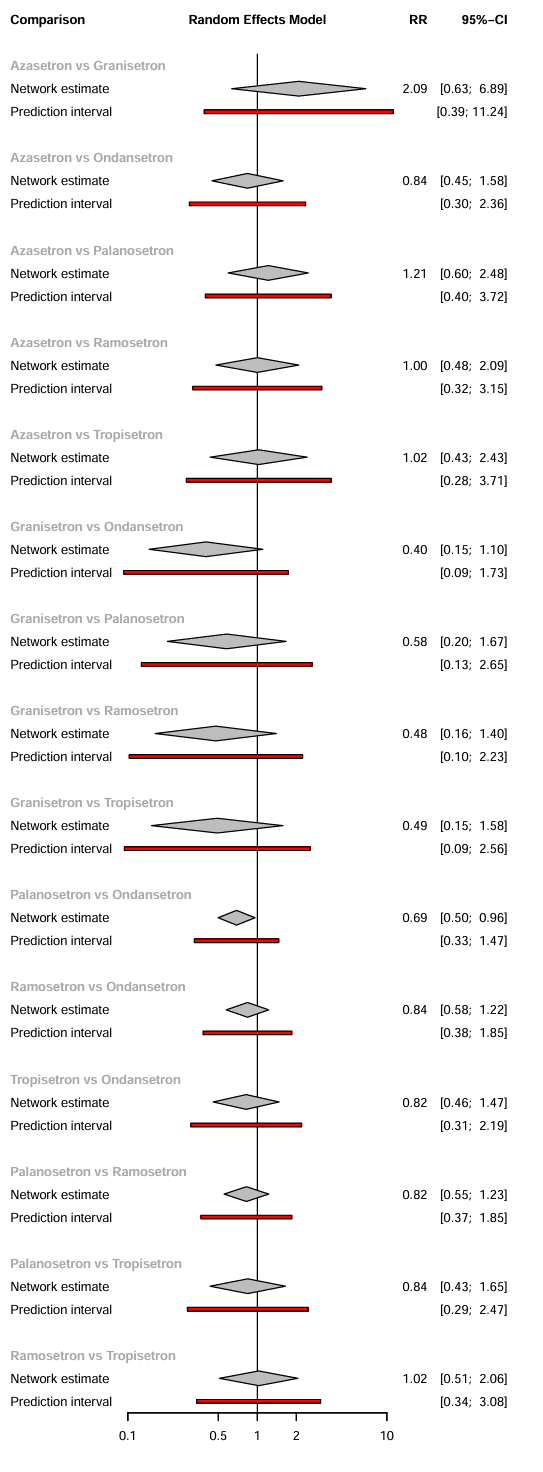


**Network calculation of “Acute vomiting”**

| Azasetron | . | 0.20 (0.01; 4.06) | . | . |
| --- | --- | --- | --- | --- |
| 0.74 (0.02; 29.92) | Granisetron | 0.33 (0.01; 7.87) | 0.52 (0.04; 6.02) | 0.33 (0.01; 7.91) |
| 0.20 (0.01; 4.06) | 0.27 (0.03; 2.33) | Ondansetron | 1.45 (0.73;2.88) | 1.63 (0.99; 2.69) |
| 0.39 (0.02; 8.47) | 0.53 (0.06; 4.62) | 1.97 (1.09; 3.57) | Palanosetron | 0.51 (0.24; 1.08) |
| 0.28 (0.01; 5.81) | 0.37 (0.04;3.24) | 1.38 (0.86;2.21) | 0.70 (0.38; 1.31) | Ramosetron |

**P-score of “Acute vomiting”**

Azasetron 0.7341

Granisetron 0.7124

Palanosetron 0.6038

Ramosetron 0.3580

Ondansetron 0.0916

**Forest diagram of “Acute vomiting”**


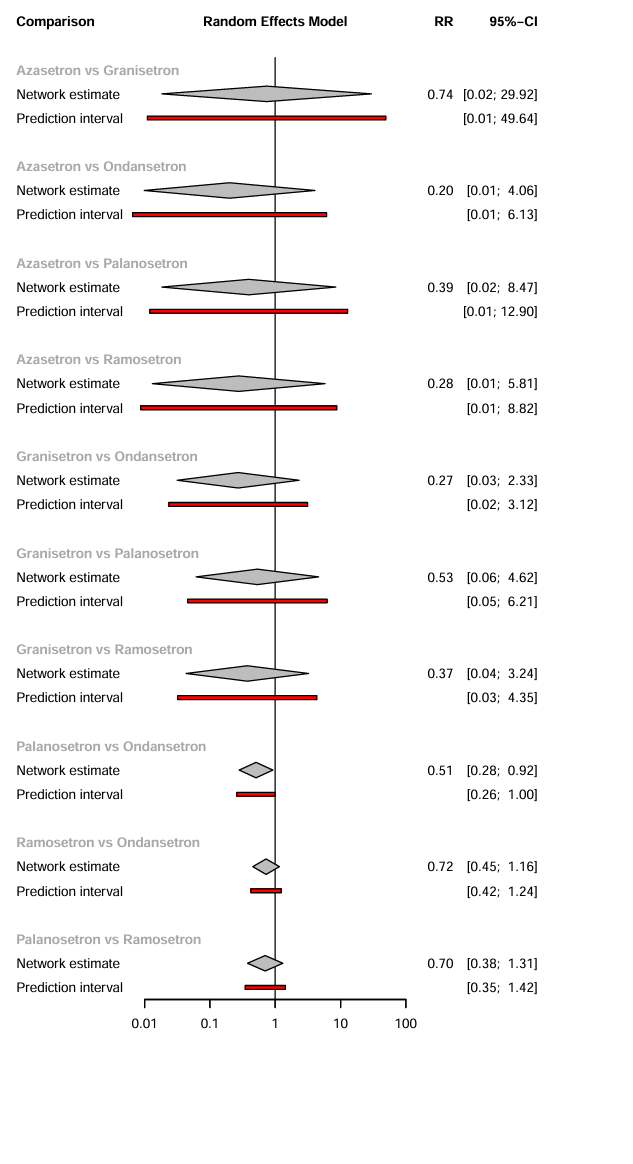


**Network calculation of “Late vomiting”**

| Azasetron | . | 2.00 (0.19; 21.34) | . | . |
| --- | --- | --- | --- | --- |
| 6.99 (0.49; 100.19) | Granisetron | 0.26 (0.07; 0.99) | 1.06 (0.16; 6.98) | 1.00 (0.02; 49.04) |
| 2.00 (0.19; 21.34) | 0.29 (0.08; 0.97) | Ondansetron | 1.96 (1.18; 3.25) | 1.20 (0.62; 2.34) |
| 3.70 (0.33; 41.36) | 0.53 (0.15; 1.89) | 1.85 (1.16; 2.96) | Palanosetron | 0.96 (0.30; 3.07) |
| 2.64 (0.23; 30.32) | 0.38 (0.10; 1.44) | 1.32 (0.74; 2.38) | 0.71 (0.36; 1.41) | Ramosetron |

**P-score of “Late vomiting”**

Granisetron 0.9155

Palanosetron 0.7121

Ramosetron 0.4624

Ondansetron 0.2299

Azasetron 0.1801

**Forest diagram of “Late vomiting”**


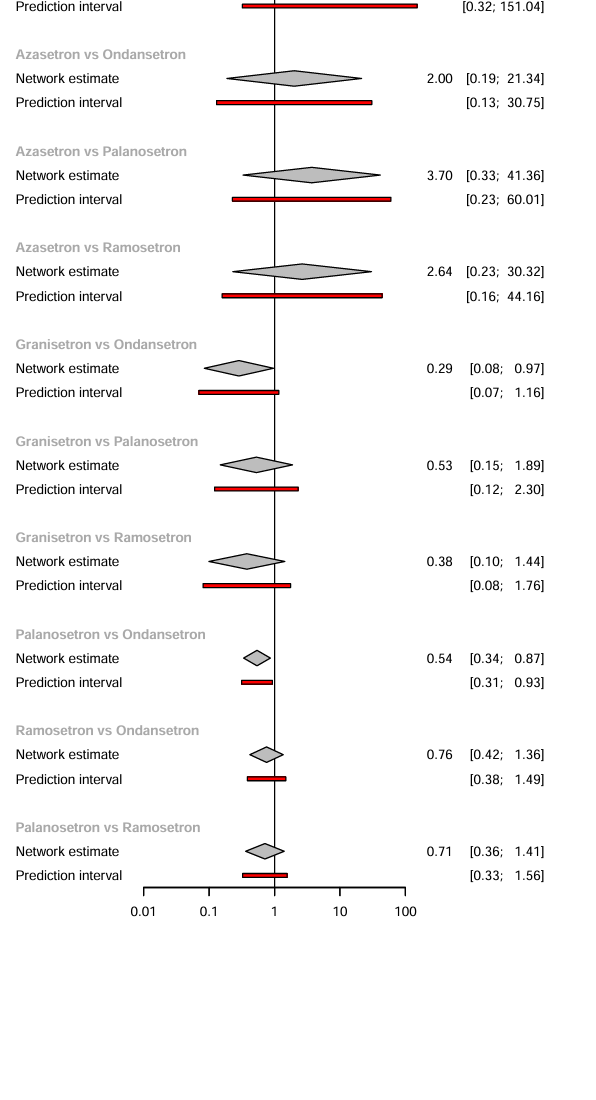


**Network calculation of “>24h vomiting”**

| Azasetron | . | 3.00 (0.13; 71.89) | . | . |
| --- | --- | --- | --- | --- |
| 9.83 (0.08; 1144.68) | Granisetron | . | 1.00 (0.02; 49.04) | 1.00 (0.02; 49.04) |
| 3.00 (0.13; 71.89) | 0.31 (0.01; 10.54) | Ondansetron | 3.93 (0.44; 35.00) | 2.34 (0.82; 6.69) |
| 14.42 (0.40; 523.14) | 1.47 (0.05; 47.47) | 4.81 (0.90; 25.66) | Palanosetron | 0.37 (0.03; 3.96) |
| 6.70 (0.24; 187.14) | 0.68 (0.02; 22.04) | 2.23 (0.82; 6.07) | 0.46 (0.08; 2.54) | Ramosetron |

**P-score of “>24h vomiting”**

Palanosetron 0.8229

Granisetron 0.6428

Ramosetron 0.6034

Ondansetron 0.2744

Azasetron 0.1566

**Forest diagram of “>24h vomiting”**


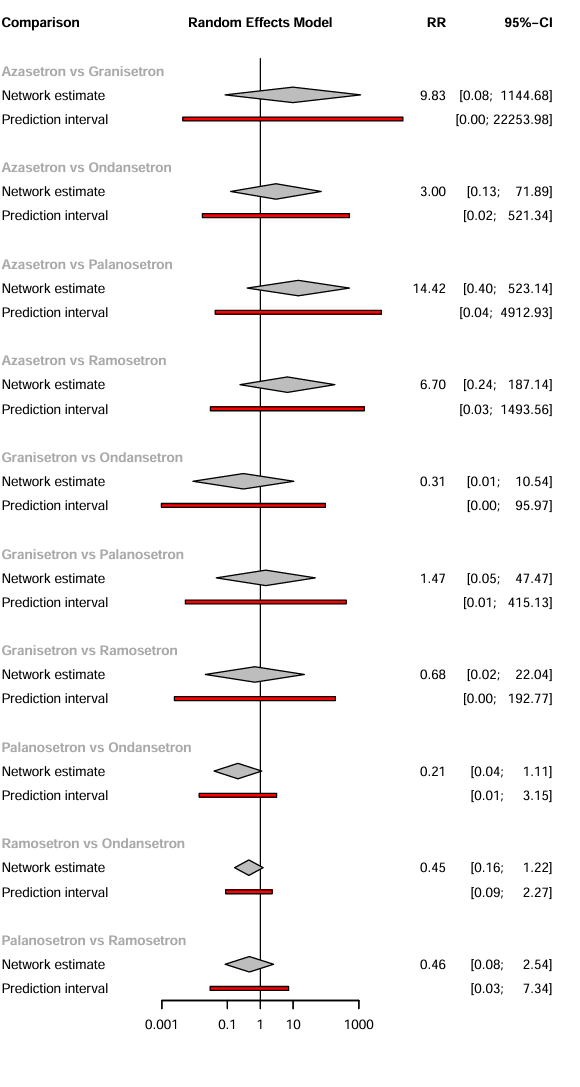


**Network calculation of “Overall vomiting”**

| Azasetron | . | 0.62 (0.17; 2.31) | . | . |
| --- | --- | --- | --- | --- |
| 2.31 (0.36; 14.84) | Granisetron | 0.23 (0.06;0.88) | 2.00 (0.17; 23.76) | . |
| 0.62 (0.17; 2.31) | 0.27 (0.07; 1.01) | Ondansetron | 1.42 (0.84; 2.41) | 0.89 (0.40; 2.01) |
| 0.94 (0.23; 3.80) | 0.41 (0.10; 1.64) | 1.50 (0.92; 2.46) | Palanosetron | 0.41 (0.14; 1.25) |
| 0.49 (0.11; 2.15) | 0.21 (0.05; 0.93) | 0.79 (0.40; 1.55) | 0.52 (0.25; 1.09) | Ramosetron |

**P-score of “Overall vomiting”**

Granisetron 0.9157

Palanosetron 0.6185

Azasetron 0.5775

Ondansetron 0.2688

Ramosetron 0.1196

**Forest diagram of “Overall vomiting”**


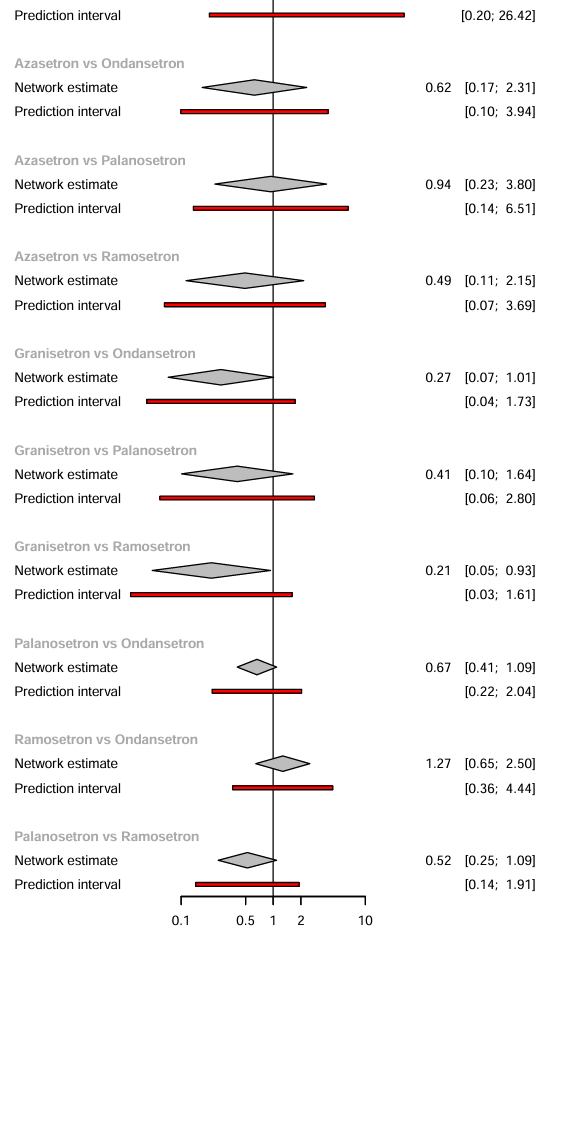


**Network calculation of “Acute PONV”**

| Dolasetron | 1.48 (0.70; 3.11) | 1.29 (0.63; 2.64) | . | . |
| --- | --- | --- | --- | --- |
| 1.50 (0.76; 2.97) | Granisetron | 0.85 (0.48; 1.48) | 1.00 (0.02; 50.24) | . |
| 1.28 (0.66; 2.49) | 0.85 (0.49; 1.49) | Ondansetron | 1.44 (0.92;2.26) | 0.89 (0.54; 1.48) |
| 1.61 (0.75; 3.47) | 1.07 (0.55; 2.11) | 1.26 (0.86; 1.85) | Palanosetron | 0.99 (0.59; 1.66) |
| 1.34 (0.61; 2.92) | 0.89 (0.45; 1.78) | 1.05 (0.70; 1.57) | 0.83 (0.55; 1.26) | Ramosetron |

**P-score of “Acute PONV”**

Palanosetron 0.7904

Granisetron 0.6574

Ramosetron 0.4813

Ondansetron 0.3950

Dolasetron 0.1759

**Forest diagram of “Acute PONV”**


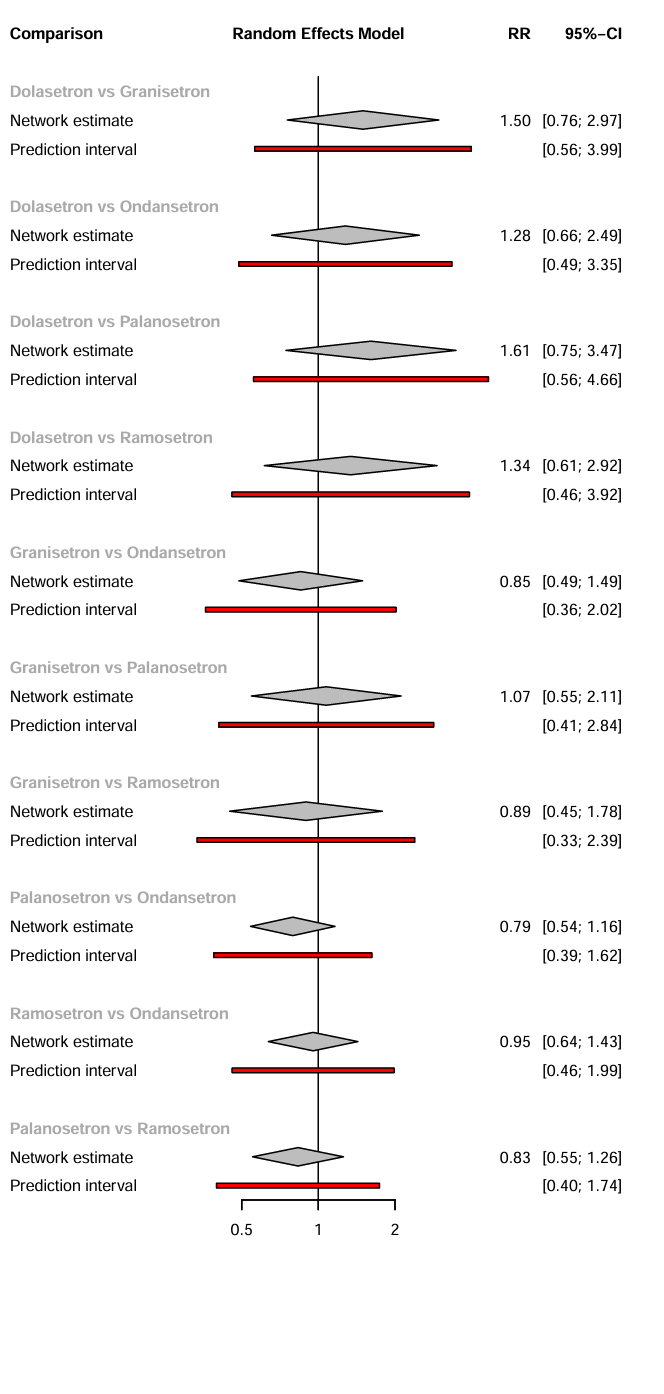


**Network calculation of “Late PONV”**

| Dolasetron | 1.06 (0.53; 2.13) | 0.94 (0.49; 1.84) | . | . |
| --- | --- | --- | --- | --- |
| 1.11 (0.57; 2.15) | Granisetron | 0.78 (0.44; 1.37) | 2.50 (0.51; 12.35) | . |
| 0.91 (0.48; 1.73) | 0.82 (0.47; 1.44) | Ondansetron | 1.44 (1.07; 1.93) | 0.85 (0.45; 1.62) |
| 1.23 (0.61; 2.45) | 1.11 (0.60; 2.05) | 1.34 (1.02; 1.77) | Palanosetron | 0.97 (0.66; 1.43) |
| 1.06 (0.50; 2.24) | 0.96 (0.49; 1.88) | 1.16 (0.79; 1.72) | 0.87 (0.62; 1.22) | Ramosetron |

**P-score of “Late PONV”**

Palanosetron 0.7811

Granisetron 0.5752

Ramosetron 0.4975

Dolasetron 0.4275

Ondansetron 0.2188

**Forest diagram of “Late PONV”**


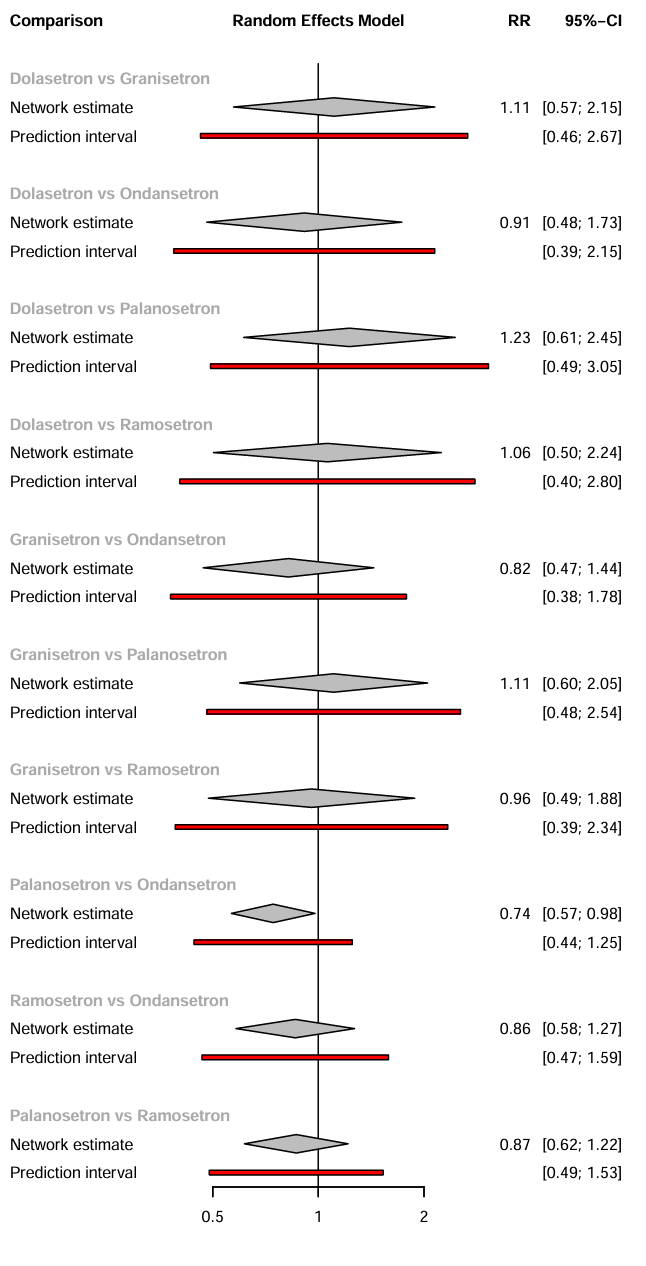


**Network calculation of “>24h PONV”**

| Ondansetron | 1.28 (0.70; 2.34) | 8.00 (0.98; 65.21) |
| --- | --- | --- |
| 1.46 (0.82; 2.62) | Palanosetron | 0.97 (0.57; 1.63) |
| 1.57 (0.74; 3.31) | 1.07 (0.64; 1.78) | Ramosetron |

**P-score of “>24h PONV”**

Ramosetron 0.7423

Palanosetron 0.6486

Ondansetron 0.1091

**Forest diagram of “>24h PONV”**


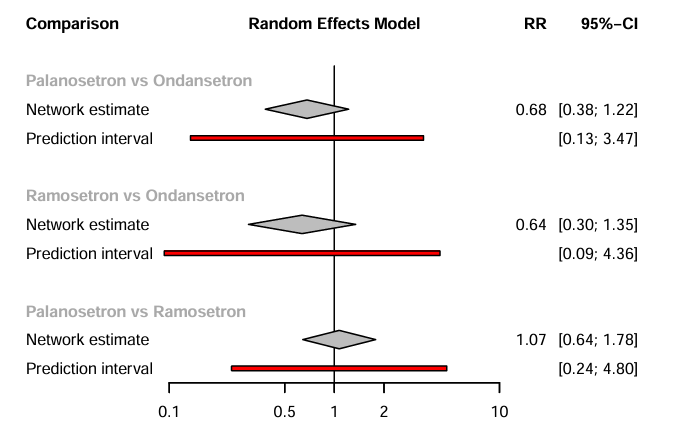


**Network calculation of “Overall PONV”**

| Azasetron | . | . | 0.75 (0.45; 1.25) | . | . |
| --- | --- | --- | --- | --- | --- |
| 0.65 (0.32; 1.33) | Dolasetron | 1.25 (0.73; 2.16) | 1.33 (0.77; 2.30) | . | . |
| 0.93 (0.49; 1.77) | 1.44 (0.87; 2.37) | Granisetron | 0.79 (0.49; 1.26) | 0.96 (0.49; 1.87) | 1.22 (0.53; 2.81) |
| 0.75 (0.45; 1.25) | 1.16 (0.70; 1.91) | 0.80 (0.54; 1.18) | Ondansetron | 1.36 (1.03; 1.78) | 1.02 (0.75; 1.38) |
| 0.94 (0.54; 1.65) | 1.45 (0.84; 2.49) | 1.01 (0.66; 1.53) | 1.25 (0.99; 1.59) | Palanosetron | 1.04 (0.75; 1.44) |
| 0.86 (0.49; 1.52) | 1.33 (0.77; 2.30) | 0.92 (0.60; 1.43) | 1.15 (0.89; 1.48) | 0.92 (0.70; 1.20) | Ramosetron |

**P-score of “Overall PONV”**

Azasetron 0.7223

Palanosetron 0.7082

Granisetron 0.6675

Ramosetron 0.5270

Ondansetron 0.2302

Dolasetron 0.1448

**Forest diagram of “Overall PONV”**


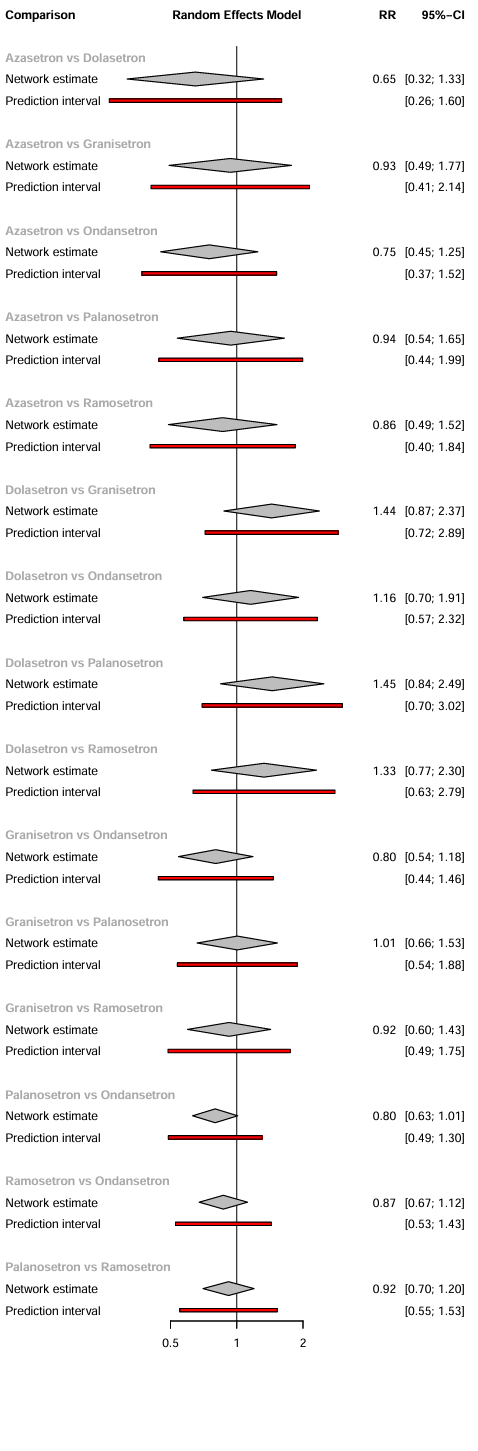


**Network calculation of “Acute rescue medicine”**

| Azasetron | . | 1.00 (0.31; 3.24) | . | . |
| --- | --- | --- | --- | --- |
| 1.90 (0.22; 16.58) | Granisetron | 0.33 (0.01; 7.87) | 0.60 (0.08; 4.51) | 1.00 (0.07; 15.36) |
| 1.00 (0.31; 3.24) | 0.53 (0.09; 3.25) | Ondansetron | 1.03 (0.65; 1.63) | 1.22 (0.77; 1.93) |
| 1.01 (0.29; 3.55) | 0.53 (0.09; 3.26) | 1.01 (0.65; 1.58) | Palanosetron | 1.22 (0.54; 2.79) |
| 1.23 (0.35; 4.31) | 0.65 (0.10; 4.07) | 1.23 (0.79; 1.92) | 1.22 (0.69; 2.15) | Ramosetron |

**P-score of “Acute rescue medicine”**

Granisetron 0.7263

Ramosetron 0.6276

Azasetron 0.4125

Palanosetron 0.3804

Ondansetron 0.3531

**Forest diagram of “Acute rescue medicine”**


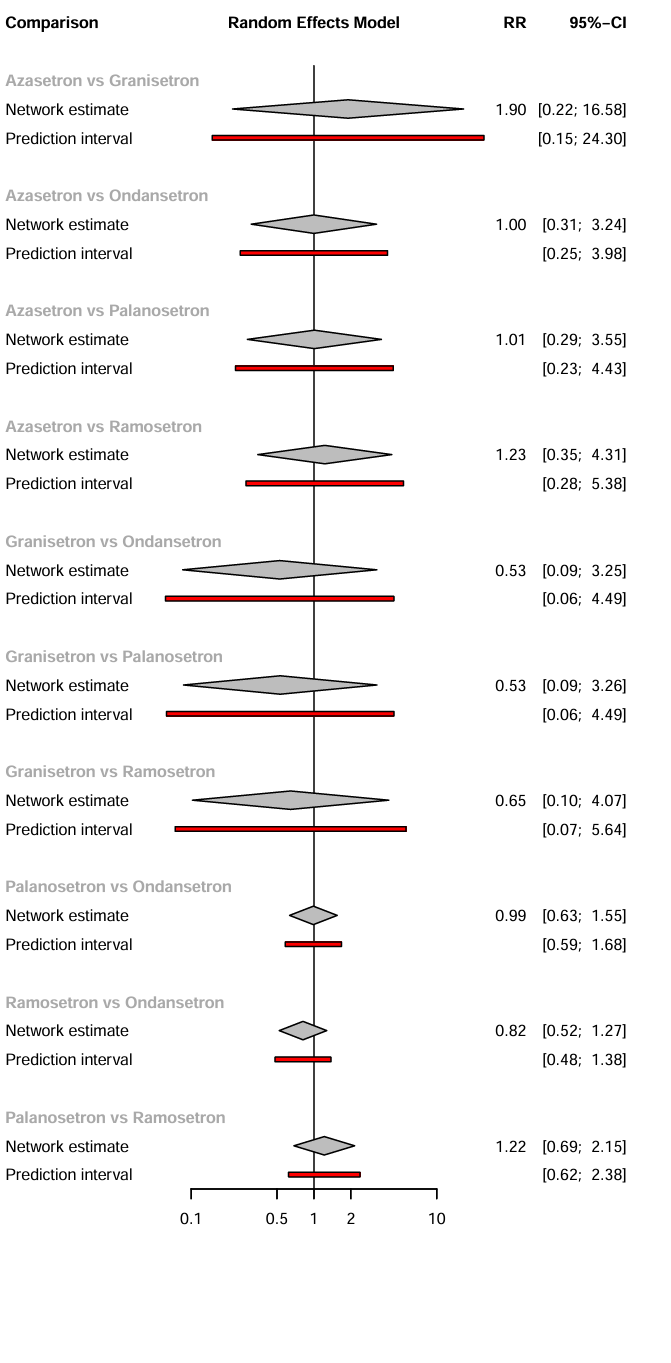


**Network calculation of “Late rescue medicine”**

| Azasetron | . | 1.00 (0.31; 3.24) | . | . |
| --- | --- | --- | --- | --- |
| 3.54 (0.61; 20.69) | Granisetron | 0.25 (0.06; 1.08) | 1.06 (0.16; 6.98) | 1.00 (0.02; 49.04) |
| 1.00 (0.31; 3.24) | 0.28 (0.08; 1.05) | Ondansetron | 1.81 (1.24; 2.66) | 1.12 (0.61; 2.07) |
| 1.77 (0.52; 6.10) | 0.50 (0.13; 1.93) | 1.77 (1.21; 2.59) | Palanosetron | 3.00 (0.13; 71.22) |
| 1.19 (0.32; 4.44) | 0.33 (0.08; 1.41) | 1.19 (0.65; 2.16) | 0.67 (0.33; 1.35) | Ramosetron |

**P-score of “Late rescue medicine”**

Granisetron 0.9163

Palanosetron 0.7110

Ramosetron 0.3769

Azasetron 0.2904

Ondansetron 0.2054

**Forest diagram of “Late rescue medicine”**


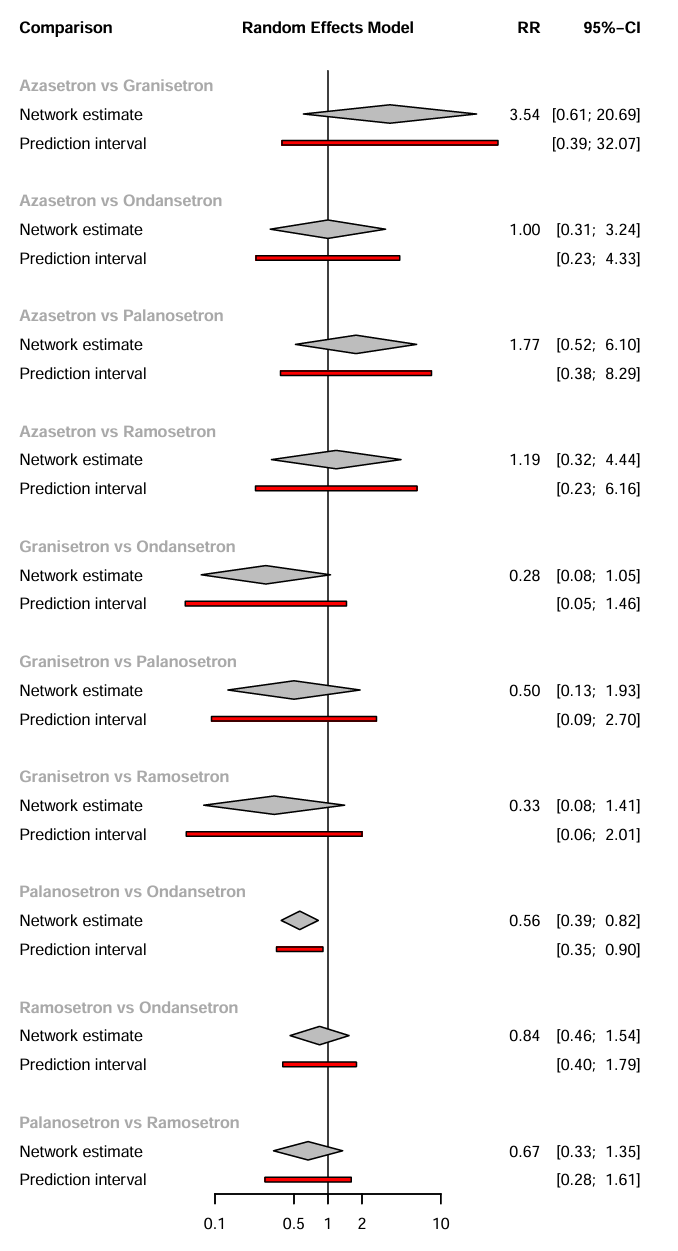


**Network calculation of “>24h rescue medicine”**

| Azasetron | . | 3.00 (0.13; 71.89) | . | . |
| --- | --- | --- | --- | --- |
| 10.23 (0.09;1151.07) | Granisetron | . | 1.00 (0.02; 49.04) | 1.00 (0.02; 49.04) |
| 3.00 (0.13; 71.89) | 0.29 (0.01; 9.68) | Ondansetron | 2.40 (1.03; 5.57) | 5.34 (0.95; 30.12) |
| 7.41 (0.28; 197.52) | 0.73 (0.02; 23.53) | 2.47 (1.08; 5.65) | Palanosetron | 1.00 (0.02; 49.04) |
| 14.10 (0.40; 491.45) | 1.38 (0.04; 44.76) | 4.70 (0.96; 22.98) | 1.90 (0.34; 10.68) | Ramosetron |

**P-score of “>24h rescue medicine”**

Ramosetron 0.8098

Granisetron 0.6467

Palanosetron 0.6322

Ondansetron 0.2602

Azasetron 0.1510

**Forest diagram of “>24h rescue medicine”**


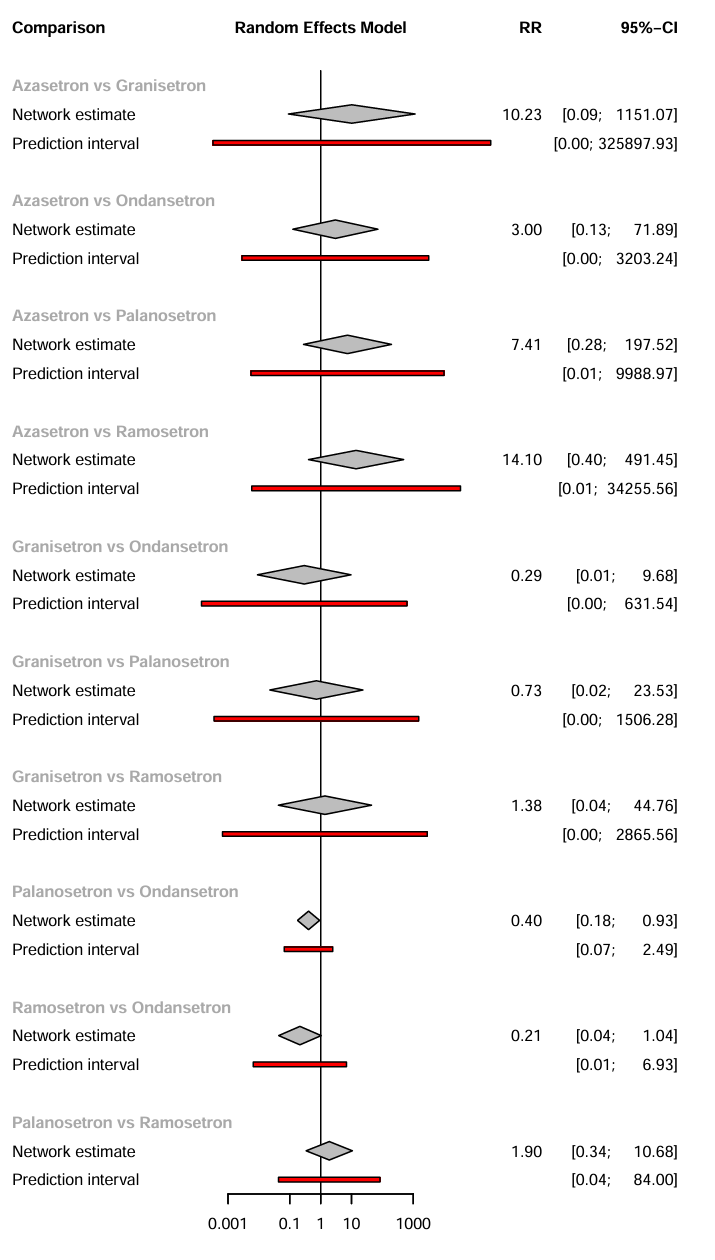


**Network calculation of “Overall rescue medicine”**

| Granisetron | 0.22 (0.04; 1.10) | 2.00 (0.17; 23.06) | . | . |
| --- | --- | --- | --- | --- |
| 0.28 (0.06; 1.36) | Ondansetron | 1.14 (0.63; 2.06) | 1.83 (0.83; 4.04) | 1.25 (0.51; 3.01) |
| 0.36 (0.07; 1.90) | 1.30 (0.75; 2.24) | Palanosetron | 0.70 (0.23; 2.14) | . |
| 0.41 (0.07; 2.25) | 1.45 (0.74; 2.83) | 1.11 (0.53; 2.34) | Ramosetron | . |
| 0.35 (0.06; 2.14) | 1.25 (0.51; 3.01) | 0.96 (0.34; 2.71) | 0.86 (0.28; 2.61) | Tropisetron |

**P-score of “Overall rescue medicine”**

Granisetron 0.8874

Ramosetron 0.5569

Palanosetron 0.4649

Tropisetron 0.4198

Ondansetron 0.1710

**Forest diagram of “Overall rescue medicine”**


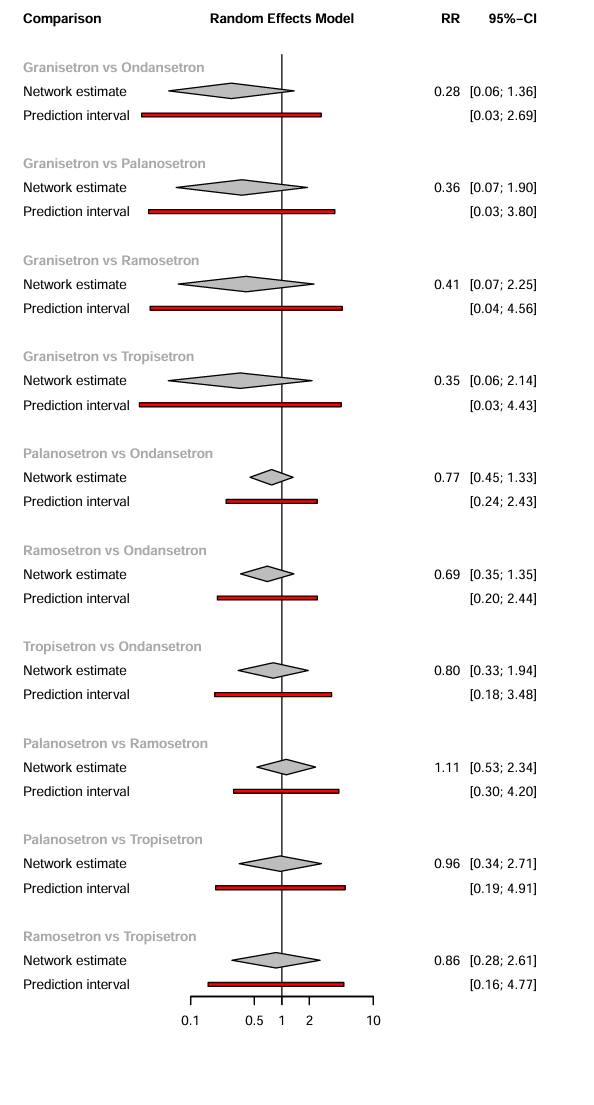


**Network calculation of “Adverse reaction”**

| Azasetron | . | 0.92 (0.42; 2.00) | . | . | . |
| --- | --- | --- | --- | --- | --- |
| 0.69 (0.23; 2.05) | Granisetron | 1.18 (0.54; 2.58) | 5.00 (0.61; 41.25) | . | . |
| 0.92 (0.42; 2.00) | 1.33 (0.62; 2.83) | Ondansetron | 0.95 (0.74; 1.22) | 1.06 (0.75; 1.50) | 1.14 (0.48; 2.71) |
| 0.90 (0.40; 2.04) | 1.31 (0.59; 2.87) | 0.99 (0.78; 1.24) | Palanosetron | 0.96 (0.63;1.47) | . |
| 0.91 (0.39; 2.10) | 1.31 (0.58; 2.96) | 0.99 (0.73; 1.34) | 1.00 (0.73; 1.38) | Ramosetron | . |
| 1.05 (0.33; 3.36) | 1.52 (0.48; 4.78) | 1.14 (0.48; 2.71) | 1.16 (0.47; 2.83) | 1.16 (0.46; 2.89) | Tropisetron |

**P-score of “Adverse reaction”**

Tropisetron 0.6322

Azasetron 0.5979

Ondansetron 0.5282

Ramosetron 0.5007

Palanosetron 0.4939

Granisetron 0.2470

**Forest diagram of “Adverse reaction”**


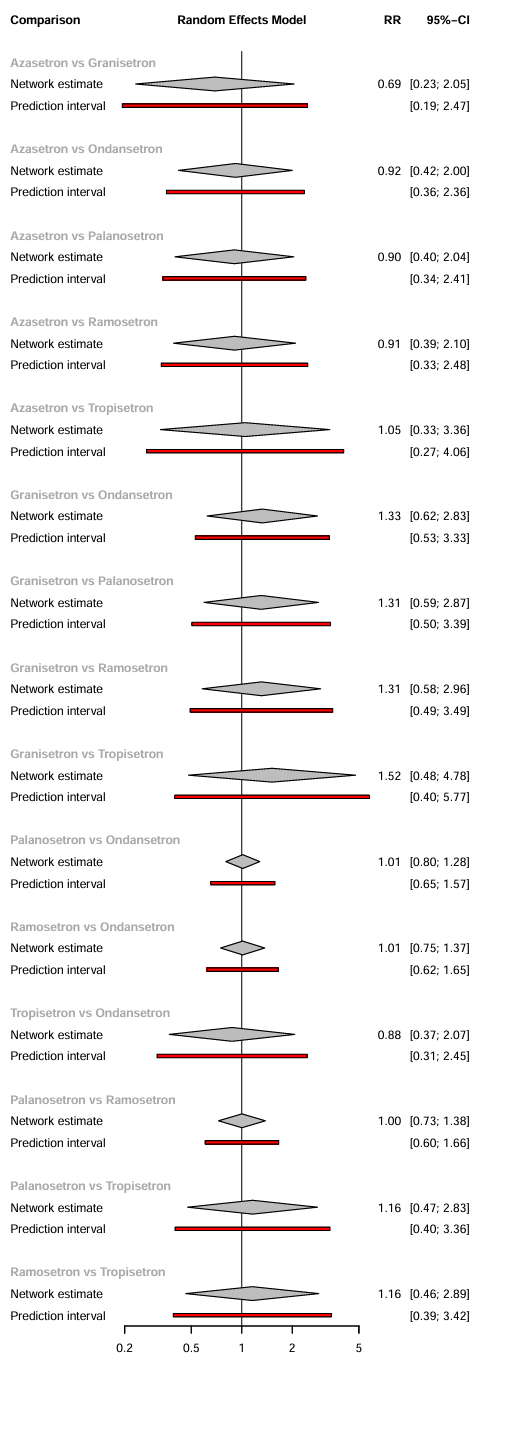

Supplement: Supplementary file 10 — Data S10. [file IJGO-171-177-s010.docx]
