# Supplementary material for: 5‐HT3 receptor antagonists for preventing postoperative nausea and vomiting after gynecological surgery: A systematic review and network meta‐analysis
Source: Int J Gynaecol Obstet. 2025 May 9;171(1):177–89. doi: 10.1002/ijgo.70197 (PMC12447676; doi:10.1002/ijgo.70197)
Supplement: Supplementary file 11 — Data S11. [file IJGO-171-177-s001.docx]

| **Section and Topic** | **Item #** | **Checklist item** | **Location where item is reported** |
| --- | --- | --- | --- |
| **TITLE** | | |  |
| Title | 1 | Identify the report as a systematic review.  “5-HT_3_receptor antagonists for preventing postoperative nausea and vomiting after gynecological surgery：a systematic review and network meta-analysis” |  |
| **ABSTRACT** | | |  |
| Abstract | 2 | See the PRISMA 2020 for Abstracts checklist.  **Title：**5-HT_3_receptor antagonists for preventing postoperative nausea and vomiting after gynecological surgery：a systematic review and network meta-analysis  **Background:** A combination of two or more antiemetic therapies targeting the different pathways of antiemetic therapy for patients at high risk for PONV was recommended. However, compliance with PONV prevention protocols remained poor. The introduction of 5-HT_3_ receptor antagonists was a major advance in the treatment of PONV. On the one hand, it is necessary to take a variety of measures to push physicians to adhere to the guidelines from the recommended combinations, on the other hand, a preferred 5-HT_3_ receptor antagonist for protecting PONV of gynecological operations might be needed to recommend. However, the efficiency between 5-HT_3_ receptor antagonists were not clear in gynecological operations.  **Objective:** To assess the effectiveness of different 5-HT_3_ antagonists in preventing postoperative nausea and vomiting (PONV) after gynecological surgery.  **Methods:** Electronic databases, including PubMed, Embase, the Cochrane Library, and Web of Science, were searched for randomized clinical trials (RCTs) to compare the efficiency of 5-HT_3_ antagonists in terms of PONV prophylaxis after gynecological surgery. Outcome measures included nausea, vomiting, PONV, rescue medicine, and adverse reaction.  **Results:** Of 1060 records identified, data were available for 21 RCT and 1959 female participants included. We observed low heterogeneity between studies in this network (Supplement 2) and no significant inconsistencies among direct and indirect comparisons (Supplement 3).  Palonosetron demonstrated superior efficacy compared to Ondansetron, with a significant difference in “Acute nausea”, “Overall nausea”, “Acute vomiting”, “Late vomiting”, “Late PONV”, “Overall PONV”， “Late rescue medicine” and “>24 hours rescue medicine”. There was a significant difference between Palonosetron and Ramosetron in “Acute nausea”, Ramosetron and Ondansetron in “>24 hours nausea”, and Granisetron and Ondansetron in “Late vomiting”. Additionally, Granisetron and Palonosetron are generally ranked higher in the P-score system.  **Conclusion:** In gynecological surgery, Palonosetron demonstrated superior efficacy to Ondansetron and Granisetron seemed the most effective alternative to Palonosetron.  **Funding:** 2021 Clinical Research Funds of Shandong Medical Association-Qilu Specialized Funding |  |
| **INTRODUCTION** | | |  |
| Rationale | 3 | Describe the rationale for the review in the context of existing knowledge.  The relative effectiveness of 5-HT_3_ receptor antagonists in gynecological surgery remained controversial. Palonosetron was often preferred over other 5-HT_3_ receptor antargonists for PONV prevention, but some studies present conflicting outcomes^12-14^. There was debate over the effectiveness of different first-generation 5-HT_3_ receptor antagonists^15-17^. A scarcity of network meta-analyses exists concerning the use of 5-HT3 receptor antagonists in gynecological surgery. |  |
| Objectives | 4 | Provide an explicit statement of the objective(s) or question(s) the review addresses.  To assess the effectiveness of different 5-HT_3_ antagonists in preventing postoperative nausea and vomiting (PONV) after gynecological surgery. |  |
| **METHODS** | | |  |
| Eligibility criteria | 5 | Specify the inclusion and exclusion criteria for the review and how studies were grouped for the syntheses.  Study Inclusion Criteria:(1) Female patients who received 5-HT_3_ antagonists to prevent nausea and vomiting following gynecologic surgical procedures were included. (2) Only RCTs were considered. (3) Only English-language literatures were included.  Study Exclusion Criteria:(1) Patients who did not undergo gynecological surgery were excluded from the study. (2) Patients who used antiemetic medications other than 5-HT_3_ antagonists were excluded from the study. (3) Non-RCTs, animal experiments, conference abstracts, protocols, reviews, letters, retrospective studies, and literature in languages other than English were excluded. |  |
| Information sources | 6 | Specify all databases, registers, websites, organisations, reference lists and other sources searched or consulted to identify studies. Specify the date when each source was last searched or consulted.  We included RCTs published in English and reported relevant outcomes. A comprehensive search was conducted across several databases, including PubMed, Embase, the Cochrane Library, and Web of Science, from their inception up to September 20, 2023. The search strategy incorporated Medical Subject Headings (MeSH) terms and free-text words, encompassing “Gynecologic Surgical Procedures”, “Postoperative Nausea and Vomiting”, “Ondansetron”, “Granisetron”, “Dolasetron”, “Tropisetron”, “Ramosetron”, “Azasetron” and “Palonosetron”. |  |
| Search strategy | 7 | Present the full search strategies for all databases, registers and websites, including any filters and limits used.  **PubMed**  ((("Gynecologic Surgical Procedures"[Mesh]) OR (((((((((((((((((((Procedures, Gynecologic Surgical[Title/Abstract]) OR (Surgical Procedure, Gynecologic[Title/Abstract])) OR (Surgery, Gynecological[Title/Abstract])) OR (Gynecological Surgeries[Title/Abstract])) OR (Gynecological Surgery[Title/Abstract])) OR (Surgeries, Gynecological[Title/Abstract])) OR (Gynecologic Surgical Procedure[Title/Abstract])) OR (Surgical Procedures, Gynecologic[Title/Abstract])) OR (Gynecological Surgical Procedure[Title/Abstract])) OR (Gynecological Surgical Procedures[Title/Abstract])) OR (Procedure, Gynecological Surgical[Title/Abstract])) OR (Procedures, Gynecological Surgical[Title/Abstract])) OR (Surgical Procedure, Gynecological[Title/Abstract])) OR (Surgical Procedures, Gynecological[Title/Abstract])) OR (Procedure, Gynecologic Surgical[Title/Abstract])) OR (Gynecologic Surgery[Title/Abstract])) OR (Gynecologic Surgeries[Title/Abstract])) OR (Surgeries, Gynecologic[Title/Abstract])) OR (Surgery, Gynecologic[Title/Abstract]))) AND (("Postoperative Nausea and Vomiting"[Mesh]) OR ((((((((((PONV[Title/Abstract]) OR (Nausea[Title/Abstract] AND Vomiting, Postoperative[Title/Abstract])) OR (Vomiting, Postoperative[Title/Abstract])) OR (Postoperative Emesis[Title/Abstract])) OR (Postoperative Vomiting[Title/Abstract])) OR (Emesis, Postoperative[Title/Abstract])) OR (Emeses, Postoperative[Title/Abstract])) OR (Postoperative Emeses[Title/Abstract])) OR (Postoperative Nausea[Title/Abstract])) OR (Nausea, Postoperative[Title/Abstract])))) AND ((((((((((((((("Ondansetron"[Mesh]) OR (((((((((((((Ondansetron, (+,-)-Isomer[Title/Abstract]) OR (Zofran ODT[Title/Abstract])) OR (ODT, Zofran[Title/Abstract])) OR (Ondansetron, (R)-Isomer[Title/Abstract])) OR (Ondansetron, (S)-Isomer[Title/Abstract])) OR (Zofran[Title/Abstract])) OR (Ondansetron Hydrochloride[Title/Abstract])) OR (Hydrochloride, Ondansetron[Title/Abstract])) OR (Ondansetron Monohydrochloride[Title/Abstract])) OR (Monohydrochloride, Ondansetron[Title/Abstract])) OR (Ondansetron Monohydrochloride Dihydrate[Title/Abstract])) OR (Dihydrate, Ondansetron Monohydrochloride[Title/Abstract])) OR (Monohydrochloride Dihydrate, Ondansetron[Title/Abstract]))) OR ("Granisetron"[Mesh])) OR (((((Kytril[Title/Abstract]) OR (Granisetron Hydrochloride[Title/Abstract])) OR (Hydrochloride, Granisetron[Title/Abstract])) OR (Granisetron Monohydrochloride[Title/Abstract])) OR (Monohydrochloride, Granisetron[Title/Abstract]))) OR ("dolasetron" [Supplementary Concept])) OR ((((dolasetron mesylate[Title/Abstract]) OR (dolasetron mesylate monohydrate[Title/Abstract])) OR (dolasetron mesilate monohydrate[Title/Abstract])) OR (Anzemet[Title/Abstract]))) OR ("Tropisetron"[Mesh])) OR (((Navoban[Title/Abstract]) OR (Indole 3 carboxylic Acid Tropine Ester[Title/Abstract])) OR (Tropisetron Hydrochloride[Title/Abstract]))) OR ("ramosetron" [Supplementary Concept])) OR ((ramosetron hydrochloride[Title/Abstract]) OR (Nasea[Title/Abstract]))) OR ("azasetron" [Supplementary Concept])) OR (azasetron, (+-)-isomer[Title/Abstract])) OR (("azasetron"[Supplementary Concept] OR "azasetron"[All Fields]) AND "isomer"[Title/Abstract])) OR ("Palonosetron"[Mesh])) OR ((((((Palonosetron, (R-(R*,R*))-isomer[Title/Abstract]) OR (Palonosetron, (3R)-[Title/Abstract])) OR (Palonosetron, (R-(R*,S*))-isomer[Title/Abstract])) OR (Aloxi[Title/Abstract])) OR (Palonosetron, (S-(R*,S*))-isomer[Title/Abstract])) OR (Palonosetron Hydrochloride[Title/Abstract]))) Filters: from 1000/1/1 - 2023/9/20  **Embase**  #5 AND #10 AND #39  #39 #11 OR #12 OR #13 OR #14 OR #15 OR #16 OR #17 OR #18 OR #19 OR #20 OR #21 OR #22 OR #23 OR #24 OR #25 OR #26 OR #27 OR #28 OR #29 OR #30 OR #31 OR #32 OR #33 OR #34 OR #35 OR #36 OR #37 OR #38  #38 (palonosetron,:ab,ti AND r-:ab,ti AND r*,r*:ab,ti AND -isomer:ab,ti OR (palonosetron,:ab,ti AND 3r:ab,ti AND -:ab,ti) OR (palonosetron,:ab,ti AND r-:ab,ti AND r*,s*:ab,ti AND -isomer:ab,ti) OR aloxi:ab,ti OR (palonosetron,:ab,ti AND s-:ab,ti AND r*,s*:ab,ti AND -isomer:ab,ti) OR 'palonosetron hydrochloride':ab,ti) AND [01-01-2023]/sd NOT [21-09-2023]/sd  #37 (palonosetron,:ab,ti AND r-:ab,ti AND r*,r*:ab,ti AND -isomer:ab,ti OR (palonosetron,:ab,ti AND 3r:ab,ti AND -:ab,ti) OR (palonosetron,:ab,ti AND r-:ab,ti AND r*,s*:ab,ti AND -isomer:ab,ti) OR aloxi:ab,ti OR (palonosetron,:ab,ti AND s-:ab,ti AND r*,s*:ab,ti AND -isomer:ab,ti) OR 'palonosetron hydrochloride':ab,ti) AND [<1966-2022]/py  #36 ('palonosetron'/exp OR 'palonosetron') AND [01-01-2023]/sd NOT [21-09-2023]/sd  #35 ('palonosetron'/exp OR 'palonosetron') AND [<1966-2022]/py  #34 azasetron,:ab,ti AND +-:ab,ti AND -isomer:ab,ti AND [01-01-2023]/sd NOT [21-09-2023]/sd  #33 azasetron,:ab,ti AND +-:ab,ti AND -isomer:ab,ti AND [<1966-2022]/py  #32 ('azasetron'/exp OR 'azasetron') AND [01-01-2023]/sd NOT [21-09-2023]/sd  #31 ('azasetron'/exp OR 'azasetron') AND [<1966-2022]/py  #30 ('ramosetron hydrochloride':ab,ti OR nasea:ab,ti) AND [01-01-2023]/sd NOT [21-09-2023]/sd  #29 ('ramosetron hydrochloride':ab,ti OR nasea:ab,ti) AND [<1966-2022]/py  #28('ramosetron'/exp OR 'ramosetron') AND [01-01-2023]/sd NOT [21-09-2023]/sd  #27 ('ramosetron'/exp OR 'ramosetron') AND [<1966-2022]/py  #26 (navoban:ab,ti OR 'indole 3 carboxylic acid tropine ester':ab,ti OR 'tropisetron hydrochloride':ab,ti) AND [01-01-2023]/sd NOT [21-09-2023]/sd  #25 (navoban:ab,ti OR 'indole 3 carboxylic acid tropine ester':ab,ti OR 'tropisetron hydrochloride':ab,ti) AND [<1966-2022]/py  #24 ('tropisetron'/exp OR 'tropisetron') AND [01-01-2023]/sd NOT [21-09-2023]/sd  #23 ('tropisetron'/exp OR 'tropisetron') AND [<1966-2022]/py  #22 ('dolasetron mesylate':ab,ti OR 'dolasetron mesylate monohydrate':ab,ti OR 'dolasetron mesilate monohydrate':ab,ti OR anzemet:ab,ti) AND [01-01-2023]/sd NOT [21-09-2023]/sd  #21 ('dolasetron mesylate':ab,ti OR 'dolasetron mesylate monohydrate':ab,ti OR 'dolasetron mesilate monohydrate':ab,ti OR anzemet:ab,ti) AND [<1966-2022]/py  #20 ('dolasetron mesilate'/exp OR 'dolasetron mesilate') AND [01-01-2023]/sd NOT [21-09-2023]/sd  #19 ('dolasetron mesilate'/exp OR 'dolasetron mesilate') AND [<1966-2022]/py  #18 (kytril:ab,ti OR 'granisetron hydrochloride':ab,ti OR 'hydrochloride, granisetron':ab,ti OR 'granisetron monohydrochloride':ab,ti OR 'monohydrochloride, granisetron':ab,ti) AND [randomized controlled trial]/lim AND [01-01-2023]/sd NOT [21-09-2023]/sd  #17 (kytril:ab,ti OR 'granisetron hydrochloride':ab,ti OR 'hydrochloride, granisetron':ab,ti OR 'granisetron monohydrochloride':ab,ti OR 'monohydrochloride, granisetron':ab,ti) AND [randomized controlled trial]/lim AND [<1966-2022]/py  #16 ('granisetron'/exp OR 'granisetron') AND [01-01-2023]/sd NOT [21-09-2023]/sd  #15 ('granisetron'/exp OR 'granisetron') AND [<1966-2022]/py  #14 (ondansetron,:ab,ti AND +,-:ab,ti AND -isomer:ab,ti OR 'zofran odt':ab,ti OR 'odt, zofran':ab,ti OR (ondansetron,:ab,ti AND r:ab,ti AND -isomer:ab,ti) OR (ondansetron,:ab,ti AND s:ab,ti AND -isomer:ab,ti) OR zofran:ab,ti OR 'ondansetron hydrochloride':ab,ti OR 'hydrochloride, ondansetron':ab,ti OR 'ondansetron monohydrochloride':ab,ti OR 'monohydrochloride, ondansetron':ab,ti OR 'ondansetron monohydrochloride dihydrate':ab,ti OR 'dihydrate, ondansetron monohydrochloride':ab,ti OR 'monohydrochloride dihydrate, ondansetron':ab,ti) AND [01-01-2023]/sd NOT [21-09-2023]/sd  #13 (ondansetron,:ab,ti AND +,-:ab,ti AND -isomer:ab,ti OR 'zofran odt':ab,ti OR 'odt, zofran':ab,ti OR (ondansetron,:ab,ti AND r:ab,ti AND -isomer:ab,ti) OR (ondansetron,:ab,ti AND s:ab,ti AND -isomer:ab,ti) OR zofran:ab,ti OR 'ondansetron hydrochloride':ab,ti OR 'hydrochloride, ondansetron':ab,ti OR 'ondansetron monohydrochloride':ab,ti OR 'monohydrochloride, ondansetron':ab,ti OR 'ondansetron monohydrochloride dihydrate':ab,ti OR 'dihydrate, ondansetron monohydrochloride':ab,ti OR 'monohydrochloride dihydrate, ondansetron':ab,ti) AND [<1966-2022]/py#12 ('ondansetron'/exp OR 'ondansetron') AND [01-01-2023]/sd NOT [21-09-2023]/sd  #11 ('ondansetron'/exp OR 'ondansetron') AND [<1966-2022]/py#10 #6 OR #7 OR #8 OR #9  #9 (ponv:ab,ti OR (nausea:ab,ti AND 'vomiting, postoperative':ab,ti) OR 'vomiting, postoperative':ab,ti OR 'postoperative emesis':ab,ti OR 'postoperative vomiting':ab,ti OR 'emesis, postoperative':ab,ti OR 'emeses, postoperative':ab,ti OR 'postoperative emeses':ab,ti OR 'postoperative nausea':ab,ti OR 'nausea, postoperative':ab,ti) AND [01-01-2023]/sd NOT [21-09-2023]/sd  #8 (ponv:ab,ti OR (nausea:ab,ti AND 'vomiting, postoperative':ab,ti) OR 'vomiting, postoperative':ab,ti OR 'postoperative emesis':ab,ti OR 'postoperative vomiting':ab,ti OR 'emesis, postoperative':ab,ti OR 'emeses, postoperative':ab,ti OR 'postoperative emeses':ab,ti OR 'postoperative nausea':ab,ti OR 'nausea, postoperative':ab,ti) AND [<1966-2022]/py  #7 ('postoperative nausea and vomiting'/exp OR 'postoperative nausea and vomiting') AND [01-01-2023]/sd NOT [21-09-2023]/sd#6 ('postoperative nausea and vomiting'/exp OR 'postoperative nausea and vomiting') AND [<1966-2022]/py  #5 #1 OR #2 OR #3 OR #4  #4 ('gynecologic surgery'/exp OR 'gynecologic surgery') AND [01-01-2023]/sd NOT [21-09-2023]/sd  #3 ('gynecologic surgery'/exp OR 'gynecologic surgery') AND [<1966-2022]/py  #2 ('procedures, gynecologic surgical':ab,ti OR 'surgical procedure, gynecologic':ab,ti OR 'surgery, gynecological':ab,ti OR 'gynecological surgeries':ab,ti OR 'gynecological surgery':ab,ti OR 'surgeries, gynecological':ab,ti OR 'gynecologic surgical procedure':ab,ti OR 'surgical procedures, gynecologic':ab,ti OR 'gynecological surgical procedure':ab,ti OR 'gynecological surgical procedures':ab,ti OR 'procedure, gynecological surgical':ab,ti OR 'procedures, gynecological surgical':ab,ti OR 'surgical procedure, gynecological':ab,ti OR 'surgical procedures, gynecological':ab,ti OR 'procedure, gynecologic surgical':ab,ti OR 'gynecologic surgery':ab,ti OR 'gynecologic surgeries':ab,ti OR 'surgeries, gynecologic':ab,ti OR 'surgery, gynecologic':ab,ti) AND [01-01-2023]/sd NOT [21-09-2023]/sd  #1 ('procedures, gynecologic surgical':ab,ti OR 'surgical procedure, gynecologic':ab,ti OR 'surgery, gynecological':ab,ti OR 'gynecological surgeries':ab,ti OR 'gynecological surgery':ab,ti OR 'surgeries, gynecological':ab,ti OR 'gynecologic surgical procedure':ab,ti OR 'surgical procedures, gynecologic':ab,ti OR 'gynecological surgical procedure':ab,ti OR 'gynecological surgical procedures':ab,ti OR 'procedure, gynecological surgical':ab,ti OR 'procedures, gynecological surgical':ab,ti OR 'surgical procedure, gynecological':ab,ti OR 'surgical procedures, gynecological':ab,ti OR 'procedure, gynecologic surgical':ab,ti OR 'gynecologic surgery':ab,ti OR 'gynecologic surgeries':ab,ti OR 'surgeries, gynecologic':ab,ti OR 'su  **Cochrane**  #1 MeSH descriptor: [Gynecologic Surgical Procedures] explode all trees  #2 (Procedures, Gynecologic Surgical):ti,ab,kw OR (Surgical Procedure, Gynecologic):ti,ab,kw OR (Surgery, Gynecological):ti,ab,kw OR (Gynecological Surgeries):ti,ab,kw OR (Gynecological Surgery):ti,ab,kw (Word variations have been searched)  #3 (Surgeries, Gynecological):ti,ab,kw OR (Gynecologic Surgical Procedure):ti,ab,kw OR (Surgical Procedures, Gynecologic):ti,ab,kw OR (Gynecological Surgical Procedure):ti,ab,kw OR (Gynecological Surgical Procedures):ti,ab,kw (Word variations have been searched)  #4 (Procedure, Gynecological Surgical):ti,ab,kw OR (Procedures, Gynecological Surgical):ti,ab,kw OR (Surgical Procedure, Gynecological):ti,ab,kw OR (Surgical Procedures, Gynecological):ti,ab,kw OR (Procedure, Gynecologic Surgical):ti,ab,kw (Word variations have been searched)  #5 (Gynecologic Surgery):ti,ab,kw OR (Gynecologic Surgeries):ti,ab,kw OR (Surgeries, Gynecologic):ti,ab,kw OR (Surgery, Gynecologic):ti,ab,kw (Word variations have been searched)  #6 #1 or #2 or #3 or #4 or #5  #7 MeSH descriptor: [Postoperative Nausea and Vomiting] explode all trees  #8 (PONV):ti,ab,kw OR (Nausea and Vomiting, Postoperative):ti,ab,kw OR (Vomiting, Postoperative):ti,ab,kw OR (Postoperative Emesis):ti,ab,kw OR (Postoperative Vomiting):ti,ab,kw (Word variations have been searched)  #9 (Emesis, Postoperative):ti,ab,kw OR (Emeses, Postoperative):ti,ab,kw OR (Postoperative Emeses):ti,ab,kw OR (Postoperative Nausea):ti,ab,kw OR (Nausea, Postoperative):ti,ab,kw (Word variations have been searched)  #10 #7 or #8 or #9  #11 MeSH descriptor: [Ondansetron] explode all trees  #12 (Zofran):ti,ab,kw OR (Ondansetron Hydrochloride):ti,ab,kw OR (Hydrochloride, Ondansetron):ti,ab,kw OR (Ondansetron Monohydrochloride):ti,ab,kw OR (Monohydrochloride, Ondansetron):ti,ab,kw (Word variations have been searched)  #13 (Ondansetron, Isomer):ti,ab,kw OR (Zofran ODT):ti,ab,kw OR (ODT, Zofran):ti,ab,kw OR (Ondansetron, (R)Isomer):ti,ab,kw OR (Ondansetron, (S)Isomer):ti,ab,kw (Word variations have been searched)  #14 (Ondansetron Monohydrochloride Dihydrate):ti,ab,kw OR (Dihydrate, Ondansetron Monohydrochloride):ti,ab,kw OR (Monohydrochloride Dihydrate, Ondansetron):ti,ab,kw (Word variations have been searched)  #15 MeSH descriptor: [Granisetron] explode all trees  #16 (Kytril):ti,ab,kw OR (Granisetron Hydrochloride):ti,ab,kw OR (Hydrochloride, Granisetron):ti,ab,kw OR (Granisetron Monohydrochloride):ti,ab,kw OR (Monohydrochloride, Granisetron):ti,ab,kw (Word variations have been searched)  #17 MeSH descriptor: [] explode all trees  #18 (dolasetron mesylate):ti,ab,kw OR (dolasetron mesylate monohydrate):ti,ab,kw OR (dolasetron mesilate monohydrate):ti,ab,kw OR (Anzemet):ti,ab,kw (Word variations have been searched)  #19 MeSH descriptor: [Tropisetron] explode all trees  #20 (Navoban):ti,ab,kw OR (Indole 3 carboxylic Acid Tropine Ester):ti,ab,kw OR (Tropisetron Hydrochloride):ti,ab,kw (Word variations have been searched)  #21 MeSH descriptor: [] explode all trees  #22 (ramosetron hydrochloride):ti,ab,kw OR (Nasea):ti,ab,kw (Word variations have been searched)  #23 MeSH descriptor: [] explode all trees  #24 (azasetron, isomer):ti,ab,kw (Word variations have been searched)  #25 MeSH descriptor: [Palonosetron] explode all trees  #26 (Palonosetron, (R-(R*,R*))isomer):ti,ab,kw OR (Palonosetron, (3R)):ti,ab,kw OR (Palonosetron, (R-(R*,S*))isomer):ti,ab,kw OR (Aloxi):ti,ab,kw OR (Palonosetron, (S-(R*,S*))isomer):ti,ab,kw (Word variations have been searched)  #27 (Palonosetron Hydrochloride):ti,ab,kw (Word variations have been searched)  #28 #11 or #12 or #13 or #14 or #15 or #16 or #17 or #18 or #19 or #20 or #21 or #22 or #23 or #24 or #25 or #26 or #27  #29 #6 and #10 and #28 with Cochrane Library publication date to Sep 2023  **Web of Science**  1: (((((((((((((((((((TS=(Gynecologic Surgical Procedures)) OR TS=(Procedures, Gynecologic Surgical)) OR TS=(Surgical Procedure, Gynecologic)) OR TS=(Surgery, Gynecological)) OR TS=(Gynecological Surgeries)) OR TS=(Gynecological Surgery)) OR TS=(Surgeries, Gynecological)) OR TS=(Gynecologic Surgical Procedure)) OR TS=(Surgical Procedures, Gynecologic)) OR TS=(Gynecological Surgical Procedure)) OR TS=(Gynecological Surgical Procedures)) OR TS=(Procedure, Gynecological Surgical)) OR TS=(Procedures, Gynecological Surgical)) OR TS=(Surgical Procedure, Gynecological)) OR TS=(Surgical Procedures, Gynecological)) OR TS=(Procedure, Gynecologic Surgical)) OR TS=(Gynecologic Surgery)) OR TS=(Gynecologic Surgeries)) OR TS=(Surgeries, Gynecologic)) OR TS=(Surgery, Gynecologic)  2: ((((((((((TS=(Postoperative Nausea and Vomiting)) OR TS=(PONV)) OR TS=(Nausea and Vomiting, Postoperative)) OR TS=(Vomiting, Postoperative)) OR TS=(Postoperative Emesis)) OR TS=(Postoperative Vomiting)) OR TS=(Emesis, Postoperative)) OR TS=(Emeses, Postoperative)) OR TS=(Postoperative Emeses)) OR TS=(Postoperative Nausea)) OR TS=(Nausea, Postoperative)  3: (((((((((((((TS=(Ondansetron)) OR TS=(Ondansetron, (+,-)-Isomer )) OR TS=(Zofran ODT)) OR TS=(ODT, Zofran)) OR TS=(Ondansetron, (R)-Isomer)) OR TS=(Ondansetron, (S)-Isomer)) OR TS=(Zofran)) OR TS=(Ondansetron Hydrochloride)) OR TS=(Hydrochloride, Ondansetron)) OR TS=(Ondansetron Monohydrochloride)) OR TS=(Monohydrochloride, Ondansetron)) OR TS=(Ondansetron Monohydrochloride Dihydrate)) OR TS=(Dihydrate, Ondansetron Monohydrochloride)) OR TS=(Monohydrochloride Dihydrate, Ondansetron)  4: (((((TS=(Granisetron)) OR TS=(Kytril)) OR TS=(Granisetron Hydrochloride)) OR TS=(Hydrochloride, Granisetron)) OR TS=(Granisetron Monohydrochloride)) OR TS=(Monohydrochloride, Granisetron)  5: ((((TS=(dolasetron)) OR TS=(dolasetron mesylate)) OR TS=(dolasetron mesylate monohydrate)) OR TS=(dolasetron mesilate monohydrate)) OR TS=(Anzemet)  6: (((TS=(Tropisetron)) OR TS=(Navoban)) OR TS=(Indole 3 carboxylic Acid Tropine Ester)) OR TS=(Tropisetron Hydrochloride)  7: ((TS=(ramosetron)) OR TS=(ramosetron hydrochloride)) OR TS=(Nasea)  8: (TS=(azasetron)) OR TS=(azasetron, (+-)-isomer )  9: (((TS=(Palonosetron)) OR TS=(Palonosetron, isomer)) OR TS=(Aloxi)) OR TS=(Palonosetron Hydrochloride)  10: #3 OR #4 OR #5 OR #6 OR #7 OR #8 OR #9  11: #10 AND #1 AND #2  12: #11 入库时间: 1900-01-01 to 2023-09-20 |  |
| Selection process | 8 | Specify the methods used to decide whether a study met the inclusion criteria of the review, including how many reviewers screened each record and each report retrieved, whether they worked independently, and if applicable, details of automation tools used in the process.  Two investigators independently assessed the study quality. When consensus could not be reached, the third author resolved the discrepancies. |  |
| Data collection process | 9 | Specify the methods used to collect data from reports, including how many reviewers collected data from each report, whether they worked independently, any processes for obtaining or confirming data from study investigators, and if applicable, details of automation tools used in the process.  Two investigators independently performed data extraction from eligible studies. When consensus could not be reached, the third author resolved the discrepancies. Generally, data extraction was performed directly from the articles. For the calculation of PONV, some data were obtained from the “complete response (CR)”.^14, 30, 33, 36^ Some data regarding the use of rescue medication were derived from the failure rates reported in the literature.^30^ |  |
| Data items | 10a | List and define all outcomes for which data were sought. Specify whether all results that were compatible with each outcome domain in each study were sought (e.g. for all measures, time points, analyses), and if not, the methods used to decide which results to collect.  Definitions of “Acute” and “Late” Periods: When the first 24 hours of post-surgery were divided into two time periods, the first was defined as the “Acute” period, and the second as the “Late” period. If the first 24 hours of post-surgery were divided into three or more parts, the time period (≥2 hours or combination should be considered if ≤2 hours) just before 2~6 hours was defined as the “Acute” period, and the time period just after 6 hours as the “Late” period.  “＞24 hours” was defined as more than 24 hours postoperatively.  “Overall” period was defined as the whole recorded time in articles.  Generally, data extraction was performed directly from the articles. For the calculation of PONV, some data were obtained from the “complete response (CR)”.^14, 30, 33, 36^ Some data regarding the use of rescue medication were derived from the failure rates reported in the literature.^30^ |  |
|  | 10b | List and define all other variables for which data were sought (e.g. participant and intervention characteristics, funding sources). Describe any assumptions made about any missing or unclear information.  Data extracted from the selected studies included the author’s name, publication year, study design, sample size, patient age, surgical methods, anesthesia techniques, postoperative analgesia approaches, interventional drugs, and the outcomes of interest. |  |
| Study risk of bias assessment | 11 | Specify the methods used to assess risk of bias in the included studies, including details of the tool(s) used, how many reviewers assessed each study and whether they worked independently, and if applicable, details of automation tools used in the process.  The risk of bias assessment of all included studies was evaluated using the Cochrane Handbook for Systematic Reviews of Interventions,^18^ which assessed key indicators including sequence generation, allocation concealment, blinding of participants and outcome assessors, and completeness or selectivity of outcome reporting. |  |
| Effect measures | 12 | Specify for each outcome the effect measure(s) (e.g. risk ratio, mean difference) used in the synthesis or presentation of results.  A random effects model was applied to pool data. Outcomes were reported as Risk Ratios (RR) with 95% credibility intervals (CI), and statistical significance was set at P values less than 0.05.  P-scores were utilized to rank the probability of effectiveness for each intervention. |  |
| Synthesis methods | 13a | Describe the processes used to decide which studies were eligible for each synthesis (e.g. tabulating the study intervention characteristics and comparing against the planned groups for each synthesis (item #5)).  The studies were eligible should included one of the Outcome Measures.  Ⅰ. Nausea: “Acute nausea”, “Late nausea”, “>24 hours nausea”, and “Overall nausea”.  Ⅱ. Vomiting: “Acute vomiting”, “Late vomiting”, “>24 hours vomiting”, and “Overall vomiting”.  Ⅲ. PONV: “Acute PONV”, “Late PONV”, “>24 hours PONV”, and “Overall PONV”.  Ⅳ. Rescue medicine: “Acute rescue medicine”, “Late rescue medicine”, “> 24 hours rescue medicine”, and “Overall rescue medicine”.  Ⅴ. Adverse reaction.  Definitions of “Acute” and “Late” Periods: When the first 24 hours of post-surgery were divided into two time periods, the first was defined as the “Acute” period, and the second as the “Late” period. If the first 24 hours of post-surgery were divided into three or more parts, the time period (≥2 hours or combination should be considered if ≤2 hours) just before 2~6 hours was defined as the “Acute” period, and the time period just after 6 hours as the “Late” period.  “＞24 hours” was defined as more than 24 hours postoperatively.  “Overall” period was defined as the whole recorded time in articles. |  |
|  | 13b | Describe any methods required to prepare the data for presentation or synthesis, such as handling of missing summary statistics, or data conversions.  Generally, data extraction was performed directly from the articles. For the calculation of PONV, some data were obtained from the “complete response (CR)”.^14, 30, 33, 36^ Some data regarding the use of rescue medication were derived from the failure rates reported in the literature.^30^ |  |
|  | 13c | Describe any methods used to tabulate or visually display results of individual studies and syntheses.  Definitions of “Acute” and “Late” Periods: When the first 24 hours of post-surgery were divided into two time periods, the first was defined as the “Acute” period, and the second as the “Late” period. If the first 24 hours of post-surgery were divided into three or more parts, the time period (≥2 hours or combination should be considered if ≤2 hours) just before 2~6 hours was defined as the “Acute” period, and the time period just after 6 hours as the “Late” period.  “＞24 hours” was defined as more than 24 hours postoperatively.  “Overall” period was defined as the whole recorded time in articles.  Generally, data extraction was performed directly from the articles. For the calculation of PONV, some data were obtained from the “complete response (CR)”.^14, 30, 33, 36^ Some data regarding the use of rescue medication were derived from the failure rates reported in the literature.^30^ |  |
|  | 13d | Describe any methods used to synthesize results and provide a rationale for the choice(s). If meta-analysis was performed, describe the model(s), method(s) to identify the presence and extent of statistical heterogeneity, and software package(s) used.  The R software (version 4.2.1) and STATA 17 were used for this network meta-analysis to compare treatments using a frequentist approach. The conclusions from direct and indirect comparisons were obtained. Heterogeneity in indirect comparison meta-analysis was quantified using the I^2^ statistic, while the Q statistic was used to assess inconsistencies between direct and indirect effects. A random effects model was applied to pool data. |  |
|  | 13e | Describe any methods used to explore possible causes of heterogeneity among study results (e.g. subgroup analysis, meta-regression).  The transitivity assumption was assessed by comparing the distribution of potential effect modifiers across comparisons, such as publication years，mean age, weight, duration of surgery and anesthesia, etc. |  |
|  | 13f | Describe any sensitivity analyses conducted to assess robustness of the synthesized results.  Sensitivity analyses were performed by separately excluding studies involving open surgery, non-opioid postoperative anesthesia, spinal anesthesia or propofol maintenance anesthesia.  Six studies reported open or mostly open surgery, and the pooled estimates were not significantly impacted by the exclusion of data from these studies (Supplement 7).  In six studies, non-opioid analgesia was used in postoperative analgesia, and removing these data did not significantly alter the pooled estimates. (Supplement 8).  We excluded one study that used spinal anesthesia and the results did not significantly deviate the pooled estimates (Supplement 9).  Two studies that maintained anesthesia with propofol and exclusion of data from these studies did not significantly influence the pooled estimates compared to the overall analysis (Supplement 10). |  |
| Reporting bias assessment | 14 | Describe any methods used to assess risk of bias due to missing results in a synthesis (arising from reporting biases).  Egger’s test and funnel plots were employed to assess publication bias in studies with 10 or more trials. |  |
| Certainty assessment | 15 | Describe any methods used to assess certainty (or confidence) in the body of evidence for an outcome.  Two investigators independently assessed the study quality and performed data extraction. When consensus could not be reached, the third author resolved the discrepancies.  21 trials were included for quality evaluation, which used Revman 5.3 (Figure 2). |  |
| **RESULTS** | | |  |
| Study selection | 16a | Describe the results of the search and selection process, from the number of records identified in the search to the number of studies included in the review, ideally using a flow diagram.  A comprehensive search yielded a total of 1060 documents. After the elimination of duplicates and screening of articles that did not meet the inclusion criteria based on their titles and abstracts, 103 studies underwent a full-text review. Ultimately, 21 studies were deemed eligible for inclusion in this network meta-analysis (Figure 1).^14, 16, 19-37^ The collective dataset encompassed 1959 female participants, with ages ranging from 26.4 to 52.5 years. All included studies were published between 1999 and 2023. |  |
|  | 16b | Cite studies that might appear to meet the inclusion criteria, but which were excluded, and explain why they were excluded.  We have not encountered such a situation. |  |
| Study characteristics | 17 | Cite each included study and present its characteristics.  Table 1 Characteristics of the included RCTs   \| Number \| Year \| Test type \| Included population \| ASA \| Anesthesia methods (major maintained medicine) \| Surgical methods \| Intervention (The number of each group) \| Postoperative analgesia \| Period definition (h) \| \| \| Outcome \| \| --- \| --- \| --- \| --- \| --- \| --- \| --- \| --- \| --- \| --- \| --- \| --- \| --- \| \| Acute \| Late \| >24 hours \| \| Das S 2022 \| 2022 \| RCT \| 100 female patients，aged 18-60 years，gynecological surgery. \| I-II \| General anesthesia  (sevoflurane) \| Laparoscopic  surgery \| Ramosteron (50) Ondansetron (50) \| Diclofenac paracetamol \| 0-6 \| 6-24 \| / \| ⑦⑧⑪⑫⑬⑭⑯⑰ \| \| Bandyopadhyay D 2022 \| 2022 \| RCT \| 90 female patients, aged 35-60 years,  gynecological surgery. \| I-II \| General anesthesia \| / \| Granisetron (30)  Palanosetron (30)  Ondansetron (30) \| Tramadol  Diclofenac \| 0-4 \| 4-24 \| / \| ①②④⑤⑦⑧⑩⑪⑫⑬⑭⑯⑰ \| \| Balyan R 2022 \| 2022 \| RCT \| 130 female patients, aged 18-70 years,  gynecological surgery. \| I-II \| General anesthesia  (sevoflurane) \| Laparoscopic  surgery \| Ondansetron (65) Palonosetron (65) \| Morphine Paracetamol Diclofenac \| 0-2 \| 2-24 \| 24-48 \| ①②③④⑤⑥⑦⑧⑨⑪⑬⑭⑮ \| \| Yadav M 2022 \| 2022 \| RCT \| 90 female patients, ≥21 years old,  gynecological surgery. \| I-II \| General anesthesia  (sevoflurane) \| Laparoscopic  surgery \| Palonosetron (45) Ondansetron (45) \| Fentanyl \| 0-2 \| 6-24 \| / \| ①②④⑤⑦⑧⑩⑪⑫⑯⑰ \| \| Lee WS 2015 \| 2015 \| RCT \| 105 female patients, laparoscopic hysterectomy. \| I-II \| General anesthesia  (sevoflurane) \| Laparoscopic  surgery \| Palonosetron (35) Ramosetron (35) Granisetron (35) \| Diclofenac \| 0-6 \| 6-24 \| 24-48 \| ①②③④⑤⑥⑫⑬⑭⑮ \| \| Kim SH 2015 \| 2015 \| RCT \| 200 Patients, gynecological surgery. \| / \| General anesthesia  (sevoflurane) \| Laparoscopic  surgery \| Palonosetron (44)  Ramosetron (44) \| Ketorolac Fentanyl \| Before discharge from  PACU \| 24 h after  discharge from PACU \| 48 h after discharge from PACU \| ⑦⑧⑨ \| \| Park SK 2013 \| 2013 \| RCT \| 100 female patients, ≥20 years old,  gynecological surgery. \| I-II \| General anesthesia  (sevoflurane) \| Laparoscopic  surgery \| Ramosetron (50)  Palonosetron (50) \| Fentanyl \| 0-6 \| 6-24 \| 24-48 \| ①②③④⑤⑥⑦⑧⑨⑩⑪⑫⑯⑰ \| \| Kim YY 2013 \| 2013 \| RCT \| 100 female patients, ≥18 years old,  gynecological surgery. \| I-II \| General anesthesia  (sevoflurane) \| Laparoscopic  surgery \| Ondansetron (50) Palonosetron (50) \| Fentanyl Ketorolac \| 0-2 \| 2-24 \| 24-72 \| ①②③④⑤⑥⑦⑧⑨⑩⑪⑫⑬⑭⑮⑯⑰ \| \| Daria U 2012 \| 2012 \| RCT \| 180 female patients, aged 18-55 years, gynecological surgery. \| I-II \| General anesthesia \| Laparoscopic surgery \| Ondasetron (30)  Granisetron (30) \| / \| 0-6 \| / \| / \| ⑦⑪ \| \| Park SK 2011 \| 2011 \| RCT \| Patients ≥ 21 years old,  gynecological  surgery. \| I-II \| General anesthesia  (sevoflurane) \| Laparoscopic  surgery \| Ondansetron (45) Palonosetron (45) \| Fentanyl \| 0-2 \| 6-24 \| / \| ①②④⑤⑦⑧⑩⑪⑫⑯⑰ \| \| Bajwa SS 2011 \| 2011 \| RCT \| 60 Patients, aged 25-40 years, ligation surgery. \| I-II \| general anesthesia  (propofol and  halothane) \| Laparoscopic surgery \| Ondansetron (30) Palonosetron (30) \| Diclofenac \| 0-6 \| 6-12 \| 24-72 \| ①②③④⑤⑥⑪⑯⑰ \| \| Kim SI 2009 \| 2009 \| RCT \| 162 female patients, aged 21-71 years,  gynecological surgery. \| / \| General anesthesia  (sevoflurane) \| Laparoscopic and open surgery \| Ramosetron (54) Ondansetron (54) \| Fentanyl \| 0-6 \| 6-24 \| / \| ①②④⑤⑩⑪⑫⑬⑭⑯⑰ \| \| Bhatia N 2008 \| 2008 \| RCT \| 120 female patients, aged 18-40 years, gynecological surgery. \| I-II \| General anesthesia  (propofol) \| Laparoscopic surgery \| Ondansetron (30) Granisetron (30) \| Ketorolac  tromethamine \| / \| / \| / \| ⑩⑪⑫⑯⑰ \| \| Yun MJ 2010 \| 2010 \| RCT \| 98 female patients, aged 20-65 years,  gynecological surgery. \| I-II \| General anesthesia  (sevoflurane) \| Laparoscopic surgery \| Ondansetron (49) Azasetron (49) \| Nalbuphine Ketorolac \| 0-6 \| 6-12 \| 24-48 \| ①②③④⑤⑥⑪⑫⑬⑭⑮⑯⑰ \| \| Ekinci O 2011 \| 2011 \| RCT \| Female patients, aged 20-72 years, total abdominal  hysterectomy. \| I-Ⅲ \| General anesthesia  (sevoflurane) \| Open surgery \| Tropisetron (20)  Ondansetron (20) \| Dipyrone \| / \| / \| / \| ⑩⑪⑰ \| \| Patil SB 2023 \| 2023 \| RCT \| 100 female patients, aged 23-65 years, hysterectomy. \| / \| Spinal anesthesia \| / \| Ondansetron (50)  Ramosetron (50) \| / \| 0-6 \| 6-24 \| / \| ①②③④⑤⑥⑩⑪⑫⑬⑭⑮ \| \| Tarigonda S 2021 \| 2021 \| RCT \| Female patients,  aged 35 -70 years, abdominal hysterectomy. \| I-II \| General anesthesia  (sevoflurane) \| Open surgery \| Ondansetron (30)  Ramosetron (30)  Palonosetron (30) \| / \| 0-6 \| 12-24 \| / \| ①②④⑪⑬ \| \| Tsui SL 1999 \| 1999 \| RCT \| 121 women, gynecological  Laparotomy. \| I-II \| General anesthesia  (sevoflurane) \| Open surgery \| Tropisetron (37)  Ondansetron (39) \| Morphine \| 0-12 \| / \| / \| ⑩⑬⑯⑰ \| \| Bridges JD 2006 \| 2006 \| RCT \| 194 female patients, aged 18-78 years,  gynecological and breast surgery. \| / \| Most general anesthesia  (sevoflurane) \| Laparoscopic and open surgery \| Dolasetron (66)  Granisetron (62)  Ondansetron (66) \| / \| 0-6 \| 6-24 \| / \| ⑦⑧⑪⑫ \| \| Lee JW 2011 \| 2011 \| RCT \| 120 women, aged  18-60 years, abdominal hysterectomy. \| I-II \| General anesthesia  (sevoflurane) \| Open surgery \| Ramosetron (60)  Ondansetron (60) \| Fentanyl  Ketorolac \| 0-2 \| 2-24 \| 24-48 \| ①②③④⑤⑥⑦⑧⑨⑪⑬⑭⑮ \| \| Sumitha CS 2021 \| 2021 \| RCT \| 120 female patients, aged 18-60 years,  lower abdominal surgery. \| I-II \| General anesthesia (halothane) \| Open surgery \| Granisetron (30)  Ondansetron (30) \| Paracetamol Ketorolac \| / \| 6-24 \| / \| ②⑤⑧⑪⑫⑯⑰ \|   ①Acute nausea; ②Late nausea; ③>24h nausea; ④Overall nausea; ⑤Acute vomiting; ⑥Late vomiting; ⑦>24h vomiting; ⑧Overall vomiting; ⑨Acute PONV; ⑩Late PONV; ⑪>24h PONV; ⑫Overall PONV; ⑬Acute rescue medicine; ⑭Late rescue medicine; ⑮>24h rescue medicine; ⑯Overall rescue medicine; ⑰Adverse reaction |  |
| Risk of bias in studies | 18 | Present assessments of risk of bias for each included study. 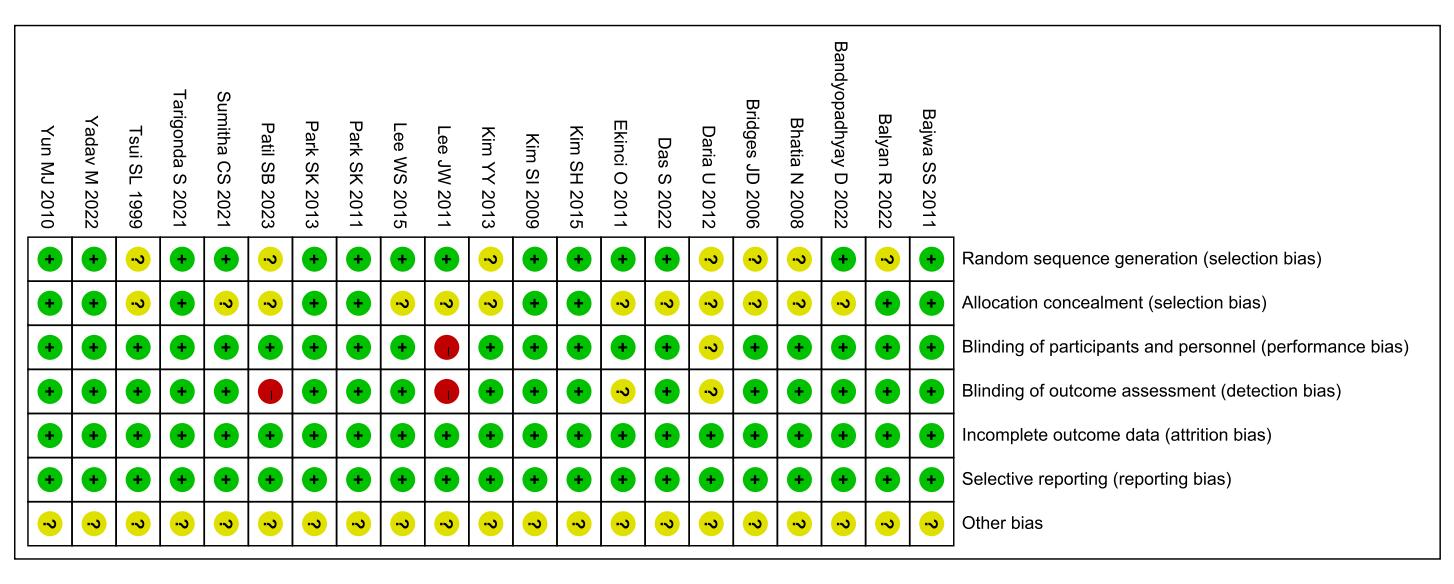 |  |
| Results of individual studies | 19 | For all outcomes, present, for each study: (a) summary statistics for each group (where appropriate) and (b) an effect estimate and its precision (e.g. confidence/credible interval), ideally using structured tables or plots.  For “Acute nausea”, 13 RCTs (Supplement 5) included in the analysis showed that Palonosetron was significantly more effective than Ondansetron (RR1.48, 95%CI 1.17-1.87) and Ramosetron (RR0.77, 95%CI 0.60-0.99) (Table 2). Based on the P-score, Palonosetron was associated with the highest efficacy, followed by Granisetron, Azasetron, Ramosetron, and Ondansetron (Table 3).  For “Late nausea”, the analysis of 14 RCTs (Supplement 5) found no statistical significance among all 5-HT_3_ antagonists (Table 2), with the P-score ranking consistent with that for “Acute nausea” (Table 3).  In the case of “>24 hours nausea”, Ramosetron outperformed Ondansetron in 8 RCTs (Supplement 5) (RR2.09, 95%CI 1.02-4.28) (Table 2). According to the P-score, Ramosetron and Azasetron were associated with the best and worst effects, respectively (Table 3).  Based on 12 RCTs (Supplement 5) included in “Overall nausea”, Palonosetron was significantly more effective than Ondansetron (RR1.47, 95%CI 1.09-1.99) (Table 2). According to the P-score, Granisetron emerged as the best choice, followed by Palanosetron, Tropisetron, Ramosetron, Azasetron, and Ondansetron (Table 3).  **Table 2 Network calculation of nausea**   \| Acute nausea \| Azasetron \| . \| 0.82 (0.37; 1.80) \| . \| . \| \| --- \| --- \| --- \| --- \| --- \| --- \| \| 1.40 (0.41; 4.74) \| Granisetron \| 0.20 (0.01; 4.00) \| 0.84 (0.30; 2.41) \| 0.83 (0.28; 2.48) \| \| 0.82 (0.37; 1.80) \| 0.58 (0.23; 1.48) \| Ondansetron \| 1.47 (1.13; 1.92) \| 1.14 (0.89; 1.46) \| \| 1.21 (0.53; 2.75) \| 0.87 (0.34; 2.18) \| 1.48 (1.17; 1.87) \| Palanosetron \| 0.82 (0.63; 1.09) \| \| 0.93 (0.41; 2.11) \| 0.67 (0.26; 1.68) \| 1.14 (0.91; 1.43) \| 0.77 (0.60; 0.99) \| Ramosetron \| \| Late nausea \| Azasetron \| . \| 0.88 (0.42; 1.81) \| . \| . \| \| 1.29 (0.41; 4.01) \| Granisetron \| 0.41 (0.12; 1.41) \| 1.71 (0.50; 5.87) \| 1.00 (0.25; 3.93) \| \| 0.88 (0.42; 1.81) \| 0.68 (0.28; 1.63) \| Ondansetron \| 1.49 (1.06; 2.09) \| 0.85 (0.53; 1.37) \| \| 1.17 (0.53; 2.58) \| 0.91 (0.37; 2.20) \| 1.33 (0.98; 1.82) \| Palanosetron \| 1.14 (0.63; 2.04) \| \| 0.96 (0.42; 2.19) \| 0.75 (0.30; 1.83) \| 1.10 (0.75; 1.61) \| 0.82 (0.54; 1.24) \| Ramosetron \| \| >24h nausea \| Azasetron \| . \| 1.50 (0.57; 3.92) \| . \| . \| \| 3.93 (0.33; 46.66) \| Granisetron \| . \| 0.50 (0.05; 5.28) \| 1.00 (0.06; 15.40) \| \| 1.50 (0.57; 3.92) \| 0.38 (0.04; 3.73) \| Ondansetron \| 1.29 (0.79; 2.11) \| 6.37 (1.46; 27.75) \| \| 2.20 (0.75; 6.40) \| 0.56 (0.06; 5.23) \| 1.47 (0.92; 2.34) \| Palanosetron \| 1.14 (0.59; 2.20) \| \| 3.13 (0.94; 10.38) \| 0.80 (0.08; 7.66) \| 2.09 (1.02; 4.28) \| 1.42 (0.78; 2.61) \| Ramosetron \| \| Overall nausea \| Azasetron \| . \| 0.84 (0.46;1.52) \| . \| . \| \| 2.22 (0.79;6.23) \| Granisetron \| 0.35 (0.15; 0.82) \| 3.00 (0.32; 28.31) \| . \| \| 0.84 (0.46;1.52) \| 0.38 (0.16; 0.88) \| Ondansetron \| 1.60 (1.14;2.25) \| 1.05 (0.68; 1.62) \| \| 1.24 (0.63;2.41) \| 0.56 (0.23; 1.36) \| 1.47 (1.09; 1.99) \| Palanosetron \| 0.97 (0.59; 1.59) \| \| 1.01 (0.51;2.02) \| 0.46 (0.18; 1.13) \| 1.20 (0.85; 1.71) \| 0.82 (0.57; 1.18) \| Ramosetron \| \| 1.02 (0.46;2.29) \| 0.46 (0.17; 1.26) \| 1.22 (0.71; 2.10) \| 0.83 (0.44; 1.54) \| 1.01 (0.53; 1.94) \| |  |
| Results of syntheses | 20a | For each synthesis, briefly summarise the characteristics and risk of bias among contributing studies.  We observed low heterogeneity between studies in this network (Supplement 2) and no significant inconsistencies among direct and indirect comparisons (Supplement 3).  The funnel plot of the studies with 10 or more trials all demonstrated a fundamental symmetry, suggesting the absence of publication bias in the reviewed articles (Supplement 4). |  |
|  | 20b | Present results of all statistical syntheses conducted. If meta-analysis was done, present for each the summary estimate and its precision (e.g. confidence/credible interval) and measures of statistical heterogeneity. If comparing groups, describe the direction of the effect.  For “Acute nausea”, 13 RCTs (Supplement 5) included in the analysis showed that Palonosetron was significantly more effective than Ondansetron (RR1.48, 95%CI 1.17-1.87) and Ramosetron (RR0.77, 95%CI 0.60-0.99) (Table 2). In the case of “>24 hours nausea”, Ramosetron outperformed Ondansetron in 8 RCTs (Supplement 5) (RR2.09, 95%CI 1.02-4.28) (Table 2). Based on 12 RCTs (Supplement 5) included in “Overall nausea”, Palonosetron was significantly more effective than Ondansetron (RR1.47, 95%CI 1.09-1.99) (Table 2). In the analysis of “Acute vomiting”, there was a significant difference in Palonosetron’s effectiveness over Ondansetron (RR2.04, 95%CI 1.14-3.66) based on 13 RCTs (Supplement 5). For “Late vomiting”, 13 RCTs (Supplement 5) revealed that Granisetron (RR0.28, 95%CI 0.08-0.96) and Palanosetron (RR1.90, 95%CI 1.19-3.02) were significantly more effective than Ondansetron (Table 4). Palonosetron was superior to Ondansetron (RR1.34, 95%CI 1.02-1.77) (Table 5) in the treatment of “Late PONV”, according to 11 RCTs (Supplement 5). For “Overall PONV”, 14 RCTs (Supplement 5) included，significant difference was noted between Palanosetron and Ondansetron (RR1.25, 95%CI 1.00-1.58) (Table 5). For “Late rescue medicine”, 9 RCTs (Supplement 5) demonstrated that the effectiveness of Palanosetron was significantly higher than that of Ondansetron (RR1.77, 95%CI 1.21-2.59) (Table 6). Among six RCTs (Supplement 5) that evaluated “>24 hours rescue medicine”, Palanosetron performed significantly better than Ondansetron (RR2.47, 95%CI 1.08-5.65) (Table 6).  **Table 3 P-score of outcome measures**   \|  \| Palanosetron \| Granisetron \| Ramosetron \| Ondansetron \| Azasetron \| Tropisetron \| Dolasetron \| \| --- \| --- \| --- \| --- \| --- \| --- \| --- \| --- \| \| Acute nausea \| 0.7590 \| 0.7506 \| 0.3796 \| 0.1417 \| 0.4691 \|  \|  \| \| Late nausea \| 0.7138 \| 0.7000 \| 0.3945 \| 0.2264 \| 0.4653 \|  \|  \| \| >24h nausea \| 0.5756 \| 0.7327 \| 0.8101 \| 0.2694 \| 0.1121 \|  \|  \| \| Overall nausea \| 0.6818 \| 0.9424 \| 0.4062 \| 0.1379 \| 0.4026 \| 0.4292 \|  \| \| Acute vomiting \| 0.6130 \| 0.7129 \| 0.3550 \| 0.0882 \| 0.7310 \|  \|  \| \| Late vomiting \| 0.7188 \| 0.9139 \| 0.4606 \| 0.2281 \|  \| 0.1785 \|  \| \| >24h vomiting \| 0.7953 \| 0.6329 \| 0.6203 \| 0.2859 \| 0.1656 \|  \|  \| \| Overall vomiting \| 0.6918 \| 0.8543 \| 0.1587 \| 0.2547 \| 0.5730 \| 0.4675 \|  \| \| Acute PONV \| 0.7904 \| 0.6574 \| 0.4813 \| 0.3950 \|  \|  \| 0.1759 \| \| Late PONV \| 0.7811 \| 0.5752 \| 0.4975 \| 0.2188 \|  \|  \| 0.4275 \| \| >24h PONV \| 0.6486 \|  \| 0.7423 \| 0.1091 \|  \|  \|  \| \| Overall PONV \| 0.7029 \| 0.6963 \| 0.5156 \| 0.2195 \| 0.7205 \|  \| 0.1452 \| \| Acute rescue medicine \| 0.3804 \| 0.7263 \| 0.6276 \| 0.3531 \| 0.4125 \|  \|  \| \| Late rescue medicine \| 0.7110 \| 0.9163 \| 0.3769 \| 0.2054 \| 0.2904 \|  \|  \| \| >24h rescue medicine \| 0.6322 \| 0.6467 \| 0.8098 \| 0.2602 \| 0.1510 \|  \|  \| \| Overall rescue medicine \| 0.4545 \| 0.8902 \| 0.5639 \| 0.1600 \|  \| 0.4315 \|  \| \| Adverse reaction \| 0.5890 \| 0.2980 \| 0.5040 \| 0.4476 \| 0.5626 \| 0.5989 \|  \| |  |
|  | 20c | Present results of all investigations of possible causes of heterogeneity among study results.  We observed low heterogeneity between studies in this network (Supplement 2).  **Supplement 2 The heterogeneity within the network**   \|  \| tau^^2^ \| tau \| I^^2^ \| \| --- \| --- \| --- \| --- \| \| Acute nausea \| 0 \| 0 \| 0% [0.0%; 56.6%] \| \| Late nausea \| 0.0450 \| 0.2121 \| 20% [0.0%; 57.2%] \| \| >24h nausea \| 0.0031 \| 0.0553 \| 0.7% [0.0%; 74.8%] \| \| Overall nausea \| 0.0450 \| 0.2122 \| 34.5% [0.0%; 69.8%] \| \| Acute vomiting \| 0 \| 0 \| 0% [0.0%; 56.6%] \| \| Late vomiting \| 0 \| 0 \| 0% [0.0%; 58.3%] \| \| >24h vomiting \| 0 \| 0 \| 0% [0.0%; 74.6%] \| \| Overall vomiting \| 0.1920 \| 0.4382 \| 38.1% [0.0%; 70.5%] \| \| Acute PONV \| 0.0583 \| 0.2415 \| 29.6% [0.0%; 66.3%] \| \| Late PONV \| 0.0311 \| 0.1764 \| 24% [0.0%; 63.1%] \| \| >24h PONV \| 0.0543 \| 0.2331 \| 25.3% [0.0%; 71.3%] \| \| Overall PONV \| 0.0316 \| 0.1777 \| 32% [0.0%; 64.1%] \| \| Acute rescue medicine \| 0 \| 0 \| 0% [0.0%; 62.4%] \| \| Late rescue medicine \| 0 \| 0 \| 0% [0.0%; 67.6%] \| \| >24h rescue medicine \| 0 \| 0 \| 0% [0.0%; 84.7%] \| \| Overall rescue medicine \| 0.0892 \| 0.2987 \| 25.4% [0.0%; 66.1%] \| \| Adverse reaction \| 0.0435 \| 0.2085 \| 30.1% [0.0%; 62.4%] \| |  |
|  | 20d | Present results of all sensitivity analyses conducted to assess the robustness of the synthesized results.  Six studies reported open or mostly open surgery, and the pooled estimates were not significantly impacted by the exclusion of data from these studies (Supplement 7).  In six studies, non-opioid analgesia was used in postoperative analgesia, and removing these data did not significantly alter the pooled estimates. (Supplement 8).  We excluded one study that used spinal anesthesia and the results did not significantly deviate the pooled estimates (Supplement 9).  Two studies that maintained anesthesia with propofol and exclusion of data from these studies did not significantly influence the pooled estimates compared to the overall analysis (Supplement 10). |  |
| Reporting biases | 21 | Present assessments of risk of bias due to missing results (arising from reporting biases) for each synthesis assessed.  We did not meet this situation. 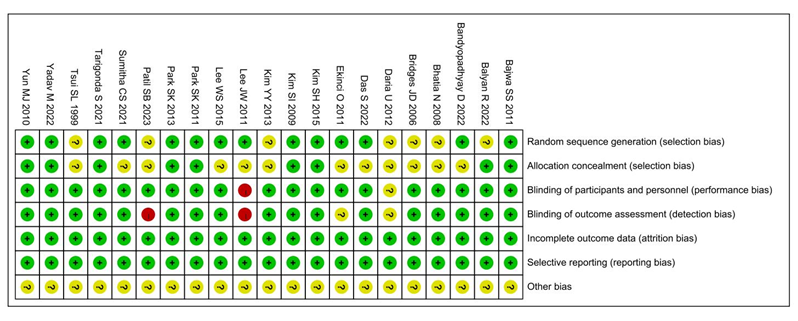 |  |
| Certainty of evidence | 22 | Present assessments of certainty (or confidence) in the body of evidence for each outcome assessed.  no significant inconsistencies among direct and indirect comparisons (Supplement 3).  **Supplement 3**  **Inconsistency between studies**   \|  \| Q \| df \| p-value \| tau.within \| tau^2^.within \| \| --- \| --- \| --- \| --- \| --- \| --- \| \| Acute nausea \| 3.11 \| 6 \| 0.7947 \| 0.1222 \| 0.0149 \| \| Late nausea \| 8.46 \| 7 \| 0.2939 \| 0.1194 \| 0.0142 \| \| >24h nausea \| 3.16 \| 2 \| 0.2059 \| 0 \| 0 \| \| Overall nausea \| 4.66 \| 3 \| 0.1987 \| 0.1622 \| 0.0263 \| \| Acute vomiting \| 4.98 \| 6 \| 0.5459 \| 0 \| 0 \| \| Late vomiting \| 2.93 \| 5 \| 0.7115 \| 0 \| 0 \| \| >24h vomiting \| 0.66 \| 2 \| 0.7199 \| 0 \| 0 \| \| Overall vomiting \| 2.69 \| 3 \| 0.4415 \| 0.4520 \| 0.2043 \| \| Acute PONV \| 2.36 \| 4 \| 0.6698 \| 0.3777 \| 0.1427 \| \| Late PONV \| 3.56 \| 4 \| 0.4690 \| 0.1932 \| 0.0373 \| \| >24h PONV \| 2.84 \| 1 \| 0.0921 \| 0 \| 0 \| \| Overall PONV \| 10.35 \| 8 \| 0.2410 \| 0.1408 \| 0.0198 \| \| Acute rescue medicine \| 0.78 \| 5 \| 0.9783 \| 0 \| 0 \| \| Late rescue medicine \| 3.07 \| 3 \| 0.3814 \| 0 \| 0 \| \| >24h rescue medicine \| 0.13 \| 1 \| 0.7179 \| 0 \| 0 \| \| Overall rescue medicine \| 6.97 \| 3 \| 0.0730 \| 0 \| 0 \| \| Adverse reaction \| 2.64 \| 5 \| 0.7549 \| 0.2792 \| 0.0780 \| |  |
| **DISCUSSION** | | |  |
| Discussion | 23a | Provide a general interpretation of the results in the context of other evidence.  In our study, after gynecological surgery, Palonosetron demonstrates superior efficacy compared to Ondansetron, mainly had a significant difference in “Acute nausea”, “Overall nausea”, “Acute vomiting”, “Late vomiting”, “Late PONV”, “Overall PONV”, “Late rescue medicine” and “> 24 hours rescue medicine”. Many prior studies indicated that Palonosetron generally demonstrated significantly superior efficacy in the prevention of postoperative nausea and vomiting compared to Ondansetron.^38-43^ However, a meta-analysis showed that, in the laparoscopic surgery, there was no significant difference between Palonosetron and Ondansetron in PONV within the initial 24 hours and more significantly effective than Ondansetron in the control of vomiting.^13^ Another study suggested that the rates of nausea and vomiting in patients administered Palonosetron were not statistically different from those who received Ondansetron during the first 24 hours following surgery. Furthermore, no significant disparities were detected in the severity of nausea or the necessity for rescue anti-emetic medication between the two groups.^44^ Our research findings align with the majority of prior studies: the efficacy of Palonosetron significantly surpasses that of Ondansetron. If a choice must be made between the two, Palonosetron is the preferred option.  Our study found that there were no significant differences between Palonosetron and Granisetron for PONV prophylaxis in various indicators including nausea, vomiting, PONV, rescue medicine and adverse. Moreover, in P-score rating system, both Palonosetron and Granisetron generally receive high rankings, with neither showing superior superiority. But a meta-analysis showed that Palonosetron was significantly more effective in preventing early vomiting, PONV in early and delayed phase.^45^ |  |
|  | 23b | Discuss any limitations of the evidence included in the review.  Due to the limited number of studies on Tropisetron, Azasetron, and Dolasetron within our research scope, this has resulted in less stable conclusions. |  |
|  | 23c | Discuss any limitations of the review processes used.  Only PubMed, Embase, the Cochrane Library, and Web of Science were searched, and only English-language literatures were included. |  |
|  | 23d | Discuss implications of the results for practice, policy, and future research.  Based on the conclusions above, we proposed the following treatment plan for the clinical application of 5-HT_3_ receptor antagonists to prevent PONV in gynecological surgery:  Ⅰ. The efficacy of Granisetron was comparable to that of Palonosetron in preventing PONV in gynecological surgery. From a cost-effectiveness perspective, Granisetron may be an optimal alternative to Palonosetron for antiemetic use.  Ⅱ. Palonosetron’s effect was significantly superior to Ondansetron. If only these two drugs were available for selection, Palonosetron was recommended for the prevention of PONV in gynecological surgery.  Future research endeavors should prioritize conducting more high-quality studies to enhance the stability and reliability of the conclusions draw |  |
| **OTHER INFORMATION** | | |  |
| Registration and protocol | 24a | Provide registration information for the review, including register name and registration number, or state that the review was not registered.  This systematic review and network meta-analysis was registered with the International Prospective Registry of Systematic Reviews (PROSPERO) under the identifier CRD42024496745. |  |
|  | 24b | Indicate where the review protocol can be accessed, or state that a protocol was not prepared.  We did not possess a formal review protocol. |  |
|  | 24c | Describe and explain any amendments to information provided at registration or in the protocol.  No amendments. |  |
| Support | 25 | Describe sources of financial or non-financial support for the review, and the role of the funders or sponsors in the review.  2021 Clinical Research Funds of Shandong Medical Association-Qilu Specialized Funding |  |
| Competing interests | 26 | Declare any competing interests of review authors.  The authors declare that they have no conflict of interest. |  |
| Availability of data, code and other materials | 27 | Report which of the following are publicly available and where they can be found: template data collection forms; data extracted from included studies; data used for all analyses; analytic code; any other materials used in the review.  We have yet to determine the appropriate method for disseminating the materials. |  |

*From:*  Page MJ, McKenzie JE, Bossuyt PM, Boutron I, Hoffmann TC, Mulrow CD, et al. The PRISMA 2020 statement: an updated guideline for reporting systematic reviews. BMJ 2021;372:n71. doi: 10.1136/bmj.n71
