# Supplementary material for: 5‐HT3 receptor antagonists for preventing postoperative nausea and vomiting after gynecological surgery: A systematic review and network meta‐analysis
Source: Int J Gynaecol Obstet. 2025 May 9;171(1):177–89. doi: 10.1002/ijgo.70197 (PMC12447676; doi:10.1002/ijgo.70197)
Supplement: Supplementary file 12 — Data S12. [file IJGO-171-177-s006.docx]

Data S12 Adverse reactions categorization

| **5-HT_3_ antagonists (number)** | **Headache**  **Number（rate）** | **Dizziness Number（rate）** | **Constipation Number（rate）** | **Drowsiness Number（rate）** | **Hypotension Number（rate）** | **Rashes or pruritus Number（rate）** | **Sedation Number（rate）** | **Myalgia Number（rate）** | **Pain Number（rate）** | **Bradycardia Number（rate）** | **Dyspepsia Number（rate）** | **Vertigo Number（rate）** | **Anxiety Number（rate）** | **Dry mouth Number（rate）** |
| --- | --- | --- | --- | --- | --- | --- | --- | --- | --- | --- | --- | --- | --- | --- |
| **Ondansetron (668)** | **81(12.1%)** | **58(8.7%)** | **12(1.8%)** | **25(3.7%)** |  | **2(0.3%)** | **7(1.0%)** | **3(0.4%)** | **9(1.3%)** |  | **2(0.3%)** |  | **2(0.3%)** |  |
| **Ramosetron (294)** | **27（9.2%）** | **18(6.1%)** | **3(1.0%)** | **5(1.7%)** |  | **1(0.3%)** |  | **3(1.0%)** |  | **1(0.3%)** | **2(0.7%)** | **1(0.3%)** |  |  |
| **Granisetron (120)** | **13(10.8%)** | **13(10.8%)** |  |  | **1(0.8%)** | **1(0.8%)** | **2(1.7%)** |  | **4(3.3%)** |  |  |  |  | **1(0.8%)** |
| **Palanosetron (345)** | **34(9.9%)** | **36(10.4%)** | **28(8.1%)** | **30(8.7%)** |  |  |  | **2(0.6%)** | **3(0.9%)** |  | **3(0.9%)** |  | **1(0.3%)** |  |
| **Azasetron (49)** | **4(8.2%)** | **4(8.2%)** | **1(2.0%)** |  |  |  | **5(10.2%)** | **2(4.1%)** |  |  |  |  |  |  |
